# GAD-PVI: A General Accelerated Dynamic-Weight Particle-Based Variational Inference Framework

**DOI:** 10.3390/e26080679

**Published:** 2024-08-11

**Authors:** Fangyikang Wang, Huminhao Zhu, Chao Zhang, Hanbin Zhao, Hui Qian

**Affiliations:** College of Computer Science and Technology, Zhejiang University, Hangzhou 310058, China; wangfangyikang@zju.edu.cn (F.W.); zhuhuminhao@zju.edu.cn (H.Z.); zhaohanbin@zju.edu.cn (H.Z.); qianhui@zju.edu.cn (H.Q.)

**Keywords:** variational inference, Bayesian sampling, probability gradient flow

## Abstract

Particle-based Variational Inference (ParVI) methods have been widely adopted in deep Bayesian inference tasks such as Bayesian neural networks or Gaussian Processes, owing to their efficiency in generating high-quality samples given the score of the target distribution. Typically, ParVI methods evolve a weighted-particle system by approximating the first-order Wasserstein gradient flow to reduce the dissimilarity between the particle system’s empirical distribution and the target distribution. Recent advancements in ParVI have explored sophisticated gradient flows to obtain refined particle systems with either accelerated position updates or dynamic weight adjustments. In this paper, we introduce the semi-Hamiltonian gradient flow on a novel Information–Fisher–Rao space, known as the SHIFR flow, and propose the first ParVI framework that possesses both accelerated position update and dynamical weight adjustment simultaneously, named the General Accelerated Dynamic-Weight Particle-based Variational Inference (GAD-PVI) framework. GAD-PVI is compatible with different dissimilarities between the empirical distribution and the target distribution, as well as different approximation approaches to gradient flow. Moreover, when the appropriate dissimilarity is selected, GAD-PVI is also suitable for obtaining high-quality samples even when analytical scores cannot be obtained. Experiments conducted under both the score-based tasks and sample-based tasks demonstrate the faster convergence and reduced approximation error of GAD-PVI methods over the state-of-the-art.

## 1. Introduction

Bayesian inference is an active area in modern machine learning that provides powerful tools for modeling unknown distributions and reasoning under uncertainty. Its applications range from natural language processing [1,2,3] and image processing [4,5,6,7] to knowledge representation [8,9,10,11]. The core of Bayesian inference is to estimate the target posterior distribution given the data.

Markov Chain Monte Carlo (MCMC) methods have been extensively employed in Bayesian inference, serving as a cornerstone for sampling from complex probability distributions. These methods rely on constructing a Markov chain that has the desired distribution as its equilibrium distribution. Through iterative sampling, MCMC methods facilitate the exploration of the sample space, providing a robust framework for estimating the posterior distributions critical to Bayesian approaches [12,13,14]. In the MCMC literature, acceleration methods such as Hamilton Monte Carlo (HMC) and underdamped Langevin dynamics, which optimize sampling efficiency and convergence speed, have been widely studied [15,16]. Furthermore, Sequential Monte Carlo (SMC) methods use dynamic weight techniques combined with resampling to tackle particle degeneracy and have been integrated with Hamiltonian Monte Carlo (HMC) to boost convergence [17,18,19].

Recently, Particle-based Variational Inference (ParVI) methods have gained significant attention in the Bayesian inference literature owing to their effectiveness in providing approximations of the target posterior distribution π [20,21,22,23,24,25]. The essence of ParVI lies in deterministically evolving a system of finite particles iteratively and approximating the target distribution with this set of finite particles. Compared to traditional MCMC methods, PVI introduces repulsive forces among particles. This fundamental addition prevents the particles from collapsing or degenerating, ensuring a more robust distribution coverage. This feature is particularly beneficial in high-dimensional spaces, where MCMC methods may encounter difficulties due to particle degeneration. Typically, the rule of updating the particles is designed by simulating the probability space gradient flow of certain dissimilarity functional F(μ):=D(μ|π) vanishing at μ=π. Since the seminal work on Stein Variational Gradient Descent (SVGD) [20] to the subsequent BLOB method [26], the Gradient Flow with Smoothed Density (GFSD) method [27], and the Kernel Setin Discrepancy Descent (KSDD) method [28], various effective ParVI methods have been proposed that adopt different dissimilarities or empirical approaches to simulate the probability gradient flow. (See Table 1).

However, these classical ParVIs only focus on simulating the *first-order* gradient flow in the *Wasserstein* space. To improve the efficiency of ParVIs, recent works explore different aspects of the underlying geometry structures in the probability space and design two types of refined particle systems with either *accelerated position update* or *dynamic weight adjustment*.

- *Accelerated position update.* By considering the second-order information of the Wasserstein probability space, different accelerated position update strategies have been proposed [27,32]: Liu et al. [27] follows the accelerated gradient descent methods in the Wasserstein probability space [34,35] and derives the Wasserstein Nesterov’s (WNES) and Wasserstein Accelerated Gradient (WAG) methods, which update the particles’ positions with an extra momentum. Inspired by the Accelerated Flow on the $R^{d}$ space [36], the Accelerated Flow (ACCEL) method [32] directly discretizes the Hamiltonian gradient flow in the Wasserstein space and updates the position with the damped velocity field, effectively decreasing the Hamiltonian potential of the particle system. Later, Wang and Li [33] considered the accelerated gradient flow for general information probability spaces [37], and derived novel accelerated position update strategies according to the Kalman–Wasserstein/Stein Hamiltonian flow. Following similar analyses as in [36], they theoretically show that, under mild conditions, the Hamiltonian flow usually converges to the equilibrium faster compared with the original first-order counterpart, aligning with the Nesterov acceleration framework [38]. Numerous experimental studies demonstrate that these accelerated position-update strategies usually drift the particle system to the target distribution more efficiently [27,32,33,39].
- *Dynamic weight adjustment.* Dynamic weight techniques, developed within Markov Chain Monte Carlo (MCMC) frameworks, have shown significant promise in improving sampling efficiency by adapting the weight of samples throughout the computation process. Building on these foundations, ref. [23] introduces the novel application of dynamic weights within the Wasserstein–Fisher–Rao (WFR) space to develop Dynamic-weight Particle-based Variational Inference (DPVI) methods. Specifically, they derive effective dynamical weight adjustment approaches by mimicking the reaction variational step in a JKO splitting scheme of first-order WFR gradient flow [40,41]. The seminal papers [42,43] provide foundational insights into the WFR geometry, particularly discussing an un-normalized dynamic weight variant based on a novel metric interpolating between the quadratic Wasserstein and the Fisher–Rao metrics, which is critical for developing these dynamic weight adjustment schemes. Compared with the commonly used fixed weight strategy, these dynamical weight adjustment schemes usually lead to less approximation error, especially when the number of particles is limited [23].

Given the contributions of both the acceleration technique and the dynamic-weight technique in enhancing the efficiency of ParVI, researchers have increasingly focused on developing a ParVI algorithm that can effectively integrate these features. One natural idea is to consider a ParVI algorithm that incorporates the second-order gradient flow in the WFR space, serving as a direct combination of an accelerated position update method (e.g., ACCEL, WNES, AIG) with a dynamic weight adjustment method (e.g., DPVI). However, we demonstrate that discretizing this flow generally does not yield a practical algorithm. Through our investigation, we discover that the primary obstacle lies in the *intractable kinetic energy* on the Fisher–Rao structure. For a detailed discussion on the IFR Hamiltonian flow, please refer to Appendix A.2.

### 1.1. Contribution

In this paper, we propose the first ParVI method, which possesses both accelerated position update and dynamical weight adjustment simultaneously. Specifically, we first construct a novel Information–Fisher–Rao (IFR) probability space, whereby the original information space is augmented with a Fisher–Rao structure. Infinitesimally, this Fisher–Rao structure is orthogonal to the Information structure. The orthogonality between metrics typically means orthogonality between tangent spaces, and details can be found in the work of [40]. Then, we present a novel Semi-Hamiltonian IFR (SHIFR) flow in this space, which simplifies the influence of the kinetic energy on the Fisher–Rao structure in the Hamiltonian IFR flow. By discretizing the SHIFR flow, a practical General Accelerated Dynamic-weight Particle-based Variational Inference (GAD-PVI) framework is proposed. The main contributions of our paper are as follows:

- We investigate the convergence property of the SHIFR flow and show that the target distribution π is the stationary distribution of the proposed semi-Hamiltonian flow for proper dissimilarity functional D(·|π). Moreover, our theoretical result also shows that the augmented Fisher–Rao structure yields an additional decrease in the local functional dissipation, compared to the Hamiltonian flow in the vanilla information space.
- We derive an effective finite-particle approximation to the SHIFR flow, which directly evolves the position, weight, and velocity of the particles via a set of ordinary differential equations. The finite particle system is compatible with different dissimilarity and associated smoothing approaches. We prove that the mean-field limit of the proposed particle system converges to the exact SHIFR flow under mild conditions.
- By adopting explicit Euler discretization for the finite-particle system, we create the General Accelerated Dynamic-weight Particle-based Variational Inference (GAD-PVI) framework, which updates positions in an acceleration manner and dynamically adjusts weights. We derive various GAD-PVI instances by using three different dissimilarities and associated smoothing approaches (KL-BLOB, KL-GFSD, and KSD-KSDD) on the Wasserstein/Kalman–Wasserstein/Stein IFR space, respectively.
- Furthermore, we showcase the versatility of our GAD-PVI by extending its applicability to scenarios where the analytic score is unavailable. We illustrate that the GAD-PVI algorithm can be utilized to develop methods for generating new samples from an unknown target distribution, given only a set of i.i.d. samples. This is achieved by employing suitable dissimilarities and their associated approximation approaches, such as Maximum-Mean-Distance–Maximum-Mean-Distance Flow (MMD-MMDF) and Sinkhorn-Divergence–Sinkhorn-Divergence Flow (SD-SDF), in the GAD-PVI framework.

We evaluate our algorithms under both the variational inference scenario tasks and the i.i.d. sample’s accessible sampling tasks. The empirical results demonstrate the superiority of our GAD-PVI methods.

### 1.2. Notation

Given a probability measure μ on $R^{d}$, we denote $\mu \in P_{2}\left(R^{d}\right)$ if its second moment is finite. For a given functional $F\left(\cdot \right):P_{2}\left(R^{d}\right)\to R$, $\frac{\delta F(\tilde{\mu })}{\delta \mu }\left(\cdot \right):R^{d}\to R$ denote its first variation at $\mu =\tilde{\mu }$. We use $C(R^{n})$ to denote the set of continuous functions map from $R^{n}$ to R. We denote $x^{i}\in R^{d}$ as the *i*-th particle, for i∈{1…M} and $K\left(\cdot ,\cdot \right):R^{d}\;\times \;R^{d}\to R$ as a positive semi-definite kernel function. We also denote the Dirac delta distribution with point mass located at $x^{i}$ as $\delta_{x^{i}}$, and use $f*g:R^{d}\to R$ to denote the convolution operation between $f:R^{d}\to R$ and $g:R^{d}\to R$. Furthermore, we use ∇ and ∇·() to denote the gradient and the divergence operator, respectively. We denote a general information probability space as $\left(P\left(R^{n}\right),G\left(\mu \right)\right)$, where G(μ)[·] denotes the one-to-one information metric tensor mapping elements in the tangent space $T_{\mu }P\left(R^{n}\right)\subset C\left(R^{n}\right)$ to the cotangent space $T_{\mu }^{*}P\left(R^{n}\right)\subset C\left(R^{n}\right)$. The inverse map of G(μ)[·] is denoted as $G^{-1}\left(\mu \right)\left[\cdot \right]:T_{\mu }^{*}P\left(R^{n}\right)\to T_{\mu }P\left(R^{n}\right)$.

## 2. Related Works

The core of Bayesian inference is to estimate the posterior distribution given the data. By reformulating the inference problem into an optimization problem, variational inference (VI) seeks an approximation within a certain family of distributions that minimizes the Kullback–Leibler (KL) divergence to the posterior. However, the construction of approximating distributions can be restrictive, which may lead to poor approximation [44].

Recently proposed Particle-based Variational Inference methods (ParVIs) use a set of samples, or particles, to represent the approximating distribution and deterministically update particles by minimizing the KL divergence to the target. ParVIs are more non-parametrically flexible than VIs. Stein Variational Gradient Descent (SVGD) [20] is the first and most representative of the ParVI-type algorithms. It updates the set of particles by incorporating a proper vector field that minimizes the kernelized Stein discrepancy with respect to the target. SVGD was later understood as simulating the Wasserstein gradient flow of the KL-divergence on a certain kernel-dependent probability space $P_{H}\left(R^{n}\right)$ [45]. The unique benefits of SVGD make it popular in various applications, including Generative Models [46,47], reinforcement learning [48,49], and recommendation systems [50].

Inspired by this gradient flow understanding of SVGD, more ParVIs have been developed by simulating the gradient flow on the Wasserstein space $P_{2}\left(R^{n}\right)$. Like the BLOB method [29], the Gradient Flow with Smoothed Density (GFSD) method [27] and the Kernel Stein Discrepancy Descend (KSDD) method [28] utilize different kernel tricksters to approximate the vector field form of the gradient flow and update the finite set of particles.

On accelerating ParVI, initially, Wasserstein Nesterov’s (WNES) method and Wasserstein Accelerated Gradient (WAG) method [27] leveraged the geometry of the underlying space to devise the Riemannian acceleration mechanism of ParVI with auxiliary points. More recently, The Accelerated Flow (ACCEL) [32] method has utilized the Hamiltonian dynamic on the original probability space to develop an accelerated ParVI method by incorporating a set of momentum variables. Further, the Accelerated Information Gradient Flow (AIG) method [33] extends such technique to general metric probability space beyond the Wasserstein metric. We follow the idea of leveraging the Hamiltonian flow on the probability space to develop second-order accelerated ParVIs, as this approach provides theoretical guarantees.

Regarding the dynamic-weight ParVIs, DPVI methods [23] include the first dynamic-weight ParVI method, which maintains a set of weighted particles and hence has a better approximation ability. DPVIs are proposed by leveraging the augmented Wasserstein–Fisher–Rao space rather than the vanilla Wasserstein space. The idea behind DPVI originates from MCMC–Birth–Death (MCMC-BD) [51], an MCMC-type sampling algorithm that is the first algorithm to introduce the Wasserstein–Fisher–Rao flow into the sampling literature. However, it is important to note that while MCMC-BD can transfer particles, it is unable to obtain weighted particles.

Recently, several studies have begun utilizing the mechanism of probability gradient flow to generate new samples from an unknown target distribution when only given a set of i.i.d. samples. One pioneering study introduced the Maximum Mean Discrepancy Flow (MMDF) method [30], which considers a particle flow to minimize the Maximum Mean Discrepancy (MMD) between the model particles and the accessible set of i.i.d. samples. The Sinkhorn Divergence Flow (SDF) method [52] instead considers the Sinkhorn Divergence (SD), which operates by finding the push-forward mapping in a Reproducing Kernel Hilbert Space that allows the fastest descent of SD and consequently solves the SD minimization problem iteratively. Note that this sampling setting is closely related to the emerging field of Generative Models (GMs), which includes well-known models such as Generative Adversarial Networks (GANs) [53], variational autoencoders (VAEs) [54], and Diffusion Models (DMs) [55,56]. However, it is important to clarify that the objective of this paper is not to achieve state-of-the-art performance in Generative Models but rather to demonstrate the extensibility of our technique within this particular setting. Indeed, there have been studies attempting to obtain state-of-the-art Generative Models based on Wasserstein Gradient Flow, such as the Neural Sinkhorn Gradient Flow (NSGF) method [31] and the S-JKO (JSD) method [57]. However, these methods typically require complex network design and additional components. Addressing this challenge remains an open question and is not the primary focus of this paper.

## 3. Preliminaries

When dealing with Bayesian inference tasks, variational inference methods approximate the target posterior π with an easy-to-sample distribution μ and recast the inference task as an optimization problem over $P_{2}\left(R^{d}\right)$ [58]:

(1) $$\begin{array}{c} \min_{\mu \in P_{2}\left(R^{n}\right)}F\left(\mu \right):=D\left(\mu |\pi \right). \end{array}$$

To solve this optimization problem, Particle-based Variational Inference (ParVI) methods generally simulate the gradient flow of F(μ) in a certain probability space with a finite particle system, which transports the initial empirical distribution toward the target distribution π iteratively. Given an information metric tensor G(μ)[·], the gradient flow in the information probability space $\left(P\left(R^{n}\right),G\left(\mu \right)\right)$ takes the following form [59]:

(2) $$\begin{array}{c} \partial_{t}\mu_{t}=-G{\left(\mu_{t}\right)}^{-1}\left[\frac{\delta F(\mu_{t})}{\delta \mu }\right]. \end{array}$$

### 3.1. Wasserstein Gradient Flow and Classical ParVIs

Since the seminal work on Stein Variational Gradient Descent (SVGD) [20], many ParVI methods have focused on flows in the Wasserstein space, where the inverse of the Wasserstein metric tensor is defined as

(3) $$\begin{array}{c} G^{W}{\left(\mu \right)}^{-1}\left[\varphi \right]=-\nabla \cdot \left(\mu \nabla \varphi \right),\forall \mu \in P\left(R^{n}\right),\forall \varphi \in T_{\mu }P\left(R^{n}\right), \end{array}$$

and the Wasserstein gradient flow is defined as

(4) $$\begin{array}{c} \partial_{t}\mu_{t}=\nabla \cdot \left(\mu_{t}\nabla \frac{\delta F(\mu_{t})}{\delta \mu }\right). \end{array}$$

Based on the probability flow (4) on the density, existing ParVIs maintain a set of particles $x_{t}^{i}$ and directly modify the particle position according to the following ordinary differential equation:

(5) $$\begin{array}{c} dx_{t}^{i}=\nabla \frac{\delta F({\tilde{\mu }}_{t})}{\delta \mu }\left(x_{t}^{i}\right)dt, \end{array}$$

where ${\tilde{\mu }}_{t}=\sum_{i=1}^{M}w_{t}^{i}\delta_{x_{t}^{i}}$ denotes the empirical distribution. Since the first total variation $\frac{\delta F({\tilde{\mu }}_{t})}{\delta \mu }$ of F might not be well defined for the discrete empirical distribution, various ParVI methods have been proposed by choosing different dissimilarities F and associated particle approaches, depending on the accessible information of the target distribution π. When the score of the target distribution ∇logπ(·) is accessible, some have ParVIs adopted the KL divergence or Kernel Stein Discrepancy (KSD) as the F, e.g., KL-BLOB [26], KL-GFSD [27], and KSD-KSDD [28]. When only samples of π are provided, integral-based dissimilarities like Maximum Mean Divergence (MMD) and Sinkhorn Divergence, which are naturally compatible with sample approximation, are adopted to develop ParVIs, like the MMDF method [30] and SD method [52].

### 3.2. Hamiltonian Gradient Flows and Accelerated ParVIs

The following Hamiltonian gradient flow in the general information probability space has recently been utilized to derive more efficient ParVI methods

(6) $$\begin{array}{c} \left\{\begin{array}{cc} \; & \partial_{t}\mu_{t}=\frac{\delta }{\delta \Phi }\;H\left(\mu_{t},\Phi_{t}\right),\\ \; & \partial_{t}\Phi_{t}\;=\;-\;\gamma_{t}\Phi_{t}\;-\;\frac{\delta }{\delta \mu }\;H\left(\mu_{t},\Phi_{t}\right), \end{array}\right. \end{array}$$

where $\Phi_{t}:R^{n}\to R$ represents the Hamiltonian momentum variable, while $H\left(\mu_{t},\Phi_{t}\right)=\frac{1}{2}\int \Phi_{t}G{\left(\mu_{t}\right)}^{-1}\left[\Phi_{t}\right]dx+F\left(\mu_{t}\right)$ signifies the Hamiltonian potential. It is pertinent to note that the Hamiltonian momentum variable $\Phi_{t}$ can be interpreted as the momentum of $\mu_{t}$. Note that the Hamiltonian flow (6) can be regarded as the second-order accelerated version of the information gradient flow (2) and usually converges faster to the equilibrium of the target distribution under mild conditions [32,33,39]. By reformulating the partial differential equations in terms of $\left(\mu_{t},\Phi_{t}\right)$ into Lagrangian formulations with respect to samples $X_{t}∼\mu_{t}$ and $V_{t}∼\nabla \Phi_{t}$ and further finite approximation, we obtain a simple augmented particle system $\left(x_{t}^{i},v_{t}^{i}\right)$, which evolves the position $x_{t}^{i}$ and velocity $v_{t}^{i}$ of particles simultaneously. As the position update rule of $x_{t}^{i}$ also uses the extra velocity information, the induced system is said to have an accelerated position update. By discretizing the continuous particle system, several accelerated ParVI methods have been proposed, which converge faster to the target distribution in numerous real-world Bayesian inference tasks [32,33].

### 3.3. Wasserstein–Fisher–Rao Flow and Dynamic-Weight ParVIs

Recently, the Wasserstein–Fisher–Rao (WFR) Flow has been used to derive effective dynamic weight adjustment approaches to mitigate the fixed-weight restriction of ParVIs [23]. The inverse of the WFR metric tensor is

(7) $$\begin{array}{c} G^{WFR}{\left(\mu \right)}^{-1}\left[\Phi \right]=-\nabla \cdot \left(\mu \nabla \Phi \right)+\left(\Phi -\int \Phi \right)\mu , \end{array}$$

where $\Phi \in T_{\mu }^{*}P\left(R^{n}\right)$, and the WFR gradient flow are written as

(8) $$\begin{array}{c} \partial_{t}\mu_{t}\;=\;\underset{\mathrm{Wasserstein}\;\mathrm{transport}}{\underset{︸}{\nabla \;\cdot \;(\mu_{t}\nabla \frac{\delta F(\mu_{t})}{\delta \mu })}}\;-\;\underset{\mathrm{Fisher}-\mathrm{Rao}\;\mathrm{variational}\;\mathrm{distortion}}{\underset{︸}{\left(\frac{\delta F(\mu_{t})}{\delta \mu }\;-\;\int \frac{\delta F(\mu_{t})}{\delta \mu }d\mu_{t}\right)\mu_{t}}}. \end{array}$$

Since the WFR space can be regarded as the orthogonal sum of the Wasserstein space and the Fisher–Rao space, ref. [23] mimics a JKO splitting scheme for the WFR flow, which deals with the position and the weight with the Wasserstein transport and the Fisher–Rao variational distortion, respectively. Given a set of particles with position, $x_{t}^{i}$ and weight $w_{t}^{i}$, the Fisher–Rao distortion can be approximated by the following code

(9) $$\begin{array}{cc} \frac{d}{dt}w_{t}^{i} & =-\left(\frac{\delta F({\tilde{\mu }}_{t})}{\delta \mu }\left(x_{t}^{i}\right)-\sum_{i=1}^{M}w_{t}^{i}\frac{\delta F({\tilde{\mu }}_{t})}{\delta \mu }\left(x_{t}^{i}\right)\right)w_{t}^{i}. \end{array}$$

According to (9), ref. [23] derive two dynamical weight-adjustment schemes and propose the Dynamic-Weight Particle-based Variational Inference (DPVI) framework, which is compatible with several dissimilarity functionals and associated particle approaches. The dynamic weight technique employed here differs from the one utilized in the dynamic-weight Sequential Monte Carlo (SMC) literature [19]. SMC-type algorithms achieve dynamic weight sampling through importance sampling, which is predicated on maintaining an estimated posterior. The weights of each incoming particle are individually calculated upon their arrival. The dynamic weight technique combined with sequential importance resampling is employed to circumvent degeneracy. In contrast, the Fisher–Rao dynamic weight technique iteratively adjusts the weights of the existing particles in an interactive way to augment the final approximation accuracy.

### 3.4. Dissimilarity Functionals

ParVIs typically select the probability gradient flow functional to be a divergence with respect to the target distribution, denoted as F(μ):=D(μ|π). In this context, we will now introduce four frequently employed dissimilarities, along with their corresponding first variation forms. The first two dissimilarities are typically employed in score-based tasks due to their logarithmic form. On the other hand, the last two dissimilarities are commonly utilized in sample-based tasks, where only a set of i.i.d. samples is available, as they have an integral form that can be estimated using the Monte Carlo method. In this subsection, *K* denotes a positive semi-definite kernel function.

#### 3.4.1. Kullback–Leibler Divergence

The Kullback–Leibler (KL) divergence is the most often used divergence in the ParVI field [20,27,33], when selecting KL divergence, the functional is written as

(10) $$\begin{array}{c} F^{KL}\left(\mu \right):=KL\left(\mu |\pi \right)=\int \log\mu \left(x\right)\frac{\mu (x)}{\pi (x)}dx \end{array}$$

The first variation of this function has the form

(11) $$\begin{array}{c} \frac{\delta F^{KL}\left(\mu \right)}{\delta \mu }\left(\cdot \right)=\frac{\delta KL(\mu |\pi )}{\delta \mu }\left(\cdot \right)=\log\mu \left(\cdot \right)-\log\pi \left(\cdot \right)+C \end{array}$$

#### 3.4.2. Kernel Stein Discrepancy

The Kernel Stein Discrepancy (KSD) has recently been adopted as the dissimilarity functional in the ParVI method KSDD [28], which is written as follows:

(12) $$\begin{array}{c} KSD\left(\mu |\pi \right):=\sqrt{\;\int \int \;k_{\pi }\left(x,y\right)d\mu \left(x\right)d\pi \left(y\right)} \end{array}$$

where $k_{\pi }$ is the Stein Kernel, defined through $k_{\pi }\left(x,y\right)=\nabla \log p{\left(x\right)}^{T}\nabla \log p\left(y\right)K\left(x,y\right)+\nabla \log p{\left(x\right)}^{T}\nabla_{y}K\left(x,y\right)+\nabla_{x}K{\left(x,y\right)}^{T}\nabla \log p\left(y\right)+\nabla_{x}\cdot \nabla_{y}K\left(x,y\right)$. We follow the KSDD method to consider $F^{KSD}\left(\mu \right):=\frac{1}{2}KSD^{2}\left(\mu |\pi \right)$; then, the first variation of this functional is written as

(13) $$\begin{array}{c} \frac{\delta F^{KSD}\left(\mu \right)}{\delta \mu }\left(\cdot \right)=\int \nabla_{x}k_{\pi }\left(x,\cdot \right)d\mu \left(x\right) \end{array}$$

#### 3.4.3. Maximum Mean Discrepancy

The Maximum Mean Discrepancy is a widely used integral probability metric, which has the following form:

(14) $$\begin{array}{cc} \; & {\mathrm{MMD}}^{2}\left(\mu |\pi \right):=\;\int \int \;K\left(x,x^{\prime }\right)d\mu \left(x\right)d\mu \left(x^{\prime }\right)\\ & +\;\int \int \;K\left(y,y^{\prime }\right)d\pi \left(y\right)d\pi \left(y^{\prime }\right)-2\;\int \int \;K\left(x,y\right)d\mu \left(x\right)d\pi \left(y\right). \end{array}$$

Consider $F^{\mathrm{MMD}}\left(\mu \right):=\frac{1}{2}{\mathrm{MMD}}^{2}\left(\mu |\pi \right)$; then, the first variation of this functional is written as

(15) $$\begin{array}{c} \frac{\delta F^{\mathrm{MMD}}\left(\mu \right)}{\delta \mu }\left(\cdot \right)=\int K\left(x,\cdot \right)d\mu \left(x\right)-\int K\left(y,\cdot \right)d\pi \left(y\right) \end{array}$$

#### 3.4.4. Sinkhorn Divergence

Sinkhorn divergence (SD) is derived as a computationally efficient counterpart to the famous Wasserstein distance by utilizing the regularization technique. The entropy-regularized Wasserstein distance is defined as

(16) $$\begin{array}{cc} \; & W_{p,\varepsilon }\left(\mu ,\pi \right)=\\ \; & \underset{\gamma \in \Gamma (\mu ,\pi )}{\inf}\left[{\left(\int_{R^{n}\;\times \;R^{n}}{∥x-y∥}^{2}d\gamma \left(x,y\right)\right)}^{\frac{1}{p}}+\varepsilon KL\left(\gamma |\mu ⊗\pi \right)\right], \end{array}$$

where ε>0 is a regularization coefficient, μ⊗π denotes the product measure, i.e.,  μ⊗π(x,y)=μ(x)π(y) and we fix p=2 and abbreviate $W_{2,\varepsilon }:=W_{\varepsilon }$. According to the Fenchel–Rockafellar theorem, the entropy-regularized Wasserstein problem $W_{\varepsilon }$ (16) has an equivalent dual formulation, which is given as follows [60]:

(17) $$\begin{array}{cc} W_{\varepsilon }\left(\mu ,\pi \right) & =\max_{f,g\in C(R^{n})}\langle \mu ,f\rangle +\langle \pi ,g\rangle \\ \; & -\varepsilon \langle \mu ⊗\pi ,\exp\left(\frac{1}{\varepsilon }\left(f⊕g-C\right)\right)-1\rangle , \end{array}$$

where *C* is the cost function in (16) and f⊕g is the tensor sum: (x,y)↦f(x)+g(y). The maximizers $f_{\mu ,\pi }$ and $g_{\mu ,\pi }$ of (17) are called the $W_{\varepsilon }$-potentials of $W_{\varepsilon }\left(\mu ,\pi \right)$. Note that, although computationally more efficient than the $W_{p}$ distance, the $W_{\varepsilon }$ distance is not a true metric, as there exists $\mu \in P_{2}\left(R^{n}\right)$ such that $W_{\varepsilon }\left(\mu ,\mu \right)\neq 0$ when ε≠0, which restricts the applicability of $W_{\varepsilon }$. As a result, the following Sinkhorn divergence $S_{\varepsilon }\left(\mu ,\pi \right):P_{2}\left(R^{n}\right)\;\times \;P_{2}\left(R^{n}\right)\to R$ is proposed [60]:

(18) $$\begin{array}{c} S_{\varepsilon }\left(\mu ,\pi \right)=W_{\varepsilon }\left(\mu ,\pi \right)-\frac{1}{2}\left(W_{\varepsilon }\left(\mu ,\mu \right)+W_{\varepsilon }\left(\pi ,\pi \right)\right). \end{array}$$

Consider $F^{SD}\left(\cdot \right)=S_{\varepsilon }\left(\cdot ,\pi \right)$. Let $\left(f_{\mu ,\pi },g_{\mu ,\pi }\right)$ be the $W_{\varepsilon }$-potentials of $W_{\varepsilon }\left(\mu ,\pi \right)$ and let $\left(f_{\mu ,\mu },g_{\mu ,\mu }\right)$ be the $W_{\varepsilon }$-potentials of $W_{\varepsilon }\left(\mu ,\mu \right)$. The first variation of the Sinkhorn functional $F^{SD}$ is

(19) $$\frac{\delta F^{SD}}{\delta \mu }=f_{\mu ,\pi }-f_{\mu ,\mu }.$$

## 4. Methodology

In this section, we present our General Accelerated Dynamic-weight Particle-based Variational Inference (GAD-PVI) framework, detailed in Algorithm 1. We first introduce a novel augmented Information–Fisher–Rao space, and the Semi-Hamiltonian-Information–Fisher–Rao (SHIFR) flow in the space. The theoretical analysis of SHIFR shows that it usually possesses an additional decrease in the local functional dissipation compared to the Hamiltonian flow in the original information space. Then, effective finite-particle systems, which directly evolve the position, weight, and velocity of the particles via a set of ordinary differential equations, are constructed based on SHIFR flows in several IFR spaces with different underlying information metric tensors. We demonstrate that the mean-field limit of the constructed particle system exactly converges to the SHIFR flow in the corresponding probability space. Next, we develop the GAD-PVI framework by discretizing these continuous-time finite-particle formulations, which enables simultaneous accelerated updates of particles’ positions and dynamic adjustment of particles’ weights. We present nine effective GAD-PVI algorithms that use different underlying information metric tensors, dissimilarity functionals, and the associated finite-particle empirical approximation.
**Algorithm 1** General Accelerated Dynamic-weight Particle-based Variational Inference (GAD-PVI) framework**Input**: Initial distribution ${\tilde{\mu }}_{0}=\sum_{i=1}^{M}w_{0}^{i}\delta_{x_{0}^{i}}$, position adjusting step-size $\eta_{pos}$, weight adjusting step-size $\eta_{wei}$, velocity field adjusting step-size $\eta_{vel}$, velocity damping parameter γ.1:Choose a suitable functional F and its empirical approximation $U_{\tilde{\mu }}$ according to the sampling setting.2:**for** k=0,1,…,T−1 **do**3:   **for** i=1,2,…,M **do**4:     Update positions $x_{k+1}^{i}$’s according to (26).5:   **end for**6:   **for** i=1,2,…,M **do**7:     Adjust velocity field $v_{k+1}^{i}$’s according to (27).8:   **end for**9:   **if** Adopt CA strategy **then**10:     **for** i=1,2,…,M **do**11:        Adjust weights $w_{k+1}^{i}$’s according to (28).12:     **end for**13:**else if** Adopt DK strategy **then**14:     **for** i=1,2,…,M **do**15:        Calculate the duplicate/kill rate: $R_{k+1}^{i}=-\lambda \eta \left(U_{{\tilde{\mu }}_{k}}\left(x_{k+1}^{i}\right)-\frac{1}{M}\sum_{i=1}^{M}U_{{\tilde{\mu }}_{k}}\left(x_{k+1}^{i}\right)\right)$16:     **end for**17:     **for** i=1,2,…,M **do**18:        **if** $R_{k+1}^{i}>0$ **then**19:          Duplicate the particle $x_{k+1}^{i}$ with probability $1-\exp(-R_{k+1}^{i})$ and kill one which is uniformly chosen from the rest.20:        **else**21:          Kill the particle $x_{k+1}^{i}$ with probability $1-\exp(R_{k+1}^{i})$ and duplicate one which is uniformly chosen from the rest.22:        **end if**23:     **end for**24:   **end if**25:**end for**26:**Output**: ${\tilde{\mu }}_{T}=\sum_{i=1}^{M}w_{T}^{i}\delta_{x_{T}^{i}}$.

### 4.1. Information–Fisher–Rao Space and Semi-Hamiltonian-Information–Fisher–Rao Flow

To define the augmented Information–Fisher–Rao probability space, we introduce the Information–Fisher–Rao metric tensor $G^{IFR}\left(\mu \right)$, whose inverse is defined as follows.

(20) $$\begin{array}{c} G^{IFR}{\left(\mu \right)}^{-1}\left[\Phi \right]=G^{I}{\left(\mu \right)}^{-1}\left[\Phi \right]+\left(\Phi -\int \Phi d\mu \right)\mu , \end{array}$$

where $\Phi \in T_{\mu }^{*}P\left(R^{n}\right)$ and $G^{I}\left(\mu \right)$ denotes certain underlying information metric tensor. Note that $G^{IFR}\left(\mu \right)$ is formed by the inf-convolution of $G^{I}\left(\mu \right)$ and Fisher–Rao metric tensor.

Based on $G^{IFR}\left(\mu \right)$, we introduce the following novel semi-Hamiltonian flow of F on the Information–Fisher–Rao space $\left(P\left(R^{n}\right),G^{IFR}\left(\mu \right)\right)$

(21) $$\begin{array}{c} \left\{\begin{array}{cc} \; & \partial_{t}\mu_{t}=\frac{\delta }{\delta \Phi }\;H^{IFR}\left(\mu_{t},\Phi_{t}\right),\\ \; & \partial_{t}\Phi_{t}\;=\;-\;\gamma_{t}\Phi_{t}\;-\;\frac{1}{2}\frac{\delta }{\delta \mu }\;\left(\;\int \Phi_{t}G^{I}{\left(\mu_{t}\right)}^{-1}\left[\Phi_{t}\right]dx\;\right)\;\;-\;\frac{\delta F(\mu_{t})}{\delta \mu }. \end{array}\right. \end{array}$$

where $\Phi_{t}$ denotes the Hamiltonian velocity and

(22) $$\begin{array}{cc} H^{IFR}\left(\mu_{t},\Phi_{t}\right) & =\underset{\mathrm{Information}\;\mathrm{kinetic}\;\mathrm{energy}}{\underset{︸}{\frac{1}{2}\;\;\int \Phi_{t}G^{I}{\left(\mu_{t}\right)}^{-1}\left[\Phi_{t}\right]dx}}\;\\ \; & +\underset{\mathrm{Fisher}-\mathrm{Rao}\;\mathrm{kinetic}\;\mathrm{energy}}{\underset{︸}{\frac{1}{2}\int \Phi_{t}\left(\Phi_{t}-\int \Phi d\mu_{t}\right)d\mu_{t}}}+\underset{\mathrm{potential}\;\mathrm{energy}}{\underset{︸}{\frac{\delta F(\mu_{t})}{\delta \mu }}}, \end{array}$$

denotes the Hamiltonian potential in the IFR space. Compared to the full Hamiltonian flow of F in the IFR space, the SHIFR flow (21) ignores the influence of the Fisher–Rao kinetic energy on the Hamiltonian field $\Phi_{t}$. Intuitively, at the gradient flow level, the Fisher–Rao metric modifies the mass in the vertical dimension, while the Wasserstein metric redistributes mass in the horizontal dimension. In the finite particle approximation system, we manipulate the weights and positions of particles to emulate the underlying infinite-dimensional mass of the distribution μ. The Fisher–Rao metric exclusively adjusts the weights of particles, serving as an analogy for altering the mass in the vertical dimension in the infinite case. Conversely, the Wasserstein metric changes the position of particles, acting as an analogy for adjusting the mass in the horizontal dimension. These two techniques interact with each other, collectively facilitating faster convergence.

Later, we will show that SHIFR can be directly transformed into a particle system consisting of odes on the positions, velocities, and weights of particles for proper underlying information metric tensor, while it is generally infeasible to obtain such a direct particle system according to the corresponding full Hamiltonian flow because it is difficult to handle the Fisher–Rao kinetic energy. Given that the Fisher–Rao kinetic energy term diminishes when approaching the flow’s equilibrium, it is acceptable for the SHIFR flow to disregard this complex term while still maintaining the target distribution π as its stationary distribution. The following proposition establishes that the stationary property of the SHIFR Flow (21) is still the target distribution.

**Proposition** **1.** *The target distribution and zero-velocity $\left(\mu_{\infty }=\pi ,\Phi_{\infty }=0\right)$ (0 means that a function defined on $R^{n}$ that always maps to zero) is the stationary distribution of the SHIFR flow (21) with dissimilarity functional D(·|π), which satisfies D(π|π)=0 with any information metric tensor $G^{I}\left(\mu \right)\left[\cdot \right]$.*

Moreover, this semi-Hamiltonian flow would converge faster than the Hamiltonian flow in the original information space on account of the extra functional dissipation property. Here, we establish the extra decrease property in terms of functional dissipation of the SHIFR gradient flow (21) in the following proposition.

**Proposition** **2.** *For arbitrary $\bar{\mu }\in P\left(R^{n}\right)$ and $\bar{\Phi }\in C\left(R^{n}\right)$, the local dissipation of functional $\frac{dF(\mu_{t})}{dt}$ following the SHIFR gradient flow (21) starting from $\left(\bar{\mu },\bar{\Phi }\right)$ has an additional functional dissipation term compared to the ones following the Hamiltonian flow in non-augmented space (6). Take the Wasserstein case as an example. Denote the probability path starting from $\left(\bar{\mu },\bar{\Phi }\right)$ following the W-SHIFR flow as $\left(\mu_{t}^{SHIFR},\Phi_{t}^{SHIFR}\right)$, and following the Hamiltonian flow in vanilla space as $\left(\mu_{t}^{H},\Phi_{t}^{H}\right)$. We have*

(23) $$\begin{array}{c} {\left.\frac{dF(\mu_{t}^{SHIFR})}{dt}\right|}_{t=0}{\left.\leq \frac{dF(\mu_{t}^{H})}{dt}\right|}_{t=0} \end{array}$$

We acknowledge that these theoretical analyses are currently limited to the variational inference case, where the functional is set to a dissimilarity with respect to a target distribution π, denoted as F(μ):=D(μ|π). Future work will aim to explore the semi-Hamiltonian system in a broader statistical physics context.

With different underlying information metric tensor $G^{I}\left(\mu \right)$ in $H^{IFR}\left(\mu_{t},\Phi_{t}\right)$, we can obtain different SHIFR flows. Suitable $G^{I}\left(\mu \right)$ includes the Wasserstein metric tensor, the Kalman–Wasserstein metric tensor (KW-metric) and the Stein metric tensor (S-metric). For instance, the  SHIFR flow with Wasserstein metric (Wasserstein–SHIFR flow) is written as

(24) $$\begin{array}{c} \left\{\begin{array}{cc} \; & \partial_{t}\mu_{t}\;=-\nabla \cdot \left(\mu_{t}\nabla \Phi_{t}\right)\;-\;\left(\frac{\delta F(\mu_{t})}{\delta \mu }\;-\;\int \frac{\delta F(\mu_{t})}{\delta \mu }d\mu_{t}\right)\mu_{t},\\ \; & \partial_{t}\Phi_{t}\;=\;-\;\gamma_{t}\Phi_{t}\;-\;{∥\nabla \Phi_{t}∥}^{2}\;-\;\frac{\delta F(\mu_{t})}{\delta \mu }. \end{array}\right. \end{array}$$

Note that in the subsequent section, we focus on the Wasserstein–SHIFR flow and defer the detailed formulations with respect to KW-SHIFR and S-SHIFR to Appendix B.1 and Appendix B.2 due to limited space.

### 4.2. Finite-Particles Formulations to SHIFR Flows

Now, we derive the finite-particle approximation to the SHIFR flow, which directly evolves the position $x_{t}^{i}$, weight $w_{t}^{i}$, and velocity $v_{t}^{i}$ of the particles. Specifically, we construct the following ordinary differential equation system to simulate the Wasserstein–SHIFR flow (24):

(25) $$\begin{array}{c} \left\{\begin{array}{cc} dx_{t}^{i} & =v_{t}^{i}dt,\\ dv_{t}^{i} & =(-\gamma v_{t}^{i}-\nabla \frac{\delta F({\tilde{\mu }}_{t})}{\delta \mu }\left(x_{t}^{i}\right))dt,\\ dw_{t}^{i} & =-\left(\frac{\delta F({\tilde{\mu }}_{t})}{\delta \mu }\left(x_{t}^{i}\right)-\sum_{i=1}^{M}w_{t}^{i}\frac{\delta F({\tilde{\mu }}_{t})}{\delta \mu }\left(x_{t}^{i}\right)\right)w_{t}^{i}dt,\\ {\tilde{\mu }}_{t} & =\sum_{i=1}^{M}w_{t}^{i}\delta_{x_{t}^{i}}. \end{array}\right. \end{array}$$

While the dynamic weight adjustment component of the proposed method (25) is quite similar to the ones in [23], as both are derived based on the Fisher–Rao structure of the underlying gradient flow, the proposed method can further achieve accelerated position updates. The following proposition demonstrates that the mean-field limit of the particle system (25) corresponds precisely to the Wasserstein–SHIFR flow in (24).

**Proposition** **3.** *Suppose the empirical distribution ${\tilde{\mu }}_{0}^{M}$ of M weighted particles weakly converges to a distribution $\mu_{0}$ when M→∞. Then, the path of (25) starting from ${\tilde{\mu }}_{0}^{M}$ and $\Phi_{0}$ with initial velocity 0 weakly converges to a solution of the Wasserstein–SHIFR gradient flow (24) starting from $\mu_{t}{|}_{t=0}=\mu_{0}=$ and $\Phi_{t}{|}_{t=0}=0$ as M→∞:*

Here, Proposition 3 serves as a bridge, substantiating that our proposed particle methods (25) possess appealing theoretical properties, as they are rooted in a mean-field limit of the Wasserstein–SHIFR flow (24) with superior theoretical attributes.

### 4.3. GAD-PVI Framework

Generally, it is impossible to obtain an analytic solution of the continuous finite-particle formulations (25); thus, a numerical integration method is required to derive an approximate solution. Note that any numerical solver, such as the implicit Euler method [61] and the higher-order Runge–Kutta method [62], can be used. Here, we follow the tradition of ParVIs to adopt the first-order explicit Euler discretization [63], since it is efficient and easy to implement [23], and propose our GAD-PVI framework, as listed in Algorithm 1. Like other ParVI methods, our GAD-PVI algorithm also sustains a set of particles and alters their attributes. However, the distinctive feature of GAD-PVI methods is their unique capacity to concurrently modify three different attributes, namely position, weight, and velocity. This pioneering approach sets GAD-PVI methods apart from other ParVI variants.

#### 4.3.1. Updating Rules

Suppose the functional F and its empirical approximation of the first variation $U_{\tilde{\mu }}\approx \frac{\delta F(\tilde{\mu })}{\delta \mu }$ is decided. We adopt a Jacobi-type strategy to update the position $x_{k}^{i}$, velocity field $v_{k}^{i}$, and weight $w_{k}^{i}$; i.e., the calculations in the k+1-th iteration are totally based on the variables obtained in the *k*-th iteration. Therefore, starting from *M* weighted particles located at ${\left\{x_{0}^{i}\right\}}_{i=1}^{M}$ with weights ${\left\{w_{0}^{i}\right\}}_{i=0}^{M}$ and ${\left\{v_{0}^{i}=0\right\}}_{i=0}^{M}$, GAD-PVI with respect to the Wasserstein–SHIFR flow first updates the positions of particles according to the following rule:

(26) $$\begin{array}{c} x_{k+1}^{i}=x_{k}^{i}+\eta_{pos}v_{k}^{i}. \end{array}$$

Then, it adjusts the velocity field as

(27) $$\begin{array}{c} v_{k+1}^{i}=\left(1-\gamma \eta_{vel}\right)v_{k}^{i}-\eta_{vel}\nabla U_{{\tilde{\mu }}_{k}}\left(x_{k}^{i}\right), \end{array}$$

and particles’ weights as follows:

(28) $$\begin{array}{c} w_{k+1}^{i}\;=w_{k}^{i}\;-\;\eta_{wei}\left(U_{{\tilde{\mu }}_{k}}\left(x_{k}^{i}\right)\;-\;\sum_{j=1}^{M}w_{k}^{j}U_{{\tilde{\mu }}_{k}}\left(x_{k}^{j}\right)\right). \end{array}$$

Here ${\tilde{\mu }}_{k}=\sum_{i=1}^{M}w_{k}^{i}\delta_{x_{k}^{i}}$ denotes the empirical distribution, and  $\eta_{pos}/\eta_{vel}/\eta_{wei}$ are the discretization step sizes. It can be verified that the total mass of ${\tilde{\mu }}_{k}$ is conserved and ${\tilde{\mu }}_{k}$ remains a valid probability distribution during the whole procedure of GAD-PVI, i.e., $\sum_{i}w_{k}^{i}=1$ for all *k*. The detailed updating rules of GAD-PVI with respect to the KW-SHIFR and S-SHIFR can be found in Appendix B.3.

Notice that, in comparison to traditional ParVIs, the incorporation of a position acceleration scheme and dynamic-weight scheme results in minimal additional computational costs. This is due to the fact that the number of operations that contribute to time complexity bottlenecks, specifically the calculation of $U_{\tilde{\mu }}$ and $\nabla U_{\tilde{\mu }}$, remains unchanged.

#### 4.3.2. Dissimilarities and Approximation Approaches

Our GAD-PVI framework is compatible with different dissimilarities (F) and their associated approximation approaches of the first variation. By selecting appropriate dissimilarities and approximation approaches, our GAD-PVI framework can be employed effectively for both score-based tasks and sample-based tasks.

##### Score-Based Scenario

When the score function value of the target distribution is accessible, the commonly used underlying dissimilarities in ParVIs are KL-divergence [20,27,33] and KSD [64].

For KL divergence, the total variation (11) includes the term logμ(x), which is ill-defined for the discrete empirical distribution ${\tilde{\mu }}_{k}$. Consequently, the use of approximation approaches is necessary to resolve this issue. Commonly employed approximation approaches include BLOB [29] and GFSD [27].

The **BLOB** approximation approach reformulates the intractable term logμ as $\frac{\delta }{\delta \mu }E_{\mu }\left[\log\mu \right]$ and smooth the density with a kernel function *K*, resulting in the approximation

(29) $$\begin{array}{cc} \log\tilde{\mu } & \approx \frac{\delta }{\delta \tilde{\mu }}E_{\tilde{\mu }}\left[\log\left(\tilde{\mu }*K\right)\right]\\ \; & :=\log\sum_{i=1}^{M}w^{i}K\left(\cdot ,x^{i}\right)+\sum_{i=1}^{M}\frac{w^{i}K\left(\cdot ,x^{i}\right)}{\sum_{j=1}^{M}w^{j}K\left(x^{i},x^{j}\right)}. \end{array}$$

for a discrete density $\tilde{\mu }=\sum_{i=1}^{M}w^{i}x^{i}$. This leads to the following approximation results:

(30) $$\begin{array}{c} \begin{array}{cc} U_{{\tilde{\mu }}_{k}}\left(x\right)= & -\log\pi (x)+\log\sum_{i=1}^{M}w_{k}^{i}K\left(x,x_{k}^{i}\right)\\ \; & +\sum_{i=1}^{M}\frac{w_{k}^{i}K\left(x,x_{k}^{i}\right)}{\sum_{j=1}^{M}w_{k}^{j}K\left(x_{k}^{i},x_{k}^{j}\right)}. \end{array} \end{array}$$

The **GFSD** approximation approach directly approximates μ by smoothing the empirical distribution $\tilde{\mu }$ with a kernel function *K*: $\hat{\mu }=\tilde{\mu }*K=\sum_{i=1}^{M}w^{i}K\left(\cdot ,x^{i}\right)$, which leads to the following approximations:

(31) $$\begin{array}{cc} U_{{\tilde{\mu }}_{k}}\left(x\right)= & -\log\pi (x)+\log\sum_{i=1}^{M}w_{k}^{i}K\left(x,x_{k}^{i}\right), \end{array}$$

(32) $$\begin{array}{cc} \nabla U_{{\tilde{\mu }}_{k}}\left(x\right)=\; & -\nabla \log\pi (x)+\frac{\sum_{i=1}^{M}w_{k}^{i}\nabla_{x}K\left(x,x_{k}^{i}\right)}{\sum_{i=1}^{M}w_{k}^{i}K\left(x,x_{k}^{i}\right)}. \end{array}$$

The **KSDD** directly approximates the first variation and its gradient of KSD (12) by employing Monte Carlo sampling with the empirical distribution $\tilde{\mu }$. KSDD constructs the following finite-particle approximations:

(33) $$\begin{array}{cc} U_{{\tilde{\mu }}_{k}}\left(x\right)= & \sum_{i=1}^{M}w_{k}^{i}k_{\pi }\left(x_{k}^{i},x\right), \end{array}$$

(34) $$\begin{array}{cc} \nabla U_{{\tilde{\mu }}_{k}}\left(x\right)= & \sum_{i=1}^{M}w_{k}^{i}\nabla_{x}k_{\pi }\left(x_{k}^{i},x\right). \end{array}$$

##### Sample-Based Scenario

When only the samples of the target distribution ${\left\{y_{j}\right\}}_{j=1}^{N}\overset{i.i.d.}{∼}\pi$ is accessible, we then take $\frac{1}{N}\sum_{j=1}^{N}\delta_{y_{j}}$ as a surrogate of π; the commonly used underlying dissimilarities in this case are MMD [30] and SD [52].

**MMDF** directly approximates the first variation and its gradient of MMD (14) by employing Monte Carlo sampling with both the empirical distribution $\tilde{\mu }$ and samples from the target distribution. Let ${\tilde{\mu }}_{k}=\sum_{i=1}^{M}w_{k}^{i}\delta_{x_{k}^{i}}$ and $\pi =\sum_{j=1}^{N}a^{j}\delta_{y^{j}}$; MMDF constructs the following finite-particle approximations:

(35) $$\begin{array}{cc} U_{{\tilde{\mu }}_{k}}\left(x\right)= & \sum_{i=1}^{M}w_{k}^{i}K\left(x_{k}^{i},x\right)-\sum_{j=1}^{N}a^{j}K\left(y^{j},x\right), \end{array}$$

(36) $$\begin{array}{cc} \nabla U_{{\tilde{\mu }}_{k}}\left(x\right)\;=\; & \sum_{i=1}^{M}w_{k}^{i}\nabla K\left(x_{k}^{i},x\right)\;-\;\sum_{j=1}^{N}a^{j}\nabla K\left(y^{j},x\right). \end{array}$$

**SDF** directly leverages samples from μ and π to obtain the approximated Sinkhorn potentials $f_{{\tilde{\mu }}_{k},\pi }$ and $f_{{\tilde{\mu }}_{k},{\tilde{\mu }}_{k}}$. Consequently, it is able to approximate the first variation of SD as follows.

(37) $$\begin{array}{cc} U_{{\tilde{\mu }}_{k}}\left(x\right)= & f_{{\tilde{\mu }}_{k},\pi }-f_{{\tilde{\mu }}_{k},{\tilde{\mu }}_{k}}, \end{array}$$

(38) $$\begin{array}{cc} \nabla U_{{\tilde{\mu }}_{k}}\left(x\right)= & \nabla f_{{\tilde{\mu }}_{k},\pi }-\nabla f_{{\tilde{\mu }}_{k},{\tilde{\mu }}_{k}}. \end{array}$$

The details of utilizing samples to obtain the approximated Sinkhorn potential can be found in [31,52].

#### 4.3.3. An Alternative Weight Adjusting Approach

Except for the Continuous Adjusting (CA) strategy, the Duplicate/Kill (DK) strategy, which is a probabilistic discretization strategy to the Fisher–Rao part of (24), can also be adopted in GAD-PVI. This strategy duplicates/kills particle $x_{k+1}^{i}$ according to an exponential clock with an instantaneous rate:

(39) $$\begin{array}{c} R_{k+1}^{i}\;=\;-\;\eta_{wei}\left(\frac{\delta F({\tilde{\mu }}_{k})}{\delta \mu }\left(x_{k}^{i}\right)\;-\;\sum_{j=1}^{M}w_{k}^{j}\frac{\delta F(\tilde{\mu_{k}})}{\delta \mu }\left(x_{k}^{j}\right)\right). \end{array}$$

Specifically, if $R_{k+1}^{i}>0$, we duplicate the particle $x_{k+1}^{i}$ with probability $1-\exp\left(-R_{k+1}^{i}\right)$ and kill another one with uniform probability to conserve the total mass; if $R_{k+1}^{i}<0$, we kill the particle $x_{k+1}^{i}$ with probability $1-\exp\left(R_{k+1}^{i}\right)$, and duplicate another one with uniform probability. By replacing the CA strategy (28) in the GAD-PVI framework, we could obtain the DK variants of GAD-PVI methods.

#### 4.3.4. GAD-PVI Instances

With different underlying information metric tensors (W-metric, KW-metric and S-metric), weight adjustment approaches (CA and DK), and dissimilarities/associated approximation approaches (KL-BLOB, KL-GFSD, KSD-KSDD, MMD-MMDF, SD-SDF), we can derive various instances of GAD-PVI, named as WGAD/KWGAD/SGAD-CA/DK-BLOB/GFSD/KSDD/MMDF/SDF. While the aforementioned updating rules, approximation approaches, and weight adjustment methods have been previously proposed, we conduct a comprehensive investigation of these components within a single framework and view them from a modular standpoint. Moreover, GAD-PVI designs a general inference framework for both the score-function scenario and the sample-based scenario, whereas previous works have only focused on one of these.

## 5. Experiments

In this section, we conduct empirical studies with our GAD-PVI algorithms. Our empirical studies include score-based tasks where the score function value of the target distribution is accessible, as well as sample-based tasks where only i.i.d. samples from the target distribution are available. Here, we focus on the instances of GAD-PVI with respect to the W-SHIFR flows. The experimental results for methods with respect to the KW-SHIFR and S-SHIFR flows are provided in Appendix A.3. Given that the Full Hamiltonian Flow on the IFR Space does not facilitate the development of practical algorithms, it is not feasible to incorporate the direct combination of an accelerated position update method (such as ACCEL, WNES, AIG) with a dynamic weight adjustment method (such as DPVI) into our experiments.

Our proposed algorithm simultaneously incorporates both accelerated position updates and dynamic weight adjustments. Therefore, comparing it to these baseline methods, which only possess one of these characteristics, can function as an ablation study for the components within our method.

Compared to existing dynamic-weight ParVIs, GAD-PVI benefits from the accelerated position update derived from the Hamiltonian acceleration mechanism of the SHIFR flow, enabling it to converge more quickly to the target distribution. When compared to ParVIs with only accelerated position updates, GAD-PVI benefits from the dynamic weight adjustment resulting from the Fisher–Rao component of the SHIFR flow, achieving superior approximation accuracy to the target distribution.

### 5.1. Score-Based Experiments

In the setting where we can access the analytical score function value of the target distribution, we choose KL-BLOB and KL-GFSD as the dissimilarity and empirical approximation of our GAD-PVI methods. Note that we do not include GAD-PVI methods with the KSDD empirical approaches, as they are more computationally expensive and have been widely reported to be less stable [23,64]. We include four classes of methods as our baseline: classical ParVI algorithms (SVGD, GFSD, and BLOB), the Nesterov accelerated ParVI algorithms (WNES-BLOB/GFSD), the Hamiltonian accelerated ParVI algorithms (WAIG-BLOB/GFSD), and the Dynamic-weight ParVI algorithms (DPVI-CA/DK-BLOB/GFSD).

In this score function setting, we consider four tasks, comprising two simulations: a 10-D Single-mode Gaussian model (SG) and a Gaussian mixture model (GMM), as well as two real-world applications: Gaussian Process (GP) regression and Bayesian neural network (BNN). For all the algorithms, the particles’ weights are initialized to be equal. In the first three experiments, we tune the parameters to achieve the best $W_{2}$ distance. In the BNN task, we split 1/5 of the training set as our validation set to tune the parameters. Note that the position step size is tuned via a grid search for the fixed-weight ParVI algorithms and then used in the corresponding dynamic-weight algorithms. The acceleration parameters and weight adjustment parameters are tuned via grid search for each specific algorithm. We repeat all the experiments 10 times and report the average results. Due to limited space, only parts of the results are reported in this section. We refer readers to Appendix C for the results on SG and additional results for GMM, GP, and BNN.

#### 5.1.1. Gaussian Mixture Model

**Figure 1:**
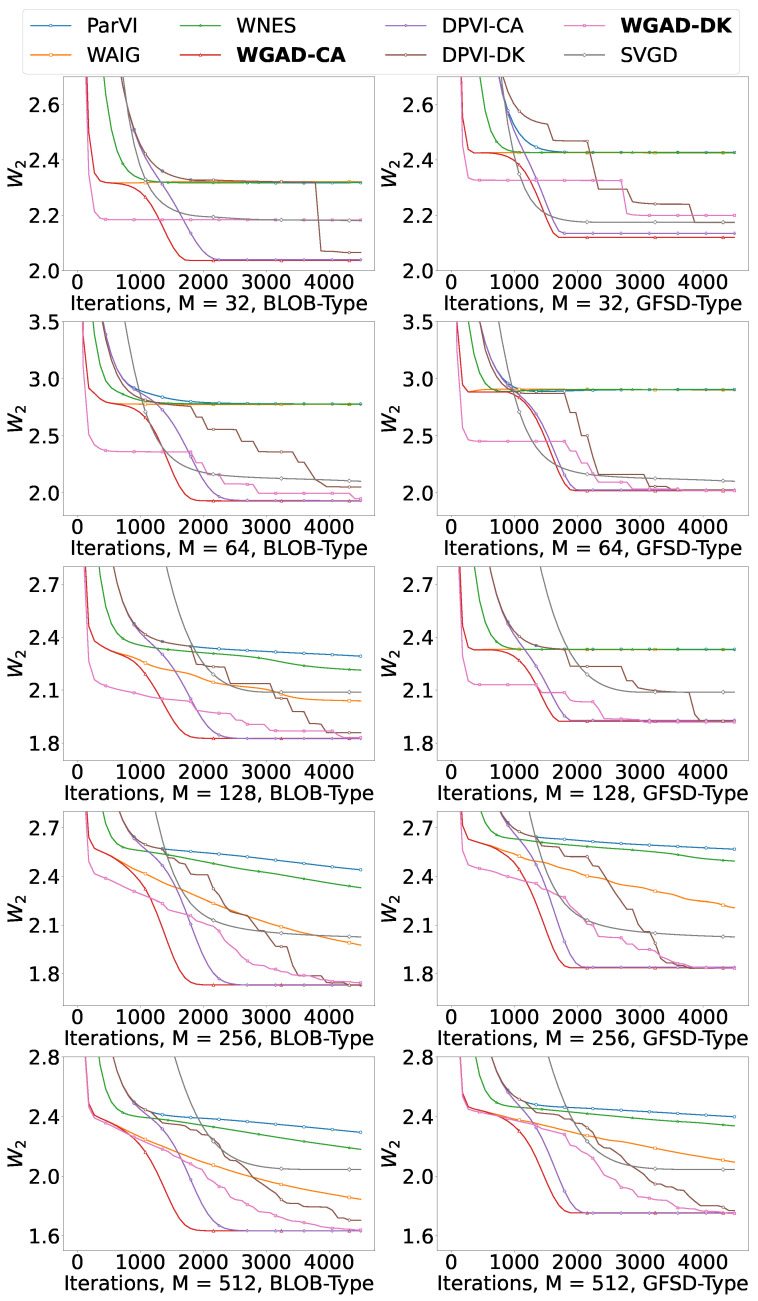
$W_{2}$ to the target with respect to iterations in the GMM task.

We consider approximating a 10-D Gaussian mixture model with two components, weighted by 1/3 and 2/3 respectively. We run all algorithms with particle number M∈{32,64,128,256,512}. In Figure 1, we report the 2-Wasserstein ($W_{2}$) distance between the empirical distribution generated by each algorithm and the target distribution with respect to iterations of different ParVI methods. We generate 5000 samples from the target distribution π as a reference to evaluate the $W_{2}$ distance by using the POT library http://jmlr.org/papers/v22/20-451.html (accessed on 16 August 2023).

The results demonstrate that our GAD-PVI algorithms consistently outperform their counterpart with only one (or none) of the accelerated position update strategy and dynamic weight adjustment approach. Furthermore, the CA weight-adjustment approach usually results in a lower $W_{2}$ compared to the DK scheme, and WGAD-CA-BLOB/GFSD usually has the fastest convergence and the lowest final $W_{2}$ distance to the target.

#### 5.1.2. Gaussian Process Regression

The Gaussian Process (GP) model is widely adopted for uncertainty quantification in regression problems [65]. We follow the experimental setting in [66], and use the dataset LIDAR (denoted as $D={\left\{\left(x_{i},y_{i}\right)\right\}}_{i=1}^{N}$), which consists of 221 observations. Of scalar variables $x_{i}$ and $y_{i}$. We denote $x={\left[x_{1},x_{2},\ldots ,x_{N}\right]}^{T}$ and $y={\left[y_{1},y_{2},\ldots ,y_{N}\right]}^{T}$, and the target log-posterior with respect to the model parameter $\phi =(\phi_{1},\phi_{2})$ is defined as follows:

$$\begin{array}{c} \log p(\phi |D)\;=\;-\frac{y^{T}K_{y}^{-1}y}{2}\;-\;\frac{\log\det\left(K_{y}\right)}{2}\;-\;\log\left(1\;+\;x^{T}x\right). \end{array}$$

Here, $K_{y}$ is a covariance function $K_{y}=K+0.04I$ with $K_{i,j}=\exp\left(\phi_{1}\right)\exp\left(-\exp\left(\phi_{2}\right){\left(x_{i}-x_{j}\right)}^{2}\right)$, and I represents the identity matrix. In this task, we set the particle number to M=128 for all the algorithms.

We report the $W_{2}$ distance between the empirical distribution after 10,000 iterations and the target distribution in Table 2. The target distribution is approximated by 10,000 reference particles generated by the HMC method after it achieves its equilibrium [67]. It can be observed that both the accelerated position update and the dynamic weight adjustment result in a decreased $W_{2}$, and GAD-PVI algorithms consistently achieve the lowest $W_{2}$ to the target. Furthermore, the results also show that the CA variants usually outperform their DK counterpart, as CA is able to adjust the weight continuously on [0,1] while DK sets the weight to either 0 or 1/M.

**Figure 2:**
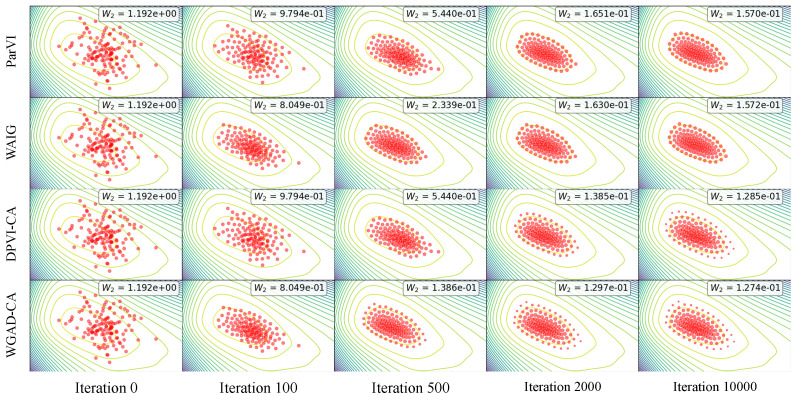
The contour lines of the log posterior in the Gaussian Process task (all variants with BLOB strategy).

In Figure 2, we plot the contour lines of the log posterior and the particles generated by four representative algorithms, namely BLOB, WAIG-BLOB, DPVI-CA-BLOB, and WGAD-CA-BLOB, at different iterations (0, 100, 500, 2000, 10,000). The results indicate that the particles in WAIG-BLOB and WGAD-CA-BLOB exhibit a faster convergence to the high probability area of the target due to their accelerated position updating strategy, and the DPVI-CA and WGAD-CA algorithms finally offer broader final coverage, as the CA dynamic weight adjustment strategy enables the particles to represent the region with arbitrary local density mass instead of a fixed 1/M mass.

#### 5.1.3. Bayesian Neural Network

In this experiment, we study a Bayesian regression task with a Bayesian neural network on four datasets from UCI, http://archive.ics.uci.edu/ml/datasets (accessed on 16 August 2023), and and LIBSVM, https://www.csie.ntu.edu.tw/~cjlin/libsvmtools/datasets/ (accessed on 16 August 2023). Given a training dataset $D={\left\{\left(x_{i},y_{i}\right)\right\}}_{i=1}^{N}$, where $x_{i}$ denotes the input covariate vector and $y_{i}$ is the corresponding prediction, this task aims at predicting the output $y^{*}$ for a new input $x^{*}$ from the perspective of Bayesian inference:

$$\begin{array}{c} p\left(y^{*}|x^{*},D\right)=\int p\left(y^{*}|x^{*},w\right)p\left(w|D\right)dw, \end{array}$$

where w denotes the model parameter of the neural network and p(w|D) is the target posterior given training dataset D. Since the explicit integration is intractable, one can resort to sampling methods to approximate the target posterior p(w|D) and transfer the integration problem into calculating the average with a set of samples. We follow the experimental setting from [20,23], which models the output as a Gaussian distribution and uses a Gamma(1,0.1) prior for the inverse covariance. We use a one-hidden-layer neural network with 50 hidden units and maintain 128 particles. For all the datasets, we set the batch size as 128.

We present the Root Mean Squared Error (RMSE) of various ParVI algorithms in Table 3. The results demonstrate that the combination of the accelerated position updating strategy and the dynamically weighted adjustment leads to a lower RMSE. Notably, WGAD-CA type algorithms outperform other methods in the majority of cases.

### 5.2. Sample-Based Experiments

In the setting where we can only obtain the i.i.d. samples of the target distribution, we choose MMD-MMDF and SD-SDF as the dissimilarity and empirical approximation of our GAD-PVI methods.

We are the first to consider the intricate structure of the underlying gradient flow within the ParVI algorithm under the sample-based setting. Consequently, we include classical ParVI algorithms (MMDF and SDF) as our baseline methods, along with DPVI-type and WAIG-type algorithms as ablation baselines. In this sample-based setting, we consider two tasks: the shape morphing task between different 2-D icons and the Sketching task of high-resolution pictures. For all the algorithms, the particles’ weights are initialized to be equal.

Note that the position steps are tuned via grid search for the baseline ParVI algorithms and then used in the corresponding GAD-PVI algorithms. The acceleration parameters and weight adjustment parameters are tuned via grid search for each specific algorithm. We repeat all the experiments 10 times and report the average results.

#### 5.2.1. Shape Morphing

In this experiment, we study the task of shape morphing between different icons. The source shape and target shape are distributions lying in $R^{2}$. We need to move points sampled uniformly from shape A to shape B. This task is often considered in the Wasserstein Barycenter literature [68,69,70]. Note that, to make the task more complex, we add an unbalanced distortion on the X-axis to the target distribution so that the probability distribution density on the left side of the target distribution is greater than that on the right. We consider a single loop shape morphing between the four icons, i.e., A(CAT), B(SPIRAL), C(HEART), and D(CHICKEN).

**Figure 3:**
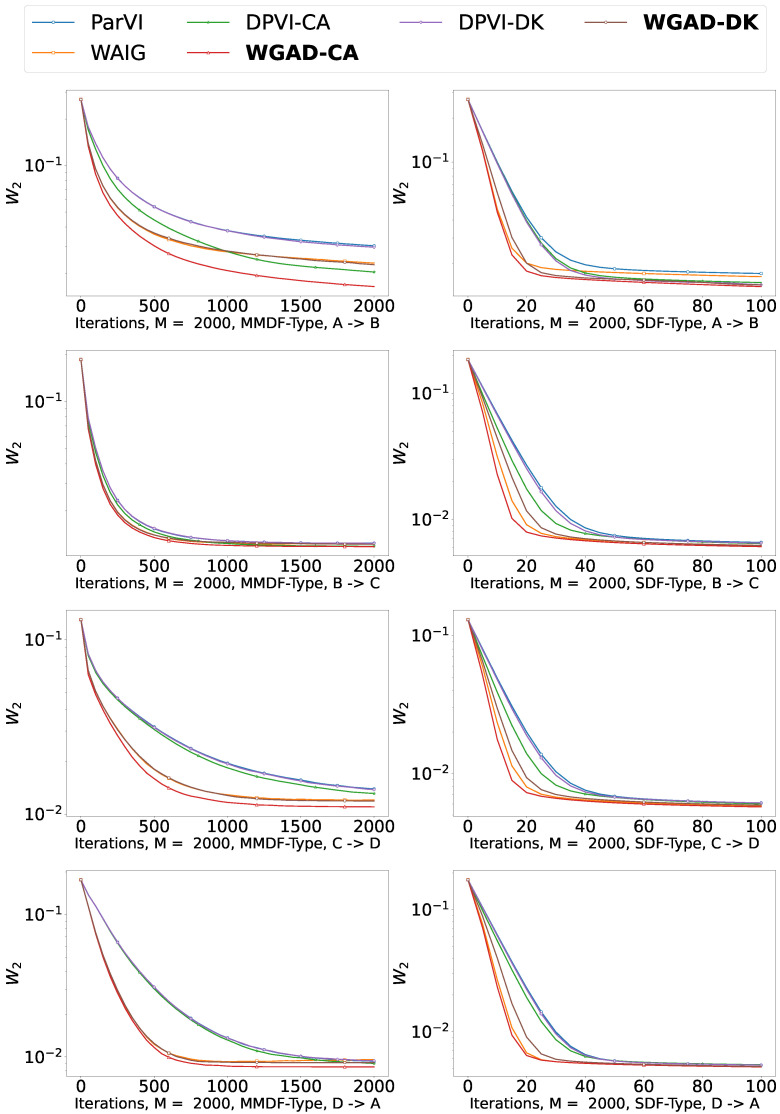
$W_{2}$ distance to the target with respect to iterations in the shape morphing task.

In Figure 3, we report the 2-Wasserstein ($W_{2}$) distance between the empirical distribution generated by each algorithm and the target icon with respect to iterations of different ParVI methods. We generate 2000 samples from the target distribution π as a reference to evaluate the $W_{2}$ distance. The results demonstrate that our GAD-PVI algorithms consistently outperform their baselines.

Table 4 presents the average $W_{2}$ distance to the target distribution after 2000 iterations (MMDF-Type) or 100 iterations (SDF-Type). It can be observed that GAD-PVI methods consistently achieve the lowest $W_{2}$ distance to the target, attributed to their dynamic weight adjustment.

#### 5.2.2. Picture Sketching

This section presents the results of the picture sketching experiment, a task that can be viewed as approximating a given picture with particles and is therefore called picture sketching. Specifically, this section uses real cheetah images as original data, image pixels as particle points, and gray values of pixels as weights to generate discrete target distribution in $R^{2}$ space. In this experiment, all algorithms are initialized to 1000 equal-weight particle points, and all particle points are initially sampled from uniform noise distribution.

**Figure 4:**
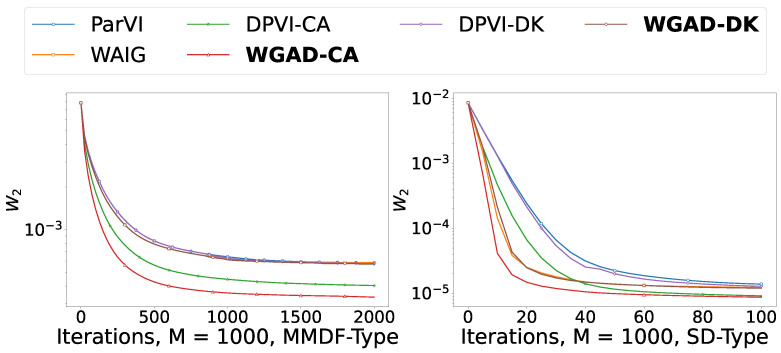
$W_{2}$ distance to the target with respect to iterations in the sketching task.

In Figure 4, we report the 2-Wasserstein ($W_{2}$) between the empirical distribution generated by each algorithm and the target picture with respect to iterations of different ParVI methods. The results demonstrate that our GAD-PVI algorithms consistently outperform their baselines and the CA weight-adjustment approach usually results in the lowest $W_{2}$ distance to the target and fastest convergence.

**Figure 5:**
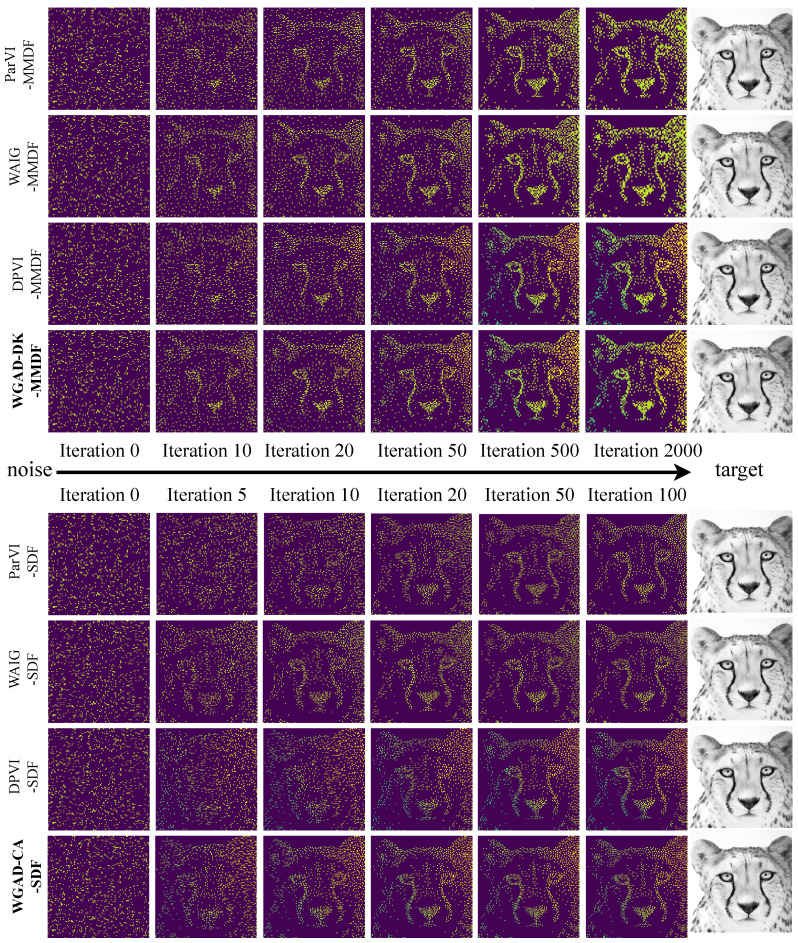
The sketching task from random noise to cheetah, M=1000.

Figure 5 shows the sketching process from the initial noise to the target picture of different ParVIs. In this visualization, particle points with higher weights are represented as pixels with brighter intensities. The results demonstrate that ParVI with accelerated position updates converges more rapidly towards the target picture. Furthermore, ParVI with dynamic-weight adjustment exhibits superior ability in accurately depicting the target picture. Specifically, the ParVI algorithm with dynamic-weight adjustment effectively captures the chiaroscuro between the cheetah’s two ears, which is not evident in the fixed-weight baseline approach.

## 6. Conclusions

In this paper, we propose the General Accelerated Dynamic-Weight Particle-based Variational Inference (GAD-PVI) framework, which adopts an accelerated position update scheme and dynamic weight adjustment approach simultaneously. Our GAD-PVI framework is developed by discretizing the Semi-Hamiltonian Information Fisher–Rao (SHIFR) flow on the novel Information–Fisher–Rao space. The theoretical analysis demonstrates that the SHIFR flow yields an additional decrease in the local functional dissipation compared to the Hamiltonian flow in the vanilla information space. We propose an effective particle system that evolves the position, weight, and velocity of particles via a set of odes for the SHIFR flows with different underlying information metrics. By directly discretizing the proposed particle system, we obtain our GAD-PVI framework. Several effective instances of the GAD-PVI framework have been provided by employing three distinct dissimilarity functionals and associated empirical approaches under the Wasserstein/Kalman–Wasserstein/Stein metric. Empirical studies demonstrate the faster convergence and reduced approximation error of GAD-PVI methods over the SOTAs.

## Figures and Tables

**Table 1:** Feature-by-feature comparison of different ParVIs.

	Features	Accelerated Position Update	Dynamic Weight Adjustment	Dissimilarity and Empirical Approximation	Underlying Probability Space	Target Distribution Accessibility
Methods						
SVGD [20]		✗	✗	KL-RKHS	Wasserstein	Score
BLOB [29]		✗	✗	KL-BLOB	Wasserstein	Score
KSDD [28]		✗	✗	KSD-KSDD	Wasserstein	Score
MMDF [30]		✗	✗	MMD-MMDF	Wasserstein	Samples
SDF [31]		✗	✗	SD-SDF	Wasserstein	Samples
ACCEL [32]		✓	✗	KL-GFSD	Wasserstein	Score
WNES, WAG [27]		✓	✗	General	Wasserstein	Score
AIG [33]		✓	✗	KL-GFSD	Information (General)	Score
DPVI [23]		✗	✓	General	WFR	Score
GAD-PVI (Ours)		✓	✓	General	IFR (General)	Both

**Table 2:** Averaged $W_{2}$ for the GP task with dataset LIDAR.

Algorithm	Empirical Strategy	
	BLOB	GFSD
ParVI	$1.570\times 10^{-1}$ ± $2.210\times 10^{-4}$	$2.143\times 10^{-1}$ ± $7.424\times 10^{-4}$
WAIG	$1.572\times 10^{-1}$ ± $2.070\times 10^{-4}$	$2.142\times 10^{-1}$ ± $7.048\times 10^{-4}$
WNES	$1.571\times 10^{-1}$ ± $3.011\times 10^{-4}$	$2.138\times 10^{-1}$ ± $7.771\times 10^{-4}$
DPVI-DK	$1.568\times 10^{-1}$ ± $1.496\times 10^{-3}$	$2.142\times 10^{-1}$ ± $2.712\times 10^{-3}$
DPVI-CA	$1.285\times 10^{-1}$ ± $2.960\times 10^{-4}$	$1.638\times 10^{-1}$ ± $4.332\times 10^{-4}$
WGAD-DK	$1.561\times 10^{-1}$ ± $1.155\times 10^{-3}$	$2.142\times 10^{-1}$ ± $1.501\times 10^{-3}$
WGAD-CA	$1.274\times 10^{-1}$ ± $2.964\times 10^{-4}$	$1.626\times 10^{-1}$ ± $4.842\times 10^{-4}$

**Table 3:** Averaged test RMSE in the BNN task.

Algorithms	Datasets			
	Concrete	kin8nm	RedWine	Space
ParVI-SVGD	6.323	8.020 × $10^{-2}$	6.330 × $10^{-1}$	9.021 × $10^{-2}$
ParVI-BLOB	6.313	7.891 × $10^{-2}$	6.318 × $10^{-1}$	8.943 × $10^{-2}$
WAIG-BLOB	6.063	7.791 × $10^{-2}$	6.267 × $10^{-1}$	8.775 × $10^{-2}$
WNES-BLOB	6.112	7.690 × $10^{-2}$	6.264 × $10^{-1}$	8.836 × $10^{-2}$
DPVI-DK-BLOB	6.285	7.889 × $10^{-2}$	6.294 × $10^{-1}$	8.853 × $10^{-2}$
DPVI-CA-BLOB	6.292	7.789 × $10^{-2}$	6.298 × $10^{-1}$	8.850 × $10^{-2}$
WGAD-DK-BLOB	6.058	7.688 × $10^{-2}$	6.267 × $10^{-1}$	8.716 × $10^{-2}$
WGAD-CA-BLOB	6.047	7.629 × $10^{-2}$	6.263 × $10^{-1}$	8.704 × $10^{-2}$
ParVI-GFSD	6.314	7.891 × $10^{-2}$	6.317 × $10^{-1}$	8.943 × $10^{-2}$
WAIG-GFSD	6.105	7.794 × $10^{-2}$	6.265 × $10^{-1}$	8.776 × $10^{-2}$
WNES-GFSD	6.123	7.756 × $10^{-2}$	6.263 × $10^{-1}$	8.836 × $10^{-2}$
DPVI-DK-GFSD	6.291	7.882 × $10^{-2}$	6.277 × $10^{-1}$	8.851 × $10^{-2}$
DPVI-CA-GFSD	6.290	7.791 × $10^{-2}$	6.298 × $10^{-1}$	8.852 × $10^{-2}$
WGAD-DK-GFSD	6.099	7.726 × $10^{-2}$	6.265 × $10^{-1}$	8.708 × $10^{-2}$
WGAD-CA-GFSD	6.088	7.634 × $10^{-2}$	6.260 × $10^{-1}$	8.710 × $10^{-2}$

**Table 4:** Averaged $W_{2}$ for the shape morphing task between different shapes (A = CAT, B = SPIRAL, C = HEART, D = CHICKEN) and averaged $W_{2}$ for the sketching task of high-resolution cheetah picture.

Algorithms	Sampling Tasks				
	A→B	B→C	C→D	D→A	Sketching
ParVI-MMDF	$3.019\times 10^{-2}$	$1.245\times 10^{-2}$	$1.396\times 10^{-2}$	$9.338\times 10^{-3}$	$5.846\times 10^{-4}$
WAIG-MMDF	$2.332\times 10^{-2}$	$1.232\times 10^{-2}$	$1.205\times 10^{-2}$	$9.589\times 10^{-3}$	$5.869\times 10^{-4}$
DPVI-DK-MMDF	$2.956\times 10^{-2}$	$1.243\times 10^{-2}$	$1.385\times 10^{-2}$	$9.411\times 10^{-3}$	$5.754\times 10^{-4}$
DPVI-CA-MMDF	$2.039\times 10^{-2}$	$1.190\times 10^{-2}$	$1.316\times 10^{-2}$	$9.008\times 10^{-3}$	$4.040\times 10^{-4}$
WGAD-DK-MMDF	$2.275\times 10^{-2}$	$1.225\times 10^{-2}$	$1.190\times 10^{-2}$	$9.126\times 10^{-3}$	$5.721\times 10^{-4}$
WGAD-CA-MMDF	$1.642\times 10^{-2}$	$1.183\times 10^{-2}$	$1.101\times 10^{-2}$	$8.518\times 10^{-3}$	$3.344\times 10^{-4}$
ParVI-SDF	$1.715\times 10^{-2}$	$6.561\times 10^{-3}$	$6.111\times 10^{-3}$	$5.325\times 10^{-3}$	$1.389\times 10^{-5}$
WAIG-SDF	$1.631\times 10^{-2}$	$6.221\times 10^{-3}$	$5.820\times 10^{-3}$	$5.148\times 10^{-3}$	$1.231\times 10^{-5}$
DPVI-DK-SDF	$1.434\times 10^{-2}$	$6.488\times 10^{-3}$	$6.115\times 10^{-3}$	$5.327\times 10^{-3}$	$1.286\times 10^{-5}$
DPVI-CA-SDF	$1.485\times 10^{-2}$	$6.423\times 10^{-3}$	$6.021\times 10^{-3}$	$5.359\times 10^{-3}$	$9.181\times 10^{-6}$
WGAD-DK-SDF	$1.431\times 10^{-2}$	$6.207\times 10^{-3}$	$5.890\times 10^{-3}$	$5.190\times 10^{-3}$	$1.204\times 10^{-5}$
WGAD-CA-SDF	$1.394\times 10^{-2}$	$6.078\times 10^{-3}$	$5.721\times 10^{-3}$	$5.143\times 10^{-3}$	$8.744\times 10^{-6}$

**Figure A1:**
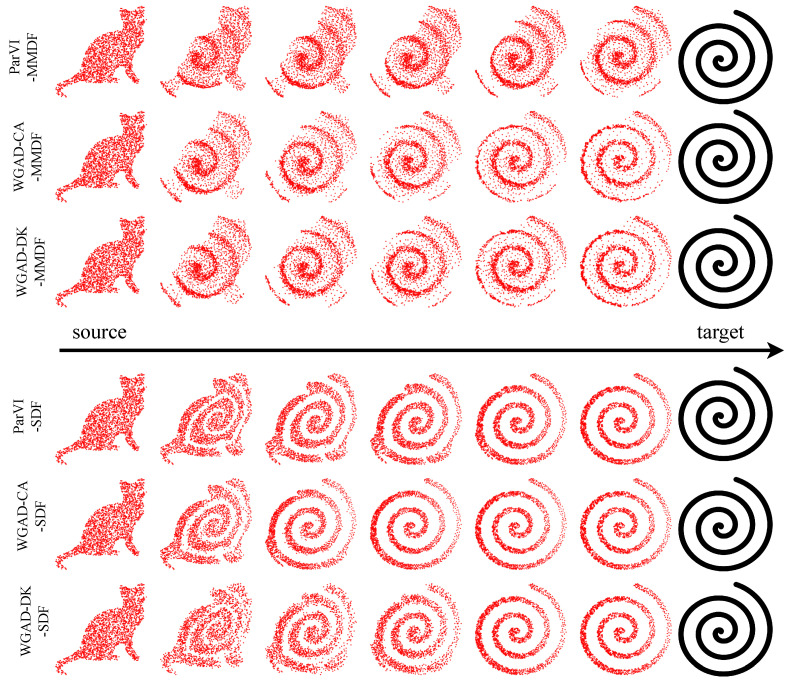
The shape morphing of the source shape CAT to the target SPIRAL.

**Figure A2:**
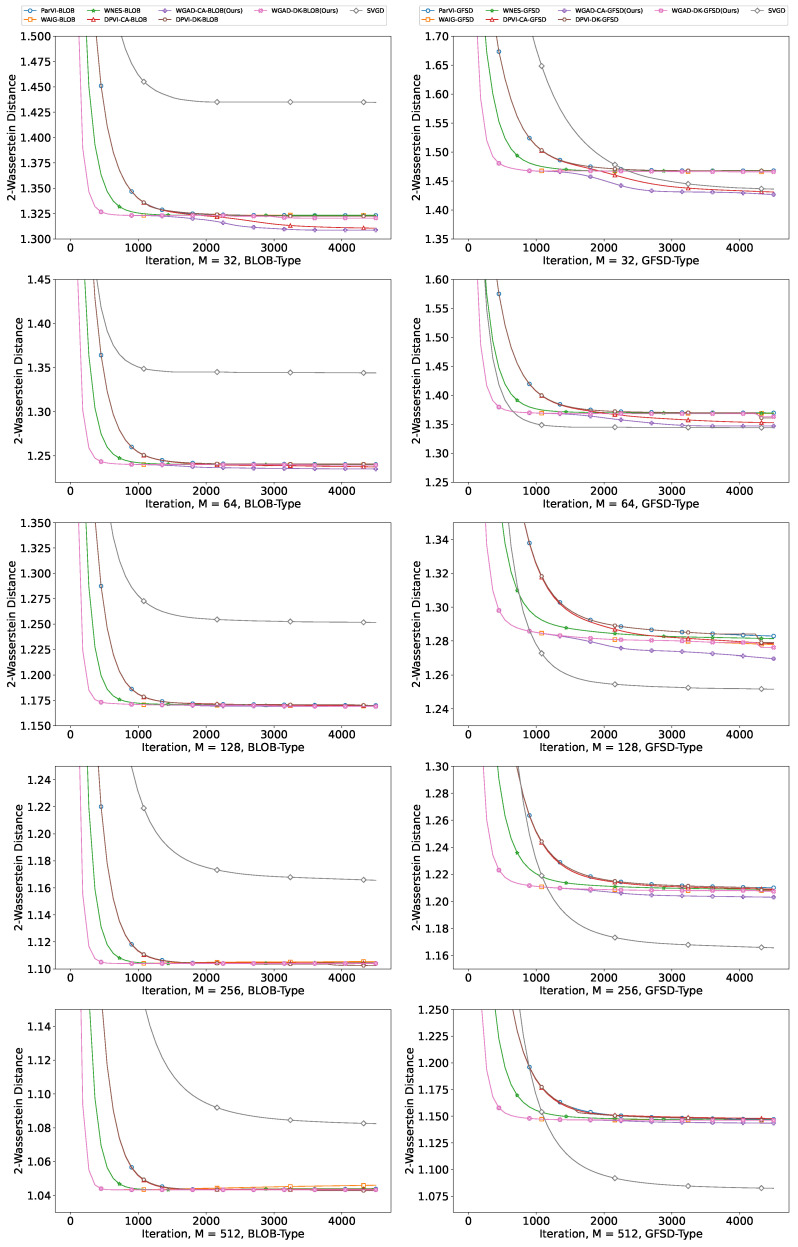
Averaged test $W_{2}$ distance to the target with respect to iterations in the SG task for algorithms (W-Type).

**Figure A3:**
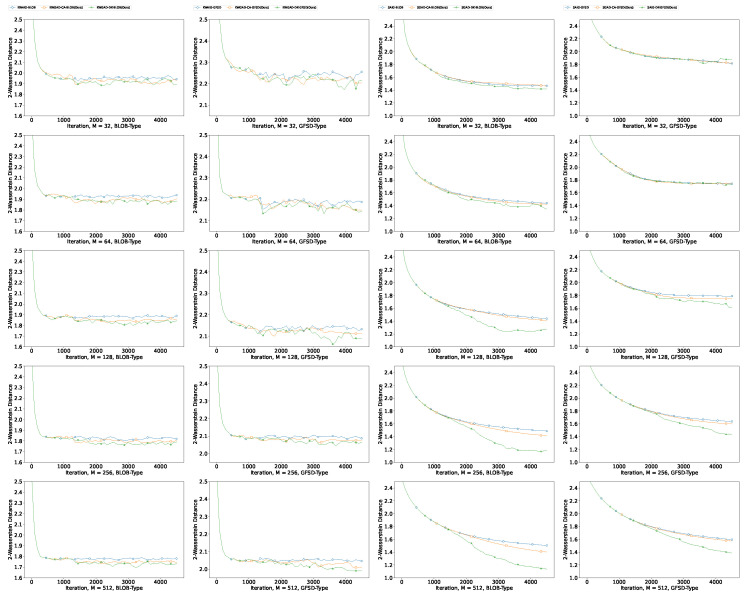
Averaged test $W_{2}$ distance to the target with respect to iterations in the SG task for algorithms (KW/S-Type).

**Figure A4:**
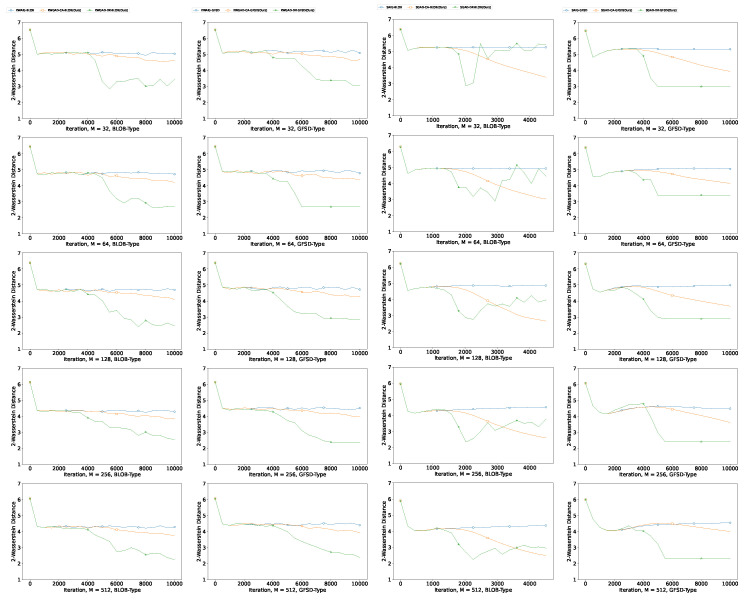
Averaged test $W_{2}$ distance to the target with respect to iterations in the GMM task for algorithms (KW/S-Type).

**Figure A5:**
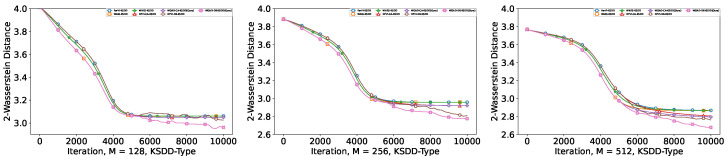
Averaged test $W_{2}$ distance to the target with respect to iterations in the SG task for algorithms (WGAD-KSDD-Type).

## Data Availability

The original contributions presented in the study are included in the article/Supplementary Material, further inquiries can be directed to the corresponding author(s).

## Supplementary Materials

The following supporting information can be downloaded at: https://www.mdpi.com/article/10.3390/e26080679/s1.

## Abbreviations

    The following abbreviations are used in this manuscript:

MCMC	Markov Chain Monte Carlo
ParVI	Particle-based Variational Inference
DPVI	Dynamic-weight Particle-based Variational Inference
IFR	Information–Fisher–Rao
SHIFR	Semi-Hamiltonian Information–Fisher–Rao
WFR	Wasserstein–Fisher–Rao
GAD-PVI	General Accelerated Dynamic-weight Particle-based Variational Inference
SVGD	Stein Variational Gradient Descent
GFSD	Gradient Flow with Smoothed Density
KSDD	Kernel Setin Discrepancy Descent
WAG	Wasserstein Accelerated Gradien
ACCEL	The Accelerated Flow method
WNES	Wasserstein Nesterov’s
AIG	Accelerated Information Gradient Flow
MMD	Maximum Mean Distance
MMDF	Maximum Mean Distance Flow
SD	Sinkhorn Divergence
SDF	Sinkhorn Divergence Flow
NSGF	Neural Sinkhorn Gradient Flow
JSD	J-JKO

## Appendix A. Definitions and Proofs

### Appendix A.1. Definition of Information Metric in Probability Space

To ensure the definition of the general information gradient flow in the probability space, we provide a brief review of the general information metrics in the probability space, as proposed by the studies of [37,59]:
**Definition** **A1** (Information metric in probability space)**.** *Denote the tangent space at $\mu \in P(R^{n})$ by $T_{\mu }P\left(R^{n}\right)=\left\{\sigma \in C\left(R^{n}\right):\int \sigma dx=0\right\}$. The cotangent space at μ is denoted as $T_{\mu }^{*}P\left(R^{n}\right)$ and can be treated as the quotient space $C(R^{n})/R$. An information metric tensor $G\left(\mu \right)\left[\cdot \right]:T_{\mu }P\left(R^{n}\right)\to T_{\mu }^{*}P\left(R^{n}\right)$ is an invertible mapping from $P(R^{n})$ to $T_{\mu }^{*}P\left(R^{n}\right)$. This information metric tensor defines the information metric (as well as the inner product) on tangent space $P(R^{n})$, for $\sigma_{1},\sigma_{2}\in T_{\mu }P\left(R^{n}\right)$ and $\Phi_{i}=G\left(\mu \right)\left[\sigma_{i}\right],i=1,2$ as*

(A1) $$\begin{array}{c} g_{\mu }\left(\sigma_{1},\sigma_{2}\right)=\int \sigma_{1}G\left(\mu \right)\sigma_{2}dx=\int \Phi_{1}G{\left(\mu \right)}^{-1}\Phi_{2}dx, \end{array}$$

Then, we denote the general information probability space as $\left(P\left(R^{n}\right),G\left(\mu \right)\right)$. As long as a metric is specified, the probability space $P(R^{n})$ together with the metric can be taken as an infinite dimensional Riemannian manifold, which is the so-called density manifold [37], which enables the definition of gradient flow.

### Appendix A.2. Full Hamiltonian Flow on the IFR Space and the Fisher–Rao Kinetic Energy

To develop ParVI methods that possess both accelerated position update and dynamical weight adjustment simultaneously, a natural choice is to directly simulate the Hamiltonian flow on the augmented IFR space By substituting the IFR metric (20) into the general Hamiltonian flow (6), we derive the full Hamiltonian flow on the IFR space as the direct accelerated probabilistic flow on the IFR space with the form

(A2) $$\begin{array}{c} \left\{\begin{array}{cc} \; & \partial_{t}\mu_{t}\;=G{\left(\mu_{t}\right)}^{-1}\left[\Phi_{t}\right]\;+\;\left(\Phi_{t}\;-\;\int \Phi_{t}d\mu_{t}\right)\mu_{t}\\ \; & \partial_{t}\Phi_{t}\;+\;\gamma_{t}\Phi_{t}\;+\underset{\mathrm{Information}\;\mathrm{kinetic}\;\mathrm{energy}}{\underset{︸}{\frac{1}{2}\frac{\delta }{\delta \mu }\left(\int \Phi_{t}G{\left(\mu_{t}\right)}^{-1}\left[\Phi_{t}\right]dx\right)}}\;+\underset{\mathrm{Fisher}-\mathrm{Rao}\;\mathrm{kinetic}\;\mathrm{energy}}{\underset{︸}{\frac{1}{2}\Phi_{t}^{2}-\Phi_{t}\int \Phi_{t}d\mu_{t}}}\;+\;\frac{\delta F(\mu_{t})}{\delta \mu }\;=\;0. \end{array}\right. \end{array}$$

where the $\Phi_{t}$ is the velocity field; the $\frac{\delta F(\mu_{t})}{\delta \mu }$ represents the potential energy dissipation; the $\frac{1}{2}\frac{\delta }{\delta \mu }\left(\int \Phi_{t}G{\left(\mu_{t}\right)}^{-1}\left[\Phi_{t}\right]dx\right)$ represents the kinetic energy dissipation of information transport; and the $\frac{1}{2}\Phi_{t}^{2}-\Phi_{t}\int \Phi_{t}d\mu_{t}$ represents the kinetic energy dissipation of Fisher–Rao distortion. As far as we know, the particle formulation of the Full Hamiltonian flow on the IFR space (A2) is intractable due to the Fisher–Rao kinetic energy term. When deriving particle systems, the $\nabla \Phi_{t}$ can be straightforwardly approximated by the velocities $v_{t}^{i}$, but the Hamiltonian field $\Phi_{t}$ is hard to approximate by finite points and iteratively updated. Actually, even the particle formulation of the accelerated Fisher–Rao flow has not been derived due to great difficulty [33]. Therefore, we ignore the influence of the Fisher–Rao kinetic energy on the Hamiltonian field and derive the SHIFR (21). We point out that the Fisher–Rao kinetic energy would vanish as the flow converges to the equilibrium of $\left(\mu_{\infty }=\pi ,\Phi_{\infty }=0\right)$ which fits the behavior of kinetic energy in a physical dynamic system. Therefore, the ignorance of the Fisher–Rao kinetic energy is tenable, and the SHIFR still has the target distribution as its stationary distribution which will be shown in Proposition 1.

### Appendix A.3. Proof of Proposition 3

First, we introduce a technical lemma for the proof of Proposition 3.

**Lemma** **A1.**
*The following probability flow dynamic formulation and particle system formulation are equivalent:*

(A3)
$$\begin{array}{c} \left\{\begin{array}{cc} \; & \partial_{t}\mu_{t}\;+\nabla \cdot \left(\mu_{t}\nabla \Phi_{t}\right)=0\;\\ \; & \partial_{t}\Phi_{t}+\gamma_{t}\Phi_{t}+\frac{1}{2}{∥\nabla \Phi_{t}∥}^{2}+\frac{\delta F(\mu_{t})}{\delta \mu }=0 \end{array}\right. \end{array}$$

(A4)
$$\begin{array}{c} \left\{\begin{array}{cc} \; & \frac{d}{dt}X_{t}=V_{t}\\ \; & \frac{d}{dt}V_{t}=-\gamma_{t}V_{t}-\nabla \left(\frac{\delta F(\mu_{t})}{\delta \mu }\right)\left(X_{t}\right) \end{array}\right. \end{array}$$

**Proof.** We start with the calculation of the gradient of the kinetic term. For a twice-differentiable Φ(x), we have

(A5) $$\begin{array}{c} \frac{1}{2}\nabla {∥\nabla \Phi ∥}^{2}=\nabla^{2}\Phi \nabla \Phi =\left(\nabla \Phi \cdot \nabla \right)\nabla \Phi \end{array}$$

From (A3), we have

$$\begin{array}{c} \partial_{t}\mu_{t}+\nabla \cdot \left(\mu_{t}\nabla \Phi_{t}\right)=0 \end{array}$$

which is the continuity equation of $\mu_{t}$ under vector field $\nabla \Phi_{t}$ [71]. Hence, we have the following equation on the particle level (denoting $V_{t}=\nabla \Phi_{t}\left(X_{t}\right)$):

$$\begin{array}{cc} \frac{d}{dt}X_{t} & =\nabla \Phi_{t}\left(X_{t}\right)\\ \; & =V_{t} \end{array}$$

Then, the vector field shall follow:

$$\begin{array}{cc} \frac{d}{dt}V_{t} & =\frac{d}{dt}\nabla \Phi_{t}\left(X_{t}\right)\\ \; & \overset{\left(1\right)}{=}\left(\partial_{t}+\nabla \Phi_{t}\left(X_{t}\right)\cdot \nabla \right)\nabla \Phi_{t}\left(X_{t}\right)\\ \; & \overset{\left(2\right)}{=}-\gamma_{t}\nabla \Phi_{t}\left(X_{t}\right)-\frac{1}{2}\nabla {∥\nabla \Phi_{t}\left(X_{t}\right)∥}^{2}-\nabla \left(\frac{\delta F(\mu_{t})}{\delta \mu }\right)\left(X_{t}\right)+\left(\nabla \Phi_{t}\left(X_{t}\right)\cdot \nabla \right)\nabla \Phi_{t}\left(X_{t}\right)\\ \; & \overset{\left(3\right)}{=}-\gamma_{t}\nabla \Phi_{t}\left(X_{t}\right)-\nabla \left(\frac{\delta F(\mu_{t})}{\delta \mu }\right)\left(X_{t}\right)\\ \; & \overset{\left(4\right)}{=}-\gamma_{t}V_{t}-\nabla \left(\frac{\delta F(\mu_{t})}{\delta \mu }\right)\left(X_{t}\right) \end{array}$$

where Equation (1) becomes valid from material derivative in fluid dynamic [72], Equation (2) comes from the PDEs of $\Phi_{t}$ in (A3), Equation (3) comes from canceling terms on each side of (A5), and Equation (4) comes from the definition of $V_{t}$.    □

Now, we are ready to give the proof of Proposition 3.

**Proof of Proposition** **3.** Let $\Psi :P_{2}\left(R^{d}\right)\to R$ be a functional on the probability space, and let ${\tilde{\mu }}_{t}^{M}$ be the distribution produced by the continuous-time composite flow (25) at time *t*. With $\mu_{t}$ denoting the mean-field limit of ${\tilde{\mu }}_{t}^{M}$ as M→∞, we have

$$\begin{array}{c} \partial_{t}\Psi \left[\mu_{t}\right]=\left(L\Psi \right)\left[\mu_{t}\right], \end{array}$$

where

(A6) $$\begin{array}{c} L\Psi \left[\mu \right]=\int \langle \nabla \Phi \left(x\right),\nabla_{x}\frac{\delta \Psi (\mu )}{\delta \mu }\left(x\right)\rangle \mu \left(x\right)dx-\int \frac{\delta \Psi (\mu )}{\delta \mu }\left(x\right)\left(\frac{\delta F(\mu )}{\delta \mu }\left(x\right)-E_{\mu }\left[\frac{\delta F(\mu )}{\delta \mu }\left(x\right)\right]\right)\mu \left(x\right)dx, \end{array}$$

in which $\Phi_{t}$ abides by

$$\begin{array}{c} \left\{\begin{array}{cc} \; & \partial_{t}\Phi_{t}+\gamma_{t}\Phi_{t}+\frac{1}{2}{∥\nabla \Phi_{t}∥}^{2}+\frac{\delta F(\mu_{t})}{\delta \mu }=0\\ \; & \Phi_{0}=0 \end{array}\right. \end{array}$$

and $\frac{\delta \Psi (\mu )}{\delta \mu }\left(\cdot \right)$ denotes the first variation of functional Ψ at μ satisfying

$$\begin{array}{c} \int \frac{\delta \Psi (\mu )}{\delta \mu }\left(x\right)\xi \left(x\right)dx=\lim_{\epsilon \to 0}\frac{\Psi (\mu +\epsilon \xi )-\Psi (\mu )}{\epsilon } \end{array}$$

for all signed measures ∫ξ(x)dx=0. Let $\left(L^{Pos}\Psi \right)\left[\mu \right]$ be the first term of (A6), and let $\left(L^{Wei}\Psi \right)\left[\mu \right]$ be the second term of (A6). We have

$$\begin{array}{c} L\Psi \left[\mu \right]=\left(L^{Pos}\Psi \right)\left[\mu \right]+\left(L^{Wei}\Psi \right)\left[\mu \right] \end{array}$$

For the measure-valued composite flow ${\tilde{\mu }}_{t}^{M}$ (25), the infinitesimal generator of Ψ with respect to ${\tilde{\mu }}_{t}^{M}$ is defined as follows:

$$\begin{array}{c} \left(L_{M}\Psi \right)\left[{\tilde{\mu }}^{M}\right]:=\lim_{t\to 0^{+}}\frac{E_{{\tilde{\mu }}_{0}^{M}={\tilde{\mu }}^{M}}\left(\Psi \left[{\tilde{\mu }}_{t}^{M}\right]\right)-\Psi \left({\tilde{\mu }}^{M}\right)}{t}, \end{array}$$

where $E_{{\tilde{\mu }}_{0}^{M}={\tilde{\mu }}^{M}}\left(\Psi \left[{\tilde{\mu }}_{t}^{M}\right]\right)$ denotes the expectation of the functional Ψ evaluated along the trajectory ${\tilde{\mu }}_{t}^{M}$ taken conditional on the initialization ${\tilde{\mu }}_{0}^{M}={\tilde{\mu }}^{M}$. As

$$\begin{array}{cc} E_{{\tilde{\mu }}_{0}^{M}={\tilde{\mu }}^{M}}\left(\Psi \left[{\tilde{\mu }}_{t}^{M}\right]\right)-\Psi \left({\tilde{\mu }}^{M}\right)= & \underset{\mathrm{weight}\;\mathrm{adjusting}\;\mathrm{infinitesimal}}{\underset{︸}{E_{{\tilde{\mu }}_{0}^{M}={\tilde{\mu }}^{M}}\left(\Psi \left[\sum_{i=1}^{M}w_{t}^{i}\delta_{x_{t}^{i}}\right]\right)-E_{{\tilde{\mu }}_{0}^{M}={\tilde{\mu }}^{M}}\left(\Psi \left[\sum_{i=1}^{M}w_{0}^{i}\delta_{x_{t}^{i}}\right]\right)}}\\ & +\underset{\mathrm{position}\;\mathrm{adjusting}\;\mathrm{infinitesimal}}{\underset{︸}{E_{{\tilde{\mu }}_{0}^{M}={\tilde{\mu }}^{M}}\left(\Psi \left[\sum_{i=1}^{M}w_{0}^{i}\delta_{x_{t}^{i}}\right]\right)-\Psi \left(\sum_{i=1}^{M}w_{0}^{i}\delta_{x_{0}^{i}}\right)}}, \end{array}$$

we follow the same idea from [23,41,73] to divide $\left(L_{M}\Psi \right)\left[{\tilde{\mu }}^{M}\right]$ into two parts $\left(L_{M}^{Pos}\Psi \right)\left[{\tilde{\mu }}^{M}\right]$, and $\left(L_{M}^{Wei}\Psi \right)\left[{\tilde{\mu }}^{M}\right]$ corresponds to the position update and the weight adjustment, respectively. According to the definition of the first variation, it can be calculated that

$$\begin{array}{cc} \left(L_{M}^{Wei}\Psi \right)\left[{\tilde{\mu }}^{M}\right] & =\lim_{t\to 0^{+}}\frac{E_{{\tilde{\mu }}_{0}^{M}={\tilde{\mu }}^{M}}\left(\Psi \left[\sum_{i=1}^{M}w_{t}^{i}\delta_{x_{t}^{i}}\right]\right)-E_{{\tilde{\mu }}_{0}^{M}={\tilde{\mu }}^{M}}\left(\Psi \left[\sum_{i=1}^{M}w_{0}^{i}\delta_{x_{t}^{i}}\right]\right)}{t}\\ & =\int \frac{\delta \Psi (\mu^{M})}{\delta \mu }\left(x\right)\sum_{i=1}^{M}\partial_{t}\rho \left(w_{t}^{i}x_{0}^{i}\right)dx\\ & =-\int \frac{\delta \Psi (\mu^{M})}{\delta \mu }\left(x\right)\left(\frac{\delta F(\mu^{M})}{\delta \mu }\left(x\right)-E_{\mu^{M}}\left[\frac{\delta F(\mu^{M})}{\delta \mu }\left(x\right)\right]\right)\mu^{M}\left(x\right)dx. \end{array}$$

and

$$\begin{array}{cc} \left(L_{n}^{Pos}\Psi \right)\left[{\tilde{\mu }}^{M}\right] & =\lim_{t\to 0^{+}}\frac{E_{{\tilde{\mu }}_{0}^{M}={\tilde{\mu }}^{M}}\left(\Psi \left[\sum_{i=1}^{M}w_{0}^{i}\delta_{x_{t}^{i}}\right]\right)-\Psi \left(\sum_{i=1}^{M}w_{0}^{i}\delta_{x_{0}^{i}}\right)}{t}\\ & =\int \frac{\delta \Psi (\mu^{M})}{\delta \mu }\left(x\right)\sum_{i=1}^{M}w_{0}^{i}\partial_{t}\rho \left(x_{t}^{i}\right)dx\\ & =\int \langle V_{\mu^{M}}\left(x\right),\nabla_{x}\frac{\delta \Psi (\mu^{M})}{\delta \mu }\left(x\right)\rangle \mu^{M}\left(x\right)dx \end{array}$$

where $V_{\mu^{M}}$ abides by

$$\begin{array}{c} \left\{\begin{array}{cc} \; & \frac{dV_{\mu^{M}}}{dt}=-\gamma_{t}V_{\mu^{M}}-\nabla \frac{\delta F(\mu^{M})}{\delta \mu }\\ \; & V_{\mu_{0}^{M}}=0 \end{array}\right. \end{array}$$

Combining the above equalities, we have

$$\begin{array}{cc} L_{M}\Psi \left[\mu_{M}\right]= & L_{M}^{Pos}\Psi \left[\mu_{M}\right]+L_{M}^{Wei}\Psi \left[\mu_{M}\right] \end{array}$$

If we take the limit of $L_{M}\Psi \left[\mu_{M}\right]$ as M→∞ on a sequence such that $\mu^{M}⇀\mu$ (i.e., $\mu^{M}$ weakly converges to μ) a.s., and $\frac{\delta F(\mu^{M})}{\delta \mu }⇀\frac{\delta F(\mu )}{\delta \mu }$ a.s., we can deduce that $V_{\mu^{M}}⇀\nabla \Phi$ in light of Lemma A1. These allow us to conclude that $L_{M}^{Wei}\Psi \left[\mu_{M}\right]\to L^{Wei}\Psi \left[\mu \right]$ and $L_{M}^{Pos}\Psi \left[\mu_{M}\right]\to L^{Pos}\Psi \left[\mu \right]$, thus $L_{M}\Psi \left[\mu_{M}\right]\to L\Psi \left[\mu \right]$. Since $\partial_{t}\Psi \left(\mu_{t}^{M}\right)=L_{M}\Psi \left[\mu^{M}\right]$ and $\partial_{t}\Psi \left(\mu_{t}\right)=L_{M}\Psi \left[\mu_{t}\right]$, we have

$$\begin{array}{c} \lim_{n\to \infty }\Psi \left(\mu_{t}^{M}\right)=\Psi \left(\mu_{t}\right), \end{array}$$

which indicates that $\mu_{t}^{M}⇀\mu_{t}$ if $\mu_{0}^{M}⇀\mu_{0}$. Since $\mu_{t}$ satisfying $\partial_{t}\Psi \left(\mu_{t}\right)=L\Psi \left[\mu_{t}\right]$ solves the partial differential Equation (24), we conclude that the path of (25) starting from ${\tilde{\mu }}_{0}^{M}$ weakly converges to a solution of the partial differential Equation (24) starting from $\mu_{0}$ as M→∞.    □

### Appendix A.4. Proof of Proposition 1

**Proof.** The SHIFR flow under an the information metric is written as

(A7) $$\begin{array}{c} \left\{\begin{array}{cc} \; & \partial_{t}\mu_{t}=G^{I}{\left(\mu_{t}\right)}^{-1}\left[\Phi_{t}\right]-\;\left(\frac{\delta F(\mu_{t})}{\delta \mu }\;-\;\int \frac{\delta F(\mu_{t})}{\delta \mu }d\mu_{t}\right)\mu_{t},\\ \; & \partial_{t}\Phi_{t}+\gamma_{t}\Phi_{t}+\frac{1}{2}\frac{\delta }{\delta \mu }\left(\int \Phi_{t}G^{I}{\left(\mu_{t}\right)}^{-1}\left[\Phi_{t}\right]dx\right)+\frac{\delta F(\mu_{t})}{\delta \mu }=0. \end{array}\right. \end{array}$$

Because the functional F(·) is specified as some dissimilarity functional D(·|π), we have

$$\begin{array}{c} \frac{\delta F(\mu_{\infty })}{\delta \mu }=\frac{\delta F(\pi )}{\delta \mu }=\frac{\delta D(\pi ,\pi )}{\delta \mu }=0. \end{array}$$

From the element of gradient flow, we also have

$$\begin{array}{c} G^{I}{\left(\mu \right)}^{-1}\left[\Phi_{\infty }\right]=G^{I}{\left(\mu \right)}^{-1}\left[0\right]=0. \end{array}$$

Substituting into (A7) that $\left(\mu_{\infty }=\pi ,\Phi_{\infty }=0\right)$, we can obtain

(A8) $$\begin{array}{cc} \; & {\left.\partial_{t}\Phi_{t}\right|}_{t=\infty }=-\gamma_{\infty }\Phi_{\infty }-\frac{1}{2}\frac{\delta }{\delta \mu }\left(\int \Phi_{\infty }G^{I}{\left(\mu_{t}\right)}^{-1}\left[\Phi_{\infty }\right]dx\right)-\frac{\delta F(\mu_{\infty })}{\delta \mu }=0, \end{array}$$

(A9) $$\begin{array}{cc} \; & {\left.\partial_{t}\mu_{t}\right|}_{t=\infty }=G^{I}{\left(\mu_{\infty }\right)}^{-1}\left[\Phi_{\infty }\right]-\;\left(\frac{\delta F(\mu_{\infty })}{\delta \mu }\;-\;\int \frac{\delta F(\mu_{\infty })}{\delta \mu }d\mu_{\infty }\right)\mu_{\infty }=0. \end{array}$$

These suffice for proof.    □

### Appendix A.5. Proof of Proposition 2

**Proof.** For the W-SHIFR case, according to the result in (A6), the following eqaulity holds for any functional $\Psi :P_{2}\left(R^{d}\right)\to R$ on the probability space $P_{2}\left(R^{d}\right)$, where

$$\begin{array}{cc} \partial_{t}\Psi \left[\mu_{t}^{SHIFR}\right] & =\int \langle \nabla \Phi \left(x\right),\nabla_{x}\frac{\delta \Psi (\mu_{t}^{SHIFR})}{\delta \mu }\left(x\right)\rangle \mu_{t}^{SHIFR}\left(x\right)dx\\ & -\int \frac{\delta \Psi (\mu_{t}^{SHIFR})}{\delta \mu }\left(x\right)\left(\frac{\delta F(\mu_{t}^{SHIFR})}{\delta \mu }\left(x\right)-E_{\mu }\left[\frac{\delta F(\mu_{t}^{SHIFR})}{\delta \mu }\left(x\right)\right]\right)\mu_{t}^{SHIFR}\left(x\right)dx, \end{array}$$

in which ${\left.\mu_{t}\right|}_{t=0}=\bar{\mu }$ and $\Phi_{t}$ abides by

$$\begin{array}{c} \left\{\begin{array}{cc} \; & \partial_{t}\Phi_{t}+\gamma_{t}\Phi_{t}+\frac{1}{2}{∥\nabla \Phi_{t}∥}^{2}+\frac{\delta F(\mu_{t})}{\delta \mu }=0\\ \; & {\left.\Phi_{t}\right|}_{t=0}=\bar{\Phi } \end{array}\right. \end{array}$$

By substituting $\Psi \left(\mu_{t}^{SHIFR}\right)=F\left(\mu_{t}^{SHIFR}\right)$, $U_{\mu_{t}^{SHIFR}}=\frac{\delta F(\mu_{t}^{SHIFR})}{\delta \mu }$ and t=0 in the above equality, we have

(A10) $$\begin{array}{ccc} {\left.\frac{dF(\mu_{t}^{\mathrm{SHIFR}})}{dt}\right|}_{t=0} & = & -\int \langle \nabla_{x}\frac{\delta F(\bar{\mu })}{\delta \mu }\left(x\right),\nabla \bar{\Phi }\left(x\right)\rangle \bar{\mu }\left(x\right)\;dx\\ & & -\int \frac{\delta F(\bar{\mu })}{\delta \mu }\left(x\right)\left(U_{\bar{\mu }}\left(x\right)-E_{\bar{\mu }}\left[U_{\bar{\mu }}\left(x\right)\right]\right)\bar{\mu }\left(x\right)\;dx\\ & = & -\int \langle \nabla_{x}\frac{\delta F(\bar{\mu })}{\delta \mu }\left(x\right),\nabla \bar{\Phi }\left(x\right)\rangle \bar{\mu }\left(x\right)\;dx\\ & & -\left(\int {\left(\frac{\delta F(\bar{\mu })}{\delta \mu }\left(x\right)\right)}^{2}\bar{\mu }\left(x\right)\;dx-{\left(\int \frac{\delta F(\bar{\mu })}{\delta \mu }\left(x\right)\bar{\mu }\left(x\right)\;dx\right)}^{2}\right). \end{array}$$

Similarly, we can obtain the following result for the Hamiltonian flow in the non-augmented Wasserstein space case:

(A11) $$\begin{array}{cc} {\left.\frac{dF(\mu_{t}^{H})}{dt}\right|}_{t=0}= & -\int \langle \nabla_{x}\frac{\delta F(\bar{\mu })}{\delta \mu }\left(x\right),\nabla \bar{\Phi }\left(x\right)\rangle \bar{\mu }\left(x\right)dx. \end{array}$$

Since the second term of (A10) is always less than or equal to zero and the first term of (A10) is the same as the first term of (A11), we can reach to the conclusion that the local dissipation of SHIFR flow has an additional functional dissipation term compared to the Hamiltonian flow in the non-augmented space:

$$\begin{array}{c} {\left.\frac{dF(\mu_{t}^{SHIFR})}{dt}\right|}_{t=0}{\left.\leq \frac{dF(\mu_{t}^{H})}{dt}\right|}_{t=0} \end{array}$$

For general information, readers can follow the same routine to obtain the extra functional dissipation property.    □

## Appendix B. Detailed Formulations

### Appendix B.1. Kalman–Wasserstein–SHIFR Flow and KWGAD-PVI Algorithms

Combining the Kalman filter to estimate the probability distributions of a dynamic system over time and the Wasserstein metric to measure the difference between these estimated distributions, the Kalman–Wasserstein metric is proposed in ensemble Kalman sampling literature [74]. The inverse of the Kalman–Wasserstein metric tensor is written as

(A12) $$\begin{array}{c} G^{KW}{\left(\mu \right)}^{-1}\left[\Phi \right]=-\nabla \cdot \left(\mu C^{\lambda }\left(\mu \right)\nabla \Phi \right),\Phi \in T_{\mu }^{*}P\left(R^{n}\right), \end{array}$$

where $\Phi \in T_{\mu }^{*}P\left(R^{n}\right)$ and, substituting into (2), the gradient flow of Kalman–Wasserstein metric is written as

(A13) $$\begin{array}{c} \partial_{t}\mu_{t}=G^{KW}{\left(\mu_{t}\right)}^{-1}\left[\frac{\delta F(\mu_{t})}{\delta \mu }\right]=-\nabla \cdot \left(\mu_{t}C^{\lambda }\left(\mu_{t}\right)\nabla \frac{\delta F(\mu_{t})}{\delta \mu }\right). \end{array}$$

where λ≥0 is the regularization constant and $C^{\lambda }\left(\mu \right)$ is the linear transformation as follows:

(A14) $$\begin{array}{c} C^{\lambda }\left(\mu \right)=\int \left(x-m\left(\mu \right)\right){\left(x-m\left(\mu \right)\right)}^{T}\mu dx+\lambda I,m\left(\mu \right)=\int x\mu dx. \end{array}$$

Substituting the Kalman–Wasserstein metric into the SHIFR flow (21) gives the Kalman–Wasserstein–SHIFR flow:

(A15) $$\begin{array}{c} \left\{\begin{array}{cc} \; & \partial_{t}\mu_{t}\;=-\nabla \cdot \left(\mu_{t}C^{\lambda }\left(\mu_{t}\right)\nabla \Phi_{t}\right)\;-\;\left(\frac{\delta F(\mu_{t})}{\delta \mu }\;-\;\int \frac{\delta F(\mu_{t})}{\delta \mu }d\mu_{t}\right)\mu_{t},\\ \; & \partial_{t}\Phi_{t}+\gamma_{t}\Phi_{t}+\frac{1}{2}\left({\left(x-m\left(\mu_{t}\right)\right)}^{T}\int \nabla \Phi_{t}\nabla \Phi_{t}^{T}d\mu_{t}\left(x-m\left(\mu_{t}\right)\right)+\nabla \Phi_{t}^{T}C^{\lambda }\left(\mu_{t}\right)\nabla \Phi_{t}\right)\\ \; & +\frac{\delta F(\mu_{t})}{\delta \mu }=0. \end{array}\right. \end{array}$$

We claim that the finite-particles formulation of the Kalman–Wasserstein–SHIFR flow (A15) evolves the positions $x^{i}$’s, the  weights $w^{i}$’s of *M* particles, and velocity field v as follows:

(A16) $$\begin{array}{c} \left\{\begin{array}{cc} dx_{t}^{i} & =C^{\lambda }\left({\tilde{\mu }}_{t}\right)v_{t}^{i}dt,\\ dv_{t}^{i} & =(-\gamma v_{t}^{i}-E\left[v_{t}v_{t}^{T}\right]\left(x^{i}-E\left[x\right]\right)-\nabla \frac{\delta F({\tilde{\mu }}_{t})}{\delta \mu }\left(x_{t}^{i}\right))dt,\\ dw_{t}^{i} & =-\left(\frac{\delta F({\tilde{\mu }}_{t})}{\delta \mu }\left(x_{t}^{i}\right)-\sum_{i=1}^{M}w_{t}^{i}\frac{\delta F({\tilde{\mu }}_{t})}{\delta \mu }\left(x_{t}^{i}\right)\right)w_{t}^{i}dt,\\ {\tilde{\mu }}_{t} & =\sum_{i=1}^{M}w_{t}^{i}\delta_{x_{t}^{i}}. \end{array}\right. \end{array}$$

Here, the expectation is taken over the empirical distribution of particles.

Then, the proposition below shows the mean-field limit of the finite-particles formulation (A16) is exactly the Kalman–Wasserstein–SHIFR flow (A15).

**Proposition** **A1.**
*Suppose the empirical distribution ${\tilde{\mu }}_{0}^{M}$ of M weighted particles weakly converges to a distribution $\mu_{0}$ when M→∞. Then, the path of (A16) starting from ${\tilde{\mu }}_{0}^{M}$ and $\Phi_{0}$ with initial velocity 0 weakly converges to a solution of the Kalman–Wasserstein–SHIFR gradient flow (A15) starting from $\mu_{t}{|}_{t=0}=\mu_{0}=$ and $\Phi_{t}{|}_{t=0}=0$ as M→∞:*

Similar to the proof scheme of Proposition 3, we start by proving a technical lemma first:

**Lemma** **A2.**
*The following fluid dynamic formulation and particle dynamic formulation are equivalent:*

*(Suppose that $X_{t}∼\mu_{t}$ and $V_{t}=\nabla \Phi_{t}\left(X_{t}\right)$, expectation is taken over particles)*

(A17)
$$\begin{array}{c} \left\{\begin{array}{cc} \; & \partial_{t}\mu_{t}\;+\nabla \cdot \left(\mu_{t}C^{\lambda }\left(\mu_{t}\right)\nabla \Phi_{t}\right)=0,\;\\ \; & \partial_{t}\Phi_{t}+\gamma_{t}\Phi_{t}+\frac{1}{2}({\left(x-m\left(\mu_{t}\right)\right)}^{T}\int \nabla \Phi_{t}\nabla \Phi_{t}^{T}d\mu_{t}\left(x-m\left(\mu_{t}\right)\right)\\ \; & +\nabla \Phi_{t}^{T}C^{\lambda }\left(\mu_{t}\right)\nabla \Phi_{t})+\frac{\delta F(\mu_{t})}{\delta \mu }=0. \end{array}\right. \end{array}$$

(A18)
$$\begin{array}{c} \left\{\begin{array}{cc} \; & \frac{d}{dt}X_{t}=C^{\lambda }\left(\mu_{t}\right)V_{t},\\ \; & \frac{d}{dt}V_{t}=-\gamma_{t}V_{t}-E\left[V_{t}V_{t}^{T}\right]\left(X_{t}-E\left[X_{t}\right]\right)-\nabla \left(\frac{\delta F(\mu_{t})}{\delta \mu }\right)\left(X_{t}\right). \end{array}\right. \end{array}$$

**Proof.** First, we establish two equations. For i=1⋯n, we have

(A19) $$\begin{array}{c} \begin{array}{cc} \left(C^{\lambda }\left(\mu_{t}\right)\nabla \Phi_{t}\cdot \nabla \right)\nabla_{i}\Phi_{t}\left(X_{t}\right)\; & =\sum_{j=1}^{n}{\left(C^{\lambda }\left(\mu_{t}\right)\nabla \Phi_{t}\right)}_{j}\nabla_{j}\nabla_{i}\Phi_{t}\left(X_{t}\right)\\ \; & =\sum_{j=1}^{n}\nabla_{ij}\Phi_{t}\left(X_{t}\right){\left(C^{\lambda }\left(\mu_{t}\right)\nabla \Phi_{t}\right)}_{j}\\ \; & ={\left(\nabla^{2}\Phi_{t}C^{\lambda }\left(\mu_{t}\right)\nabla \Phi_{t}\right)}_{i}. \end{array} \end{array}$$

Them, according to the chain rule, we have

(A20) $$\begin{array}{c} \begin{array}{c} \nabla \left(\nabla \Phi_{t}{\left(x\right)}^{T}C^{\lambda }\left(\mu_{t}\right)\nabla \Phi_{t}\left(x\right)\right)=2\nabla^{2}\Phi_{t}\left(x\right)C^{\lambda }\left(\mu_{t}\right)\nabla \Phi_{t}\left(x\right). \end{array} \end{array}$$

Since the first equation of (A17) is actually the continuity equation with velocity field $C^{\lambda }\left(\mu_{t}\right)\nabla \Phi_{t}$, it is obvious that we have $\frac{d}{dt}X_{t}=C^{\lambda }\left(\mu_{t}\right)V_{t}$. Then, we deduce the second equation of (A18):

$$\begin{array}{cc} \frac{d}{dt}V_{t} & =\frac{d}{dt}\nabla \Phi_{t}\left(X_{t}\right)\\ \; & \overset{\left(1\right)}{=}\left(\partial_{t}+C^{\lambda }\left(\mu_{t}\right)\nabla \Phi_{t}\cdot \nabla \right)\nabla \Phi_{t}\left(X_{t}\right)\\ \; & \overset{\left(2\right)}{=}\partial_{t}\nabla \Phi_{t}+\nabla^{2}\Phi_{t}C^{\lambda }\left(\mu_{t}\right)\nabla \Phi_{t}\\ \; & \overset{\left(3\right)}{=}-\gamma_{t}\nabla \Phi_{t}-\int \nabla \Phi_{t}\nabla \Phi_{t}^{T}d\mu_{t}\left(x-m\left(\mu_{t}\right)\right)-\frac{1}{2}\nabla \left(\nabla \Phi_{t}{\left(x\right)}^{T}C^{\lambda }\left(\mu_{t}\right)\nabla \Phi_{t}\left(x\right)\right)\\ \; & -\nabla \left(\frac{\delta F(\mu_{t})}{\delta \mu }\right)\left(X_{t}\right)+\nabla^{2}\Phi_{t}C^{\lambda }\left(\mu_{t}\right)\nabla \Phi_{t}\\ & \overset{\left(4\right)}{=}-\gamma_{t}\nabla \Phi_{t}-\int \nabla \Phi_{t}\nabla \Phi_{t}^{T}d\mu_{t}\left(x-m\left(\mu_{t}\right)\right)-\nabla \left(\frac{\delta F(\mu_{t})}{\delta \mu }\right)\left(X_{t}\right)\\ & \overset{\left(5\right)}{=}-\gamma_{t}V_{t}-E\left[V_{t}V_{t}^{T}\right]\left(X_{t}-E\left[X_{t}\right]\right)-\nabla \left(\frac{\delta F(\mu_{t})}{\delta \mu }\right)\left(X_{t}\right). \end{array}$$

where Equation (1) becomes valid from a material derivative in fluid dynamic [72], Equation (2) comes from Equation (A19), Equation (3) comes from the PDE (A17), Equation (4) comes from canceling terms on each side of (A20), and Equation (5) comes from the definition of $V_{t}$ and $X_{t}$.    □

**Proof of Proposition** **A1.** Substituting Lemma A1 by Lemma A2, the proof scheme of Proposition A1 is the same as the proof scheme of Proposition 3.    □

By discretizing (A16), we derive the KWGAD-PVI algorithms, which update the positions of particles according to the following rule:

(A21) $$\begin{array}{c} x_{k+1}^{i}=x_{k}^{i}+\eta_{pos}{\left[C_{k}^{\lambda }v_{k}\right]}^{i}, \end{array}$$

and adjusts the velocity field as follows:

(A22) $$\begin{array}{c} v_{k+1}^{i}=\left(1-\gamma \eta_{vel}\right)v_{k}^{i}-\frac{\eta_{vel}}{M}\left[\sum_{j=1}^{N}w_{k}^{j}\left(V_{k}^{j}\right){\left(V_{k}^{j}\right)}^{T}\right]\left(x_{k}^{i}-m_{k}\right)-\eta_{vel}\nabla U_{{\tilde{\mu }}_{k}}\left(x_{k}\right). \end{array}$$

Here, $C_{k}^{\lambda }$ and $m_{k}$ are calculated at each round by

(A23) $$\begin{array}{c} m_{k}=\frac{1}{N}\sum_{i=1}^{M}w_{k}^{i}x_{k}^{i},C_{k}^{\lambda }=\frac{1}{N-1}\sum_{i=1}^{M}w_{k}^{i}\left(x_{k}^{i}-m_{k}\right){\left(x_{k}^{i}-m_{k}\right)}^{T}+\lambda I. \end{array}$$

### Appendix B.2. Stein-SHIFR Flow and SGAD-PVI Algorithms

Involving reproducing kernel Hilbert space norm into probability space, and the Stein metric is proposed for geometrical analysis [75]. The gradient flow of Stein metric is written as

(A24) $$\begin{array}{c} \partial_{t}\mu_{t}=G^{S}{\left(\mu_{t}\right)}^{-1}\frac{\delta F(\mu_{t})}{\delta \mu }=-\nabla \cdot \left(\mu_{t}\int k\left(\cdot ,y\right)\mu_{t}\left(y\right)\nabla_{y}\frac{\delta F(\mu_{t})}{\delta \mu }\left(y\right)dy\right). \end{array}$$

Substituting the Stein metric into the SHIFR flow (21) gives the Stein-SHIFR flow:

(A25) $$\begin{array}{c} \left\{\begin{array}{cc} \; & \partial_{t}\mu_{t}\;=-\nabla \cdot \left(\mu_{t}\int k\left(\cdot ,y\right)\mu_{t}\left(y\right)\nabla_{y}\Phi_{t}\left(y\right)dy\right)-\;\left(\frac{\delta F(\mu_{t})}{\delta \mu }\;-\;\int \frac{\delta F(\mu_{t})}{\delta \mu }d\mu_{t}\right)\mu_{t},\\ \; & \partial_{t}\Phi_{t}+\gamma_{t}\Phi_{t}+\int \nabla \Phi_{t}{\left(\cdot \right)}^{T}\nabla \Phi_{t}\left(y\right)k\left(\cdot ,y\right)\mu_{t}\left(y\right)dy+\frac{\delta F(\mu_{t})}{\delta \mu }=0. \end{array}\right. \end{array}$$

We claim that the finite-particles formulation of the Stein-SHIFR flow (A25) evolves the positions $x^{i}$’s, the  weights $w^{i}$’s of *M* particles, and velocity field v as follows:

(A26) $$\begin{array}{c} \left\{\begin{array}{cc} dx_{t}^{i} & ={\left[\int k\left(x_{t},y\right)\nabla \Phi_{t}\left(y\right){\tilde{\mu }}_{t}\left(y\right)dy\right]}^{i}dt,\\ dv_{t}^{i} & =(-\gamma v_{t}^{i}-{\left[\int v_{t}^{T}\nabla \Phi_{t}\left(y\right)\nabla_{x}k\left(x_{t},y\right){\tilde{\mu }}_{t}\left(y\right)dy\right]}^{i}-\nabla \frac{\delta F({\tilde{\mu }}_{t})}{\delta \mu }\left(x_{t}^{i}\right))dt,\\ dw_{t}^{i} & =-\left(\frac{\delta F({\tilde{\mu }}_{t})}{\delta \mu }\left(x_{t}^{i}\right)-\sum_{i=1}^{M}w_{t}^{i}\frac{\delta F({\tilde{\mu }}_{t})}{\delta \mu }\left(x_{t}^{i}\right)\right)w_{t}^{i}dt,\\ {\tilde{\mu }}_{t} & =\sum_{i=1}^{M}w_{t}^{i}\delta_{x_{t}^{i}}. \end{array}\right. \end{array}$$

Then, the proposition below shows the mean-field limit of the finite-particles formulation (A26) is exactly the Stein-SHIFR flow (A25).

**Proposition** **A2.**
*Suppose the empirical distribution ${\tilde{\mu }}_{0}^{M}$ of M weighted particles weakly converges to a distribution $\mu_{0}$ when M→∞. Then, the path of (A26) starting from ${\tilde{\mu }}_{0}^{M}$ and $\Phi_{0}$ with initial velocity 0 weakly converges to a solution of the Stein-SHIFR gradient flow (A25) starting from $\mu_{t}{|}_{t=0}=\mu_{0}=$ and $\Phi_{t}{|}_{t=0}=0$ as M→∞:*

Similarly, the first proof is a technical lemma:

**Lemma** **A3.**
*The following fluid dynamic formulation and particle dynamic formulation are equivalent:*

*(Suppose that $X_{t}∼\mu_{t}$ and $V_{t}=\nabla \Phi_{t}\left(X_{t}\right)$)*

(A27)
$$\begin{array}{c} \left\{\begin{array}{cc} \; & \partial_{t}\mu_{t}\;+\nabla \cdot \left(\mu_{t}\int k\left(\cdot ,y\right)\mu_{t}\left(y\right)\nabla_{y}\Phi_{t}\left(y\right)dy\right)=0,\\ \; & \partial_{t}\Phi_{t}+\gamma_{t}\Phi_{t}+\int \nabla \Phi_{t}{\left(\cdot \right)}^{T}\nabla \Phi_{t}\left(y\right)k\left(\cdot ,y\right)\mu_{t}\left(y\right)dy+\frac{\delta F(\mu_{t})}{\delta \mu }=0. \end{array}\right. \end{array}$$

(A28)
$$\begin{array}{c} \left\{\begin{array}{cc} \; & \frac{d}{dt}X_{t}=\int k\left(X_{t},y\right)\nabla \Phi_{t}\left(y\right)\mu_{t}\left(y\right)dy,\\ \; & \frac{d}{dt}V_{t}=-\gamma_{t}V_{t}-\int V_{t}^{T}\nabla \Phi_{t}\left(y\right)\nabla_{x}k\left(X_{t},y\right)\mu_{t}\left(y\right)dy-\nabla \left(\frac{\delta F(\mu_{t})}{\delta \mu }\right)\left(X_{t}\right). \end{array}\right. \end{array}$$

**Proof.** First, we notice the following equation:

(A29) $$\begin{array}{c} \begin{array}{cc} \nabla (\int \nabla \Phi {\left(x\right)}^{T}\nabla \Phi \left(y\right)k\left(x,y\right)\mu_{t}\left(y\right)dy)\; & =\nabla^{2}\Phi \left(x\right)\int \nabla \Phi \left(y\right)k\left(x,y\right)\mu \left(y\right)dy\\ & +\int \nabla \Phi {\left(x\right)}^{T}\nabla \Phi \left(y\right)\nabla_{x}k\left(x,y\right)\mu \left(y\right)dy. \end{array} \end{array}$$

Since the first equation of (A27) is actually the continuity equation with velocity field $\int k\left(\cdot ,y\right)\mu_{t}\left(y\right)\nabla_{y}\Phi_{t}\left(y\right)dy$, it is obvious that we have $\frac{d}{dt}X_{t}=\int k\left(X_{t},y\right)\nabla \Phi_{t}\left(y\right)\mu_{t}\left(y\right)dy$. Then, we deduce the second equation of (A28):

$$\begin{array}{cc} \frac{d}{dt}V_{t} & =\frac{d}{dt}\nabla \Phi_{t}\left(X_{t}\right)\\ \; & \overset{\left(1\right)}{=}\partial_{t}\nabla \Phi_{t}\left(X_{t}\right)+\nabla^{2}\Phi_{t}\left(X_{t}\right)\left(\int k\left(X_{t},y\right)\nabla \Phi_{t}\left(y\right)\mu_{t}\left(y\right)dy\right)\\ \; & \overset{\left(2\right)}{=}-\gamma_{t}V_{t}-\nabla \left(\int \nabla \Phi {\left(x\right)}^{T}\nabla \Phi \left(y\right)k\left(x,y\right)\mu_{t}\left(y\right)dy\right)-\nabla \left(\frac{\delta F(\mu_{t})}{\delta \mu }\right)\left(X_{t}\right)\\ \; & +\nabla^{2}\Phi_{t}\left(X_{t}\right)\left(\int k\left(X_{t},y\right)\nabla \Phi_{t}\left(y\right)\mu_{t}\left(y\right)dy\right)\\ & \overset{\left(3\right)}{=}-\gamma_{t}V_{t}-\int \nabla \Phi {\left(x\right)}^{T}\nabla \Phi \left(y\right)\nabla_{x}k\left(x,y\right)\mu \left(y\right)dy-\nabla \left(\frac{\delta F(\mu_{t})}{\delta \mu }\right)\left(X_{t}\right)\\ & \overset{\left(4\right)}{=}-\gamma_{t}V_{t}-\int V_{t}^{T}\nabla \Phi_{t}\left(y\right)\nabla_{x}k\left(X_{t},y\right)\mu_{t}\left(y\right)dy-\nabla \left(\frac{\delta F(\mu_{t})}{\delta \mu }\right)\left(X_{t}\right). \end{array}$$

where Equation (1) becomes valid from material derivative in fluid dynamic [72], Equation (2) comes from the PDE (A27), Equation (3) comes from leveraging (A29), and Equation (4) comes from the definition of $V_{t}$.    □

**Proof of Proposition** **A2.** Substituting Lemma A1 by Lemma A3, the proof scheme of Proposition A2 is the same as the proof scheme of Proposition 3.    □

By discretizing (A26), Stein-GAD-PVI algorithm updates the positions of particles according to the following rule:

(A30) $$\begin{array}{c} x_{k+1}^{i}=x_{k}^{i}+\frac{\eta_{pos}}{M}\sum_{j=1}^{M}K\left(x_{k}^{i},x_{k}^{j}\right)v_{k}^{i}, \end{array}$$

and adjusts the velocity field as follows:

(A31) $$\begin{array}{c} v_{k+1}^{i}=\left(1-\gamma \eta_{vel}\right)v_{k}^{i}-\frac{\eta_{vel}}{M}\sum_{j=1}^{M}{\left(v_{k}^{i}\right)}^{T}v_{k}^{j}\nabla_{1}K\left(x_{k}^{i},x_{k}^{j}\right)-\eta_{vel}\nabla U_{{\tilde{\mu }}_{k}}\left(x_{k}\right). \end{array}$$

### Appendix B.3. GAD-PVI Algorithms in Details

By adopting different underlying information metric tensors (W-metric, KW-metic and S-metric), weight-adjustment approaches (CA and DK), and dissimilarity functionals/associated smoothing approaches (KL-BLOB, KL-GFSD, KSD-KSDD, MMD-MMDF, SD-SDF), we can derive 30 different instances of GAD-PVI, named as WGAD/KWGAD/SGAD-CA/DK-BLOB/GFSD/KSDD/MMDF/SDF. Here, we present our General Accelerated Dynamic-weight Particle-based Variational Inference (GAD-PVI) framework in a more detailed version of Algorithm 1 as Algorithm A1.

**Algorithm A1** General Accelerated Dynamic-weight Particle-based Variational Inference (GAD-PVI) framework in details
**Input**: Initial distribution ${\tilde{\mu }}_{0}=\sum_{i=1}^{M}w_{0}^{i}\delta_{x_{0}^{i}}$, position adjusting step-size $\eta_{pos}$, weight adjusting step-size $\eta_{wei}$, velocity field adjusting step-size $\eta_{vel}$, velocity damping parameter γ. 1: Choose a suitable functional F and its smoothing strategy $U_{\tilde{\mu }}\approx \frac{\delta F(\tilde{\mu })}{\delta \mu }$ from KL-BLOB/KL-GFSD/KSD-KSDD/MMD-MMDF/SD-SDF 2: **if** Under the score function accessible sampling setting **then** 3: $$\begin{array}{c} U_{\tilde{\mu }}\left(x\right)\approx \frac{\delta F(\tilde{\mu })}{\delta \mu }\left(x\right)=\left\{\begin{array}{c} -\log\pi (x)+\frac{\sum_{i=1}^{M}K\left(x,x_{k}^{i}\right)}{\sum_{i=1}^{M}K\left(x,x_{k}^{i}\right)}+\sum_{i=1}^{M}\frac{K(x,x_{k}^{i})}{\sum_{l=1}^{M}K\left(x_{k}^{i},x_{k}^{l}\right)}\;\left(\mathrm{KL}-\mathrm{BLOB}\right),\\ -\log\pi (x)+\frac{\sum_{i=1}^{M}K\left(x,x_{k}^{i}\right)}{\sum_{i=1}^{M}K\left(x,x_{k}^{i}\right)}\;\left(\mathrm{KL}-\mathrm{GFSD}\right),\\ \frac{1}{M}\sum_{i=1}^{M}k_{\pi }\left(x_{k}^{i},x\right)\;\left(\mathrm{KSD}-\mathrm{KSDD}\right). \end{array}\right. \end{array}$$ 4: **else if** Under the i.i.d. samples accessible sampling setting **then** 5: $$\begin{array}{c} U_{\tilde{\mu }}\left(x\right)\approx \frac{\delta F(\tilde{\mu })}{\delta \mu }\left(x\right)=\left\{\begin{array}{c} \sum_{i=1}^{M}w_{k}^{i}K\left(x_{k}^{i},x\right)-\sum_{j=1}^{N}a^{j}K\left(y^{j},x\right)\;\left(\mathrm{MMD}-\mathrm{MMDF}\right),\\ f_{{\tilde{\mu }}_{k},\pi }-f_{{\tilde{\mu }}_{k},{\tilde{\mu }}_{k}}\;\left(\mathrm{SD}-\mathrm{SDF}\right). \end{array}\right. \end{array}$$ 6: **end if** 7: **for** k=0,1,…,T−1 **do** 8: **for** i=1,2,…,M **do** 9: $$\begin{array}{c} \mathrm{Update}\;\mathrm{positions}\;x_{k}^{i}\;\mathrm{according}\;\mathrm{to}\;\left\{\begin{array}{c} (26)\;\left(\mathrm{WGAD}\right),\\ (A21)\;\left(\mathrm{KWGAD}\right),\\ (A30)\;\left(\mathrm{SGAD}\right). \end{array}\right. \end{array}$$ $$\begin{array}{c} \mathrm{Update}\;\mathrm{positions}\;v_{k}^{i}\;\mathrm{according}\;\mathrm{to}\;\left\{\begin{array}{c} (27)\;\left(\mathrm{WGAD}\right),\\ (A22)\;\left(\mathrm{KWGAD}\right),\\ (A31)\;\left(\mathrm{SGAD}\right). \end{array}\right. \end{array}$$ 10: **end for** 11: **if** Adopt CA strategy for weight adjustment **then** 12: Update weights $w_{k}^{i}$ according to (28) 13: **end if** 14: **if** Adopt DK strategy for weight adjustment **then** 15: **for** i=1,2,…,M **do** 16: Calculate the duplicate/kill rate: $R_{k+1}^{i}=-\lambda \eta \left(U_{{\tilde{\mu }}_{k}}\left(x_{k+1}^{i}\right)-\frac{1}{M}\sum_{i=1}^{M}U_{{\tilde{\mu }}_{k}}\left(x_{k+1}^{i}\right)\right)$ 17: **end for** 18: **for** i=1,2,…,M **do** 19: **if** $R_{k+1}^{i}>0$ **then** 20: Duplicate the particle $x_{k+1}^{i}$ with probability $1-\exp(-R_{k+1}^{i})$ and kill one which is uniformly chosen from the rest. 21: **else** 22: Kill the particle $x_{k+1}^{i}$ with probability $1-\exp(R_{k+1}^{i})$ and duplicate one which is uniformly chosen from the rest. 23: **end if** 24: **end for** 25: **end if** 26: **end for** 27: **Output**: ${\tilde{\mu }}_{T}=\frac{1}{M}\sum_{i=1}^{M}\delta_{x_{T}^{i}}$.

## Appendix C. Experiment Details

In this section, we list the details of the experimental setting, parameter tuning, and additional results of our empirical studies.

### Appendix C.1. Experiments Settings

#### Appendix C.1.1. Density of the Gaussian Mixture Model

The density of the Gaussian mixture model is defined as follows:

$$\begin{array}{c} \pi \left(x\right)\propto \frac{2}{3}\exp\left(-\frac{1}{2}{∥x-a∥}^{2}\right)+\frac{1}{3}\exp\left(-\frac{1}{2}{∥x+a∥}^{2}\right), \end{array}$$

where a=1.2∗1.

#### Appendix C.1.2. Density of the Gaussian Process Task

We follow the experimental setting in [23,66] and use the dataset LIDAR (denoted as $D={\left\{\left(x_{i},y_{i}\right)\right\}}_{i=1}^{N}$), which consists of 221 observations of the scalar variables $x_{i}$ and $y_{i}$. Denoting $x={\left[x_{1},x_{2},\ldots ,x_{N}\right]}^{T}$ and $y={\left[y_{1},y_{2},...,y_{N}\right]}^{T}$, the target log-posterior with respect to the model parameter $\phi =(\phi_{1},\phi_{2})$ is defined as follows:

$$\begin{array}{c} \log p(\phi |D)\;=\;-\frac{y^{T}K_{y}^{-1}y}{2}\;-\;\frac{\log\det\left(K_{y}\right)}{2}\;-\;\log\left(1\;+\;x^{T}x\right). \end{array}$$

Here, $K_{y}$ is a covariance function $K_{y}=K+0.04I$ with $K_{i,j}=\exp\left(\phi_{1}\right)\exp\left(-\exp\left(\phi_{2}\right){\left(x_{i}-x_{j}\right)}^{2}\right)$, and I represents the identity matrix.

#### Appendix C.1.3. Training/Validation/Test Dataset in Bayesian Neural Network

For each dataset in the Bayesian neural network task, we split it into 90% training data and 10% test data randomly, which follows the settings from [20,23,76]. Furthermore, we also randomly choose 1/5 of the training set as the validation set for parameter tuning.

#### Appendix C.1.4. Initialization of Particles’ Positions

In the Gaussian mixture model, we initialize particles according to the standard Gaussian distribution. In the Gaussian Process regression task, we initialize particles with mean vector ${\left[0,-10\right]}^{T}$ and covariance $0.09*I_{2\;\times \;2}$ for all the algorithms. As for the Bayesian neural network task, we follow the initialization convention in [20,23].

#### Appendix C.1.5. Bandwidth of Kernel Function in Different Algorithms

For all the experiments, we adopt RBF kernel as the kernel function *K*: $K\left(x,y\right)=\exp(-∥x-y{∥}_{2}^{2}/h)$, where the parameter *h* is known as the bandwidth [27]. We follow the convention in [23] and set the parameter $h=\frac{1}{M}\sum_{i=1}^{M}\left(\min_{j\neq i}∥x^{i}-x^{j}{∥}_{2}^{2}\right)$ for GFSD-type algorithms and BLOB-type algorithms.

#### Appendix C.1.6. WNES and WAG

Ref. [27] follows the accelerated gradient descend methods in the Wasserstein probability space [34,35] and derives the WNES and WAG methods, which update the particles’ positions with an extra momentum. Though their methods have the WNES and WAG types, we only conducted empirical studies of WNES as a baseline because the authors report WNES algorithms are usually more robust and efficient than WAG-type algorithms [27].

### Appendix C.2. Parameters Tuning

#### Detailed Settings for *η_pos_*, *η_wei_*, *η_vel_* and *γ*

Here, we present the parameter settings for position adjusting step-size $\eta_{pos}$, weight adjusting step-size $\eta_{wei}$, velocity field adjusting step-size $\eta_{vel}$, velocity damping parameter γ of different algorithms are provided in Table A1, Table A2, Table A3 and Table A4. All the parameters are chosen by a grid search. For the position adjusting step-size $\eta_{pos}$, we first find a suitable range using a coarse-grain grid search and then fine-tune it. Note that the position step size is tuned via grid search for the fixed-weight ParVI algorithms and then used in the corresponding dynamic-weight algorithms. The acceleration parameters and weight adjustment parameters are tuned via a grid search for each specific algorithm. As a result, it can be observed that the position adjusting step-size for any specific fixed-weight ParVI algorithm, its corresponding dynamic algorithm, and the DK variant are the same in these tables. For ease of understanding, we use the rate of weight, adjusting step-size $\eta_{wei}$ divided by the position, adjusting step-size $\eta_{pos}$ to illustrate the tuning. Moreover, inspired by the effective warmup strategy in tuning hyper-parameters, we follow the settings of [23] and construct the weight-adjusting step-size parameter schedule using the hyperbolic tangent $\lambda \tanh\left(2*{\left(t/T\right)}^{5}\right)$, with *t* being the current time step and *T* the total number of steps. This parameter tuning routine demonstrates the robustness of our GAD-PVI method with respect to hyperparameters. This is attributed to the fact that GAD-PVI can maintain stable performance when utilizing the optimal hyperparameters established for the baseline.

**Table A1:** Parameters of different algorithms in SG and GMM ($\eta_{pos}$, $\frac{\eta_{wei}}{\eta_{pos}}$, $\eta_{vel}$, and γ).

Algorithm	Tasks	
	Single Gaussian	Gaussian Mixture Model
ParVI-BLOB	$1.0\times 10^{-2}$, –, –, –	$1.0\times 10^{-2}$, –, –, –
WAIG-BLOB	$1.0\times 10^{-2}$, –, 1.0, 0.3	$1.0\times 10^{-2}$, –, 1.0, 0.3
WNES-BLOB	$1.0\times 10^{-2}$, –, 1.0, 0.2	$1.0\times 10^{-2}$, –, 1.0, 0.2
DPVI-CA-BLOB	$1.0\times 10^{-2}$, 1.0, –, –	$1.0\times 10^{-2}$, 1.0, –, –
DPVI-DK-BLOB	$1.0\times 10^{-2}$, 1.0, –, –	$1.0\times 10^{-2}$, 1.0, –, –
WGAD-CA-BLOB	$1.0\times 10^{-2}$, 1.0, 1.0, 0.3	$1.0\times 10^{-2}$, 1.0, 1.0, 0.3
WGAD-DK-BLOB	$1.0\times 10^{-2}$, $5\times 10^{-2}$, 1.0, 0.3	$1.0\times 10^{-2}$, $5\times 10^{-2}$, 1.0, 0.3
KWAIG-BLOB	$1.0\times 10^{-2}$, –, –, –	$1.0\times 10^{-2}$, –, –, –
KWGAD-CA-BLOB	$1.0\times 10^{-2}$, $5\times 10^{-3}$, 1.0, 0.9	$1.0\times 10^{-2}$, $5\times 10^{-3}$, 1.0, 0.9
KWGAD-DK-BLOB	$1.0\times 10^{-2}$, $5\times 10^{-2}$, 1.0, 0.9	$1.0\times 10^{-2}$, $5\times 10^{-2}$, 1.0, 0.9
SAIG-BLOB	$5.0\times 10^{-2}$, –, –, –	$2.5\times 10^{-2}$, –, –, –
SGAD-CA-BLOB	$5.0\times 10^{-2}$, $5\times 10^{-3}$, 1.0, 0.9	$2.5\times 10^{-2}$, $5\times 10^{-3}$, 1.0, 0.9
SGAD-DK-BLOB	$5.0\times 10^{-2}$, $5\times 10^{-2}$, 1.0, 0.9	$2.5\times 10^{-2}$, $5\times 10^{-2}$, 1.0, 0.9
ParVI-GFSD	$1.0\times 10^{-2}$, –, –, –	$1.0\times 10^{-2}$, –, –, –
WAIG-GFSD	$1.0\times 10^{-2}$, –, 1.0, 0.3	$1.0\times 10^{-2}$, –, 1.0, 0.3
WNES-GFSD	$1.0\times 10^{-2}$, –, 1.0, 0.2	$1.0\times 10^{-2}$, –, 1.0, 0.2
DPVI-CA-GFSD	$1.0\times 10^{-2}$, 1.0, –, –	$1.0\times 10^{-2}$, 0.8, –, –
DPVI-DK-GFSD	$1.0\times 10^{-2}$, 1.0, –, –	$1.0\times 10^{-2}$, 1.0, –, –
WGAD-CA-GFSD	$1.0\times 10^{-2}$, 1.0, 1.0, 0.3	$1.0\times 10^{-2}$, 0.8, 1.0, 0.3
WGAD-DK-GFSD	$1.0\times 10^{-2}$, $5\times 10^{-2}$, 1.0, 0.3	$1.0\times 10^{-2}$, $5\times 10^{-2}$, 1.0, 0.3
KWAIG-GFSD	$1.0\times 10^{-2}$, –, –, –	$1.0\times 10^{-2}$, –, –, –
KWGAD-CA-GFSD	$1.0\times 10^{-2}$, $5\times 10^{-3}$, 1.0, 0.9	$1.0\times 10^{-2}$, $5\times 10^{-3}$, 1.0, 0.9
KWGAD-DK-GFSD	$1.0\times 10^{-2}$, $5\times 10^{-2}$, 1.0,0.9	$1.0\times 10^{-2}$, $5\times 10^{-2}$, 1.0, 0.9
SAIG-GFSD	$5.0\times 10^{-2}$, –, –, –	$2.5\times 10^{-2}$, –, –, –
SGAD-CA-GFSD	$5.0\times 10^{-2}$, $5\times 10^{-3}$, 1.0, 0.9	$2.5\times 10^{-2}$, $5\times 10^{-3}$, 1.0, 0.9
SGAD-DK-GFSD	$5.0\times 10^{-2}$, $5\times 10^{-2}$, 1.0, 0.9	$2.5\times 10^{-2}$, $5\times 10^{-2}$, 1.0, 0.9

**Table A2:** Parameters of different algorithms in GP ($\eta_{pos}$, $\frac{\eta_{wei}}{\eta_{pos}}$, $\eta_{vel}$ and γ).

Algorithm	Smoothing Approaches	
	BLOB	GFSD
ParVI	$1.0\times 10^{-2}$, –, –, –	$1.0\times 10^{-2}$, –, –, –
WAIG	$1.0\times 10^{-2}$, –, 1.0, 0.4	$1.0\times 10^{-2}$, –, 1.0, 0.3
WNES	$1.0\times 10^{-2}$, –, 1.0, 0.4	$1.0\times 10^{-2}$, –, 1.0, 0.4
DPVI-CA	$1.0\times 10^{-2}$, 0.1, –, –	$1.0\times 10^{-2}$, 0.3, –, –
DPVI-DK	$1.0\times 10^{-2}$, 0.01, –, –	$1.0\times 10^{-2}$, 0.01, –, –
WGAD-CA	$1.0\times 10^{-2}$, 0.1, 1.0, 0.4	$1.0\times 10^{-2}$, 0.3, 1.0, 0.3
WGAD-DK	$1.0\times 10^{-2}$, 0.01, 1.0, 0.4	$1.0\times 10^{-2}$, 0.01, 1.0, 0.3
KWAIG	$5.0\times 10^{-3}$, –, 1.0, 0.8	$1.0\times 10^{-3}$, –, 1.0, 0.7
KWGAD-CA	$5.0\times 10^{-3}$, 0.1, 1.0, 0.8	$1.0\times 10^{-3}$, 0.3, 1.0, 0.7
KWGAD-DK	$5.0\times 10^{-3}$, 0.01, 1.0, 0.8	$1.0\times 10^{-3}$, 0.01, 1.0, 0.7
SAIG	$2.0\times 10^{-2}$, –, 1.0, 0.7	$1.0\times 10^{-2}$, –, 1.0, 0.6
SGAD-CA	$2.0\times 10^{-2}$, 0.1, 1.0, 0.7	$1.0\times 10^{-2}$, 0.3, 1.0, 0.6
SGAD-DK	$2.0\times 10^{-2}$, 0.01, 1.0, 0.7	$1.0\times 10^{-2}$, 0.01, 1.0, 0.6

**Table A3:** Parameters of different algorithms in BNN ($\eta_{pos}$, $\frac{\eta_{wei}}{\eta_{pos}}$, $\eta_{vel}$ and γ).

Algorithm	Datasets			
	Concrete	kin8nm	RedWine	Space
ParVI-BLOB	$4.0\times 10^{-6}$, –, –, –	$1.0\times 10^{-6}$, –, –, –	$3.4\times 10^{-6}$, –, –, –	$3.0\times 10^{-6}$, –, –, –
WAIG-BLOB	$4.0\times 10^{-6}$, –, 1.0, 0.2	$1.0\times 10^{-6}$, –, 1.0, 0.3	$3.4\times 10^{-6}$, –, 1.0, 0.5	$3.0\times 10^{-6}$, –, 1.0, 0.5
WNES-BLOB	$4.0\times 10^{-6}$, –, 1.0, 0.3	$1.0\times 10^{-6}$, –, 1.0, 0.2	$3.4\times 10^{-6}$, –, 1.0, 0.2	$3.0\times 10^{-6}$, –, 1.0, 0.2
DPVI-CA-BLOB	$4.0\times 10^{-6}$, 1.0, –, –	$1.0\times 10^{-6}$, 0.8, –, –	$3.4\times 10^{-6}$, 0.5, –, –	$3.0\times 10^{-6}$, 1.0, –, –
DPVI-DK-BLOB	$4.0\times 10^{-6}$, 1.0, –, –	$1.0\times 10^{-6}$, 0.8, –, –	$3.4\times 10^{-6}$, 0.5, –, –	$3.0\times 10^{-6}$, 1.0, –, –
WGAD-CA-BLOB	$4.0\times 10^{-6}$, 1.0, 1.0, 0.2	$1.0\times 10^{-6}$, 0.8, 1.0, 0.3	$3.4\times 10^{-6}$, 0.5, 1.0, 0.5	$3.0\times 10^{-6}$, 1.0, 1.0, 0.5
WGAD-DK-BLOB	$4.0\times 10^{-6}$, 1.0, 1.0, 0.2	$1.0\times 10^{-6}$, 0.8, 1.0, 0.3	$3.4\times 10^{-6}$, 0.1, 1.0, 0.5	$3.0\times 10^{-6}$, 1.0, 1.0, 0.5
KWAIG-BLOB	$4.0\times 10^{-6}$, –, 1.0, 0.7	$1.0\times 10^{-6}$, –, 1.0, 0.3	$3.4\times 10^{-6}$, –, 1.0, 0.5	$2.0\times 10^{-6}$, –, 1.0, 0.8
KWGAD-CA-BLOB	$4.0\times 10^{-6}$, 1.0, 1.0, 0.7	$1.0\times 10^{-6}$, 0.8, 1.0, 0.3	$3.4\times 10^{-6}$, 0.5, 1.0, 0.5	$2.0\times 10^{-6}$, 1.0, 1.0, 0.8
KWGAD-DK-BLOB	$4.0\times 10^{-6}$, 1.0, 1.0, 0.7	$1.0\times 10^{-6}$, 0.8, 1.0, 0.3	$3.4\times 10^{-6}$, 0.1, 1.0, 0.5	$2.0\times 10^{-6}$, 1.0, 1.0, 0.8
SAIG-BLOB	$1.0\times 10^{-5}$, –, 1.0, 0.6	$2.0\times 10^{-6}$, –, 1.0, 0.3	$7.0\times 10^{-6}$, –, 1.0, 0.5	$8.0\times 10^{-6}$, –, 1.0, 0.8
SGAD-CA-BLOB	$1.0\times 10^{-5}$, 1.0, 1.0, 0.6	$2.0\times 10^{-6}$, 0.8, 1.0, 0.3	$7.0\times 10^{-6}$, 0.5, 1.0, 0.5	$8.0\times 10^{-6}$, 1.0, 1.0, 0.8
SGAD-DK-BLOB	$1.0\times 10^{-5}$, 1.0, 1.0, 0.6	$2.0\times 10^{-6}$, 0.8, 1.0, 0.3	$7.0\times 10^{-6}$, 0.1, 1.0, 0.5	$8.0\times 10^{-6}$, 1.0, 1.0, 0.8
ParVI-GFSD	$4.0\times 10^{-6}$, –, –, –	$1.0\times 10^{-6}$, –, –, –	$3.4\times 10^{-6}$, –, –, –	$3.0\times 10^{-6}$, –, –, –
WAIG-GFSD	$4.0\times 10^{-6}$, –, 1.0, 0.1	$1.0\times 10^{-6}$, –, 1.0, 0.3	$3.4\times 10^{-6}$, –, 1.0, 0.5	$3.0\times 10^{-6}$, –, 1.0, 0.5
WNES-GFSD	$4.0\times 10^{-6}$, –, 1.0, 0.3	$1.0\times 10^{-6}$, –, 1.0, 0.3	$3.4\times 10^{-6}$, –, 1.0, 0.2	$3.0\times 10^{-6}$, –, 1.0, 0.2
DPVI-CA-GFSD	$4.0\times 10^{-6}$, 1.0, –, –	$1.0\times 10^{-6}$, 0.8, –, –	$3.4\times 10^{-6}$, 0.5, –, –	$3.0\times 10^{-6}$, 1.0, –, –
DPVI-DK-GFSD	$4.0\times 10^{-6}$, 1.0, –, –	$1.0\times 10^{-6}$, 0.8, –, –	$3.4\times 10^{-6}$, 0.5, –, –	$3.0\times 10^{-6}$, 1.0, –, –
WGAD-CA-GFSD	$4.0\times 10^{-6}$, 1.0, 1.0, 0.1	$1.0\times 10^{-6}$, 0.8, 1.0, 0.3	$3.4\times 10^{-6}$, 0.5, 1.0, 0.5	$3.0\times 10^{-6}$, 1.0, 1.0, 0.5
WGAD-DK-GFSD	$4.0\times 10^{-6}$, 1.0, 1.0, 0.1	$1.0\times 10^{-6}$, 0.8, 1.0, 0.3	$3.4\times 10^{-6}$, 0.1, 1.0, 0.5	$3.0\times 10^{-6}$, 1.0, 1.0, 0.5
KWAIG-GFSD	$4.0\times 10^{-6}$, –, 1.0, 0.6	$1.0\times 10^{-6}$, –, 1.0, 0.3	$3.4\times 10^{-6}$, –, 1.0, 0.5	$3.0\times 10^{-6}$, –, 1.0, 0.3
KWGAD-CA-GFSD	$4.0\times 10^{-6}$, 1.0, 1.0, 0.6	$1.0\times 10^{-6}$, 0.8, 1.0, 0.3	$3.4\times 10^{-6}$, 0.5, 1.0, 0.5	$3.0\times 10^{-6}$, 1.0, 1.0, 0.3
KWGAD-DK-GFSD	$4.0\times 10^{-6}$, 1.0, 1.0, 0.6	$1.0\times 10^{-6}$, 0.8, 1.0, 0.3	$3.4\times 10^{-6}$, 0.1, 1.0, 0.5	$3.0\times 10^{-6}$, 1.0, 1.0, 0.3
SAIG-GFSD	$4.0\times 10^{-6}$, –, 1.0, 0.2	$2.0\times 10^{-6}$, –, 1.0, 0.3	$7.0\times 10^{-6}$, –, 1.0, 0.5	$6.0\times 10^{-6}$, –, 1.0, 0.3
SGAD-CA-GFSD	$4.0\times 10^{-6}$, 1.0, 1.0, 0.2	$2.0\times 10^{-6}$, 0.8, 1.0, 0.3	$7.0\times 10^{-6}$, 0.5, 1.0, 0.5	$6.0\times 10^{-6}$, 1.0, 1.0, 0.3
SGAD-DK-GFSD	$4.0\times 10^{-6}$, 1.0, 1.0, 0.2	$2.0\times 10^{-6}$, 0.8, 1.0, 0.3	$7.0\times 10^{-6}$, 0.1, 1.0, 0.5	$6.0\times 10^{-6}$, 1.0, 1.0, 0.3

**Table A4:** Parameters of different algorithms in morphing and sketching ($\eta_{pos}$, $\frac{\eta_{wei}}{\eta_{pos}}$, $\eta_{vel}$ and γ).

Algorithms	Sampling Tasks				
	A→B	B→C	C→D	D→A	Sketching
ParVI-MMDF	$3.0\;\times \;10^{-2}$, –, –, –	$3.0\;\times \;10^{-2}$, –, –, –	$3.0\;\times \;10^{-2}$, –, –, –	$3.0\;\times \;10^{-2}$, –, –, –	$3.0\;\times \;10^{-2}$, –, –, –
WAIG-MMDF	$3.0\;\times \;10^{-2}$, –, 1.0, 0.8	$3.0\;\times \;10^{-2}$, –, 1.0, 0.8	$3.0\;\times \;10^{-2}$, –, 1.0, 0.8	$3.0\;\times \;10^{-2}$, –, 1.0, 0.8	$3.0\;\times \;10^{-2}$, –, 1.0, 0.8
DPVI-DK-MMDF	$3.0\;\times \;10^{-2}$, $5.0\;\times \;10^{-1}$, –, –	$3.0\;\times \;10^{-2}$, $5.0\;\times \;10^{-1}$, –, –	$3.0\;\times \;10^{-2}$, $5.0\;\times \;10^{-1}$, –, –	$3.0\;\times \;10^{-2}$, $5.0\;\times \;10^{-1}$, –, –	$3.0\;\times \;10^{-2}$, $5.0\;\times \;10^{-1}$, –, –
DPVI-CA-MMDF	$3.0\;\times \;10^{-2}$, $1.0\;\times \;10^{1}$, –, –	$3.0\;\times \;10^{-2}$, $1.0\;\times \;10^{1}$, –, –	$3.0\;\times \;10^{-2}$, $1.0\;\times \;10^{1}$, –, –	$3.0\;\times \;10^{-2}$, $1.0\;\times \;10^{1}$, –, –	$3.0\;\times \;10^{-2}$, $1.0\;\times \;10^{1}$, –, –
WGAD-DK-MMDF	$3.0\;\times \;10^{-2}$, $5.0\;\times \;10^{-1}$, 1.0, 1.0, 0.8	$3.0\;\times \;10^{-2}$, $5.0\;\times \;10^{-1}$, 1.0, 1.0, 0.8	$3.0\;\times \;10^{-2}$, $5.0\;\times \;10^{-1}$, 1.0, 1.0, 0.8	$3.0\;\times \;10^{-2}$, $5.0\;\times \;10^{-1}$, 1.0, 1.0, 0.8	$3.0\;\times \;10^{-2}$, $5.0\;\times \;10^{-1}$, 1.0, 1.0, 0.8
WGAD-CA-MMDF	$3.0\;\times \;10^{-2}$, $1.0\;\times \;10^{1}$, 1.0, 1.0, 0.8	$3.0\;\times \;10^{-2}$, $1.0\;\times \;10^{1}$, 1.0, 1.0, 0.8	$3.0\;\times \;10^{-2}$, $1.0\;\times \;10^{1}$, 1.0, 1.0, 0.8	$3.0\;\times \;10^{-2}$, $1.0\;\times \;10^{1}$, 1.0, 1.0, 0.8	$3.0\;\times \;10^{-2}$, $1.0\;\times \;10^{1}$, 1.0, 1.0, 0.8
ParVI-SDF	$1.0\;\times \;10^{-1}$, –, –, –	$1.0\;\times \;10^{-1}$, –, –, –	$1.0\;\times \;10^{-1}$, –, –, –	$1.0\;\times \;10^{-1}$, –, –, –	$1.0\;\times \;10^{-1}$, –, –, –
WAIG-SDF	$1.0\;\times \;10^{-1}$, –, 1.0, 0.8	$1.0\;\times \;10^{-1}$, –, 1.0, 0.8	$1.0\;\times \;10^{-1}$, –, 1.0, 0.8	$1.0\;\times \;10^{-1}$, –, 1.0, 0.8	$1.0\;\times \;10^{-1}$, –, 1.0, 0.8
DPVI-DK-SDF	$1.0\;\times \;10^{-1}$, $5.0\;\times \;10^{-1}$, –, –	$1.0\;\times \;10^{-1}$, $5.0\;\times \;10^{-1}$, –, –	$1.0\;\times \;10^{-1}$, $5.0\;\times \;10^{-1}$, –, –	$1.0\;\times \;10^{-1}$, $5.0\;\times \;10^{-1}$, –, –	$1.0\;\times \;10^{-1}$, $5.0\;\times \;10^{-1}$, –, –
DPVI-CA-SDF	$1.0\;\times \;10^{-1}$, $1.0\;\times \;10^{1}$, –, –	$1.0\;\times \;10^{-1}$, $1.0\;\times \;10^{1}$, –, –	$1.0\;\times \;10^{-1}$, $1.0\;\times \;10^{1}$, –, –	$1.0\;\times \;10^{-1}$, $1.0\;\times \;10^{1}$, –, –	$1.0\;\times \;10^{-1}$, $1.0\;\times \;10^{1}$, –, –
WGAD-DK-SDF	$1.0\;\times \;10^{-1}$, $5.0\;\times \;10^{-1}$, 1.0, 1.0, 0.8	$1.0\;\times \;10^{-1}$, $5.0\;\times \;10^{-1}$, 1.0, 1.0, 0.8	$1.0\;\times \;10^{-1}$, $5.0\;\times \;10^{-1}$, 1.0, 1.0, 0.8	$1.0\;\times \;10^{-1}$, $5.0\;\times \;10^{-1}$, 1.0, 1.0, 0.8	$1.0\;\times \;10^{-1}$, $5.0\;\times \;10^{-1}$, 1.0, 1.0, 0.8
WGAD-CA-SDF	$1.0\;\times \;10^{-1}$, $1.0\;\times \;10^{1}$, 1.0, 1.0, 0.8	$1.0\;\times \;10^{-1}$, $1.0\;\times \;10^{1}$, 1.0, 1.0, 0.8	$1.0\;\times \;10^{-1}$, $1.0\;\times \;10^{1}$, 1.0, 1.0, 0.8	$1.0\;\times \;10^{-1}$, $1.0\;\times \;10^{1}$, 1.0, 1.0, 0.8	$1.0\;\times \;10^{-1}$, $1.0\;\times \;10^{1}$, 1.0, 1.0, 0.8

### Appendix C.3. Additional Experiments Results

#### Appendix C.3.1. Results for SG

In this section, we give empirical results of approximating a single-mode Gaussian distribution, whose density is defined as

$$\begin{array}{c} \pi \left(x\right)\propto \exp\left(-\frac{1}{2}x^{T}\Sigma^{-1}x\right), \end{array}$$

where $\Sigma_{ii}=1.0$ and correlation $\Sigma_{ij,i\neq j}=0.8$. To investigate the influence of number *M* in this task, we run all the algorithms with M∈{32,64,128,256,512}. All the particles are initialized from a Gaussian distribution with zero mean and covariance matrix $0.5*I_{10\times 10}$.

In Figure A2 and Figure A3, we plot the $W_{2}$ distance to the target of the samples generated by each algorithm with respect to iteration and time. We generate 5000 samples from the target distribution π as a reference to evaluate $W_{2}$. As this task is a simple Single Gaussian model, the approximation error difference between the GAD-PVI algorithms and fixed-weight ones is not so obvious. When the particle number increases, the effect of the dynamic weight adjustment scheme is smaller, for such a large number of fixed-weight particles is also sufficient for approximating this simple distribution. Meanwhile, the faster convergence effect of the accelerated position update strategies is quite obvious in the figures. Moreover, we can see that the GFSD-type algorithms cannot outperform the BLOB-type algorithms and SVGD; this may be due to the lack of the repulsive mechanism in GFSD, which leads to particle system collapse in single-mode tasks. This also coincides with the discussion in Appendix B.3.

In Table A5, we further report the final $W_{2}$ distance between the empirical distribution generated by each algorithm and the target distribution. It can be observed that, in the Wasserstein metric case, the CA strategy consistently outperforms its fixed-weight counterparts with the same number of particles, and the DK variants are weakened due to the single modality of this task. However, in the KW or S metric case, it can be observed that the GAD-DK algorithms outperform others in the majority of cases, which is due to the poor transportation ability of the KW and S metrics. A direct duplicate/kill mechanism greatly enhances the transport speed from low-probability regions to high-probability regions. We also find that the KW- and S-type algorithms achieve poor approximation results compared to WGAD algorithms in terms of the Wasserstein distance to reference points, which may be because the WGAD algorithms aim to implicitly minimize the Wasserstein distance, and this is not the case for other two types.

**Table A5:** Averaged test $W_{2}$ distances for different ParVI methods in the SG task.

Algorithm	Number of Particles				
	32	64	128	256	512
ParVI-SVGD	1.320	1.228	1.162	1.102	1.027
ParVI-BLOB	1.315	1.229	1.164	1.102	1.038
WAIG-BLOB	1.314	1.230	1.164	1.100	1.038
WNes-BLOB	1.315	1.230	1.164	1.101	1.037
DPVI-DK-BLOB	1.313	1.229	1.163	1.100	1.035
DPVI-CA-BLOB	1.309	1.227	1.162	1.102	1.037
WGAD-DK-BLOB(Ours)	1.313	1.229	1.163	1.098	1.034
WGAD-CA-BLOB(Ours)	1.300	1.226	1.161	1.099	1.036
KWAIG-BLOB	1.943	1.922	1.889	1.827	1.777
KWGAD-DK-BLOB(Ours)	1.920	$1.884\times 10^{-2}$	1.848	1.798	1.757
KWGAD-CA-BLOB(Ours)	1.942	1.901	1.865	1.816	1.764
SAIG-BLOB	1.451	1.412	1.429	1.436	1.479
SGAD-DK-BLOB(Ours)	1.435	1.355	1.341	1.219	1.143
SGAD-CA-BLOB(Ours)	1.444	1.396	1.412	1.405	1.407
ParVI-GFSD	1.453	1.353	1.267	1.198	1.136
WAIG-GFSD	1.449	1.353	1.264	1.196	1.134
WNes-GFSD	1.450	1.353	1.265	1.197	1.135
DPVI-DK-GFSD	1.448	1.347	1.267	1.197	1.135
DPVI-CA-GFSD	1.446	1.349	1.259	1.195	1.133
WGAD-DK-GFSD(Ours)	1.448	1.345	1.264	1.195	1.134
WGAD-CA-GFSD(Ours)	1.398	1.332	1.252	1.191	1.131
KWAIG-GFSD	2.246	2.171	2.134	$2.082\times 10^{-2}$	2.046
KWGAD-DK-GFSD(Ours)	2.215	2.154	2.092	$2.073\times 10^{-2}$	2.022
KWGAD-CA-GFSD(Ours)	2.204	2.160	2.110	$2.081\times 10^{-2}$	2.035
SAIG-GFSD	1.823	1.789	1.760	$1.626\times 10^{-2}$	1.609
SGAD-DK-GFSD(Ours)	1.881	1.782	1.609	$1.437\times 10^{-2}$	1.401
SGAD-CA-GFSD(Ours)	1.820	1.783	1.720	$1.602\times 10^{-2}$	1.592

#### Appendix C.3.2. Additional Results for GMM

We provide additional results for the Gaussian Mixture Model experiment. In Figure 1 and Figure A4, we plot the $W_{2}$ w.r.t iteration of each algorithm for all M={32,63,128,256,512}. From these figures, we can observe that, compared with the baseline ParVI algorithms, our GAD-PVI of either CA strategy or DK variants results in a better performance, i.e., less approximation error and faster convergence. Actually, as we have discussed in the methodology part, while the weight-adjustment step in GAD-PVI greatly enhances the expressiveness of particles’ empirical distribution, the accelerated position update strategy also brings in faster convergence. From Figure 1, we find that DK variants decrease quite quickly at first in the WGAD case, which is because the duplicate/kill scheme will greatly enhance the particle transport ability, thus moving more particles to high-probability regions at first compared to moving particles step by step. In Figure A4, we observe that DK variants also show better results compared to other algorithms, which may be because of the slow transportation property of KW and S-type algorithms.

In Table A6, we further report the final $W_{2}$ distance between the empirical distribution generated by each algorithm and the target distribution. It can be observed that GAD-PVI algorithms constantly achieve better approximation results than existing algorithms. Notably, for this complex multi-mode task, DK variants show their advantage as the duplicate/kill operation allows transferring particles from a low-probability region to a distant high-probability area (e.g., among different local modes), especially in the KW/S case. However, the DK variants in the Wasserstein case are not as competitive with CA algorithms; this difference could lie in that the KW/S metric space is much more influenced by the potential barrier, which needs the DK scheme to transport particles among different local modes, but the Wasserstein metric is more robust from the sufficient multi-modality and CA strategy. Furthermore, the GAD-PVI algorithms with the CA strategy are usually more stable than their counterpart with DK, which may be ascribed to the fluctuations induced by the discrete weight adjustment (0 or 1/M) in DK.

**Table A6:** Averaged test $W_{2}$ distances for different ParVI methods in GMM tasks.

Algorithm	Number of Particles				
	32	64	128	256	512
ParVI-SVGD	2.175	2.101	2.088	2.026	2.044
ParVI-BLOB	2.317	2.779	2.292	2.440	2.294
WAIG-BLOB	2.317	2.775	2.039	1.976	1.845
WNes-BLOB	2.317	2.777	2.213	2.329	2.180
DPVI-DK-BLOB	2.065	2.066	1.859	1.735	1.704
DPVI-CA-BLOB	2.039	1.934	1.825	1.727	1.633
WGAD-DK-BLOB(Ours)	2.064	1.933	1.831	1.727	1.650
WGAD-CA-BLOB(Ours)	2.037	1.929	1.824	1.725	1.632
KWAIG-BLOB	4.937	4.674	4.600	4.242	4.221
KWGAD-DK-BLOB(Ours)	2.854	2.622	2.400	2.542	2.248
KWGAD-CA-BLOB(Ours)	4.565	4.206	4.094	3.836	3.767
SAIG-BLOB	5.070	4.632	4.554	4.140	4.032
SGAD-DK-BLOB(Ours)	2.863	2.914	2.760	1.1890	2.247
SGAD-CA-BLOB(Ours)	3.406	3.051	2.659	2.595	2.492
ParVI-GFSD	2.427	2.888	2.331	2.567	2.398
WAIG-GFSD	2.425	2.885	2.328	2.206	2.094
WNes-GFSD	2.426	2.888	2.330	2.494	2.337
DPVI-DK-GFSD	2.151	2.025	1.924	1.837	1.769
DPVI-CA-GFSD	2.134	2.025	1.928	1.838	1.755
WGAD-DK-GFSD(Ours)	2.150	2.017	1.924	1.834	1.755
WGAD-CA-GFSD(Ours)	2.120	2.019	1.923	1.835	1.754
KWAIG-GFSD	5.072	4.780	4.706	4.381	4.359
KWGAD-DK-GFSD(Ours)	3.086	2.682	2.817	2.385	2.393
KWGAD-CA-GFSD(Ours)	4.597	4.389	4.262	3.960	3.929
SAIG-GFSD	4.828	4.577	4.555	4.151	4.075
SGAD-DK-GFSD(Ours)	2.994	3.411	2.881	2.347	2.324
SGAD-CA-GFSD(Ours)	3.937	4.142	3.676	3.617	4.007

#### Appendix C.3.3. Additional Results for GP

Here, we provide additional results of the KW/S-type method for Gaussian Process Regression task in Table A7. The result is quite similar to the Wasserstein case, i.e., both the accelerated position update and the dynamic weight adjustment result in a decreased $W_{2}$ and GAD-PVI algorithms to consistently achieve loweset $W_{2}$ to the target. Note that the difference between DK type GAD-PVI and their fixed weight counterpart is not that obvious due to the fact that the one-mode nature of GP greatly weakens the advantage of DK, i.e., transferring particles from low-probability region to distant high-probability area (e.g., among different local modes).

**Table A7:** Averaged $W_{2}$ distances after 10,000 iterations with different KW/S-type algorithms in the GP task with dataset LIDAR.

Algorithm	Smoothing Strategy	
	BLOB	GFSD
KWAIG	$1.571\times 10^{-1}\pm 2.190\times 10^{-4}$	$2.146\times 10^{-1}\pm 8.608\times 10^{-4}$
GAD-KW-DK	$1.566\times 10^{-1}\pm 1.791\times 10^{-3}$	$2.072\times 10^{-1}\pm 1.772\times 10^{-3}$
GAD-KW-CA	$1.341\times 10^{-1}\pm 1.494\times 10^{-4}$	$1.991\times 10^{-1}\pm 4.415\times 10^{-4}$
SAIG	$1.570\times 10^{-1}\pm 3.791\times 10^{-4}$	$2.084\times 10^{-1}\pm 7.575\times 10^{-3}$
GAD-S-DK	$1.552\times 10^{-1}\pm 1.090\times 10^{-2}$	$2.012\times 10^{-1}\pm 4.960\times 10^{-3}$
GAD-S-CA	$1.236\times 10^{-1}\pm 2.872\times 10^{-4}$	$1.691\times 10^{-1}\pm 3.499\times 10^{-3}$

#### Appendix C.3.4. Additional Results for BNN

We provide additional test negative log-likelihood results for the Bayesian neural network experiment for all algorithms in Table A8 and the test RMSE result under KW/S-Type algorithms in Table A9. The results demonstrate that the combination of the accelerated position updating strategy and the dynamically weighted adjustment leads to a lower NLL and RMSE under difference-specific IFR space, and WGAD-PVI algorithms with CA usually achieve the best performance in the Wasserstein case, while KW/SGAD-PVI algorithms with CA or DK are comparable to each other. Note that the position step-size of GAD-PVI is set to the value tuned for their fixed weight counterpart. Actually, if we retune the position step size for all GAD-PVI algorithms, they are expected to achieve a better performance than the existing result.

**Table A8:** Averaged test NLL distances for different ParVI methods in the BNN task.

Algorithm	Datasets			
	Concrete	kin8nm	RedWine	Space
ParVI-SVGD	1.738	1.160	6.943	2.739
ParVI-BLOB	1.849	1.122	6.900	2.742
WAIG-BLOB	1.710	1.093	6.790	2.638
WNES-BLOB	1.734	1.065	6.799	2.679
DPVI-DK-BLOB	1.833	1.122	6.848	2.687
DPVI-CA-BLOB	1.837	1.093	6.856	2.685
WGAD-DK-BLOB(Ours)	1.703	1.065	6.785	2.602
WGAD-CA-BLOB(Ours)	1.697	1.048	6.782	2.595
KWAIG-BLOB	1.794	1.199	$9.322\;\times \;10^{-1}$	2.751
KWGAD-DK-BLOB(Ours)	1.788	1.169	$9.300\;\times \;10^{-1}$	2.743
KWGAD-CA-BLOB(Ours)	1.789	1.173	$9.304\;\times \;10^{-1}$	2.736
SAIG-BLOB	1.832	1.136	$9.349\;\times \;10^{-1}$	2.711
SGAD-DK-BLOB(Ours)	1.821	1.068	$9.292\;\times \;10^{-1}$	2.708
SGAD-CA-BLOB(Ours)	1.824	1.119	$9.270\;\times \;10^{-1}$	2.642
ParVI-GFSD	1.850	1.122	6.899	2.742
WAIG-GFSD	1.724	1.094	6.785	2.638
WNES-GFSD	1.740	1.108	6.781	2.679
DPVI-DK-GFSD	1.836	1.120	6.812	2.687
DPVI-CA-GFSD	1.836	1.093	6.857	2.687
WGAD-DK-GFSD(Ours)	1.722	1.075	6.780	2.593
WGAD-CA-GFSD(Ours)	1.720	1.050	6.774	2.597
KWAIG-GFSD	1.820	1.199	$9.337\;\times \;10^{-1}$	2.759
KWGAD-DK-GFSD(Ours)	1.813	1.176	$9.305\;\times \;10^{-1}$	2.740
KWGAD-CA-GFSD(Ours)	1.812	1.169	$9.319\;\times \;10^{-1}$	2.746
SAIG-GFSD	1.814	1.116	$9.444\;\times \;10^{-1}$	2.782
SGAD-DK-GFSD(Ours)	1.809	1.062	$9.391\;\times \;10^{-1}$	2.555
SGAD-CA-GFSD(Ours)	1.800	1.098	$9.359\;\times \;10^{-1}$	2.745

**Table A9:** Averaged test RMSE for different ParVI methods in the BNN task.

Algorithm	Datasets			
	Concrete	kin8nm	RedWine	Space
KWAIG-BLOB	6.217	$8.159\;\times \;10^{-2}$	6.916	$8.829\;\times \;10^{-2}$
KWGAD-DK-BLOB(Ours)	6.207	$8.069\;\times \;10^{-2}$	6.910	$8.760\;\times \;10^{-2}$
KWGAD-CA-BLOB(Ours)	6.208	$8.058\;\times \;10^{-2}$	6.896	$8.753\;\times \;10^{-2}$
SAIG-BLOB	6.323	$7.936\;\times \;10^{-2}$	6.856	$8.899\;\times \;10^{-2}$
SGAD-DK-BLOB(Ours)	6.293	$7.695\;\times \;10^{-2}$	6.828	$8.867\;\times \;10^{-2}$
SGAD-CA-BLOB(Ours)	6.269	$7.872\;\times \;10^{-2}$	6.811	$8.783\;\times \;10^{-2}$
KWAIG-GFSD	6.330	$8.158\;\times \;10^{-2}$	6.924	$8.948\;\times \;10^{-2}$
KWGAD-DK-GFSD(Ours)	6.274	$8.079\;\times \;10^{-2}$	6.854	$8.918\;\times \;10^{-2}$
KWGAD-CA-GFSD(Ours)	6.251	$8.057\;\times \;10^{-2}$	6.917	$8.927\;\times \;10^{-2}$
SAIG-GFSD	6.266	$7.864\;\times \;10^{-2}$	6.861	$8.998\;\times \;10^{-2}$
SGAD-DK-GFSD(Ours)	6.257	$7.679\;\times \;10^{-2}$	6.804	$8.638\;\times \;10^{-2}$
SGAD-CA-GFSD(Ours)	6.228	$7.801\;\times \;10^{-2}$	6.793	$8.931\;\times \;10^{-2}$

#### Appendix C.3.5. Results for Morphing

We provide additional morphing results in Figure A1, which shows the morphing process from CAT to SPIRAL.

#### Appendix C.3.6. Results for GAD-KSDD

Newly derived KSDD methods proposed by [64] evolve particle systems according to the direct minimizing of the Kernel Stein Discrepancy (KSD) of particles with respect to the target distribution. The KSDD method is the first ParVI that introduces the dissimilarity functional. whose first variation is well defined at a discrete empirical distribution, thus resulting in no approximation error when the particle number is infinite. Though theoretically impressive, the experimental performance of KSDD is not satisfying, for they are more computationally expensive and have been widely reported to be less stable. Furthermore, KSDD is also reported to make particles easily trapped at saddle points and demanding for the convexity of task and the sensitivity to parameters.

As shown in Figure A5, we make simple Wasserstein experiments of KSDD-type algorithms on SG task to illustrate that our GAD-PVI framework is compatible with the KSD-KSDD approach. The bandwidth of KSDD is reported and should be carefully determined [23,64]; we follow the conventions in [28,51] and set the parameter *h* via a grid search. From Figure A5, it can be observed that our GAD-PVI algorithms achieve the best results in different particle number settings. These illustrate our framework can cooperate with this new smoothing approach. However, due to the limit of the KSDD itself, it is not realistic to fine-tune parameters and conduct empirical studies of KSDD ParVI algorithms on complex tasks. Additionally, in this simple SG task, the final result of KSDD-type algorithms is not competitive with other methods at all. So we exclude KSDD-type experiments in GMM, GP, and BNN.
