# Supplementary figures and images for: GAD-PVI: A General Accelerated Dynamic-Weight Particle-Based Variational Inference Framework (part 2 of 2)
Source: Entropy (Basel). 2024 Aug 11;26(8):679. doi: 10.3390/e26080679 (PMC11354113; doi:10.3390/e26080679)

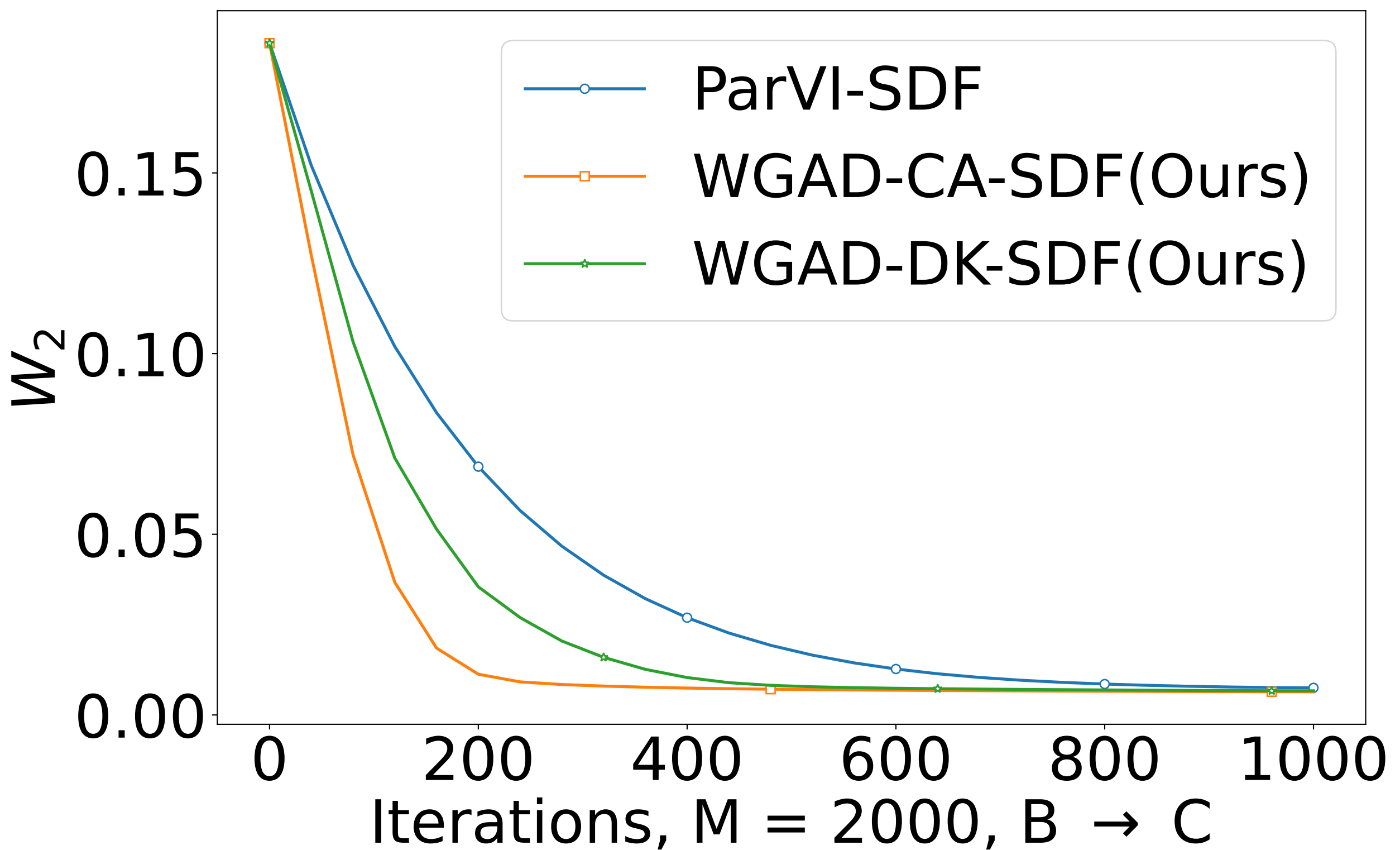

Supplement: Supplementary file 1 [file entropy-26-00679-s001.zip › dpvi_discrete-master/figures_morphing/5_figures_big/SD_w2BC.pdf]

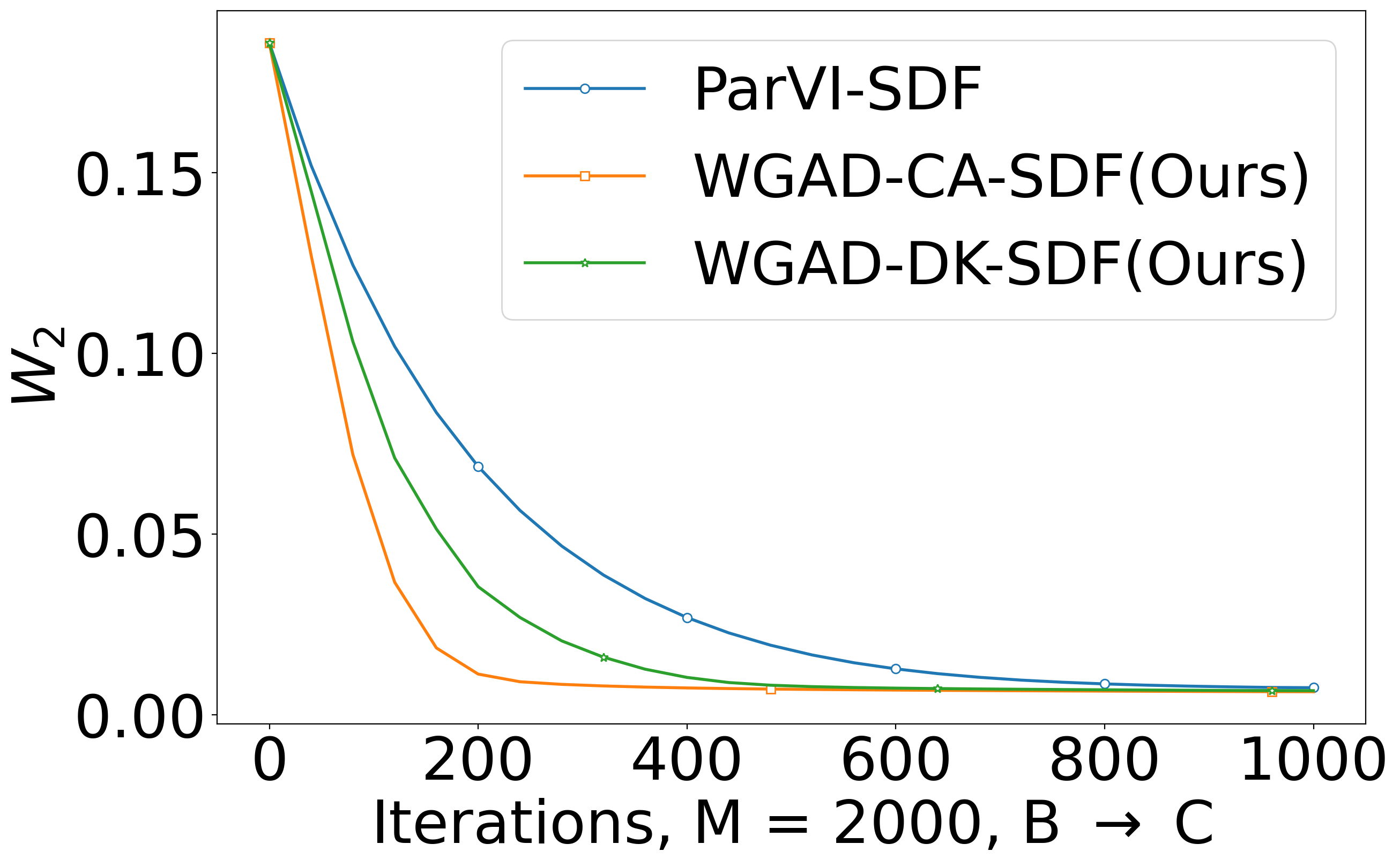

Supplement: Supplementary file 1 [file entropy-26-00679-s001.zip › dpvi_discrete-master/figures_morphing/5_figures_big/SD_w2BC.png]

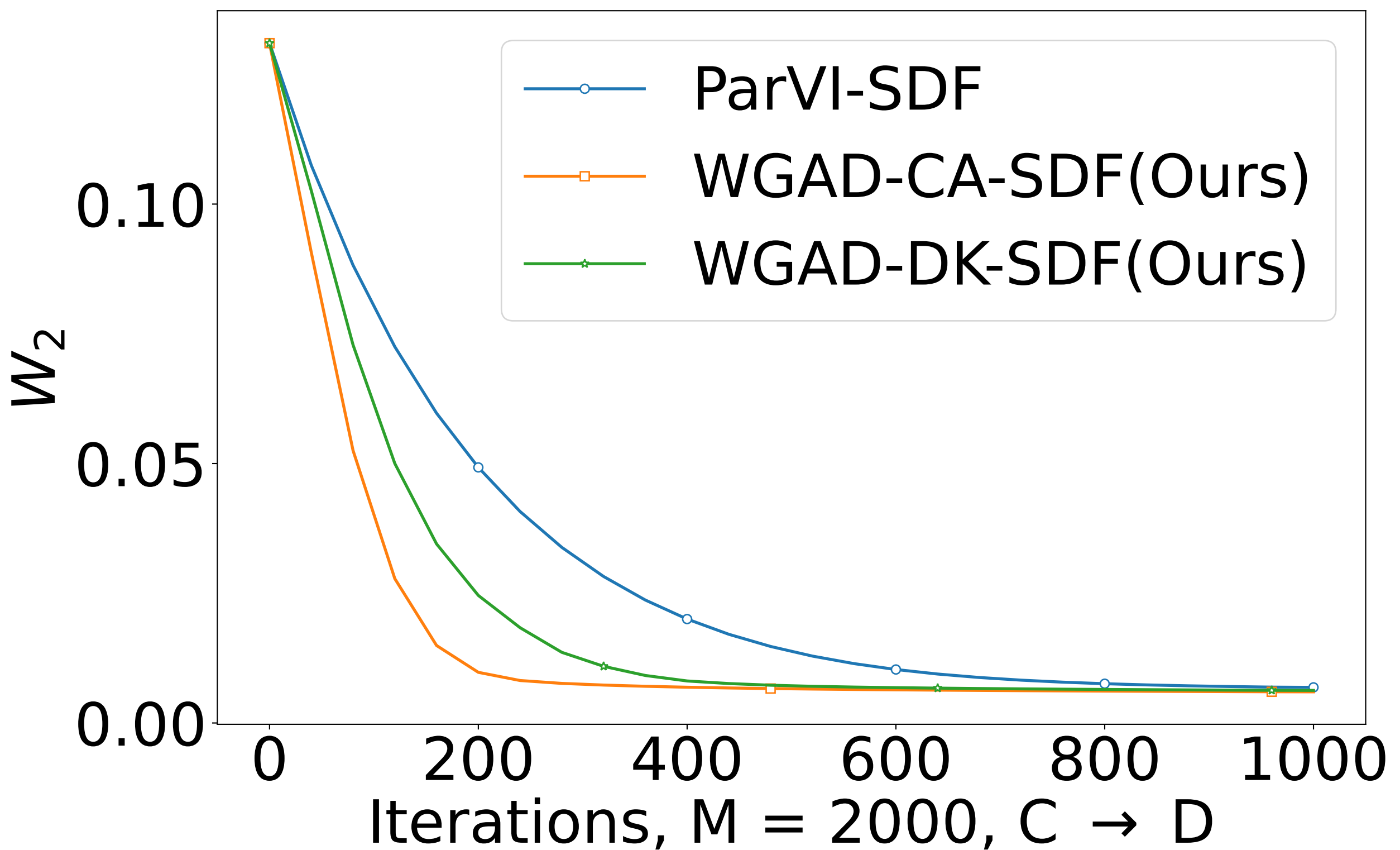

Supplement: Supplementary file 1 [file entropy-26-00679-s001.zip › dpvi_discrete-master/figures_morphing/5_figures_big/SD_w2CD.pdf]

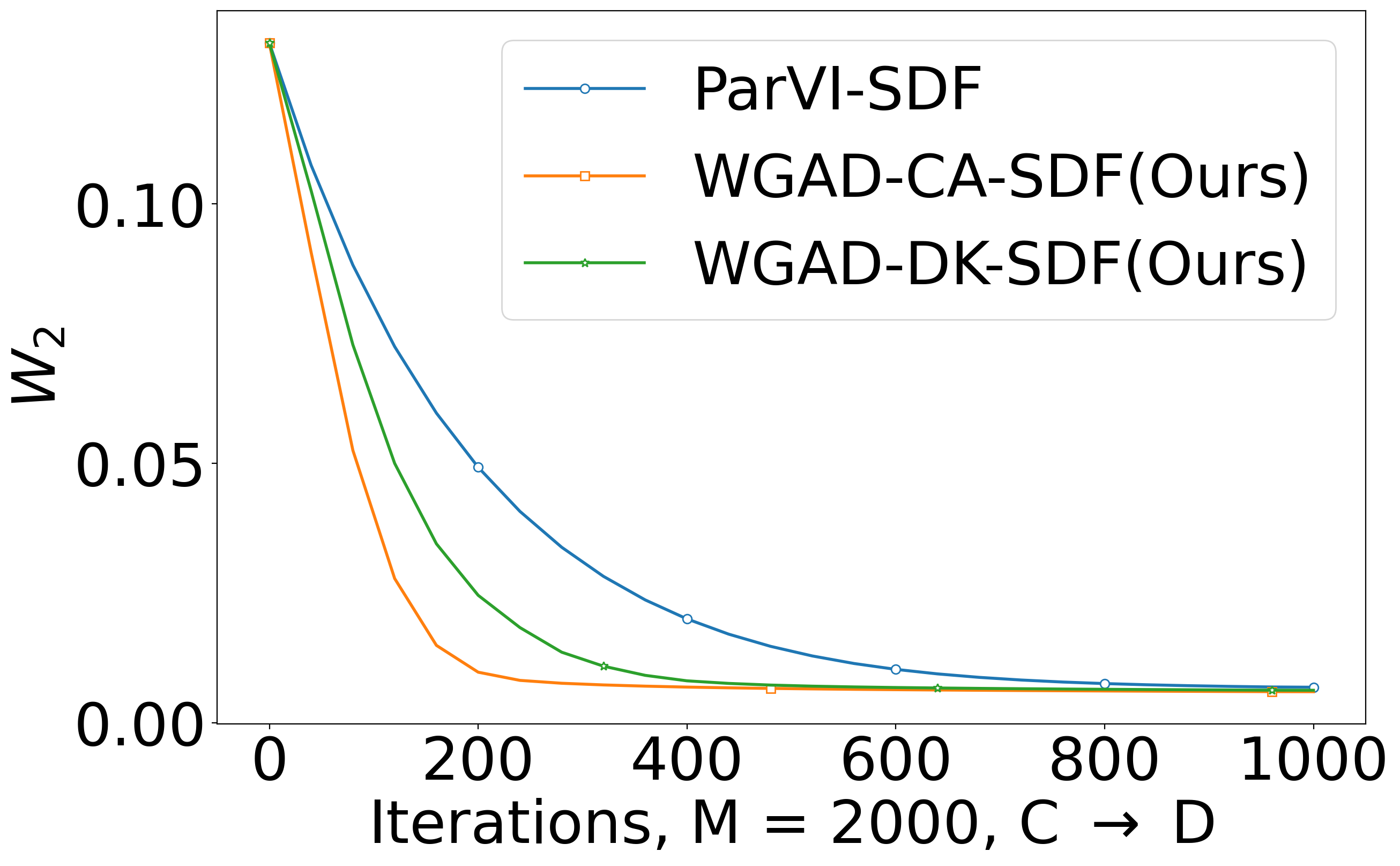

Supplement: Supplementary file 1 [file entropy-26-00679-s001.zip › dpvi_discrete-master/figures_morphing/5_figures_big/SD_w2CD.png]

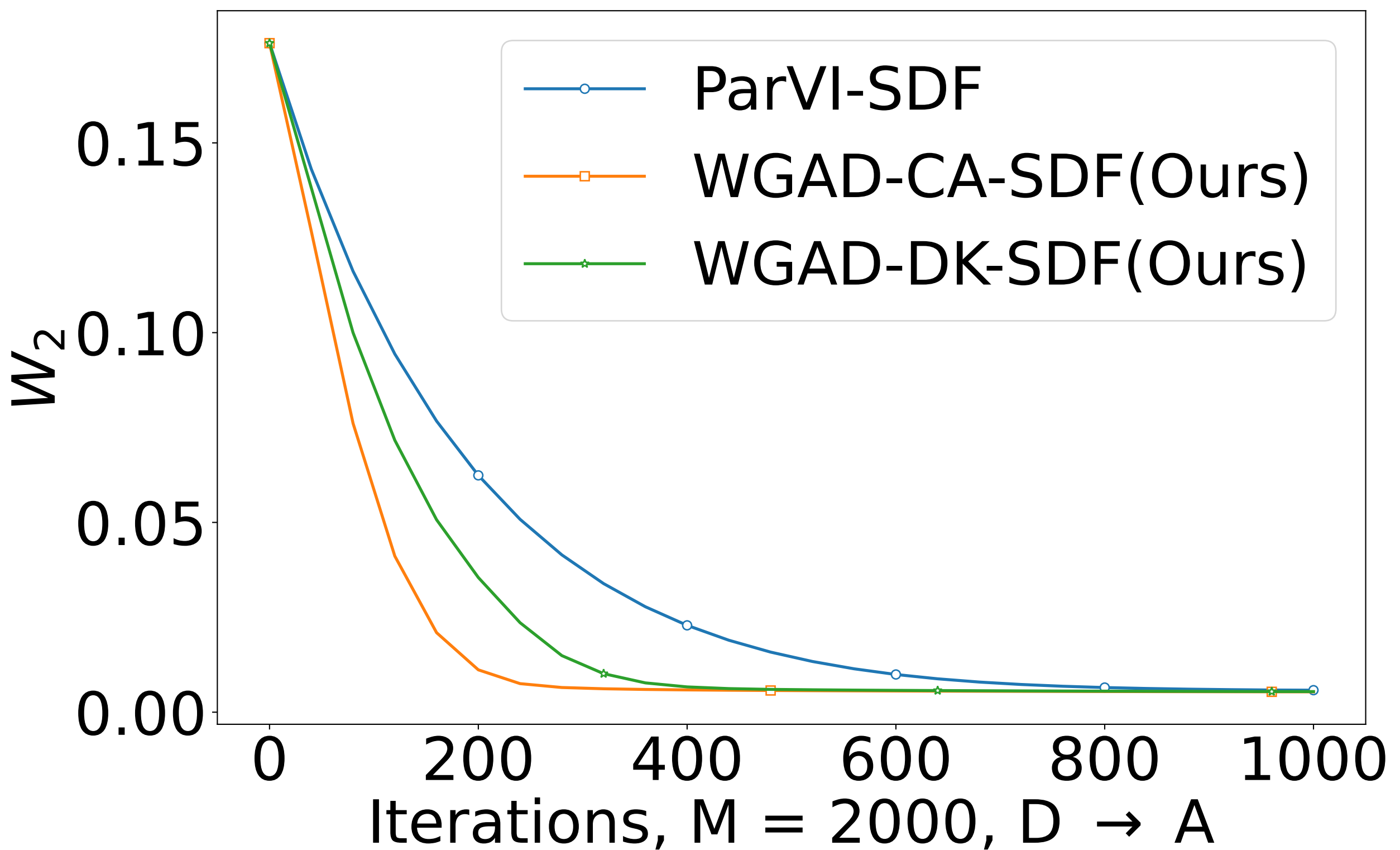

Supplement: Supplementary file 1 [file entropy-26-00679-s001.zip › dpvi_discrete-master/figures_morphing/5_figures_big/SD_w2DA.pdf]

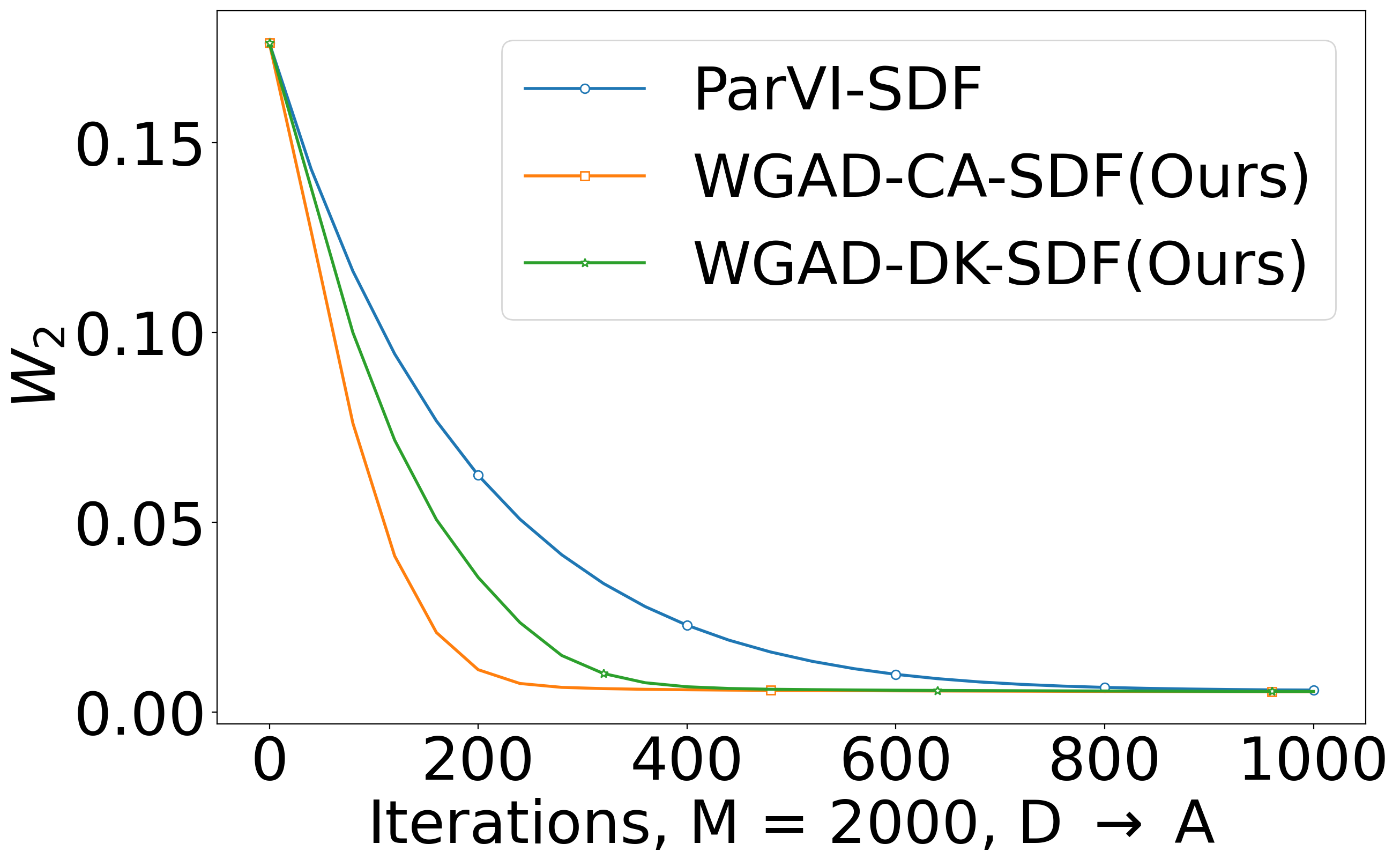

Supplement: Supplementary file 1 [file entropy-26-00679-s001.zip › dpvi_discrete-master/figures_morphing/5_figures_big/SD_w2DA.png]

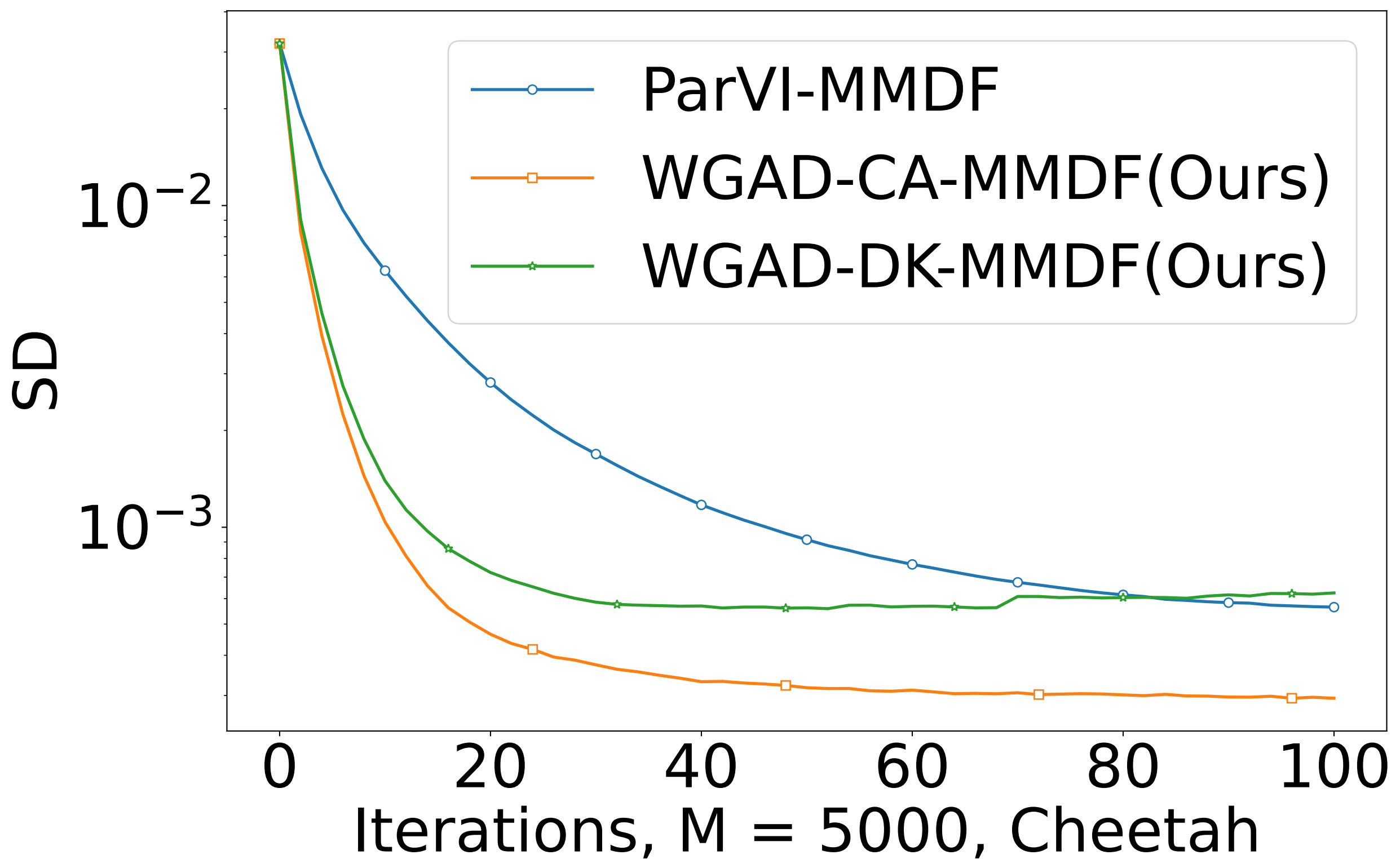

Supplement: Supplementary file 1 [file entropy-26-00679-s001.zip › dpvi_discrete-master/figures_new/5_figures_big/MMDF_w2.pdf]

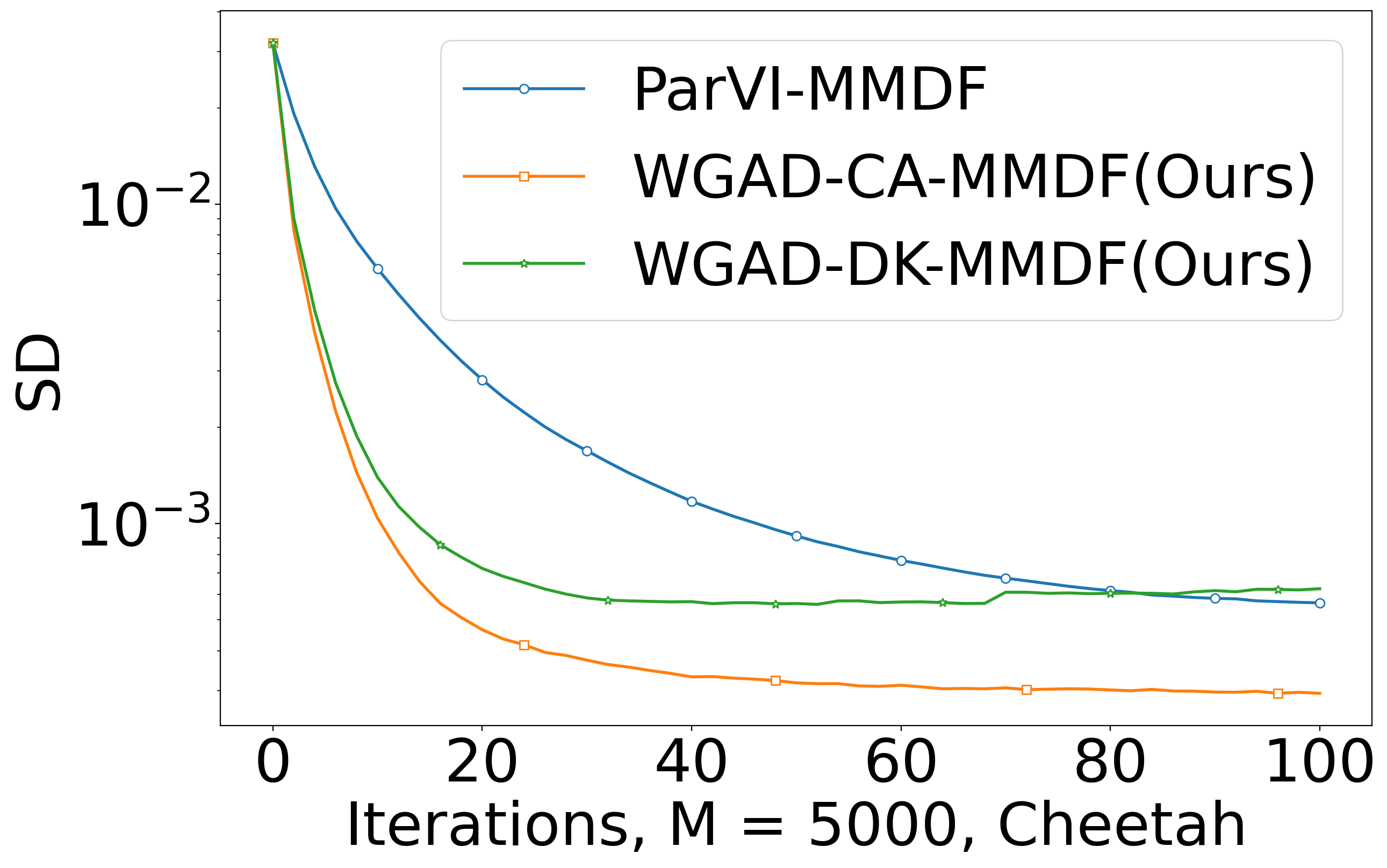

Supplement: Supplementary file 1 [file entropy-26-00679-s001.zip › dpvi_discrete-master/figures_new/5_figures_big/MMDF_w2.png]

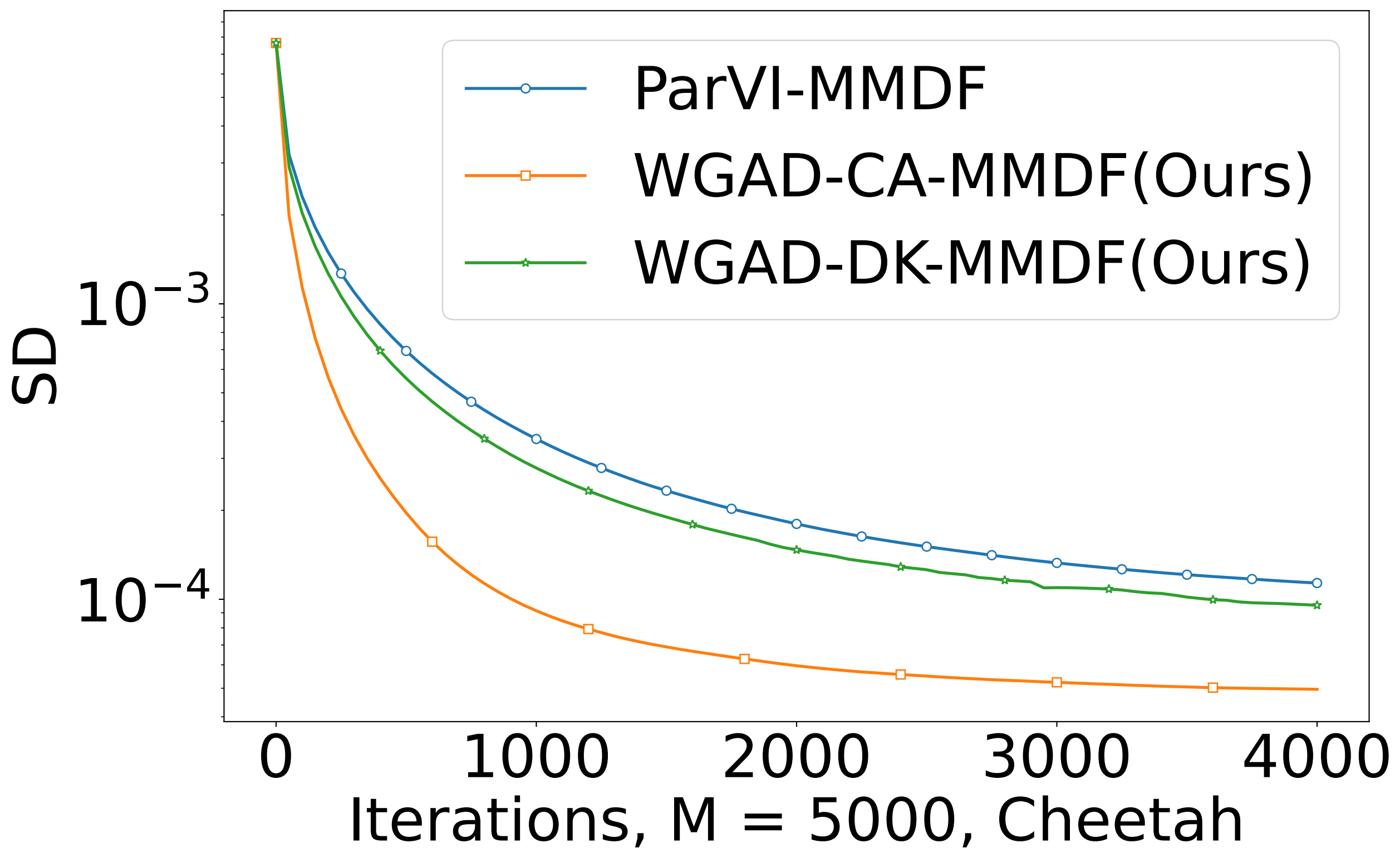

Supplement: Supplementary file 1 [file entropy-26-00679-s001.zip › dpvi_discrete-master/figures_new/5_figures_big/MMDF_w2_cheetah.pdf]

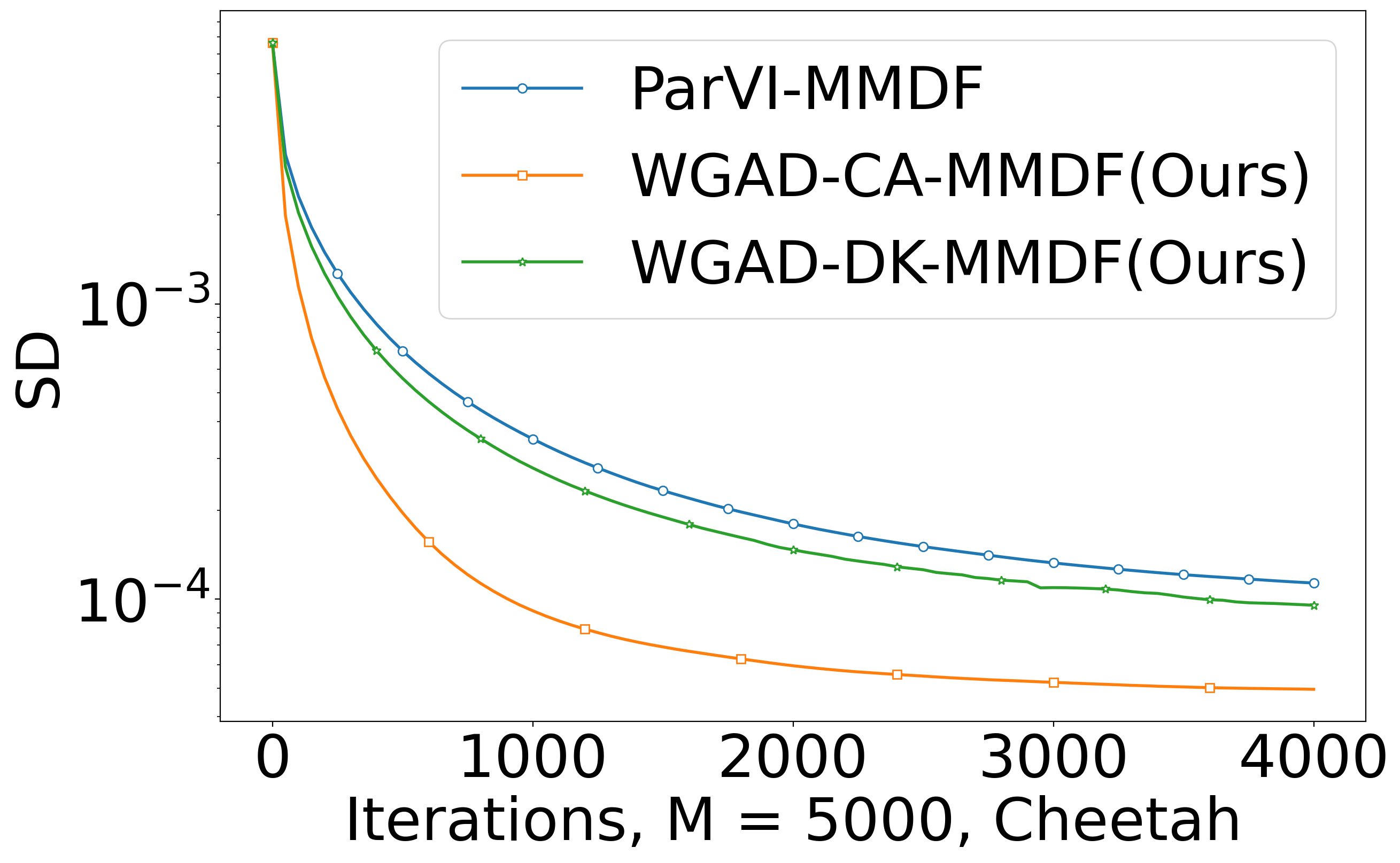

Supplement: Supplementary file 1 [file entropy-26-00679-s001.zip › dpvi_discrete-master/figures_new/5_figures_big/MMDF_w2_cheetah.png]

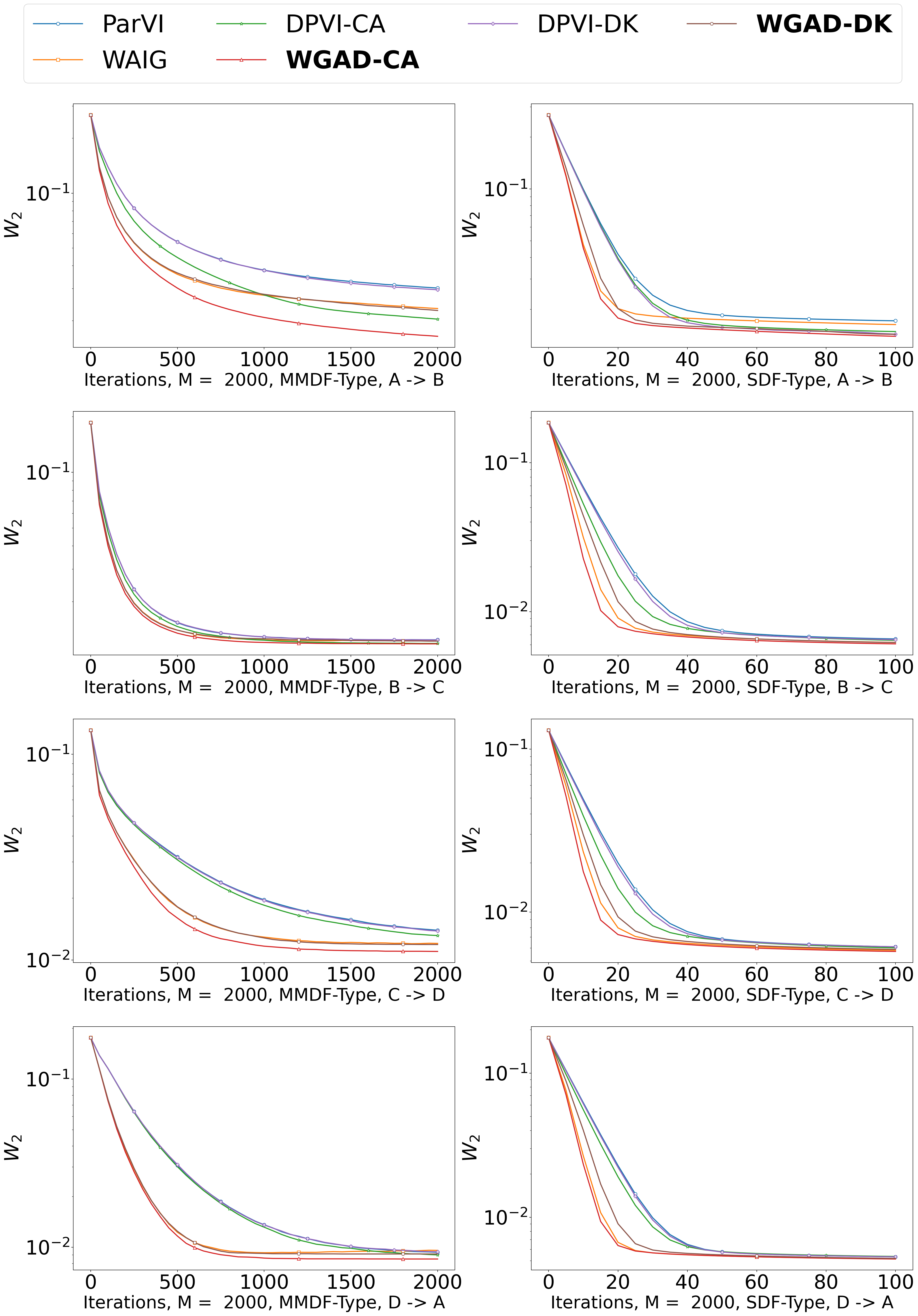

Supplement: Supplementary file 1 [file entropy-26-00679-s001.zip › dpvi_discrete-master/figures_new/5_figures_big/SD__BLOB_w2_ch.png]

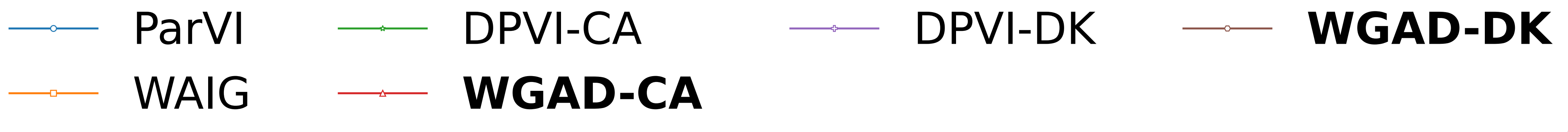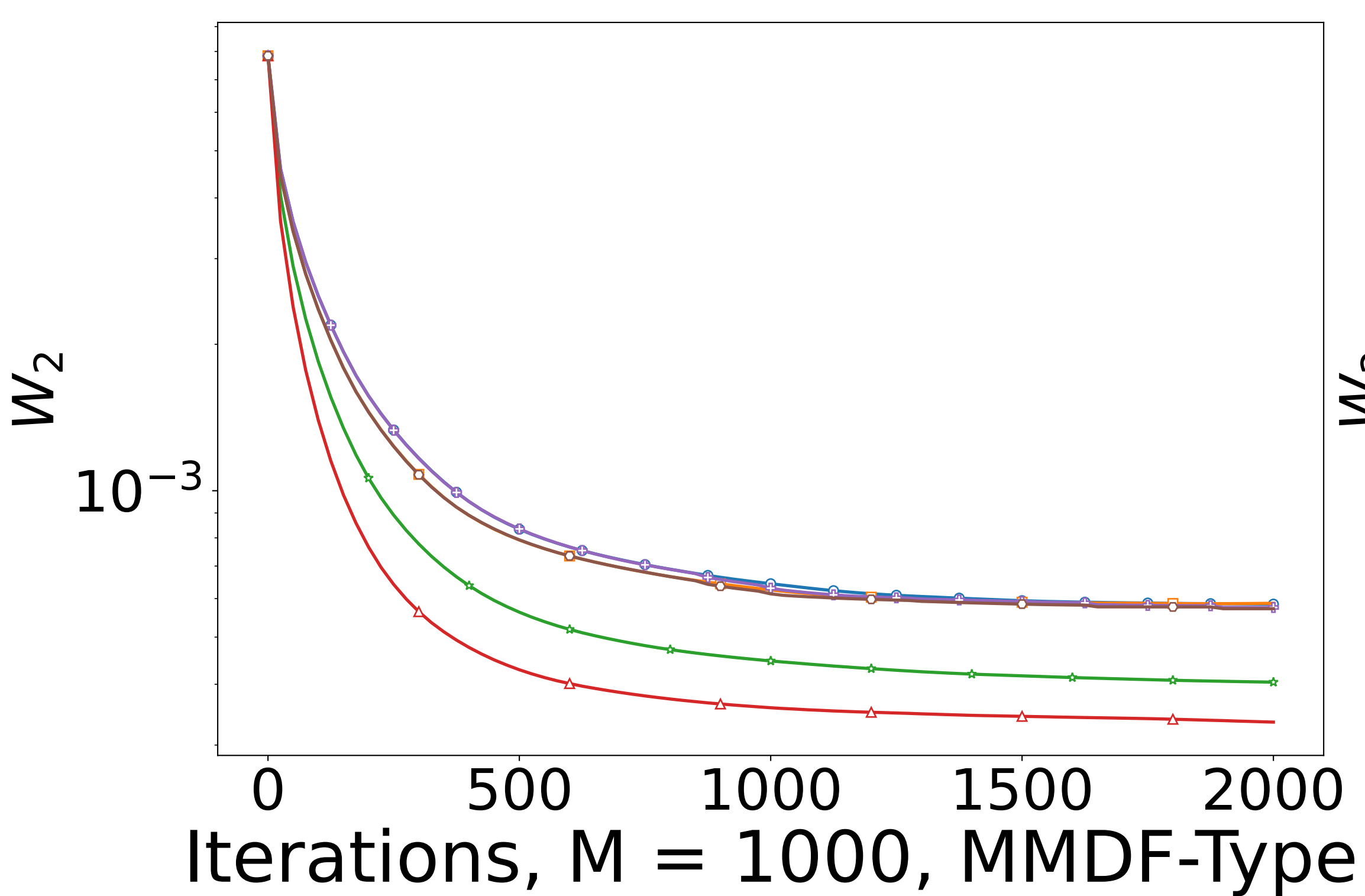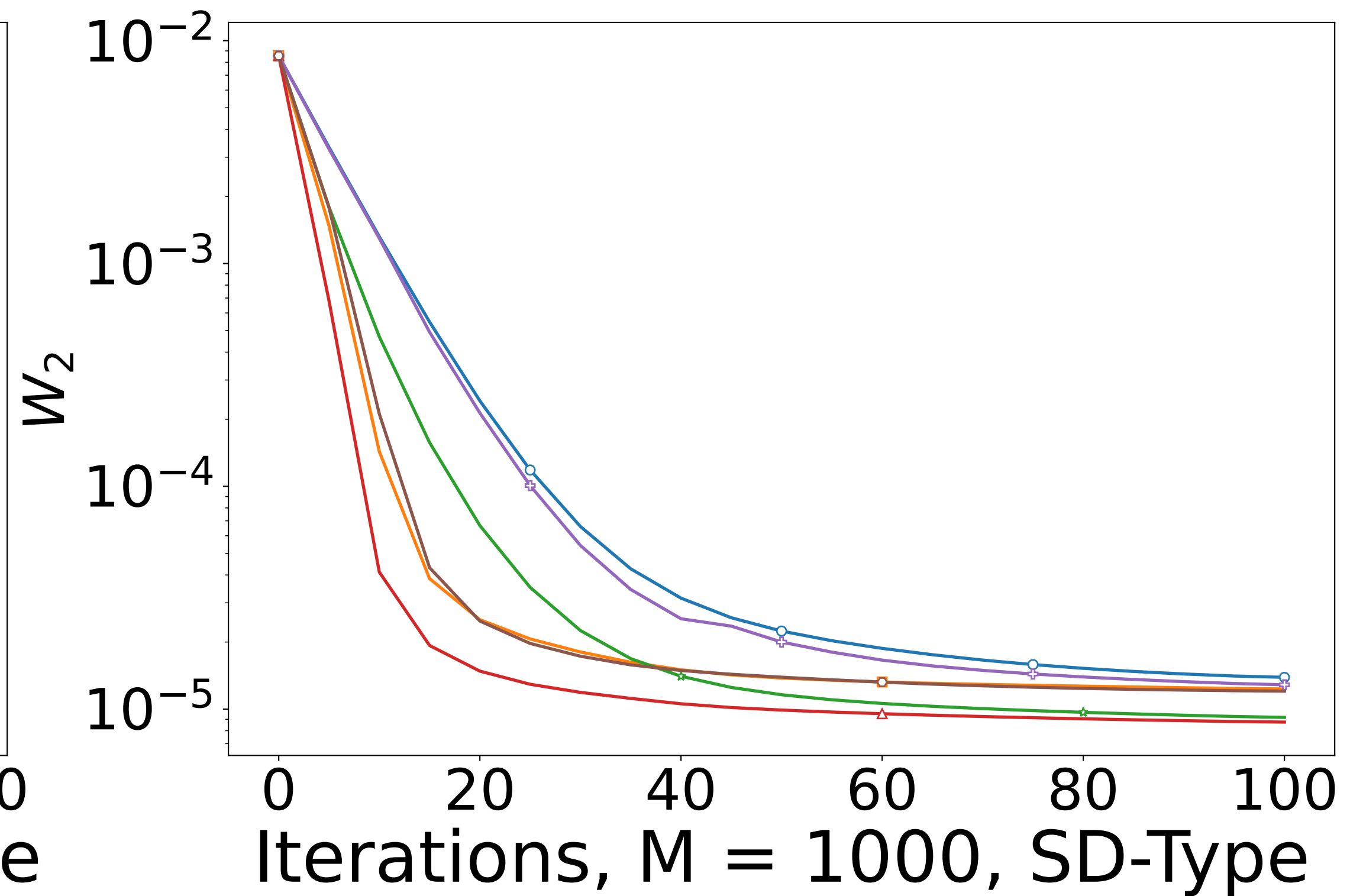

Supplement: Supplementary file 1 [file entropy-26-00679-s001.zip › dpvi_discrete-master/figures_new/5_figures_big/SD_sketching.pdf]

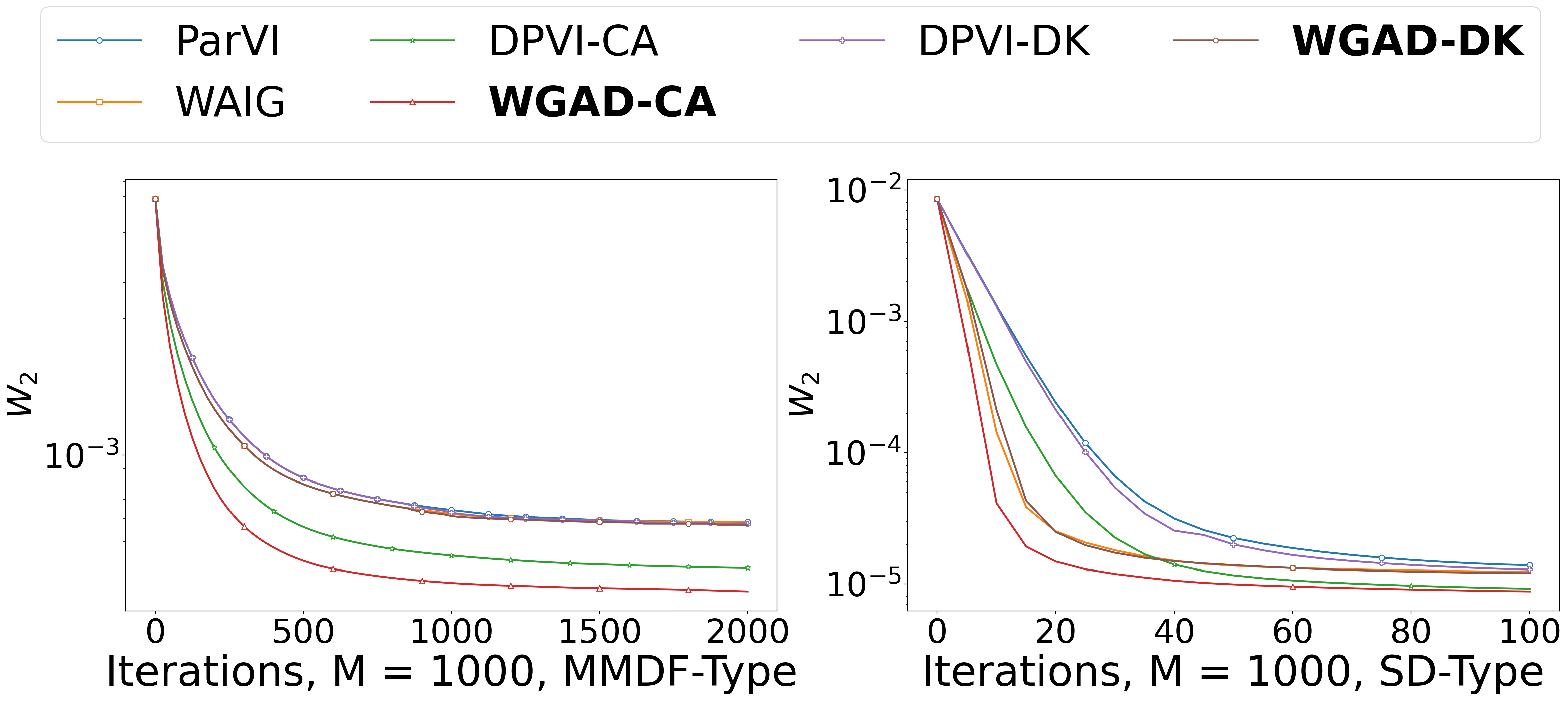

Supplement: Supplementary file 1 [file entropy-26-00679-s001.zip › dpvi_discrete-master/figures_new/5_figures_big/SD_sketching.png]

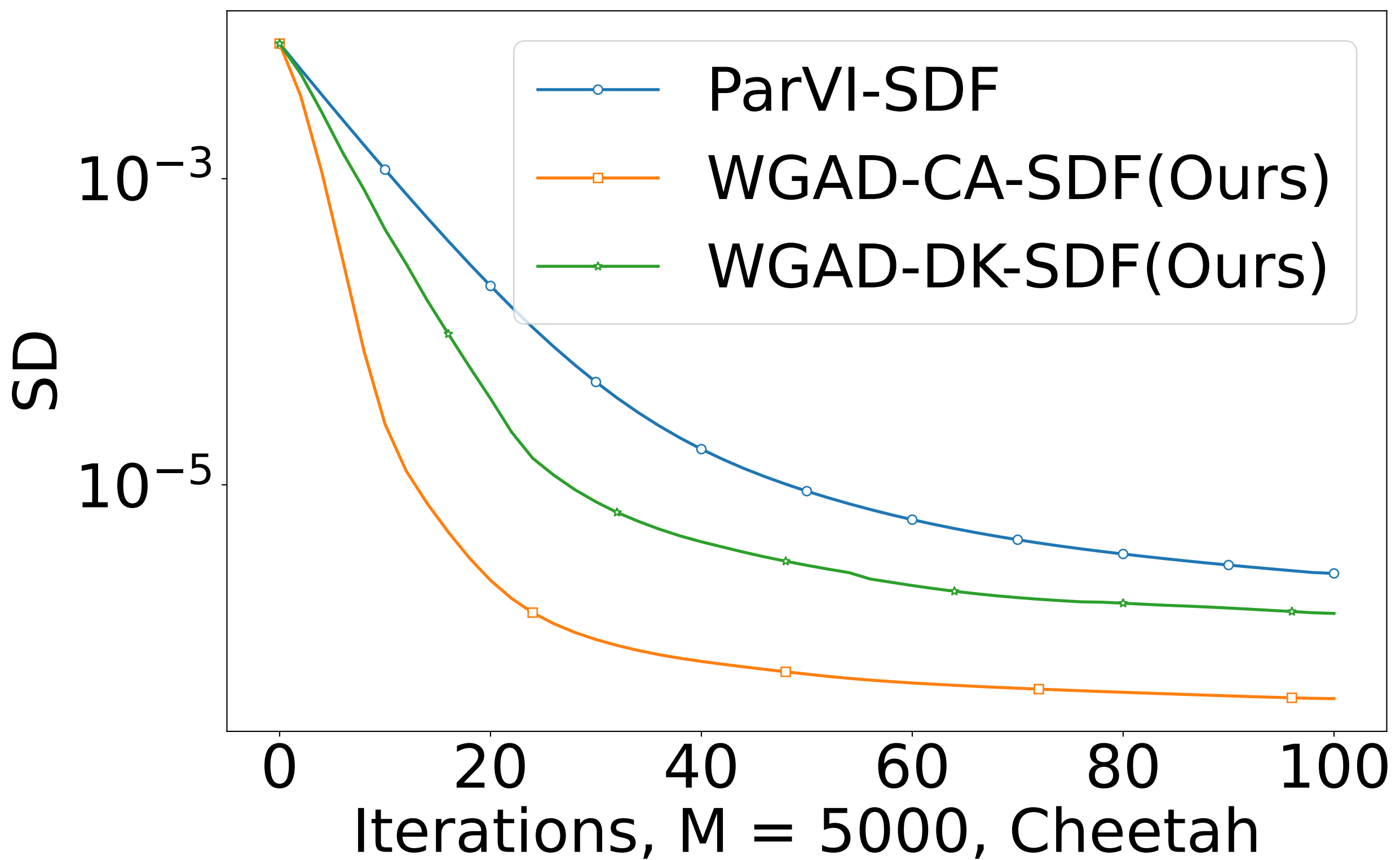

Supplement: Supplementary file 1 [file entropy-26-00679-s001.zip › dpvi_discrete-master/figures_new/5_figures_big/SD_w2_cheetah.pdf]

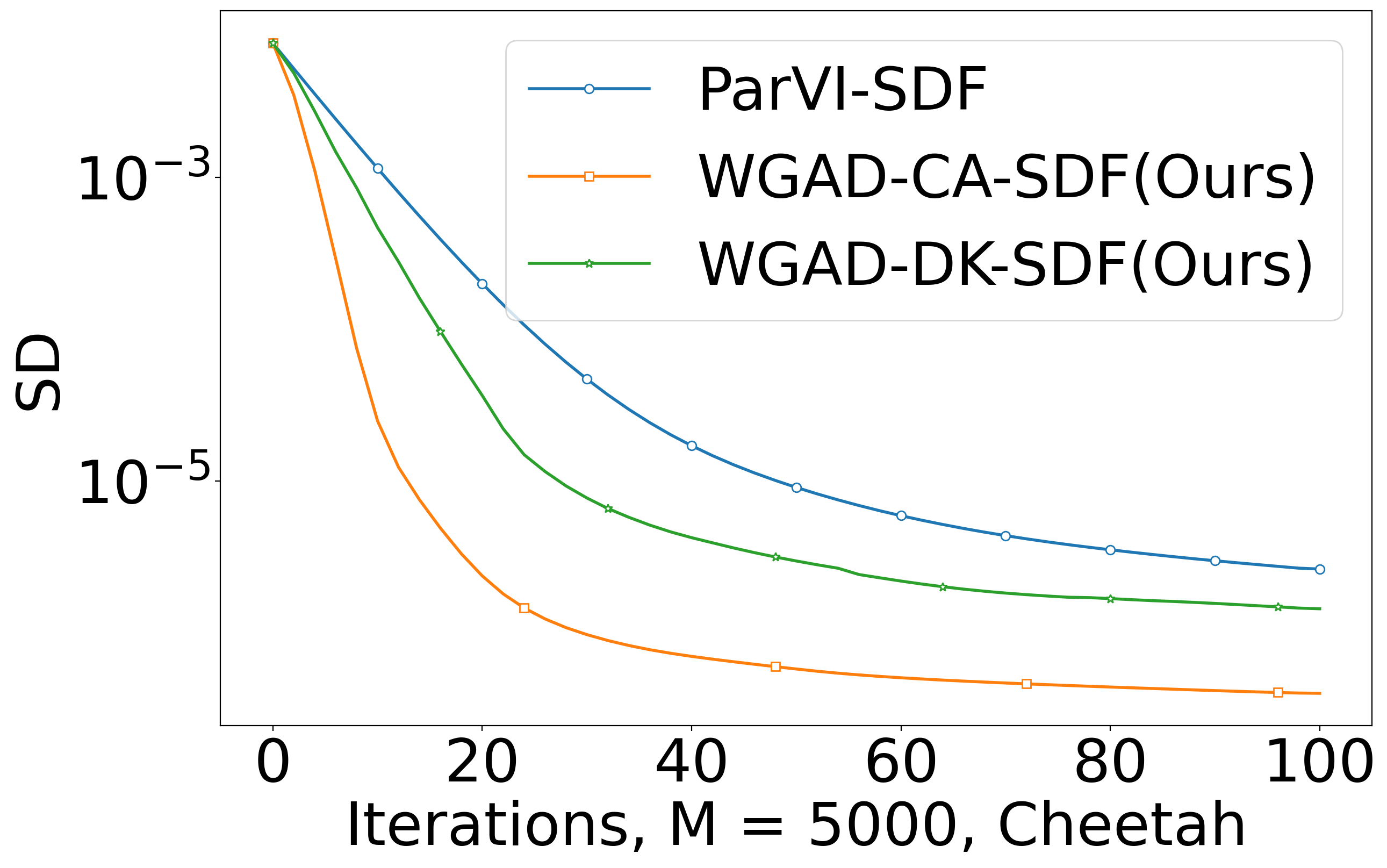

Supplement: Supplementary file 1 [file entropy-26-00679-s001.zip › dpvi_discrete-master/figures_new/5_figures_big/SD_w2_cheetah.png]

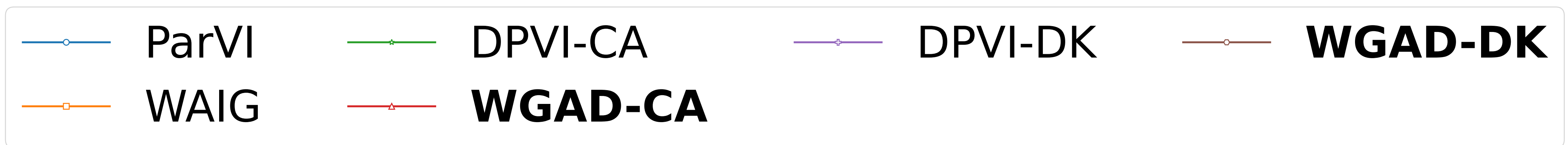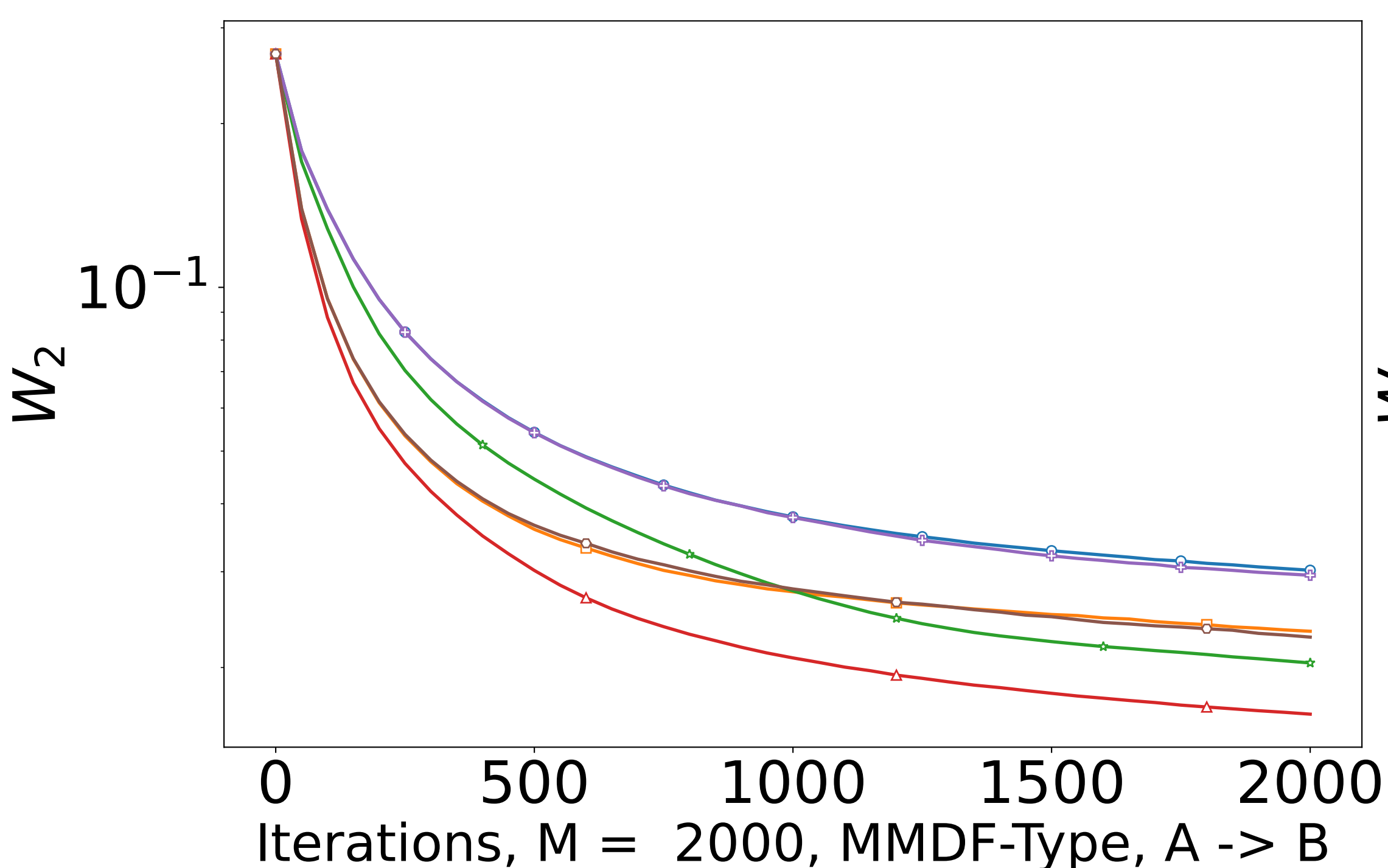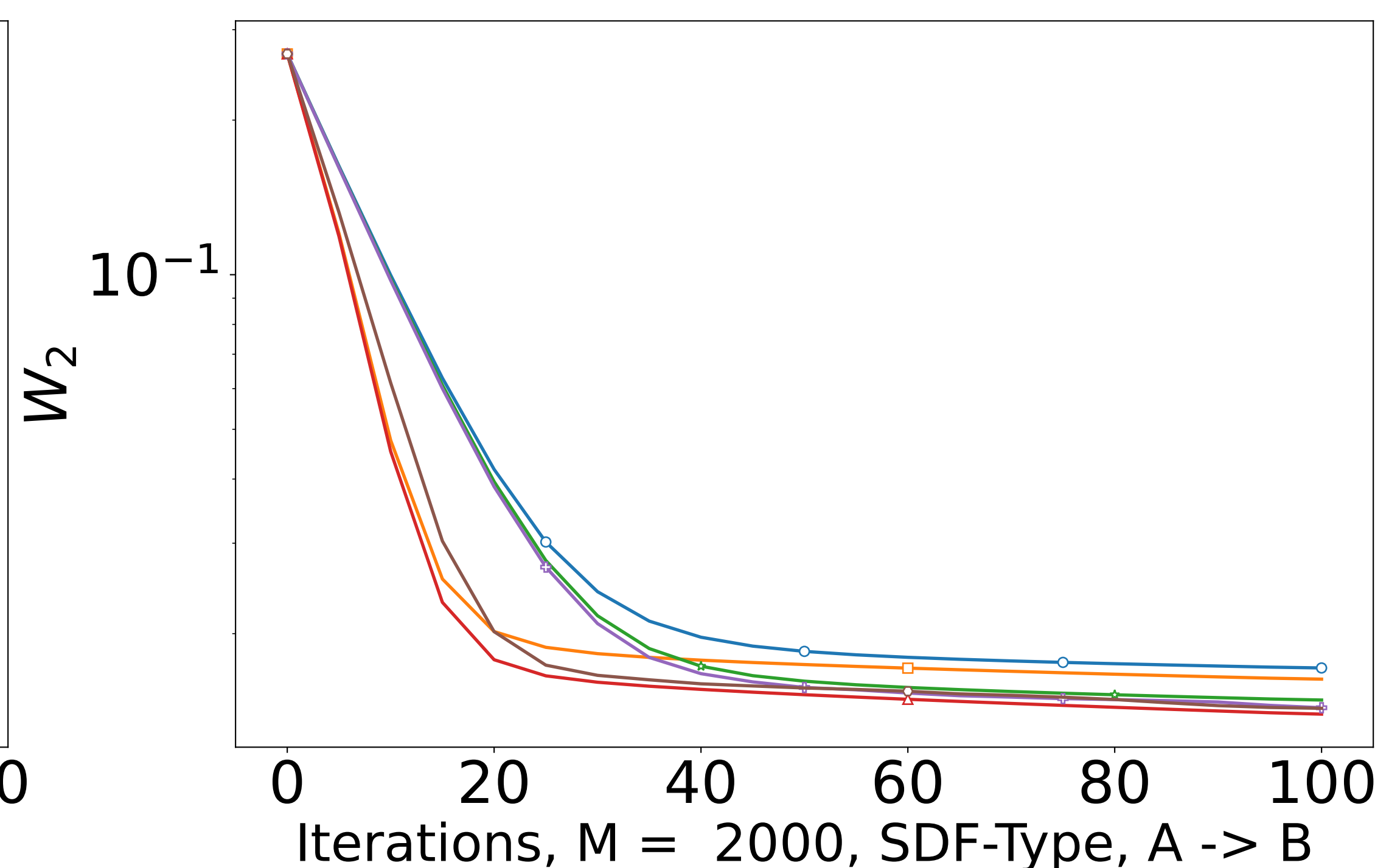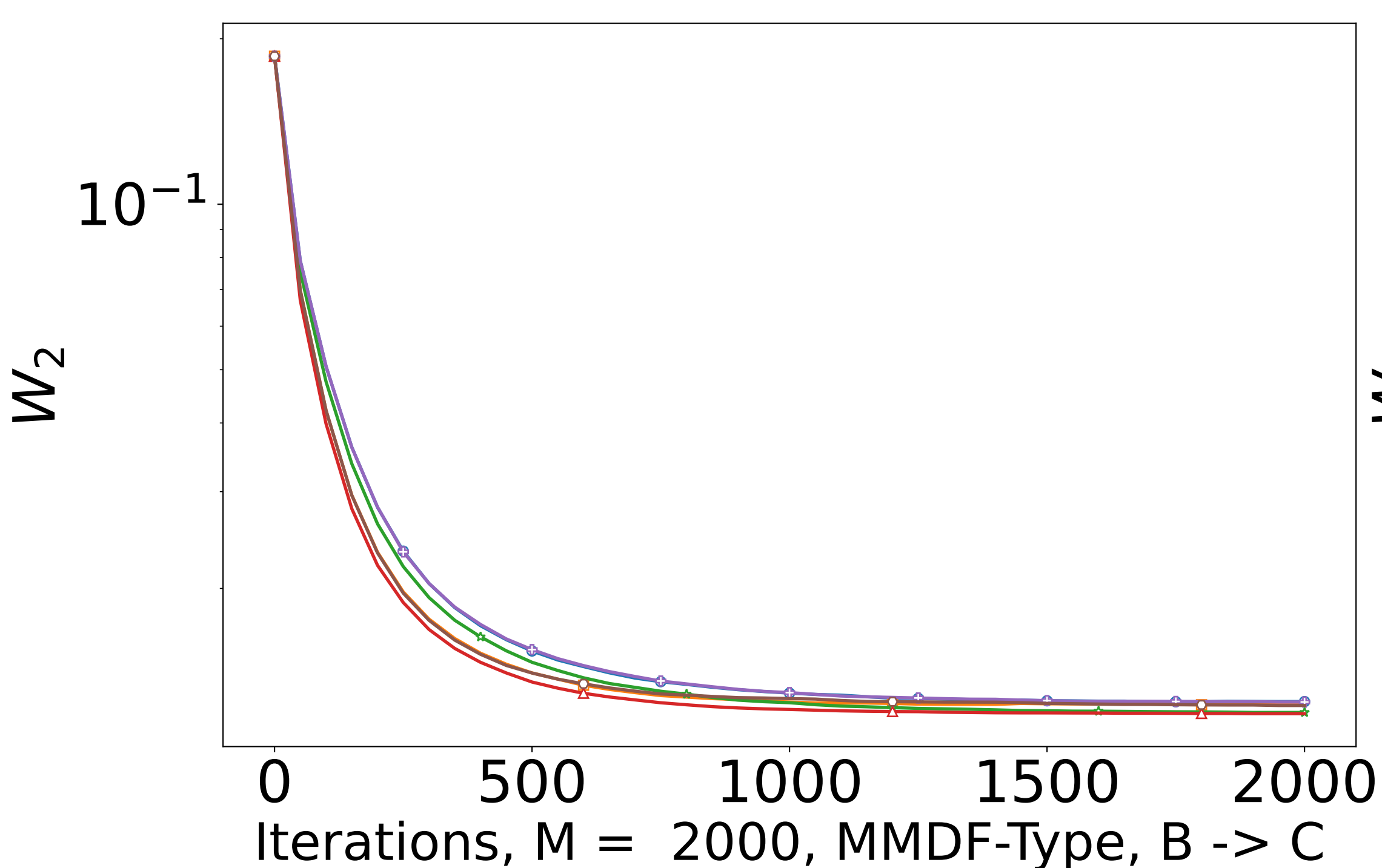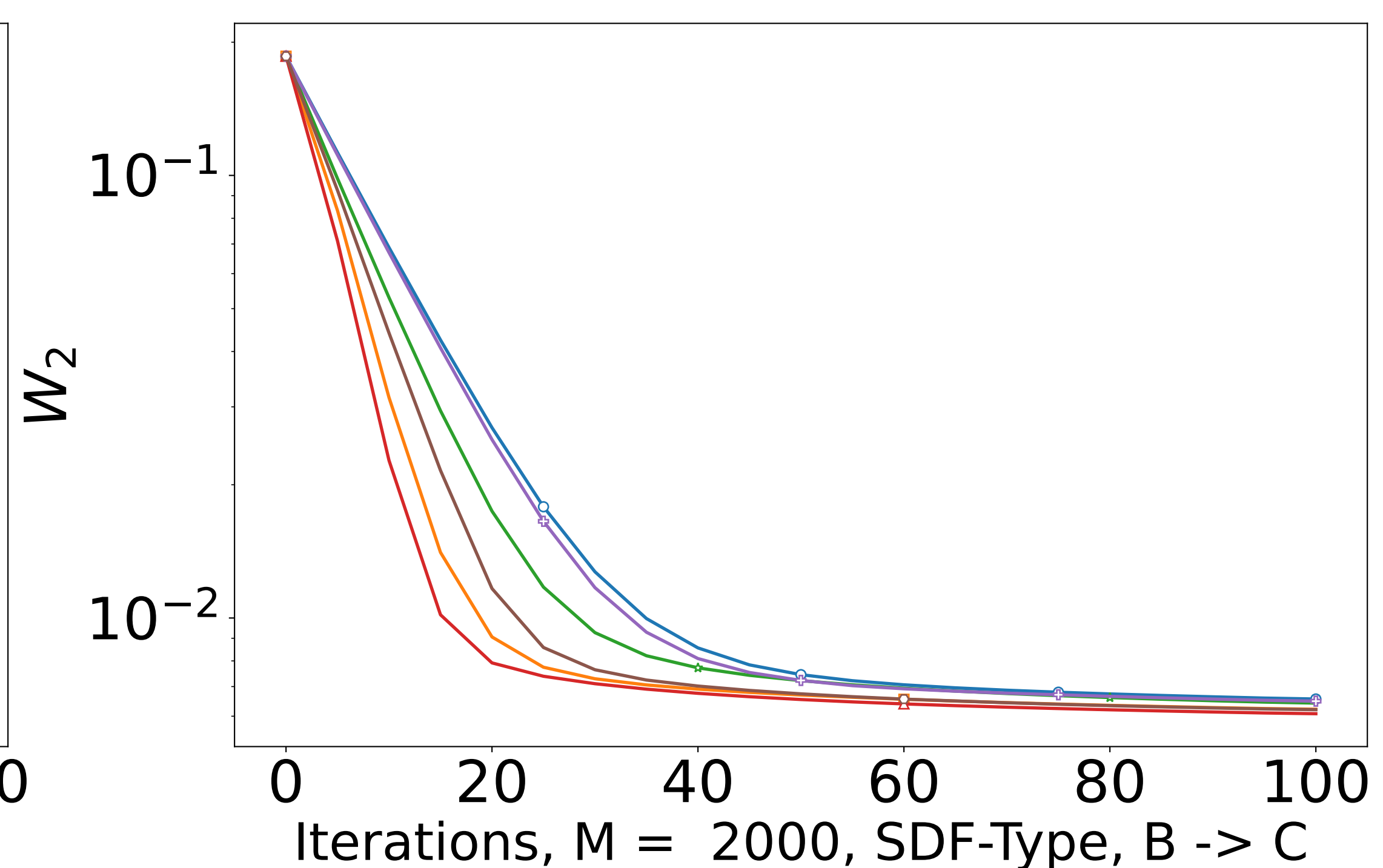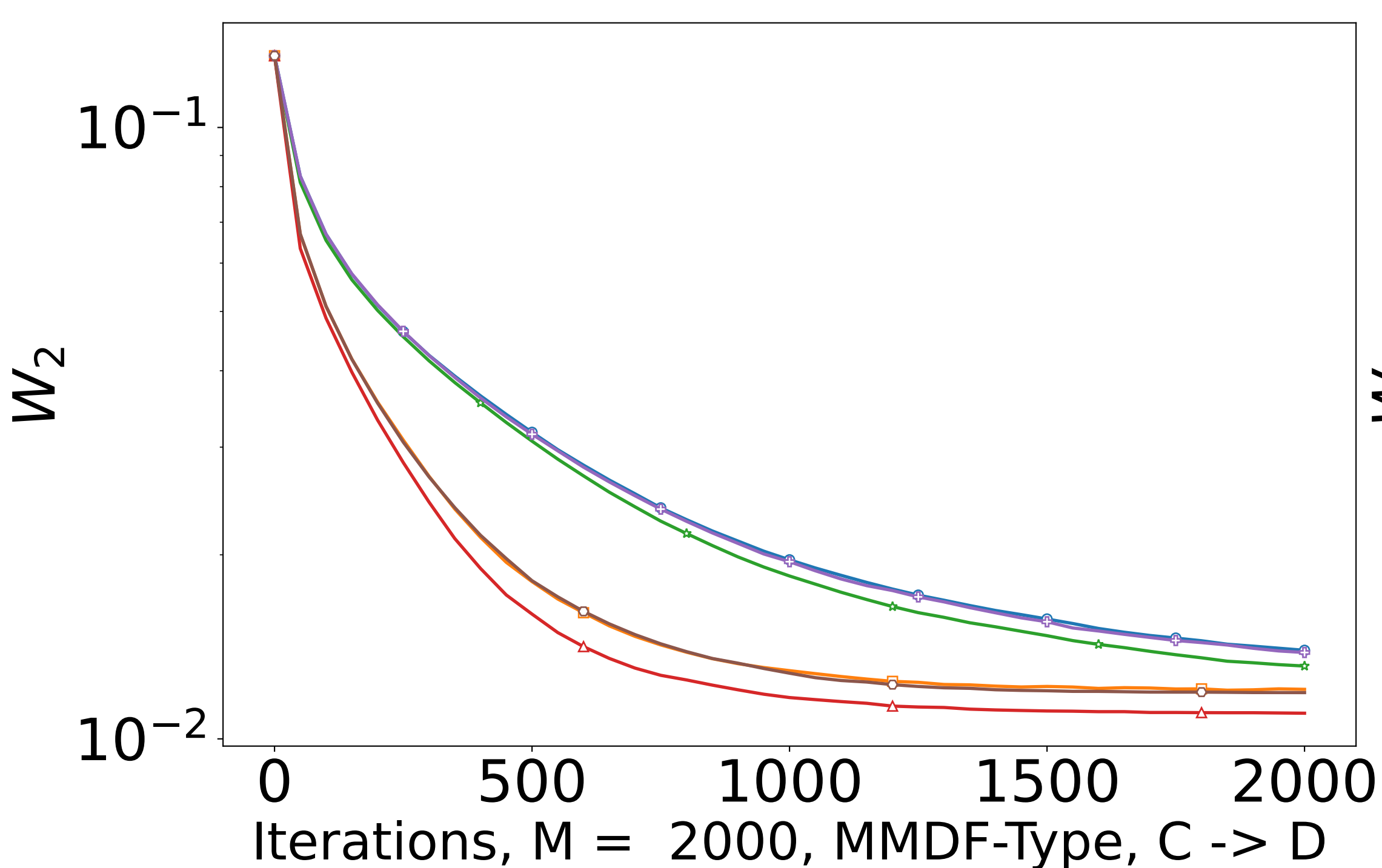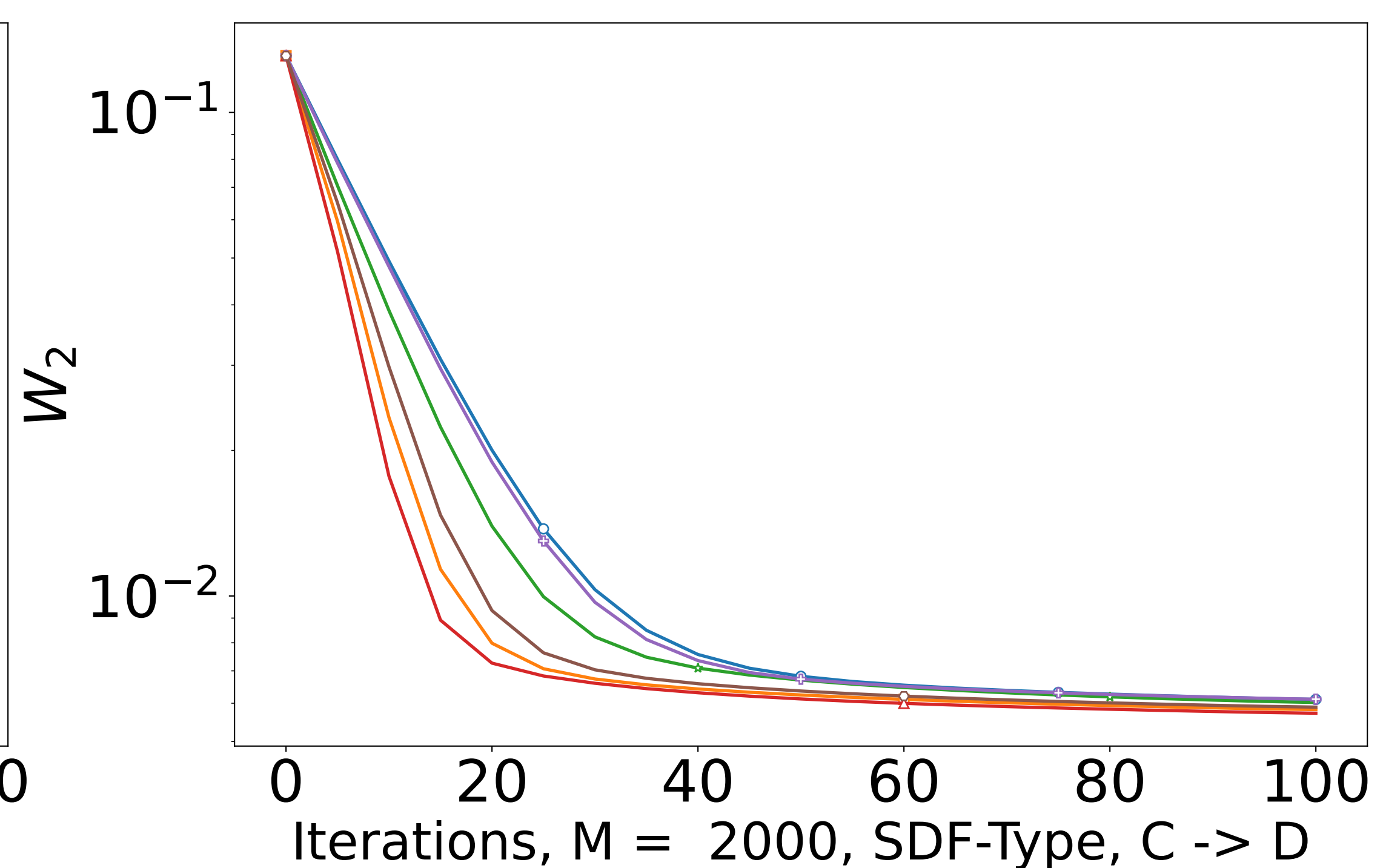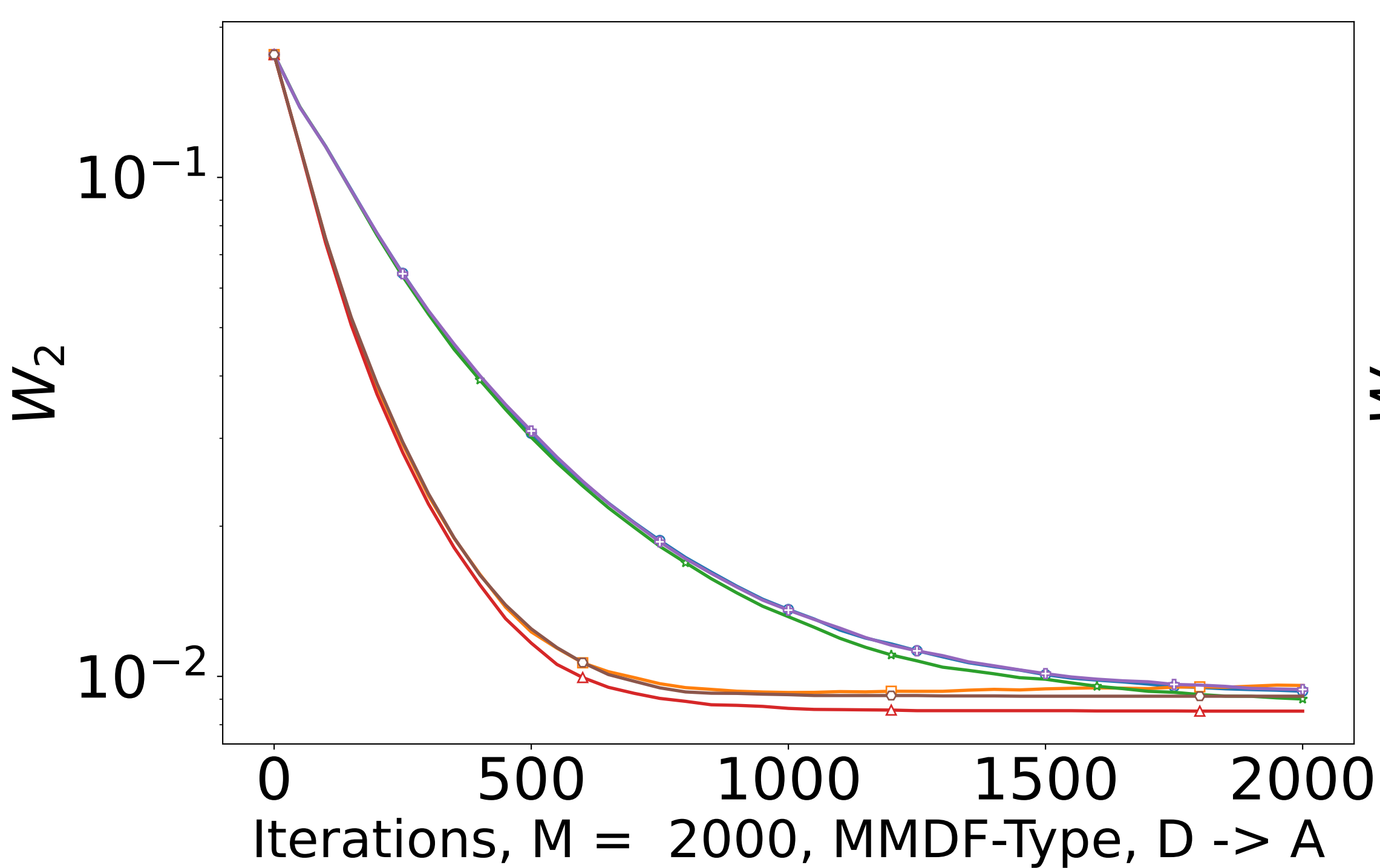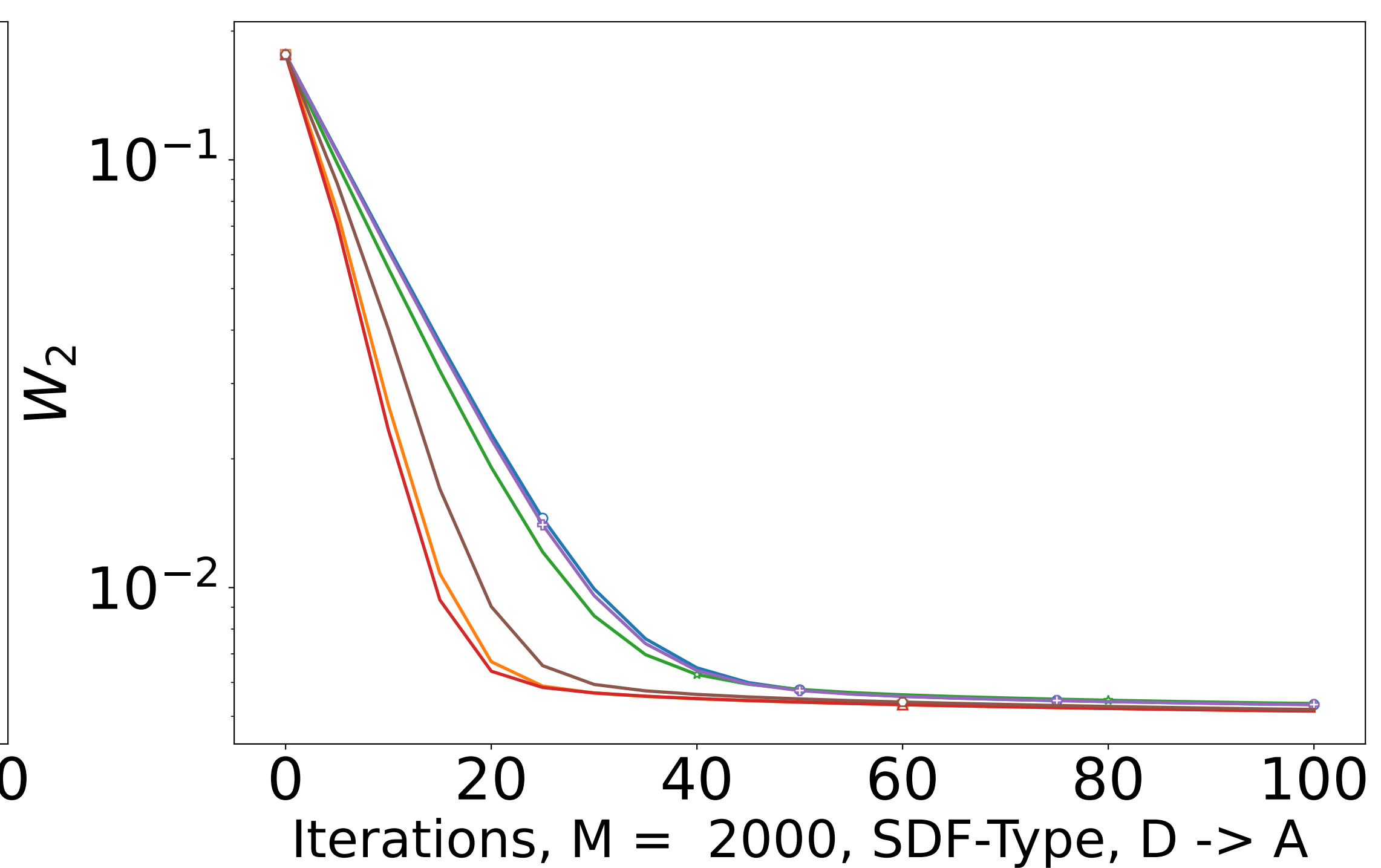

Supplement: Supplementary file 1 [file entropy-26-00679-s001.zip › dpvi_discrete-master/figures_new/5_figures_big/morphing_line.pdf]

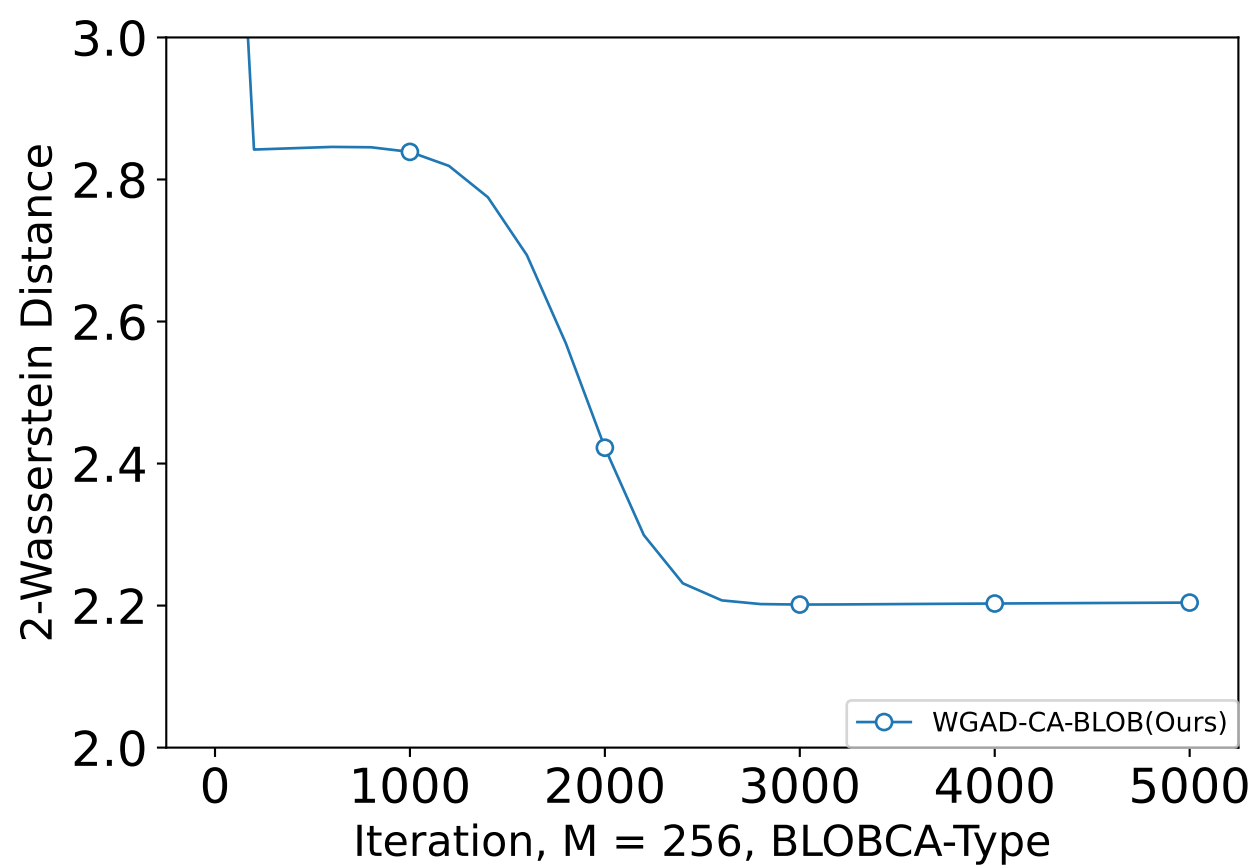

Supplement: Supplementary file 1 [file entropy-26-00679-s001.zip › dpvi_discrete-master/plot/multi_gaussian/BLOBCA_multi_gaussian_256/BLOBCA_w2.pdf]

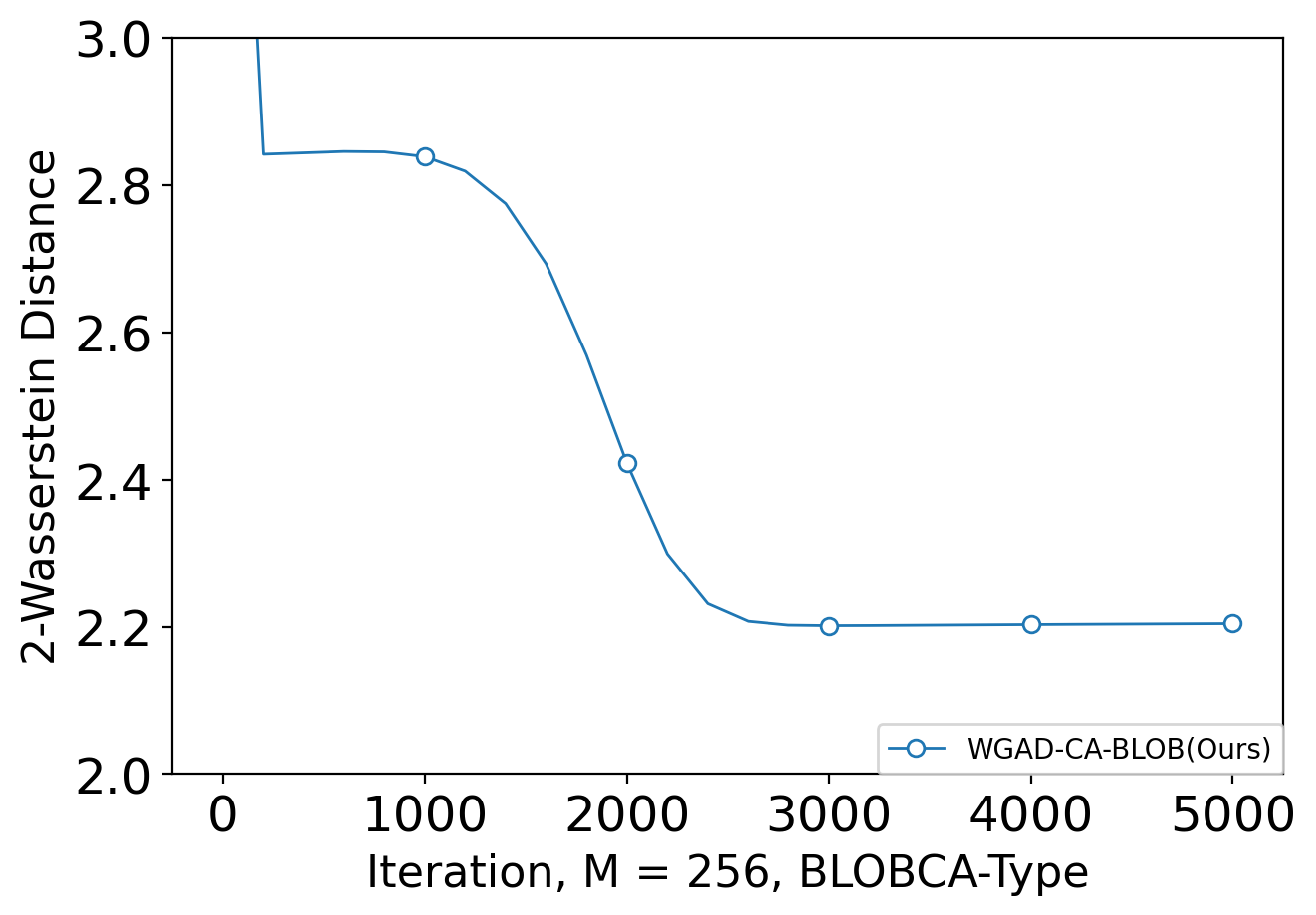

Supplement: Supplementary file 1 [file entropy-26-00679-s001.zip › dpvi_discrete-master/plot/multi_gaussian/BLOBCA_multi_gaussian_256/BLOBCA_w2.png]
