# Supplementary figures and images for: GAD-PVI: A General Accelerated Dynamic-Weight Particle-Based Variational Inference Framework (part 1 of 2)
Source: Entropy (Basel). 2024 Aug 11;26(8):679. doi: 10.3390/e26080679 (PMC11354113; doi:10.3390/e26080679)

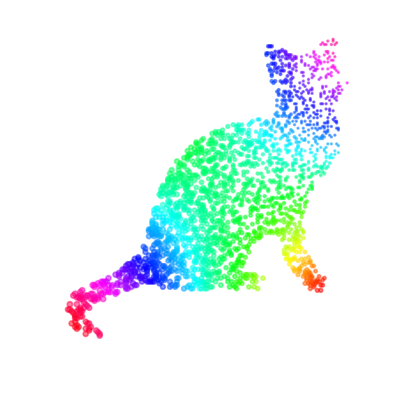

Supplement: Supplementary file 1 [file entropy-26-00679-s001.zip › dpvi_discrete-master/_tmp/demo_fisher/iter0.png]

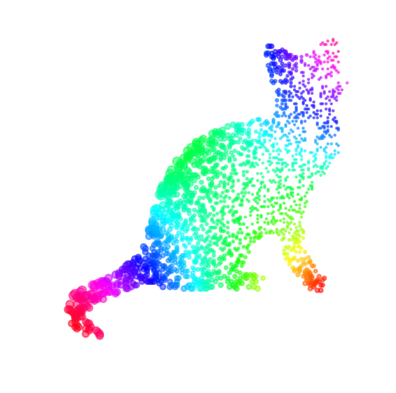

Supplement: Supplementary file 1 [file entropy-26-00679-s001.zip › dpvi_discrete-master/_tmp/demo_fisher/iter1.png]

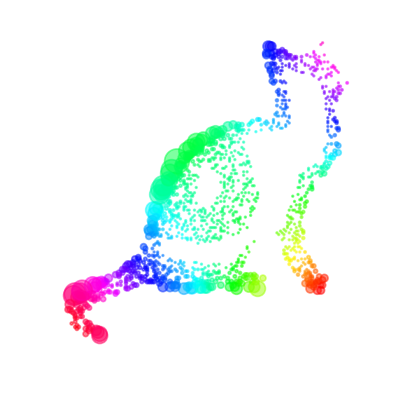

Supplement: Supplementary file 1 [file entropy-26-00679-s001.zip › dpvi_discrete-master/_tmp/demo_fisher/iter10.png]

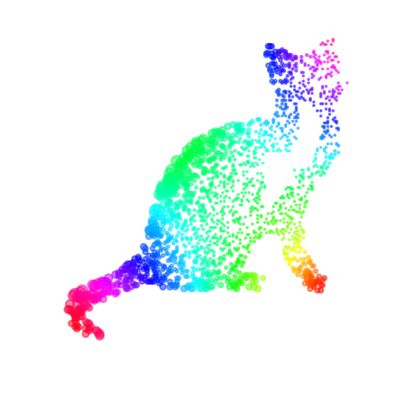

Supplement: Supplementary file 1 [file entropy-26-00679-s001.zip › dpvi_discrete-master/_tmp/demo_fisher/iter2.png]

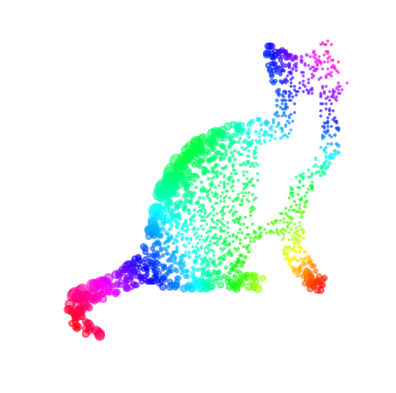

Supplement: Supplementary file 1 [file entropy-26-00679-s001.zip › dpvi_discrete-master/_tmp/demo_fisher/iter3.png]

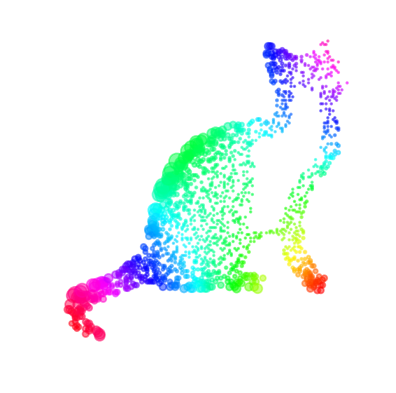

Supplement: Supplementary file 1 [file entropy-26-00679-s001.zip › dpvi_discrete-master/_tmp/demo_fisher/iter4.png]

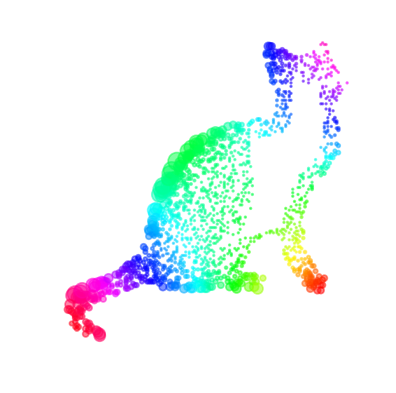

Supplement: Supplementary file 1 [file entropy-26-00679-s001.zip › dpvi_discrete-master/_tmp/demo_fisher/iter5.png]

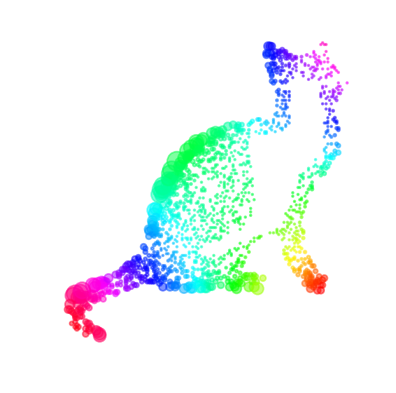

Supplement: Supplementary file 1 [file entropy-26-00679-s001.zip › dpvi_discrete-master/_tmp/demo_fisher/iter6.png]

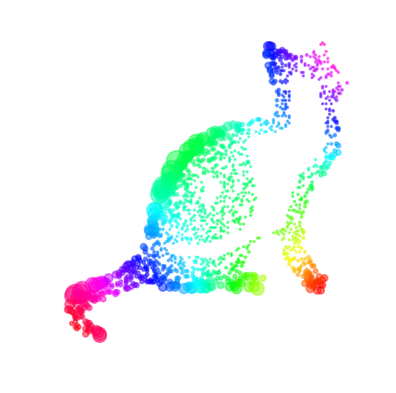

Supplement: Supplementary file 1 [file entropy-26-00679-s001.zip › dpvi_discrete-master/_tmp/demo_fisher/iter7.png]

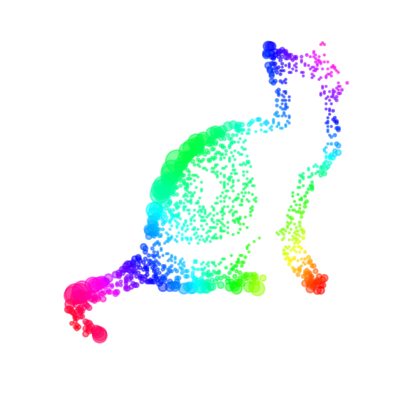

Supplement: Supplementary file 1 [file entropy-26-00679-s001.zip › dpvi_discrete-master/_tmp/demo_fisher/iter8.png]

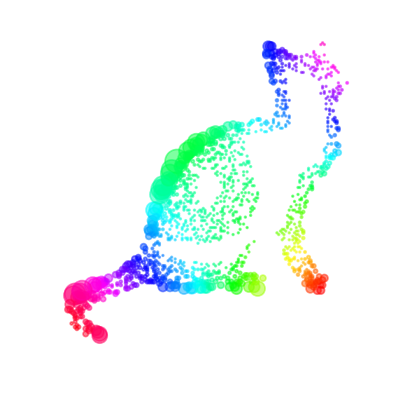

Supplement: Supplementary file 1 [file entropy-26-00679-s001.zip › dpvi_discrete-master/_tmp/demo_fisher/iter9.png]

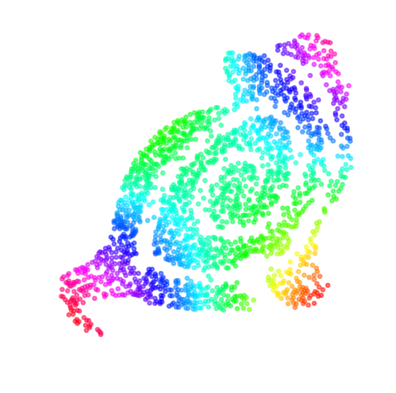

Supplement: Supplementary file 1 [file entropy-26-00679-s001.zip › dpvi_discrete-master/_tmp/demo_sd/iter0.png]

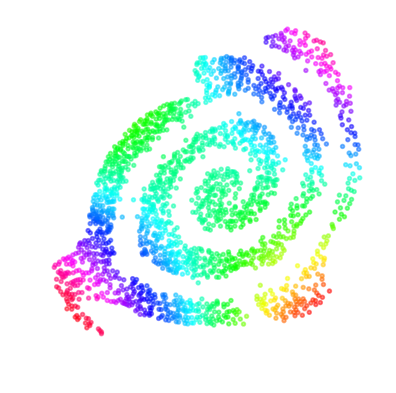

Supplement: Supplementary file 1 [file entropy-26-00679-s001.zip › dpvi_discrete-master/_tmp/demo_sd/iter1.png]

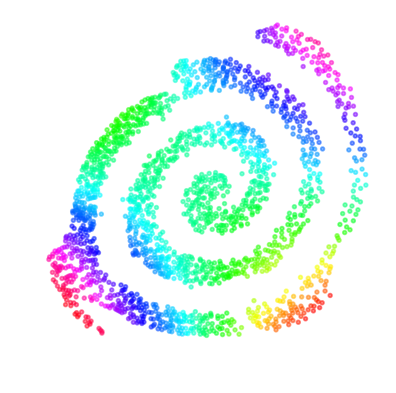

Supplement: Supplementary file 1 [file entropy-26-00679-s001.zip › dpvi_discrete-master/_tmp/demo_sd/iter2.png]

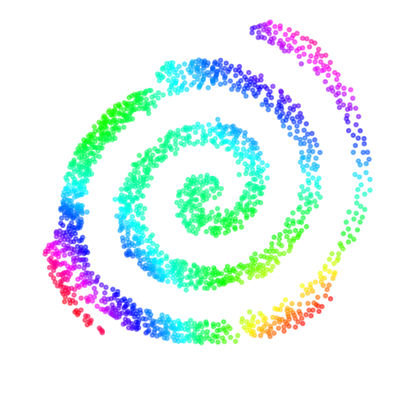

Supplement: Supplementary file 1 [file entropy-26-00679-s001.zip › dpvi_discrete-master/_tmp/demo_sd/iter3.png]

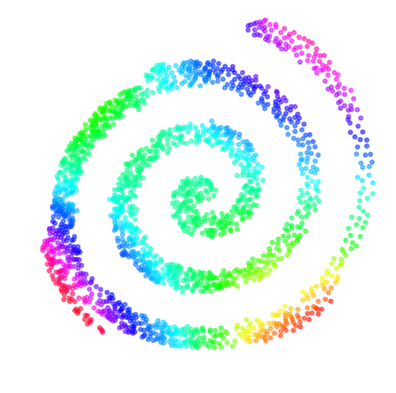

Supplement: Supplementary file 1 [file entropy-26-00679-s001.zip › dpvi_discrete-master/_tmp/demo_sd/iter4.png]

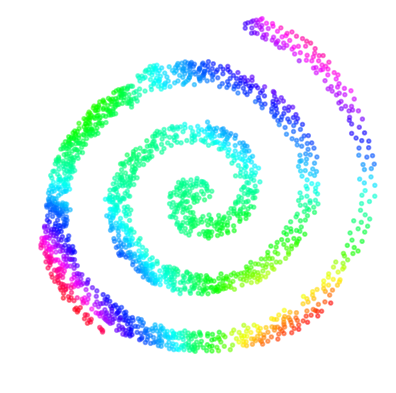

Supplement: Supplementary file 1 [file entropy-26-00679-s001.zip › dpvi_discrete-master/_tmp/demo_sd/iter5.png]

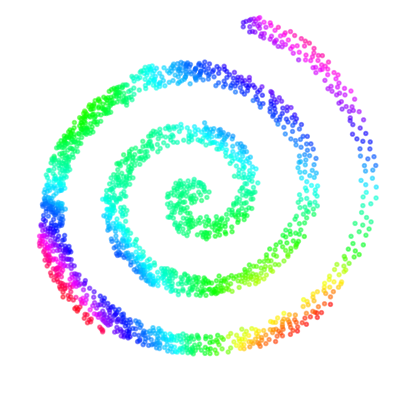

Supplement: Supplementary file 1 [file entropy-26-00679-s001.zip › dpvi_discrete-master/_tmp/demo_sd/iter6.png]

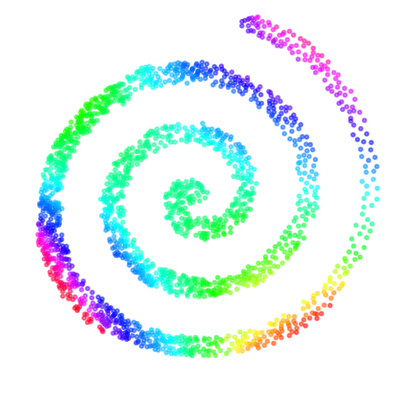

Supplement: Supplementary file 1 [file entropy-26-00679-s001.zip › dpvi_discrete-master/_tmp/demo_sd/iter7.png]

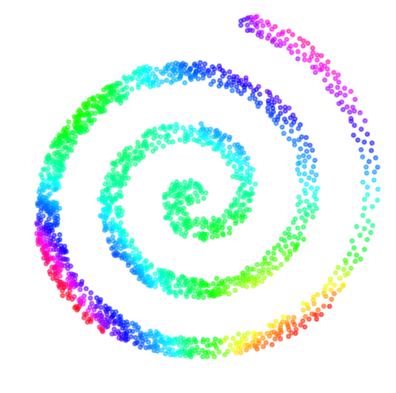

Supplement: Supplementary file 1 [file entropy-26-00679-s001.zip › dpvi_discrete-master/_tmp/demo_sd/iter8.png]

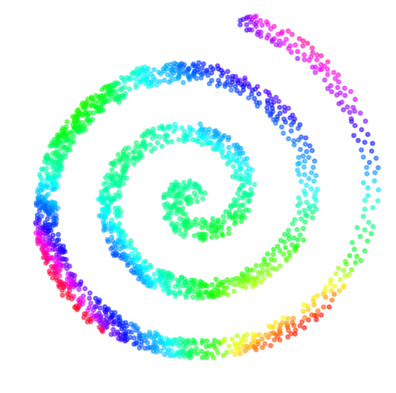

Supplement: Supplementary file 1 [file entropy-26-00679-s001.zip › dpvi_discrete-master/_tmp/demo_sd/iter9.png]

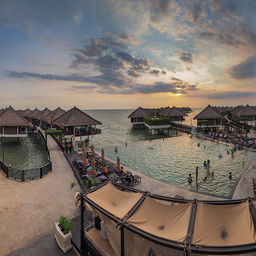

Supplement: Supplementary file 1 [file entropy-26-00679-s001.zip › dpvi_discrete-master/datasets/color_transfer/group1/source.jpg]

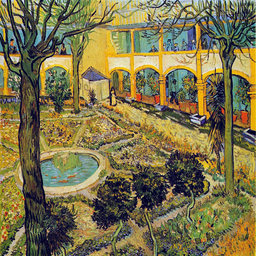

Supplement: Supplementary file 1 [file entropy-26-00679-s001.zip › dpvi_discrete-master/datasets/color_transfer/group1/target.jpg]

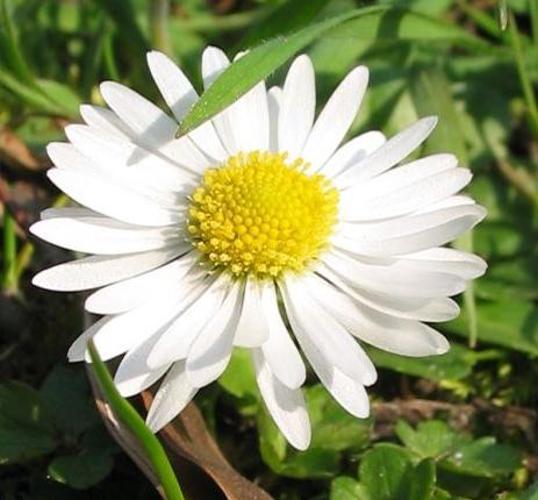

Supplement: Supplementary file 1 [file entropy-26-00679-s001.zip › dpvi_discrete-master/datasets/color_transfer/group2/source.jpg]

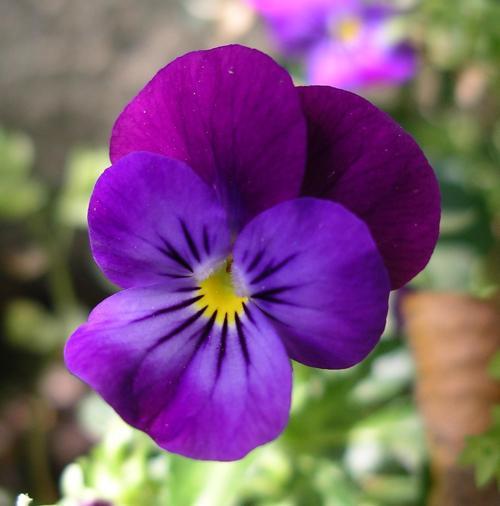

Supplement: Supplementary file 1 [file entropy-26-00679-s001.zip › dpvi_discrete-master/datasets/color_transfer/group2/target.jpg]

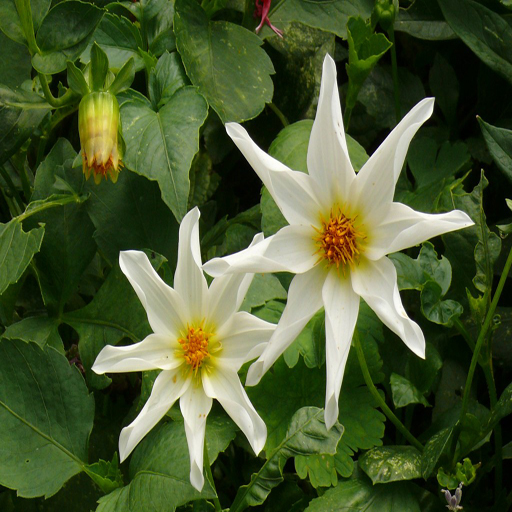

Supplement: Supplementary file 1 [file entropy-26-00679-s001.zip › dpvi_discrete-master/datasets/color_transfer/group3/source.jpg]

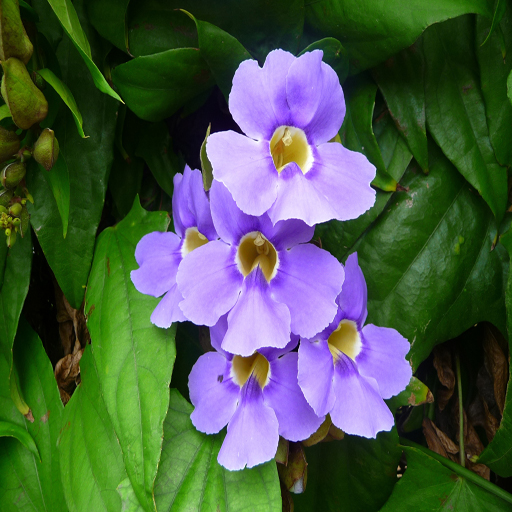

Supplement: Supplementary file 1 [file entropy-26-00679-s001.zip › dpvi_discrete-master/datasets/color_transfer/group3/target.jpg]

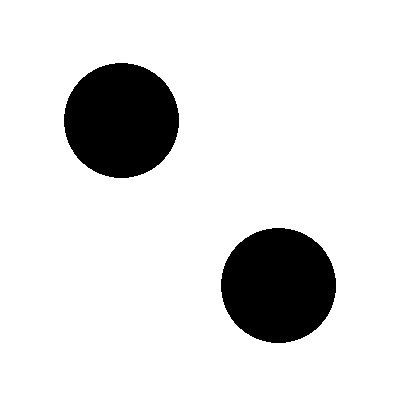

Supplement: Supplementary file 1 [file entropy-26-00679-s001.zip › dpvi_discrete-master/datasets/morphing/2disk.jpg]

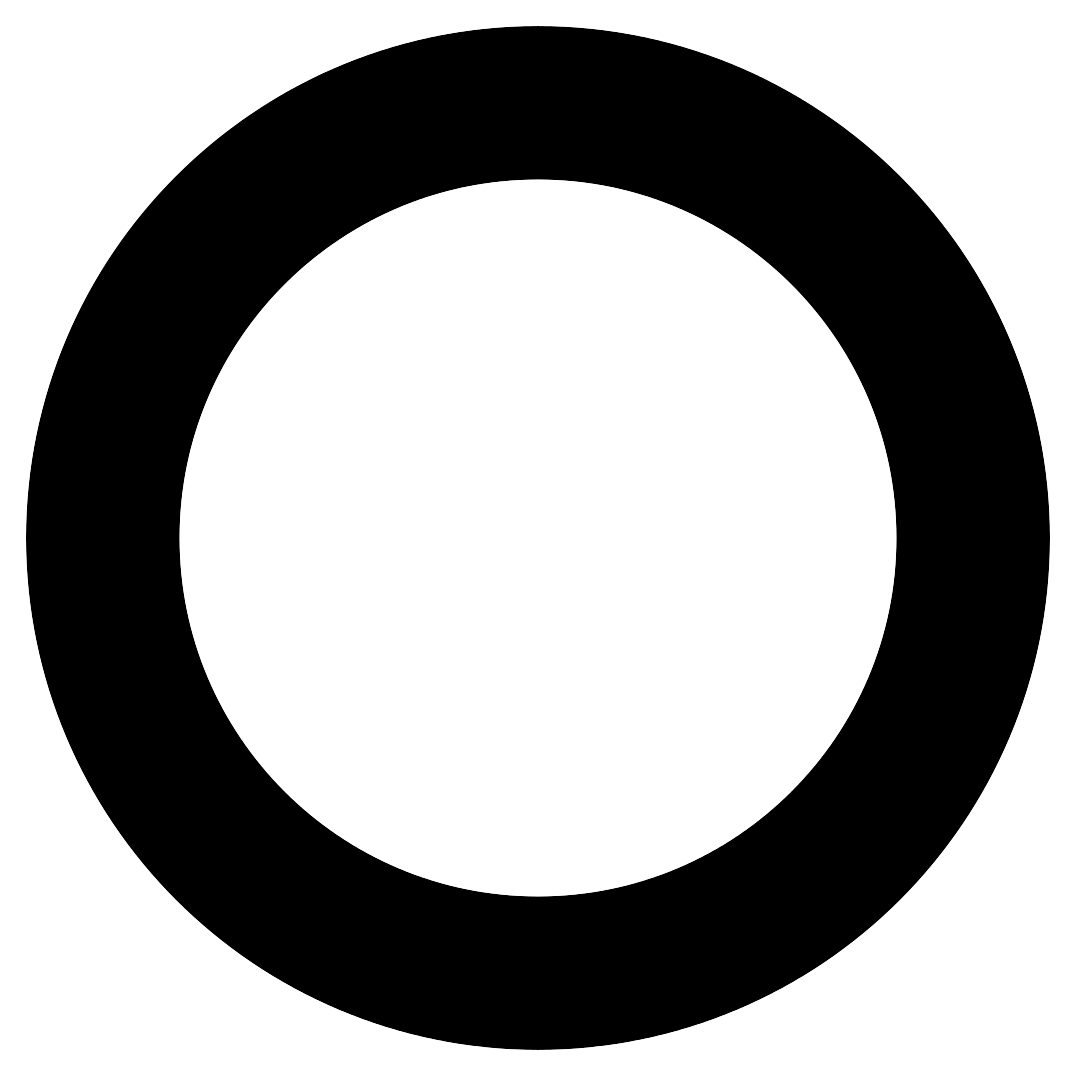

Supplement: Supplementary file 1 [file entropy-26-00679-s001.zip › dpvi_discrete-master/datasets/morphing/annulus.png]

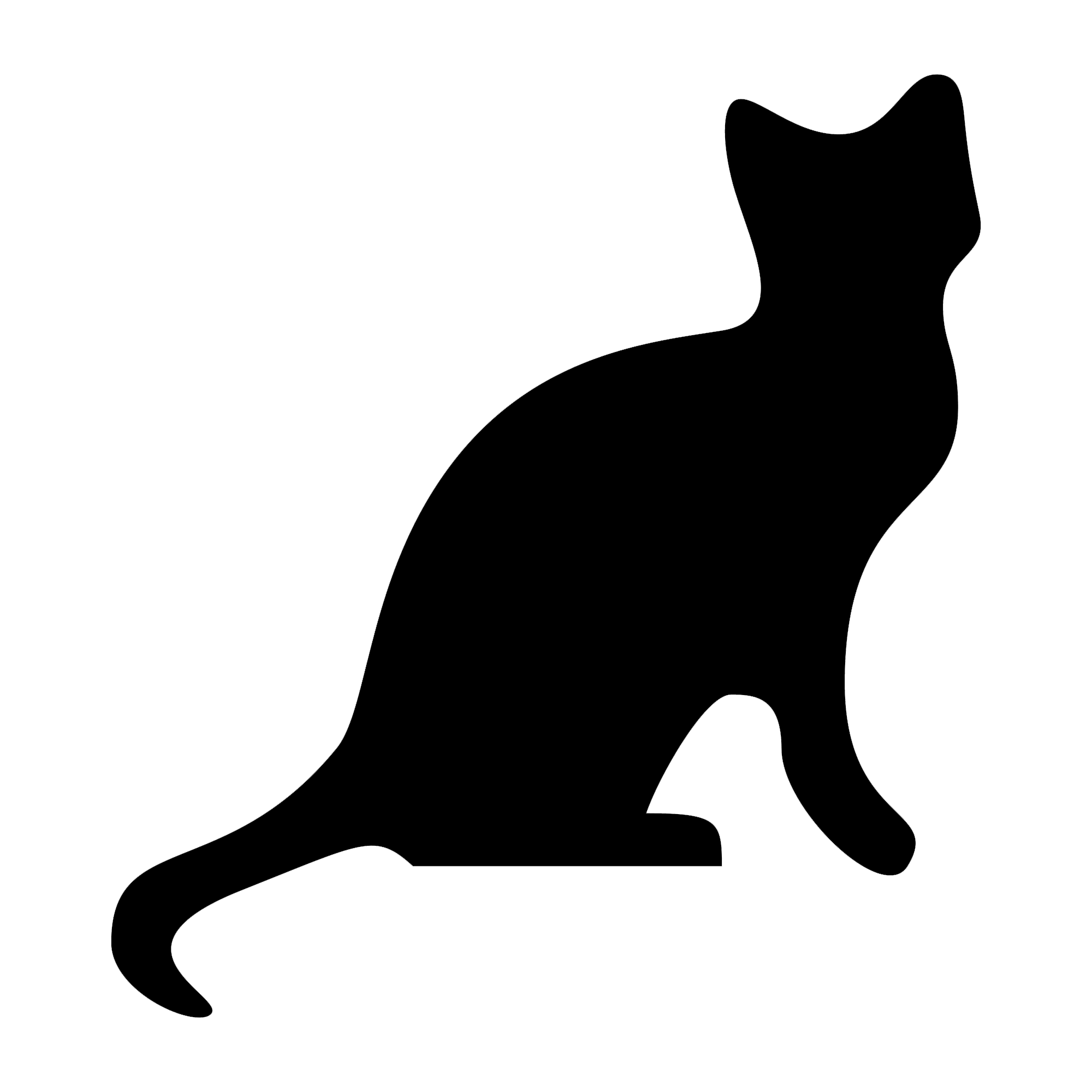

Supplement: Supplementary file 1 [file entropy-26-00679-s001.zip › dpvi_discrete-master/datasets/morphing/cat.png]

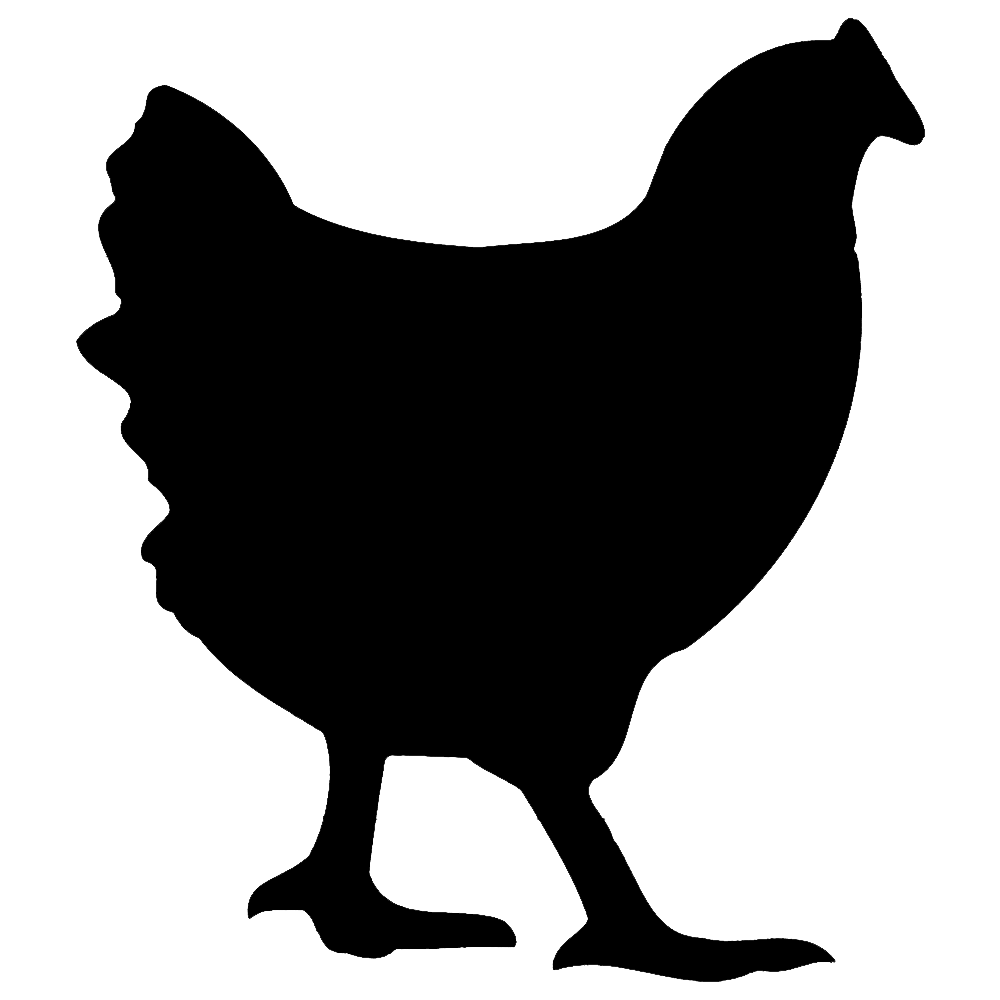

Supplement: Supplementary file 1 [file entropy-26-00679-s001.zip › dpvi_discrete-master/datasets/morphing/chicken2.png]

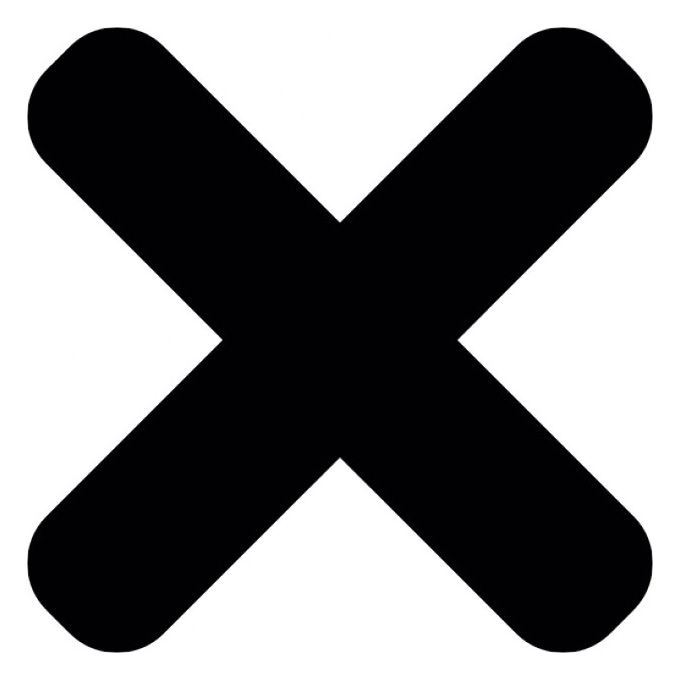

Supplement: Supplementary file 1 [file entropy-26-00679-s001.zip › dpvi_discrete-master/datasets/morphing/cross.jpg]

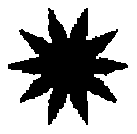

Supplement: Supplementary file 1 [file entropy-26-00679-s001.zip › dpvi_discrete-master/datasets/morphing/device.gif]

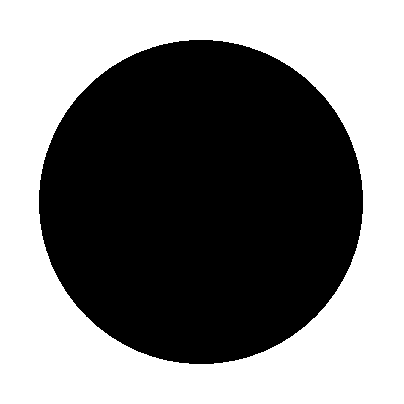

Supplement: Supplementary file 1 [file entropy-26-00679-s001.zip › dpvi_discrete-master/datasets/morphing/disk.png]

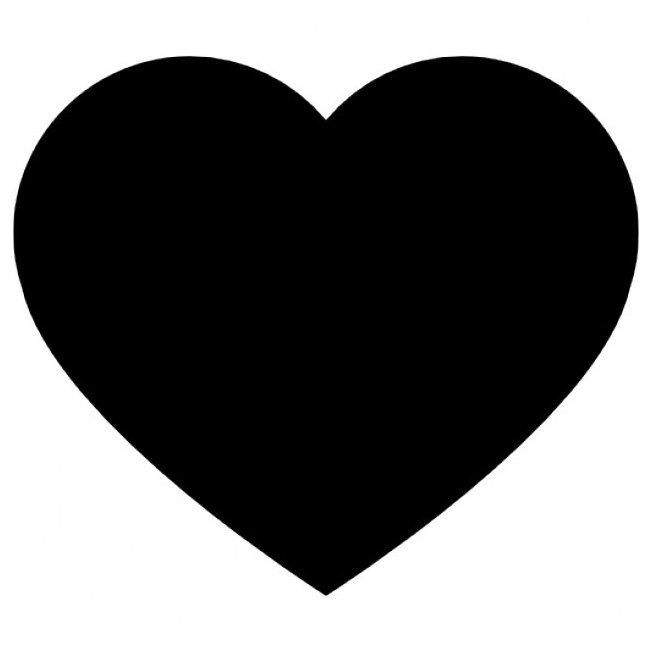

Supplement: Supplementary file 1 [file entropy-26-00679-s001.zip › dpvi_discrete-master/datasets/morphing/heart.jpg]

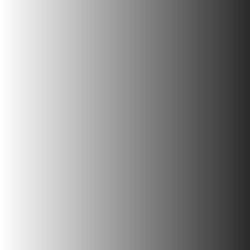

Supplement: Supplementary file 1 [file entropy-26-00679-s001.zip › dpvi_discrete-master/datasets/morphing/hgradient.png]

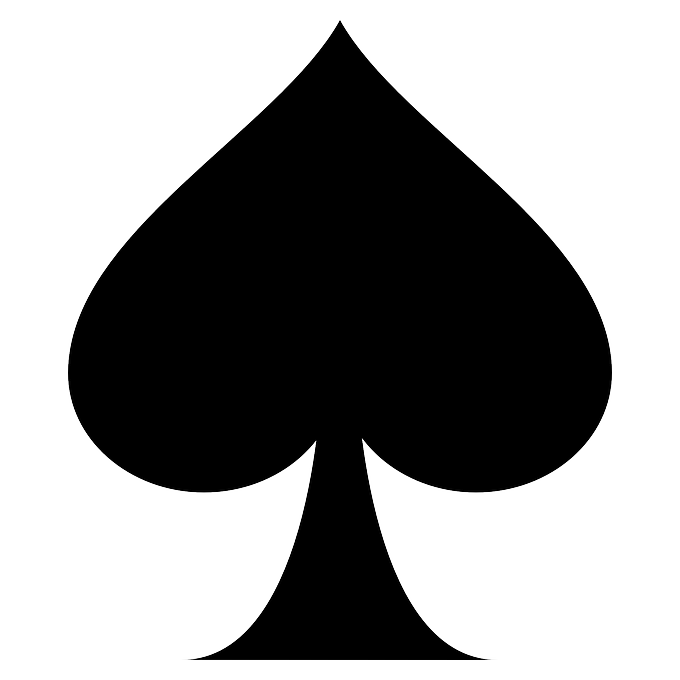

Supplement: Supplementary file 1 [file entropy-26-00679-s001.zip › dpvi_discrete-master/datasets/morphing/spade.png]

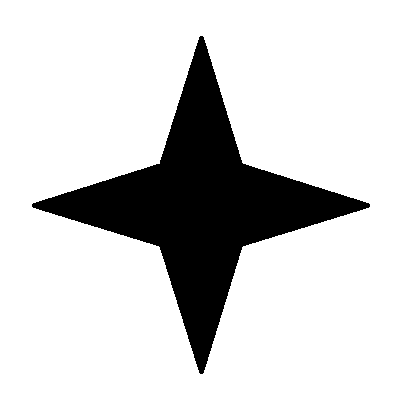

Supplement: Supplementary file 1 [file entropy-26-00679-s001.zip › dpvi_discrete-master/datasets/morphing/star4.png]

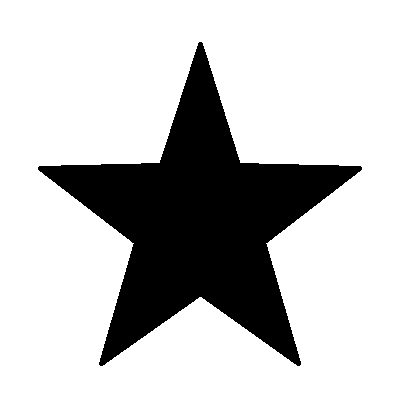

Supplement: Supplementary file 1 [file entropy-26-00679-s001.zip › dpvi_discrete-master/datasets/morphing/star5.png]

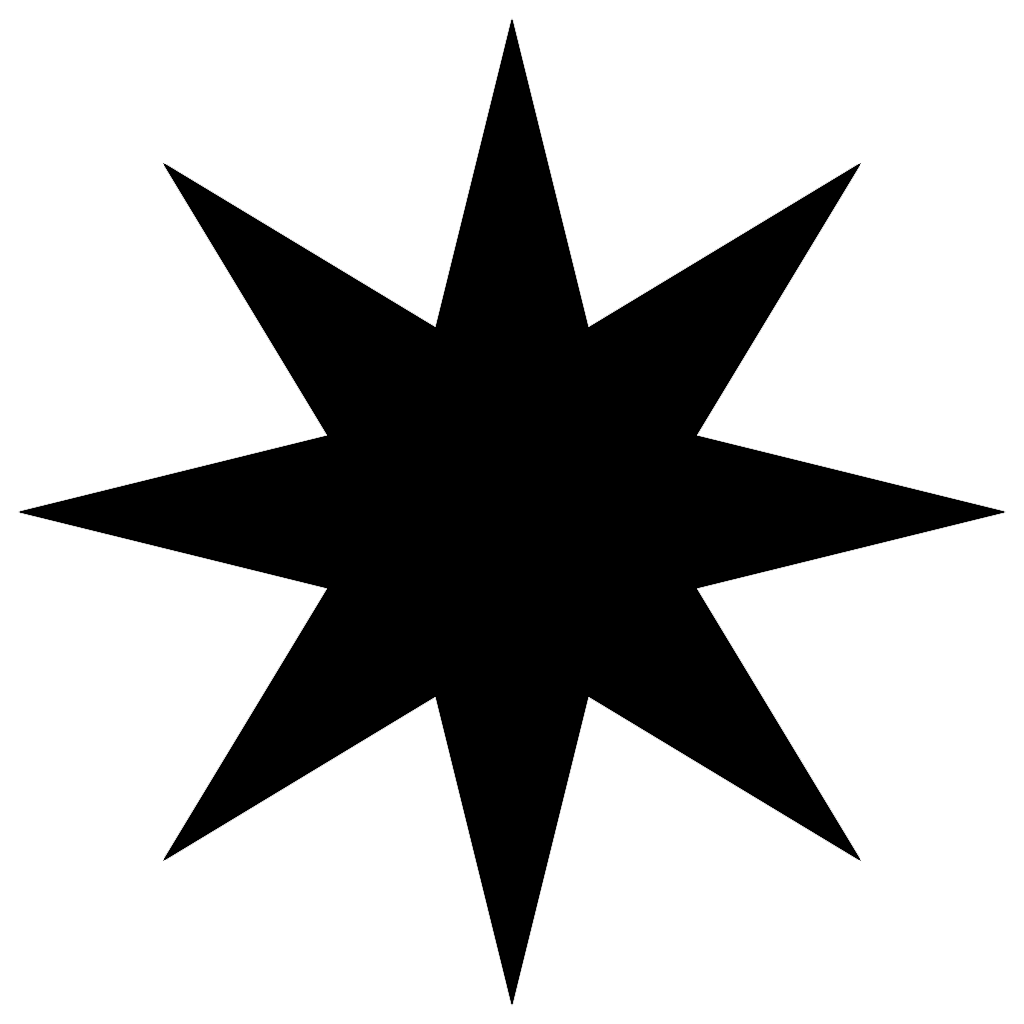

Supplement: Supplementary file 1 [file entropy-26-00679-s001.zip › dpvi_discrete-master/datasets/morphing/star8.png]

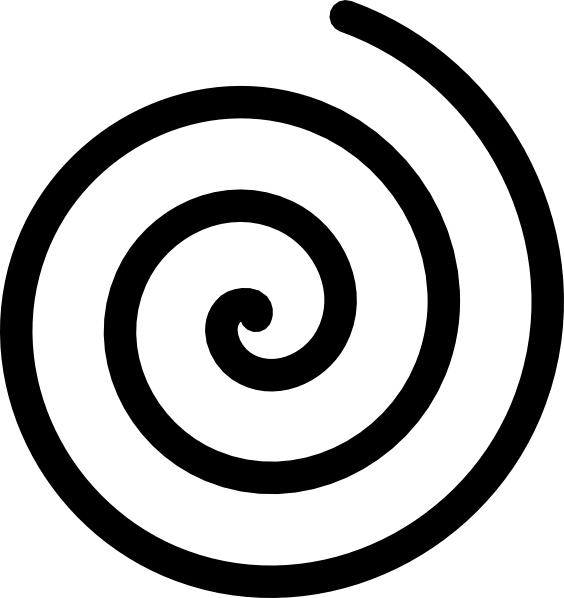

Supplement: Supplementary file 1 [file entropy-26-00679-s001.zip › dpvi_discrete-master/datasets/morphing/thinspiral.png]

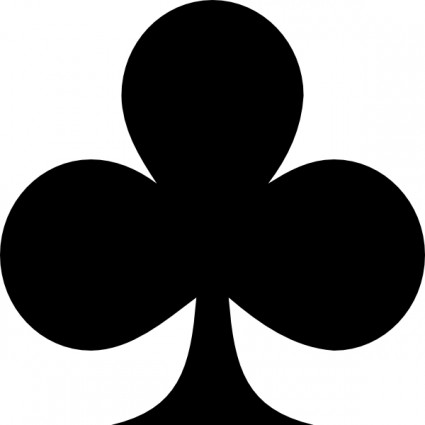

Supplement: Supplementary file 1 [file entropy-26-00679-s001.zip › dpvi_discrete-master/datasets/morphing/trefle.jpg]

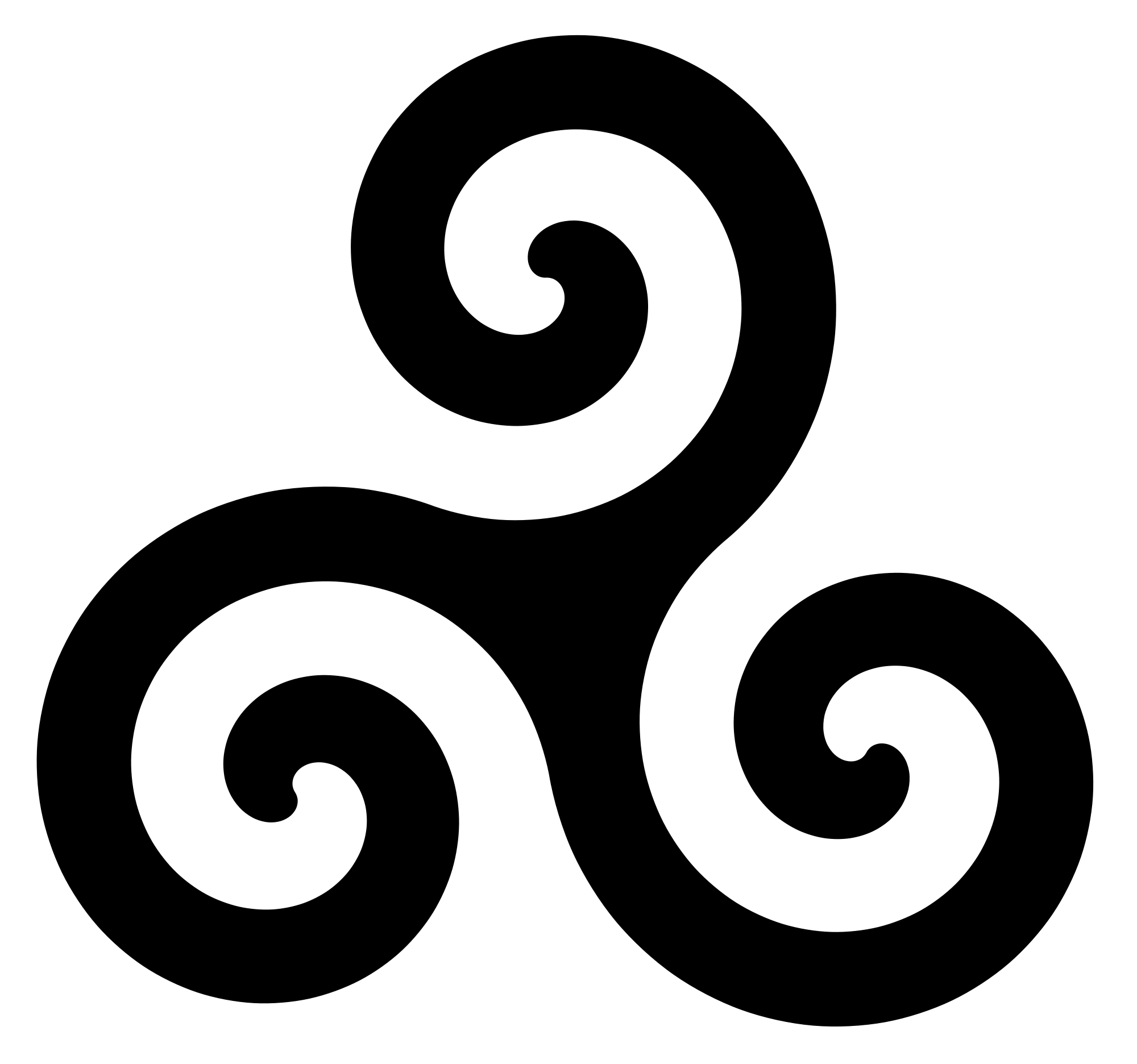

Supplement: Supplementary file 1 [file entropy-26-00679-s001.zip › dpvi_discrete-master/datasets/morphing/triskel.png]

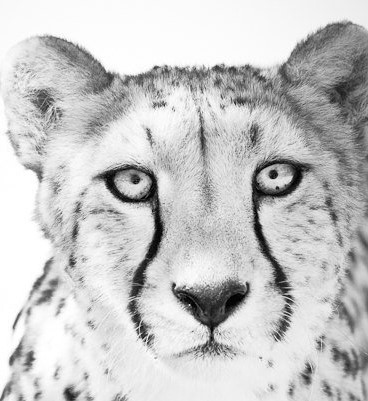

Supplement: Supplementary file 1 [file entropy-26-00679-s001.zip › dpvi_discrete-master/datasets/sketching/cheetah/target.jpg]

Sinkhorn Divergence

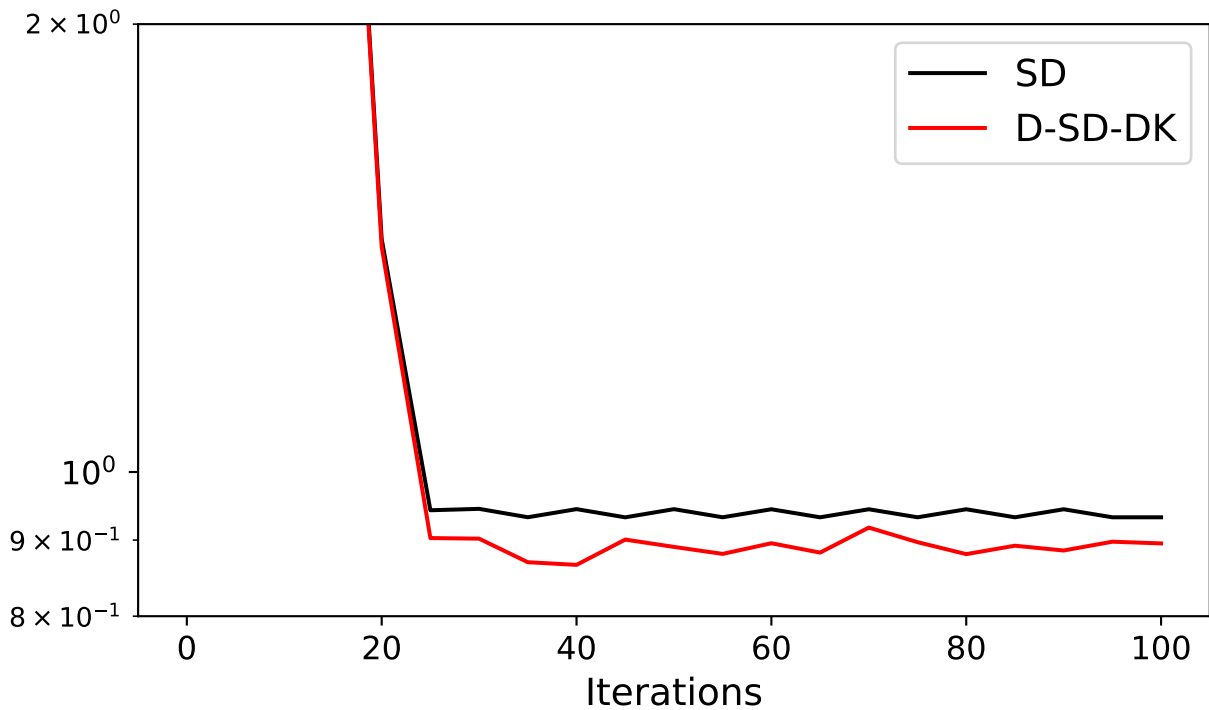

Supplement: Supplementary file 1 [file entropy-26-00679-s001.zip › dpvi_discrete-master/figures/flow/flow_iter.pdf]

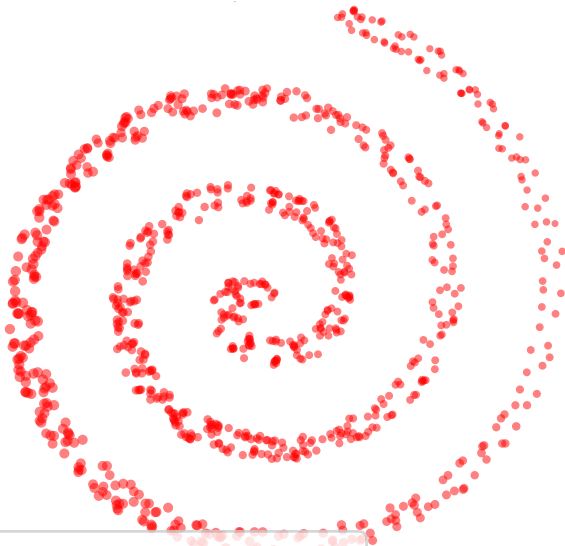

.

D-MMDF-CA

Supplement: Supplementary file 1 [file entropy-26-00679-s001.zip › dpvi_discrete-master/figures/morphing/num1000/D-MMDF-CA.pdf]

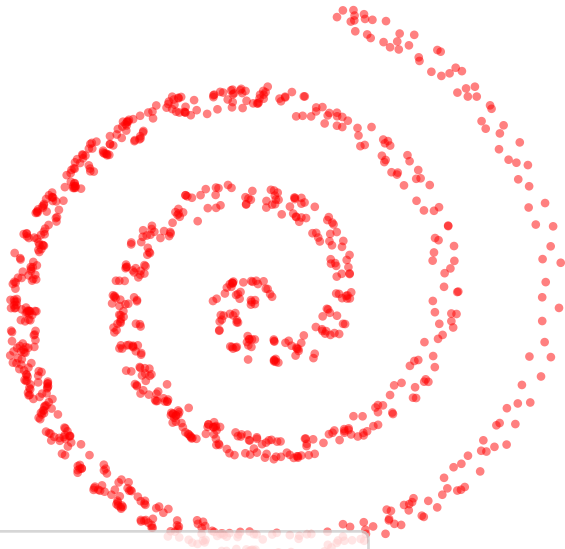

.

D-MMDF-DK

Supplement: Supplementary file 1 [file entropy-26-00679-s001.zip › dpvi_discrete-master/figures/morphing/num1000/D-MMDF-DK.pdf]

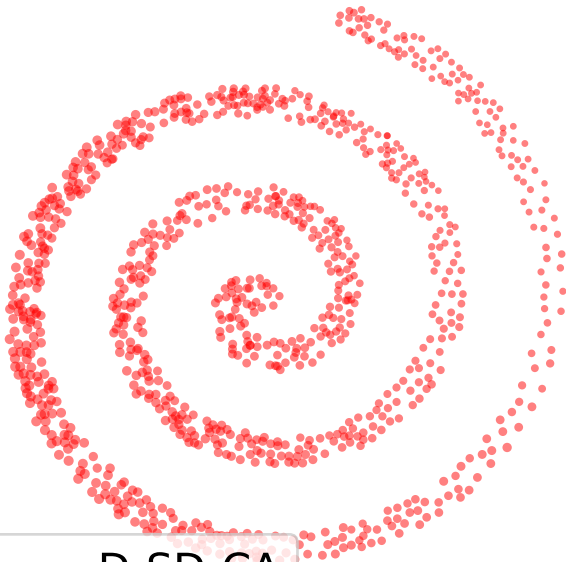

.

D-SD-CA

Supplement: Supplementary file 1 [file entropy-26-00679-s001.zip › dpvi_discrete-master/figures/morphing/num1000/D-SD-CA.pdf]

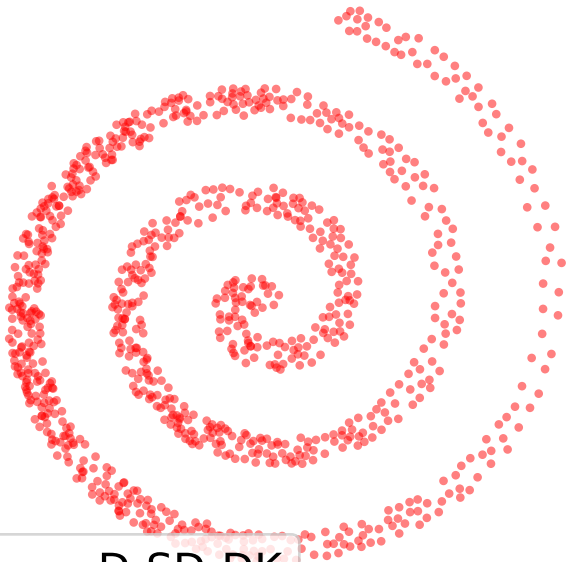

•

D-SD-DK

Supplement: Supplementary file 1 [file entropy-26-00679-s001.zip › dpvi_discrete-master/figures/morphing/num1000/D-SD-DK.pdf]

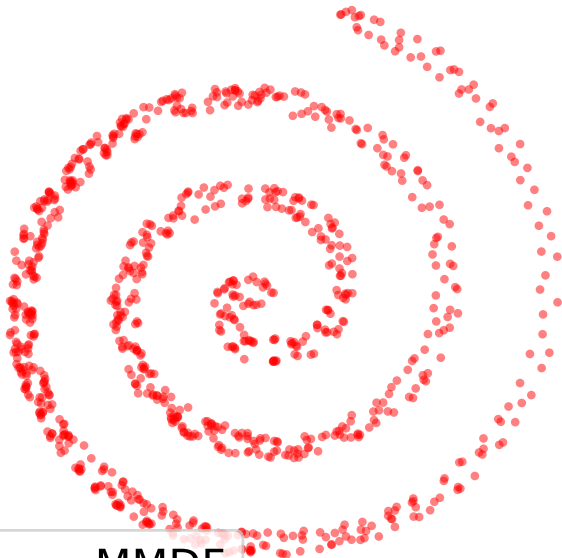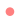

MMDF

Supplement: Supplementary file 1 [file entropy-26-00679-s001.zip › dpvi_discrete-master/figures/morphing/num1000/MMDF.pdf]

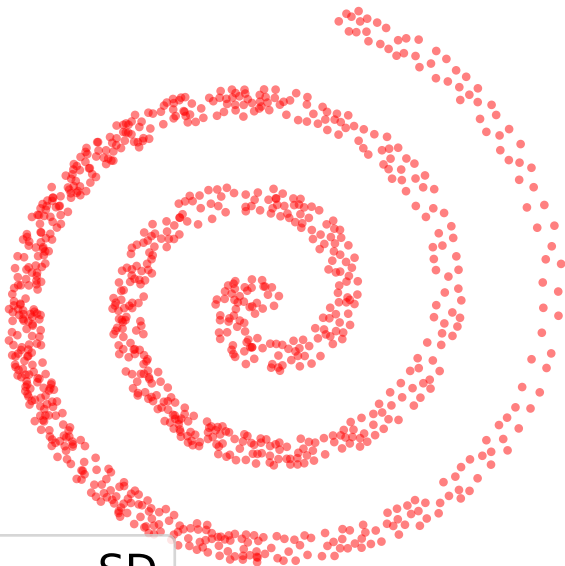

.

SD

Supplement: Supplementary file 1 [file entropy-26-00679-s001.zip › dpvi_discrete-master/figures/morphing/num1000/SD.pdf]

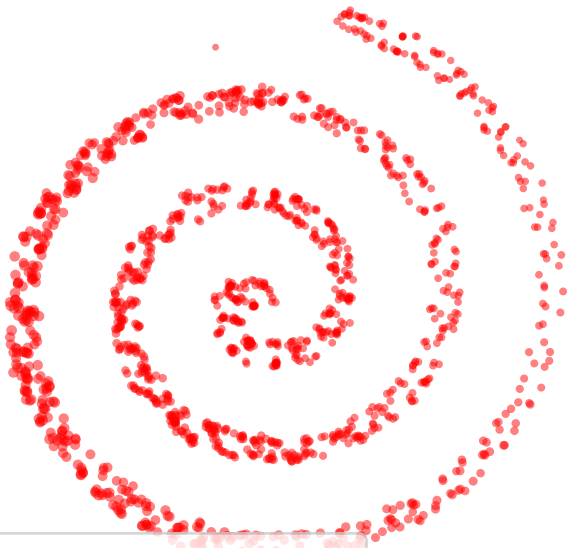

•

D-MMDF-CA

Supplement: Supplementary file 1 [file entropy-26-00679-s001.zip › dpvi_discrete-master/figures/morphing/num1500/D-MMDF-CA.pdf]

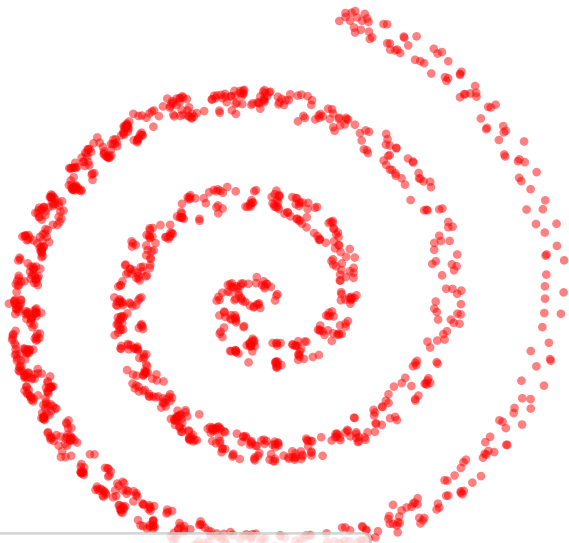

•

D-MMDF-DK

Supplement: Supplementary file 1 [file entropy-26-00679-s001.zip › dpvi_discrete-master/figures/morphing/num1500/D-MMDF-DK.pdf]

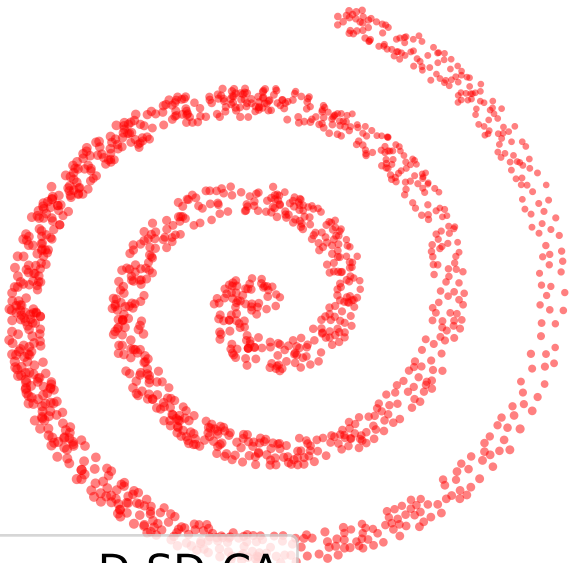

•

D-SD-CA

Supplement: Supplementary file 1 [file entropy-26-00679-s001.zip › dpvi_discrete-master/figures/morphing/num1500/D-SD-CA.pdf]

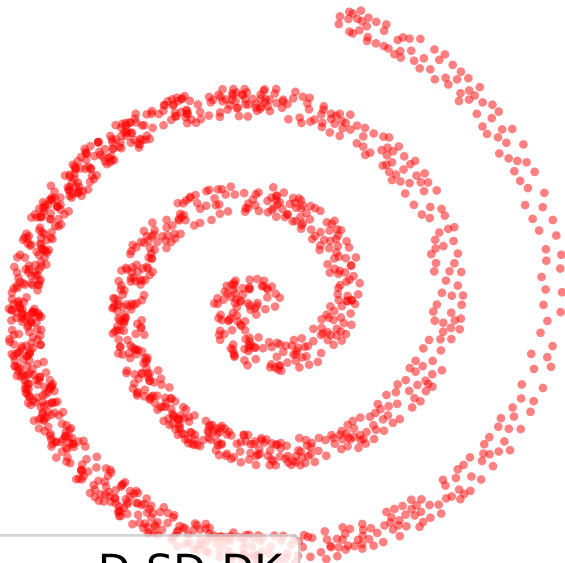

•

D-SD-DK

Supplement: Supplementary file 1 [file entropy-26-00679-s001.zip › dpvi_discrete-master/figures/morphing/num1500/D-SD-DK.pdf]

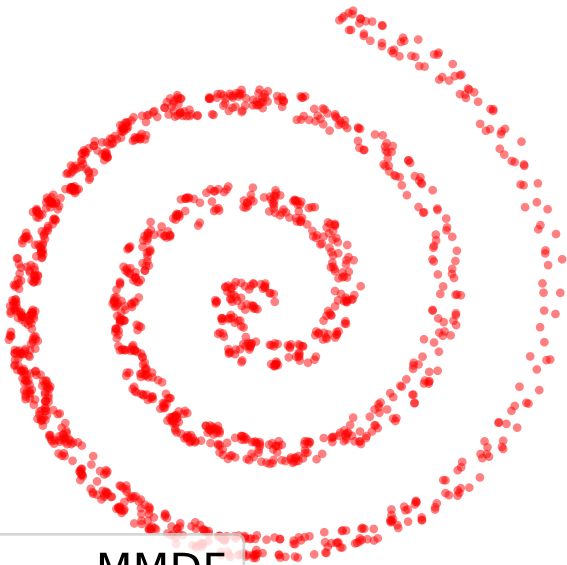

.

MMDF

Supplement: Supplementary file 1 [file entropy-26-00679-s001.zip › dpvi_discrete-master/figures/morphing/num1500/MMDF.pdf]

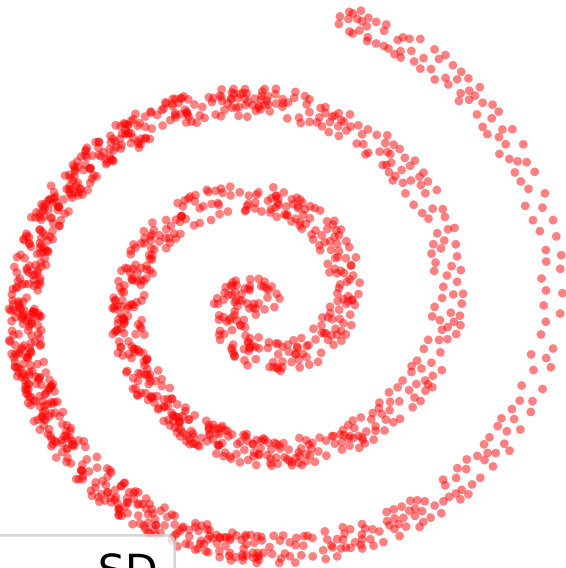

.

SD

Supplement: Supplementary file 1 [file entropy-26-00679-s001.zip › dpvi_discrete-master/figures/morphing/num1500/SD.pdf]

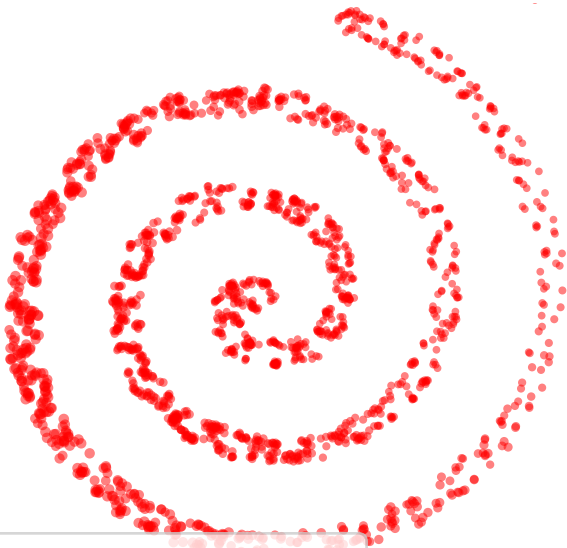

•

D-MMDF-CA

Supplement: Supplementary file 1 [file entropy-26-00679-s001.zip › dpvi_discrete-master/figures/morphing/num2000/D-MMDF-CA.pdf]

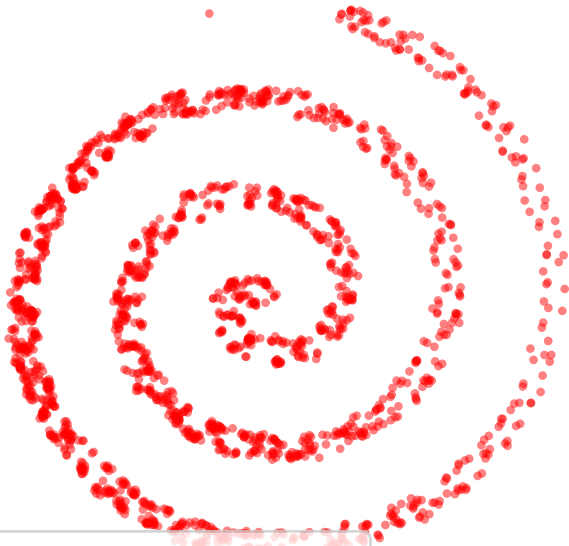

•

D-MMDF-DK

Supplement: Supplementary file 1 [file entropy-26-00679-s001.zip › dpvi_discrete-master/figures/morphing/num2000/D-MMDF-DK.pdf]

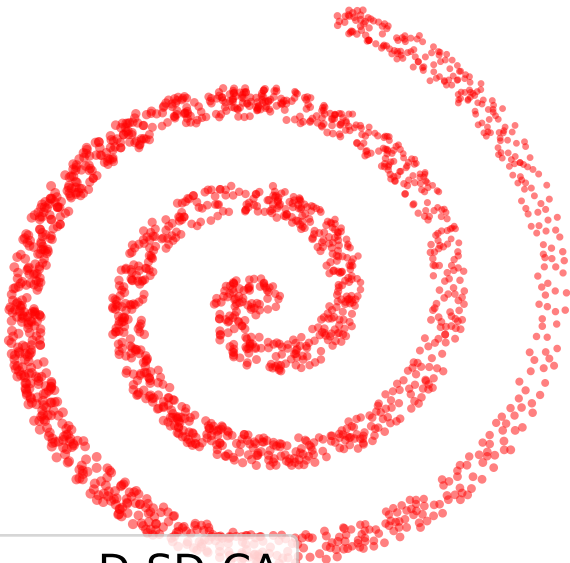

.

D-SD-CA

Supplement: Supplementary file 1 [file entropy-26-00679-s001.zip › dpvi_discrete-master/figures/morphing/num2000/D-SD-CA.pdf]

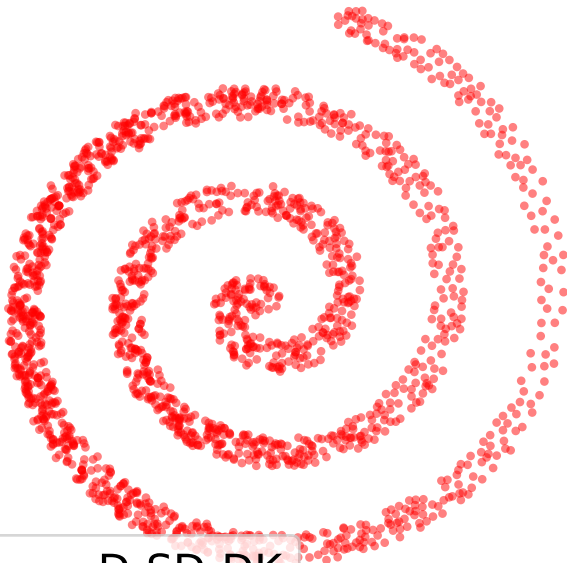

.

D-SD-DK

Supplement: Supplementary file 1 [file entropy-26-00679-s001.zip › dpvi_discrete-master/figures/morphing/num2000/D-SD-DK.pdf]

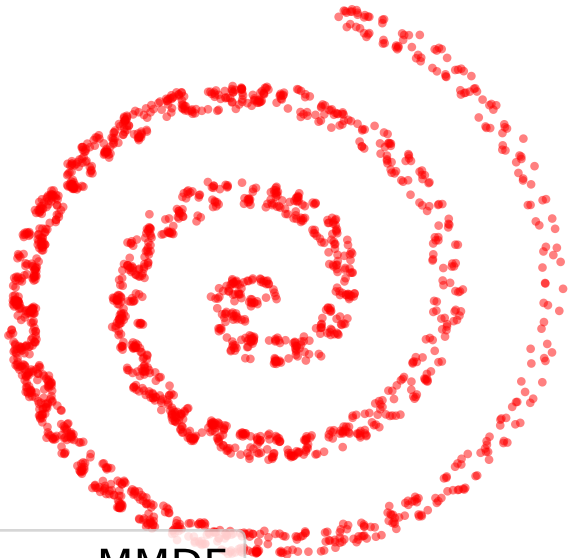

.

MMDF

Supplement: Supplementary file 1 [file entropy-26-00679-s001.zip › dpvi_discrete-master/figures/morphing/num2000/MMDF.pdf]

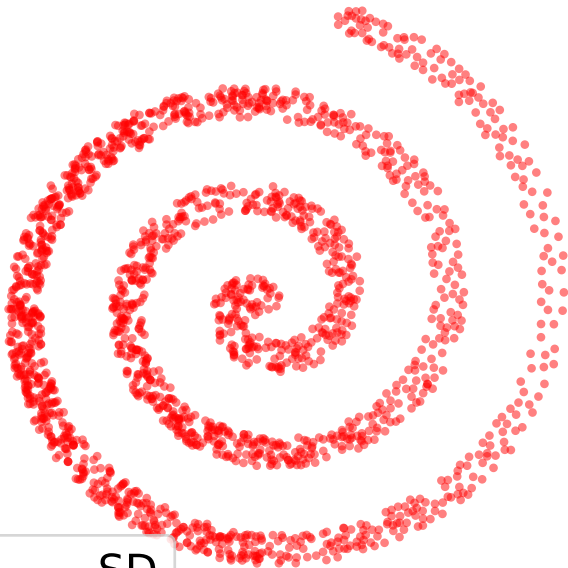

.

SD

Supplement: Supplementary file 1 [file entropy-26-00679-s001.zip › dpvi_discrete-master/figures/morphing/num2000/SD.pdf]

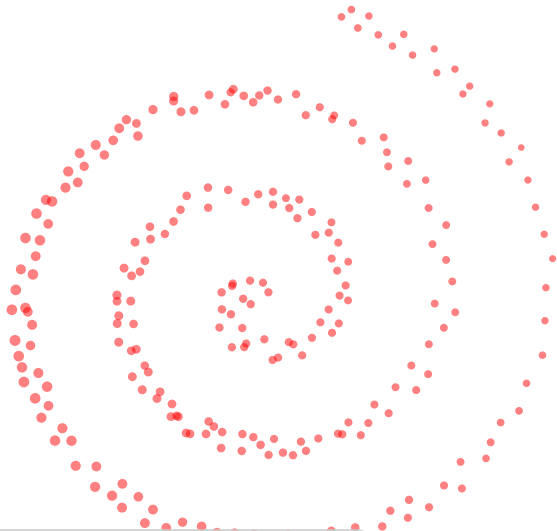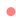

D-MMDF-CA

Supplement: Supplementary file 1 [file entropy-26-00679-s001.zip › dpvi_discrete-master/figures/morphing/num250/D-MMDF-CA.pdf]

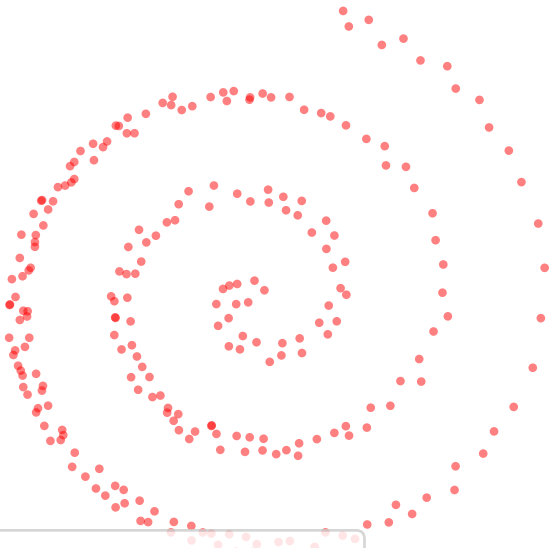

•

D-MMDF-DK

Supplement: Supplementary file 1 [file entropy-26-00679-s001.zip › dpvi_discrete-master/figures/morphing/num250/D-MMDF-DK.pdf]

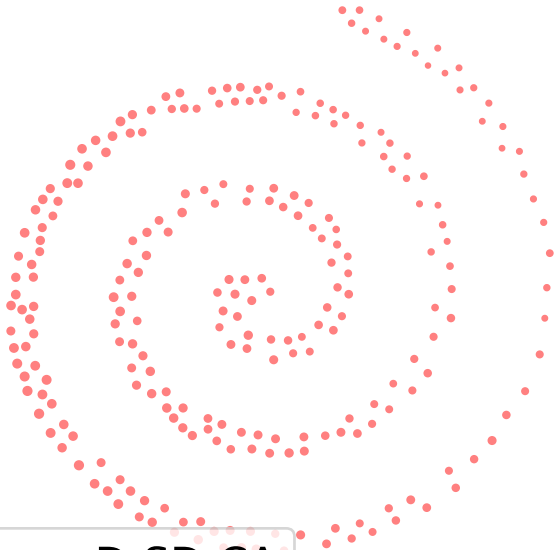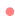

D-SD-CA

Supplement: Supplementary file 1 [file entropy-26-00679-s001.zip › dpvi_discrete-master/figures/morphing/num250/D-SD-CA.pdf]

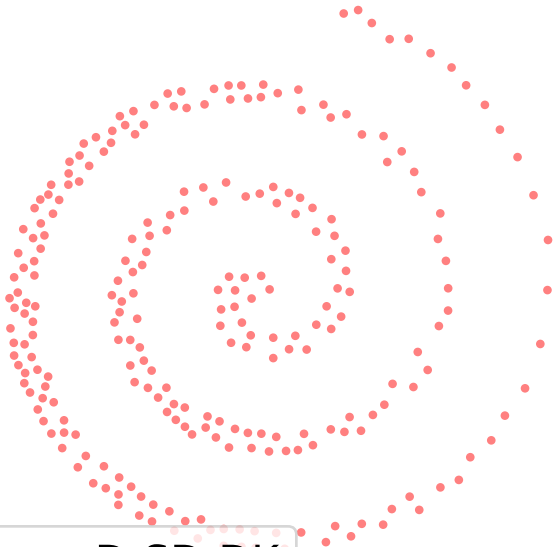

•

D-SD-DK

Supplement: Supplementary file 1 [file entropy-26-00679-s001.zip › dpvi_discrete-master/figures/morphing/num250/D-SD-DK.pdf]

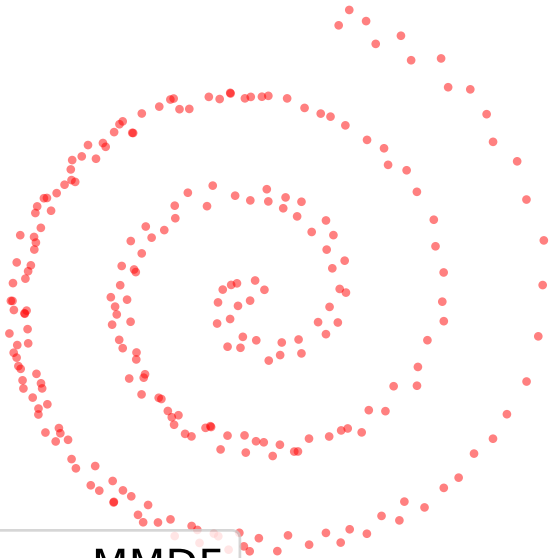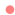

MMDF

Supplement: Supplementary file 1 [file entropy-26-00679-s001.zip › dpvi_discrete-master/figures/morphing/num250/MMDF.pdf]

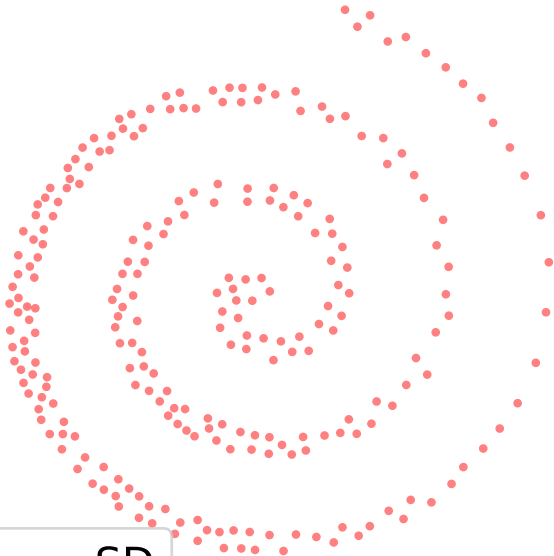

.

SD

Supplement: Supplementary file 1 [file entropy-26-00679-s001.zip › dpvi_discrete-master/figures/morphing/num250/SD.pdf]

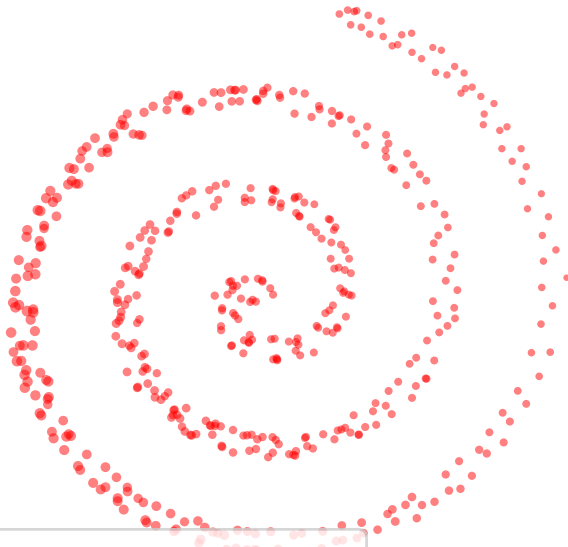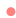

D-MMDF-CA

Supplement: Supplementary file 1 [file entropy-26-00679-s001.zip › dpvi_discrete-master/figures/morphing/num500/D-MMDF-CA.pdf]

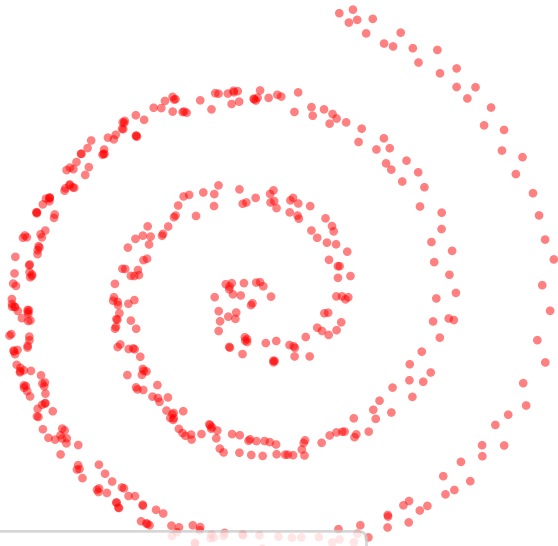

.

D-MMDF-DK

Supplement: Supplementary file 1 [file entropy-26-00679-s001.zip › dpvi_discrete-master/figures/morphing/num500/D-MMDF-DK.pdf]

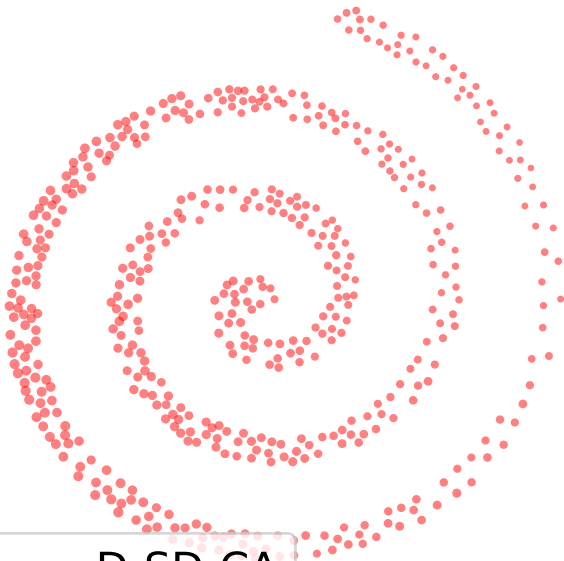

.

D-SD-CA

Supplement: Supplementary file 1 [file entropy-26-00679-s001.zip › dpvi_discrete-master/figures/morphing/num500/D-SD-CA.pdf]

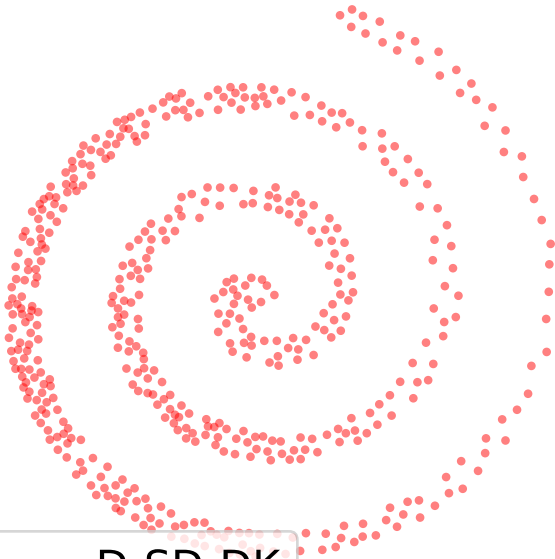

•

D-SD-DK

Supplement: Supplementary file 1 [file entropy-26-00679-s001.zip › dpvi_discrete-master/figures/morphing/num500/D-SD-DK.pdf]

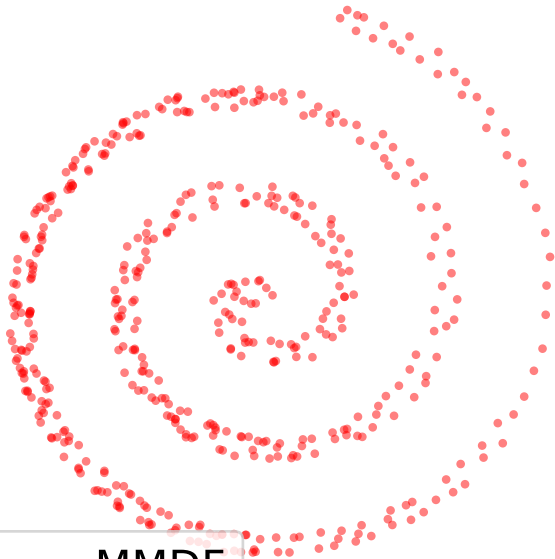

.

MMDF

Supplement: Supplementary file 1 [file entropy-26-00679-s001.zip › dpvi_discrete-master/figures/morphing/num500/MMDF.pdf]

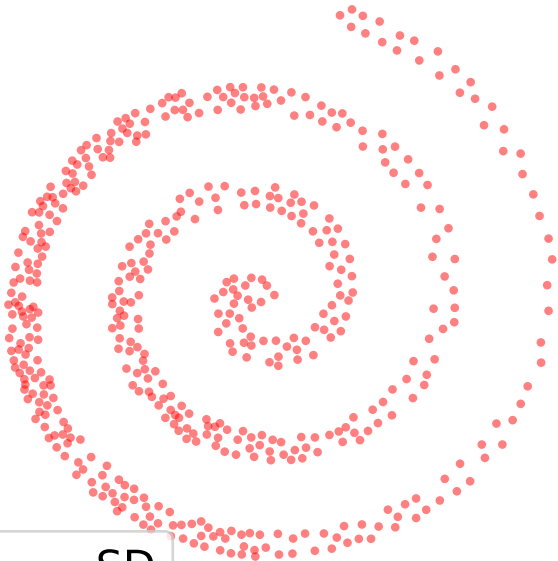

.

SD

Supplement: Supplementary file 1 [file entropy-26-00679-s001.zip › dpvi_discrete-master/figures/morphing/num500/SD.pdf]

2-Wasserstein Distance

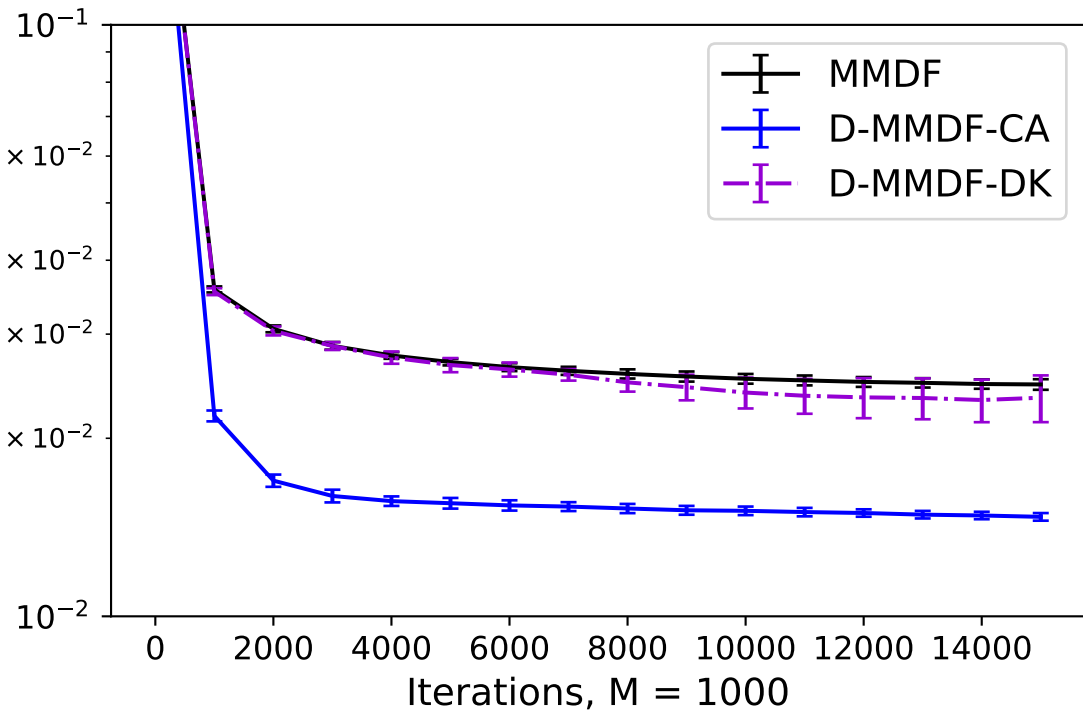

2-Wasserstein Distance

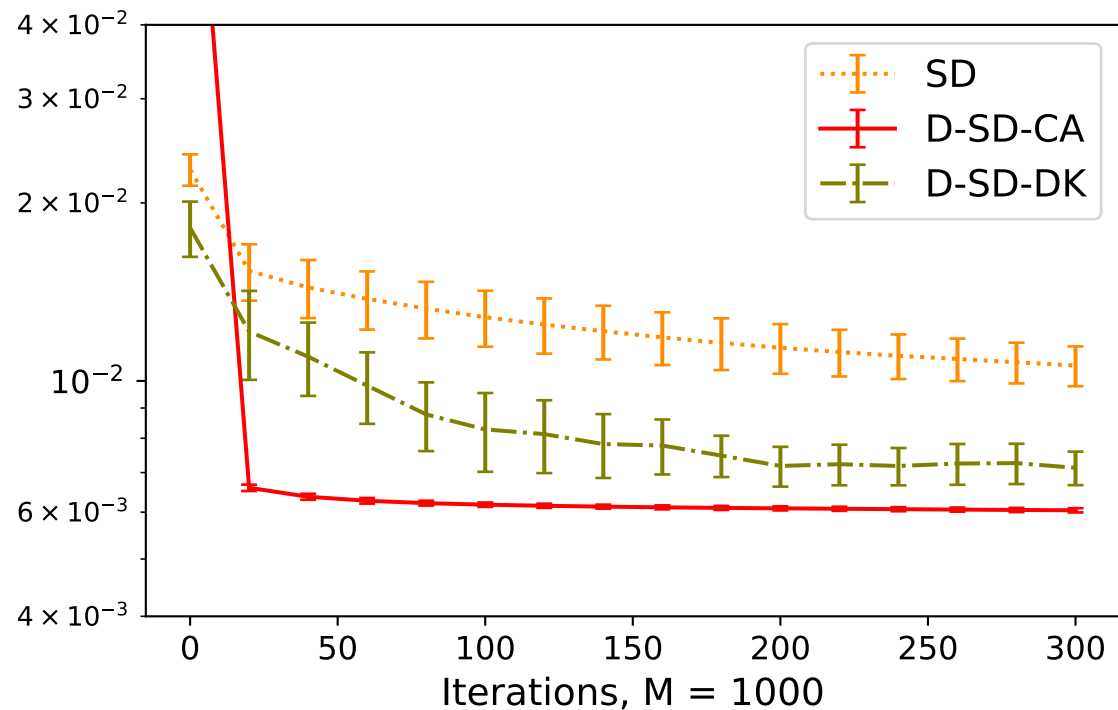

Supplement: Supplementary file 1 [file entropy-26-00679-s001.zip › dpvi_discrete-master/figures/morphing/sketching_1000_iter.pdf]

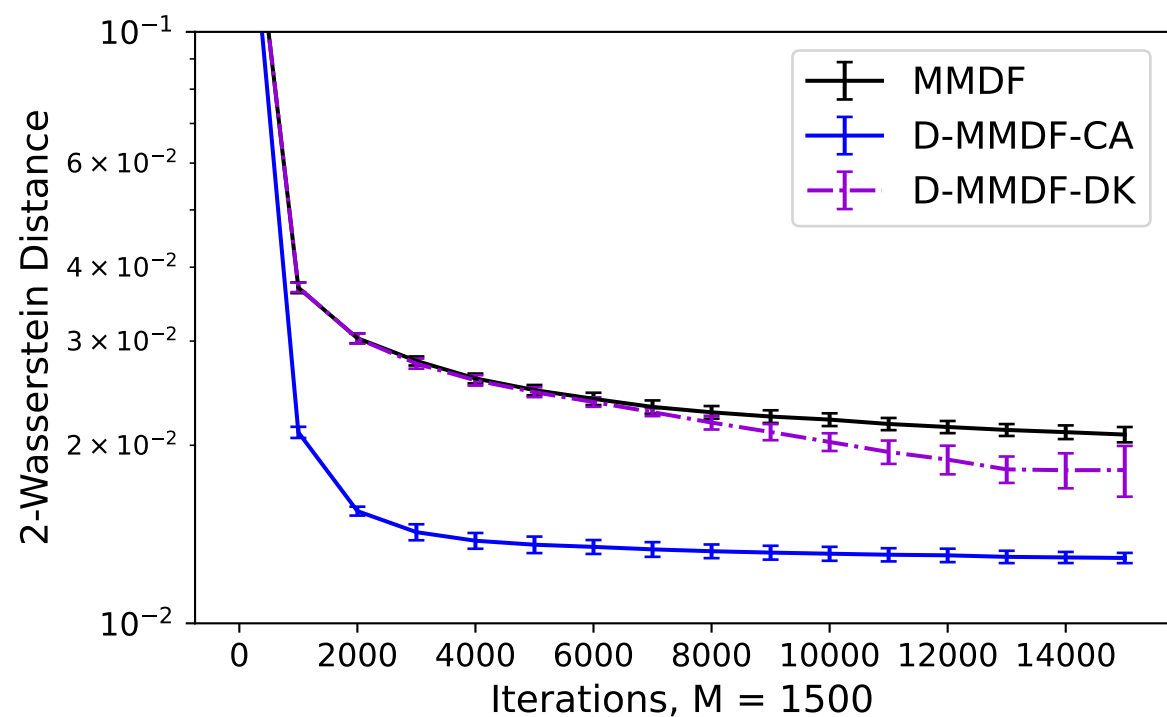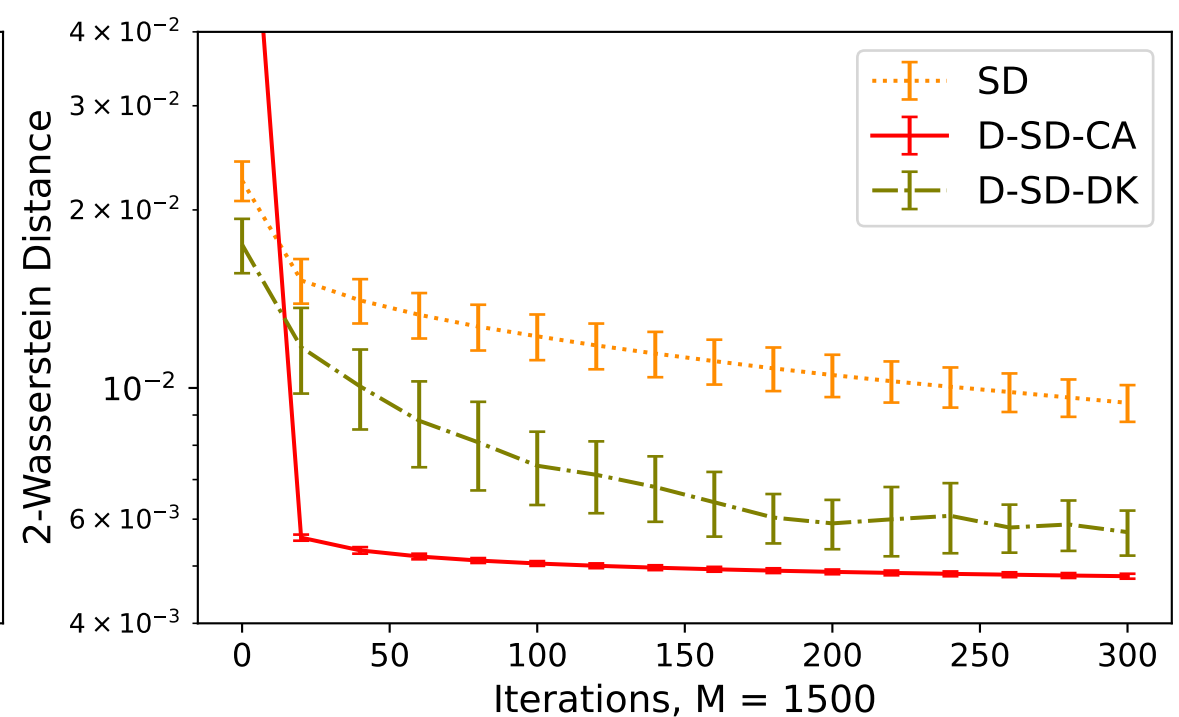

Supplement: Supplementary file 1 [file entropy-26-00679-s001.zip › dpvi_discrete-master/figures/morphing/sketching_1500_iter.pdf]

2-Wasserstein Distance

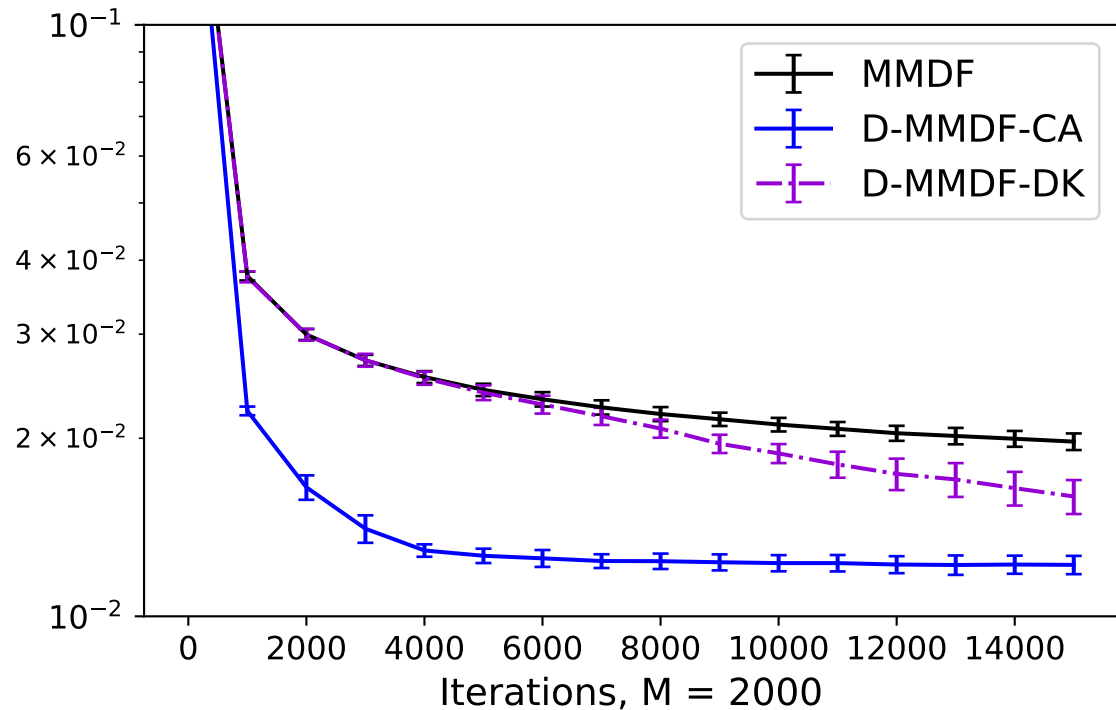

2-Wasserstein Distance

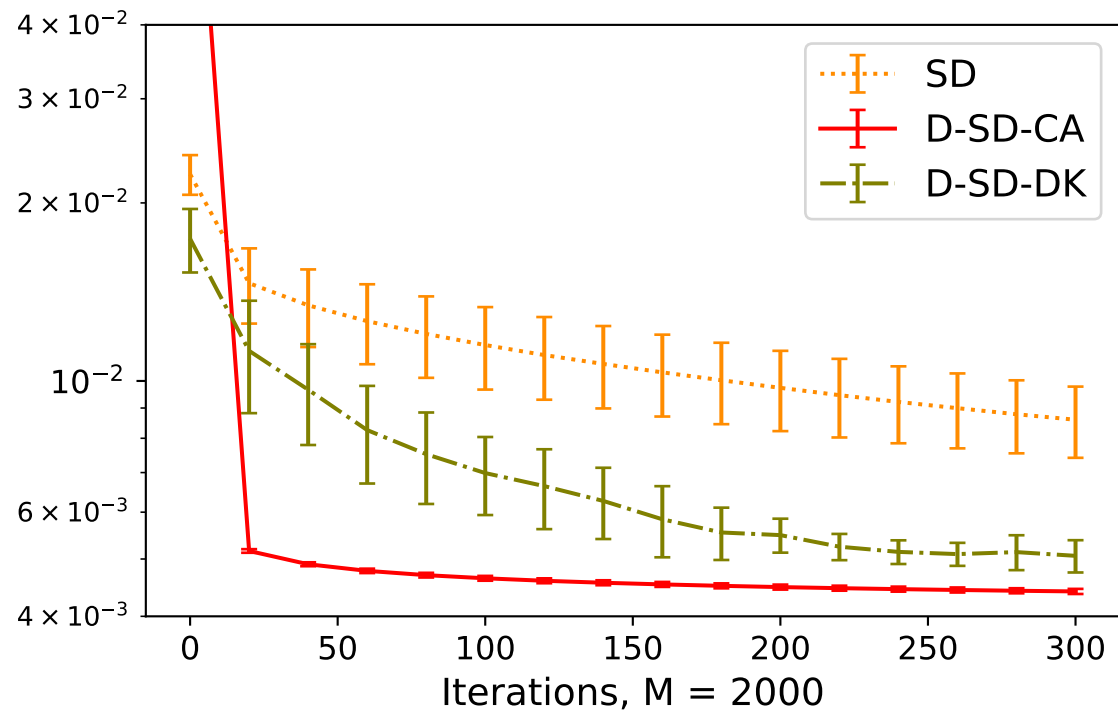

Supplement: Supplementary file 1 [file entropy-26-00679-s001.zip › dpvi_discrete-master/figures/morphing/sketching_2000_iter.pdf]

2-Wasserstein Distance

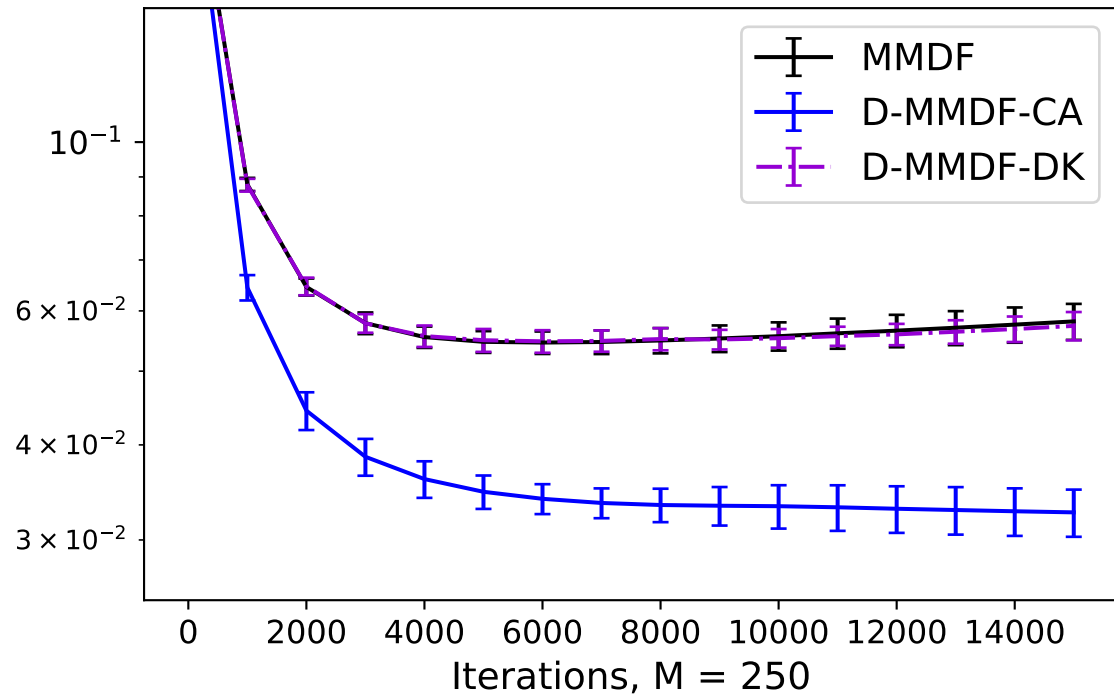

2-Wasserstein Distance

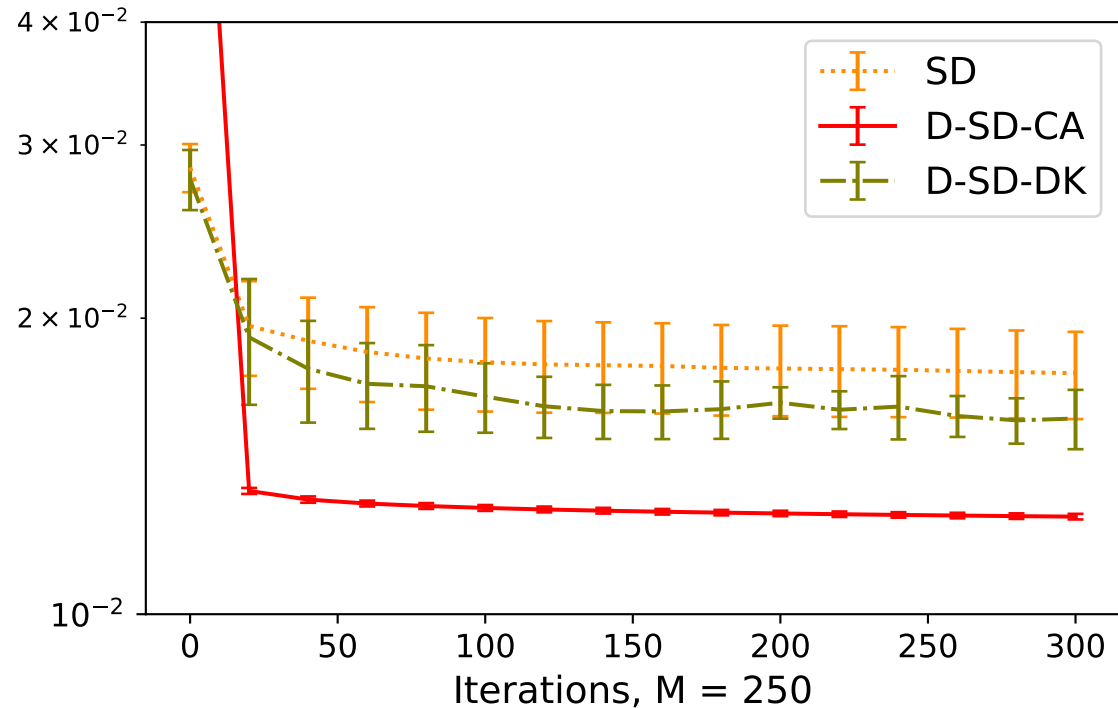

Supplement: Supplementary file 1 [file entropy-26-00679-s001.zip › dpvi_discrete-master/figures/morphing/sketching_250_iter.pdf]

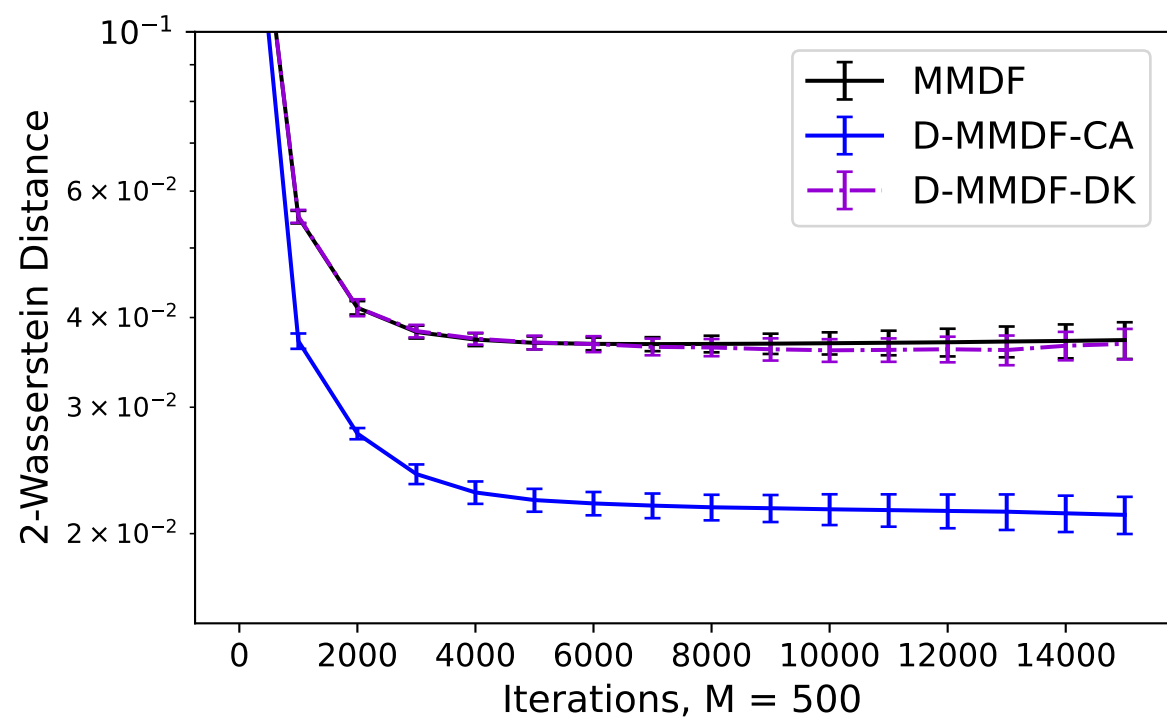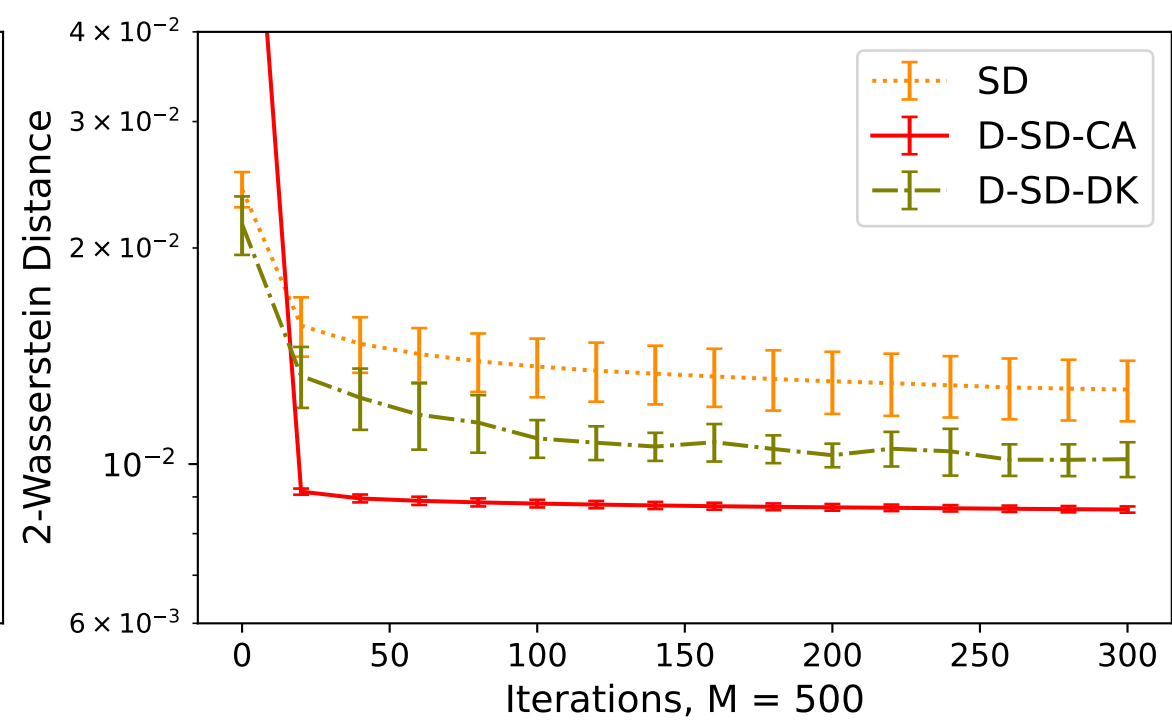

Supplement: Supplementary file 1 [file entropy-26-00679-s001.zip › dpvi_discrete-master/figures/morphing/sketching_500_iter.pdf]

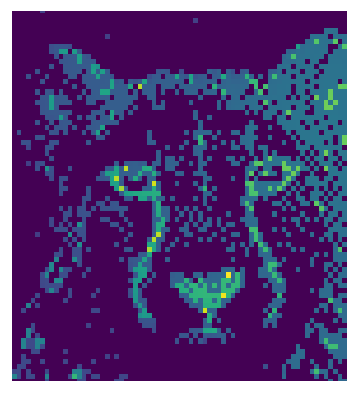

Supplement: Supplementary file 1 [file entropy-26-00679-s001.zip › dpvi_discrete-master/figures/sketching/D-MMDF-CA.png]

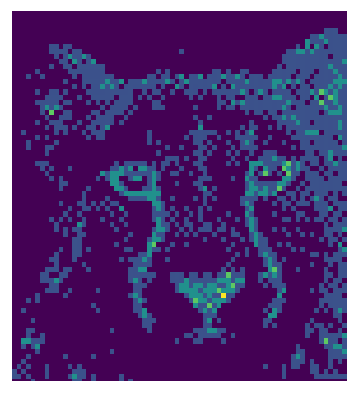

Supplement: Supplementary file 1 [file entropy-26-00679-s001.zip › dpvi_discrete-master/figures/sketching/D-MMDF-DK.png]

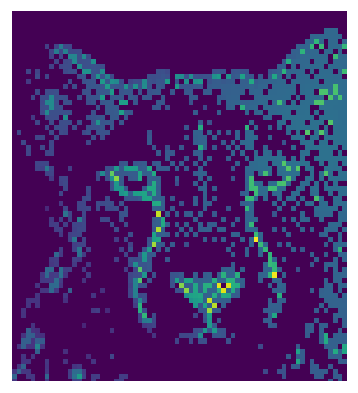

Supplement: Supplementary file 1 [file entropy-26-00679-s001.zip › dpvi_discrete-master/figures/sketching/D-SD-CA.png]

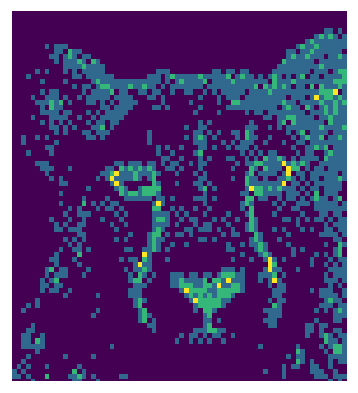

Supplement: Supplementary file 1 [file entropy-26-00679-s001.zip › dpvi_discrete-master/figures/sketching/D-SD-DK.png]

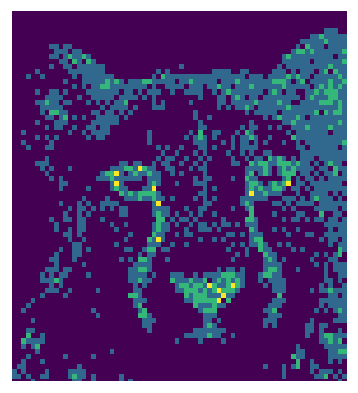

Supplement: Supplementary file 1 [file entropy-26-00679-s001.zip › dpvi_discrete-master/figures/sketching/MMDF.png]

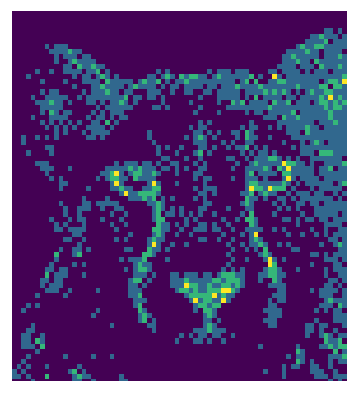

Supplement: Supplementary file 1 [file entropy-26-00679-s001.zip › dpvi_discrete-master/figures/sketching/SD.png]

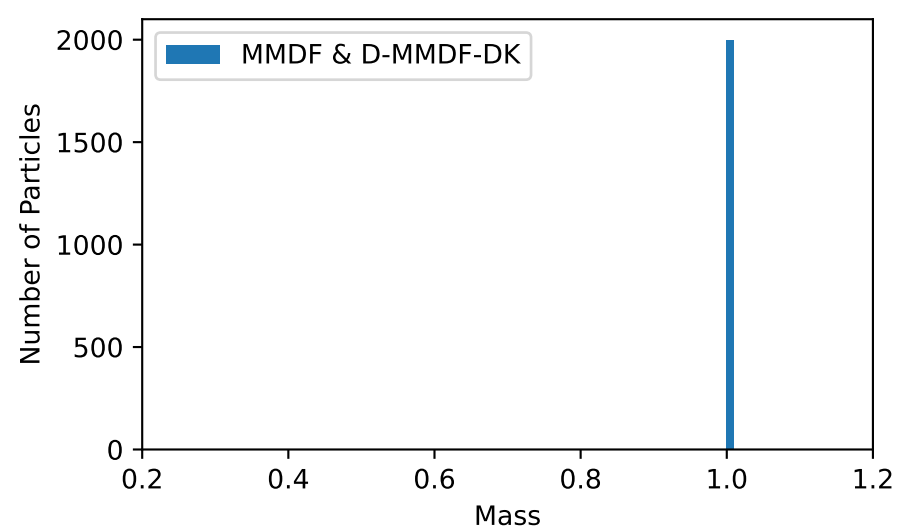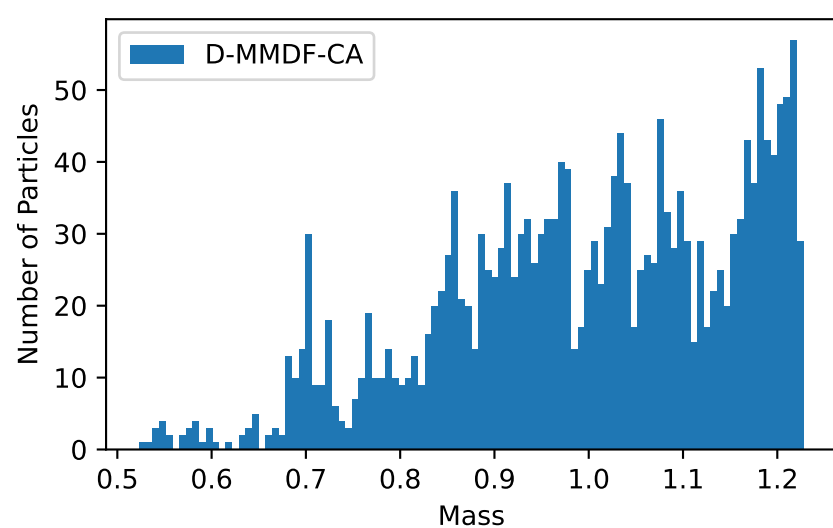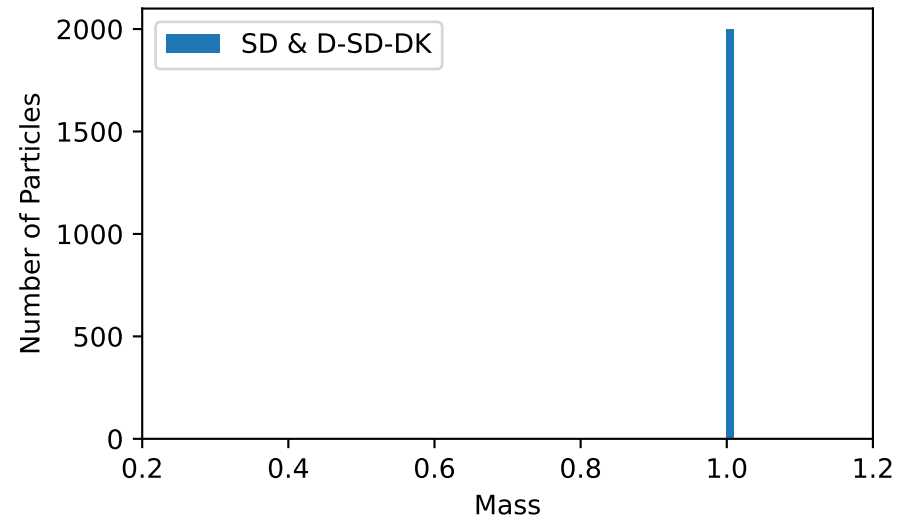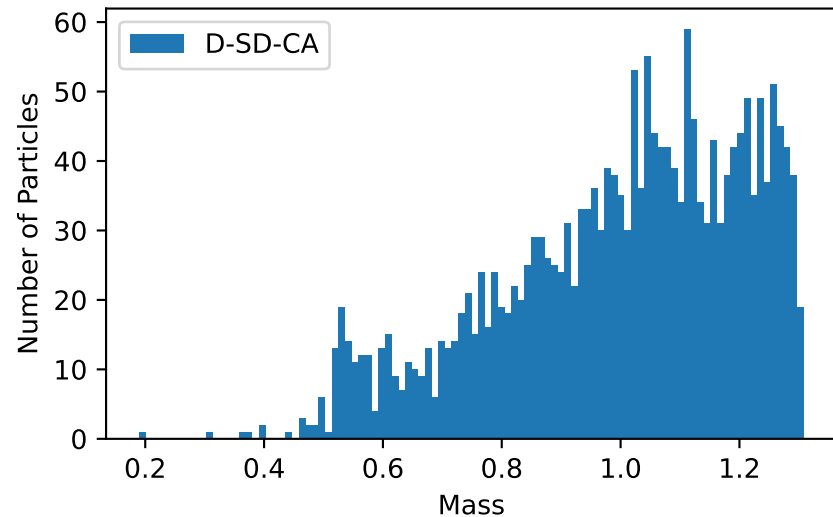

Supplement: Supplementary file 1 [file entropy-26-00679-s001.zip › dpvi_discrete-master/figures/sketching/histogram.pdf]

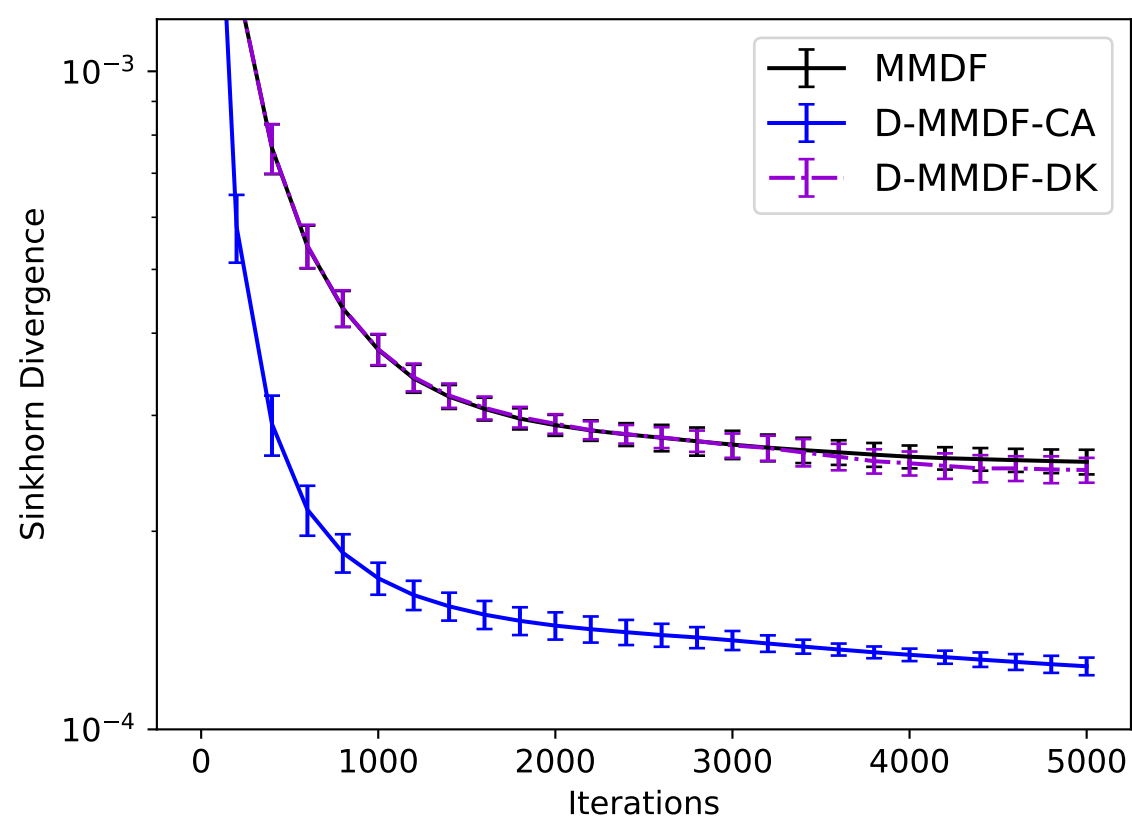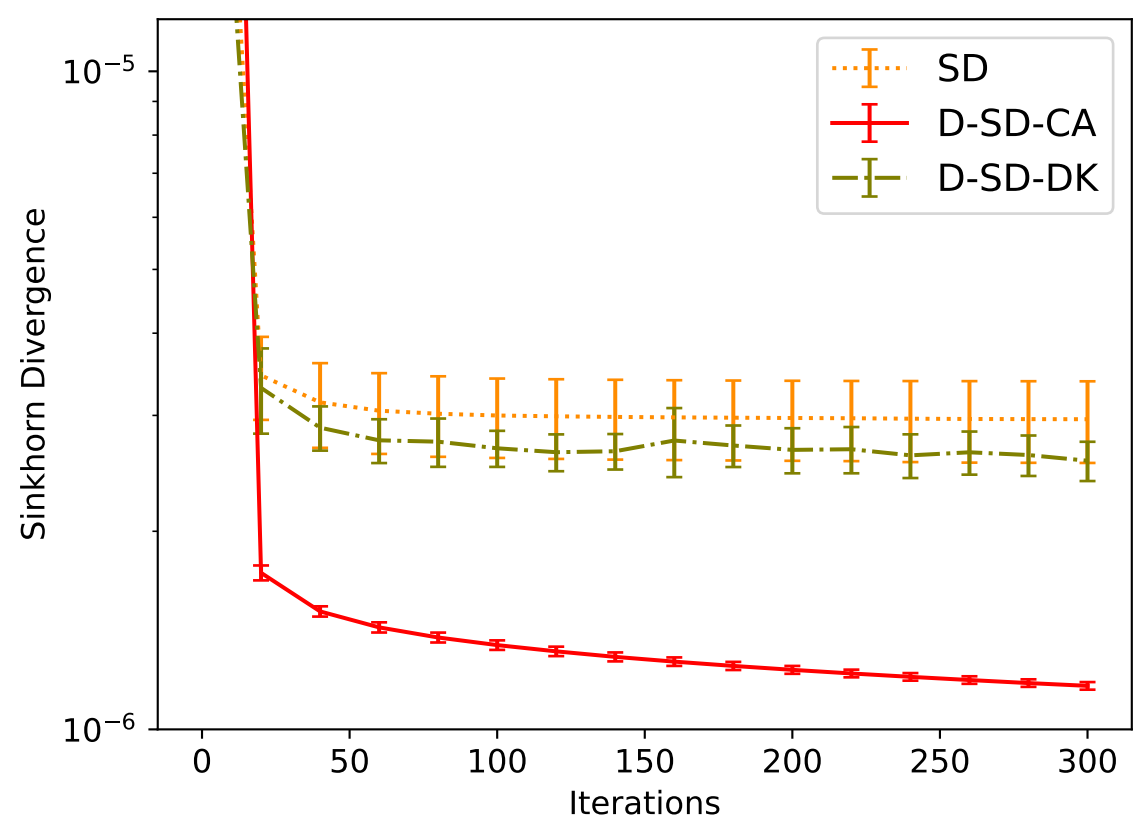

Supplement: Supplementary file 1 [file entropy-26-00679-s001.zip › dpvi_discrete-master/figures/sketching/sketching_iter.pdf]

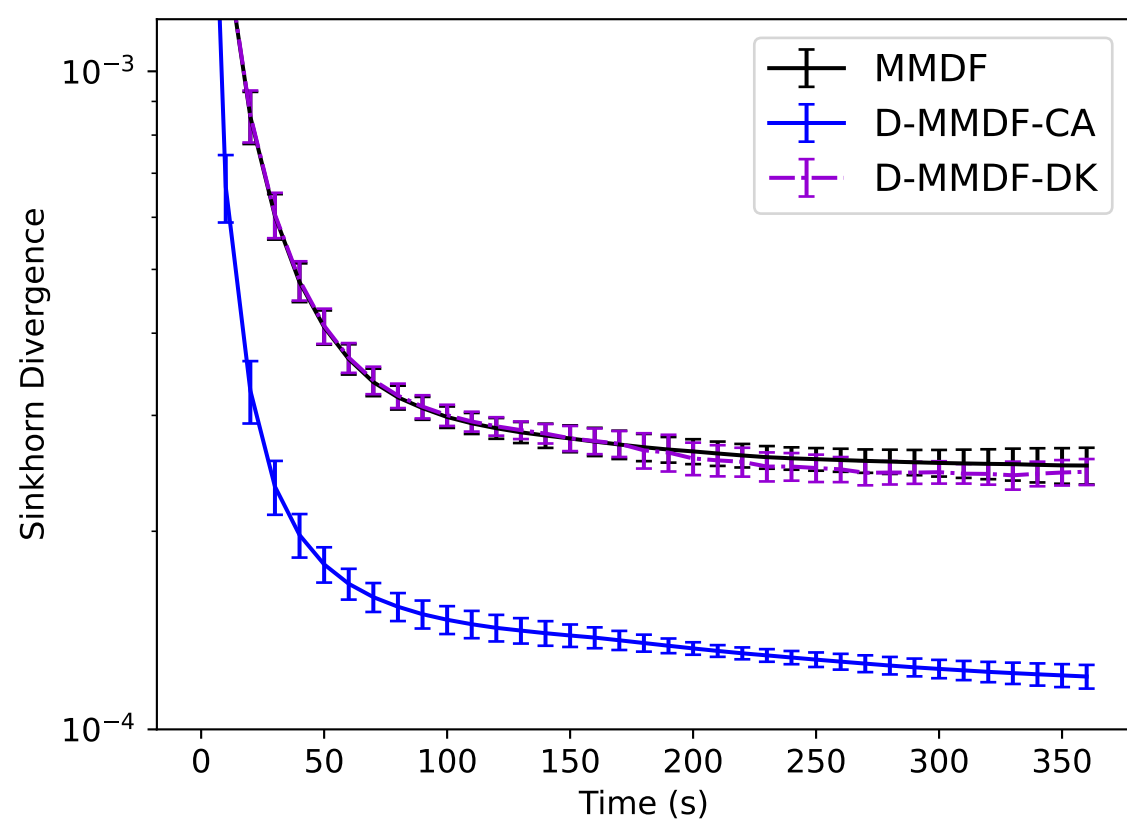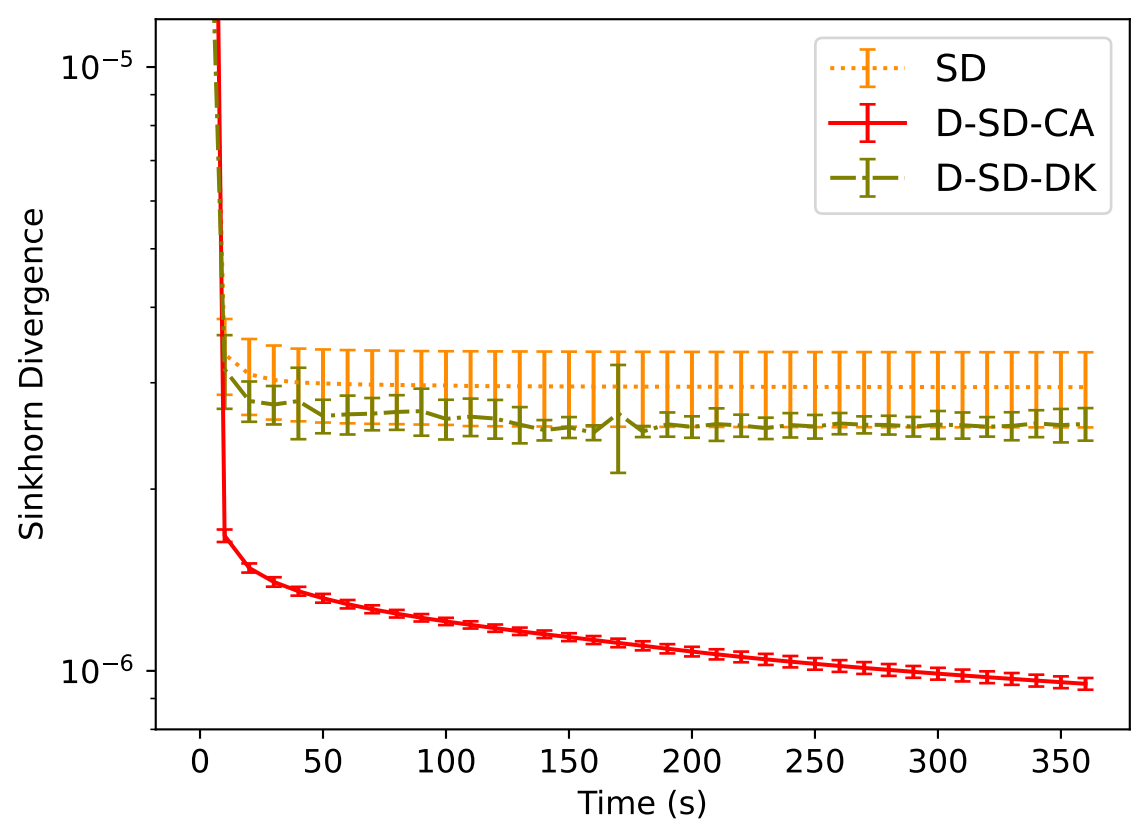

Supplement: Supplementary file 1 [file entropy-26-00679-s001.zip › dpvi_discrete-master/figures/sketching/sketching_time.pdf]

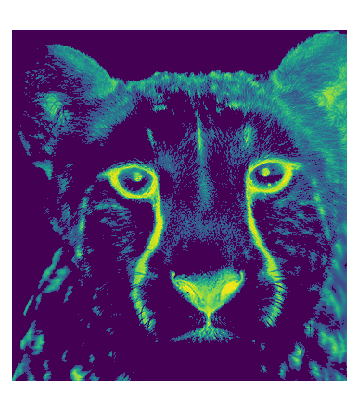

Supplement: Supplementary file 1 [file entropy-26-00679-s001.zip › dpvi_discrete-master/figures/sketching/target.png]

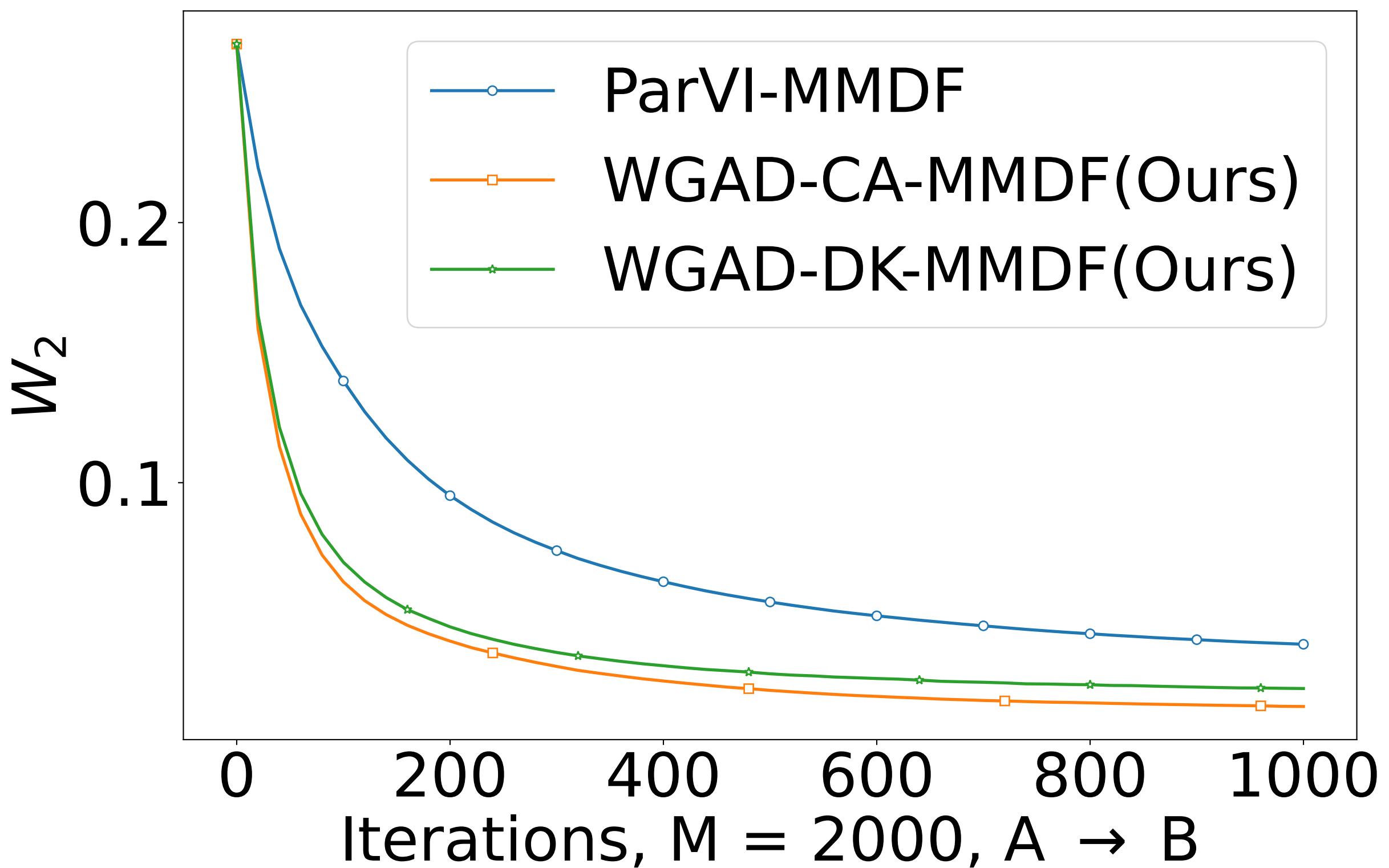

Supplement: Supplementary file 1 [file entropy-26-00679-s001.zip › dpvi_discrete-master/figures_morphing/5_figures_big/MMDF_w2AB.pdf]

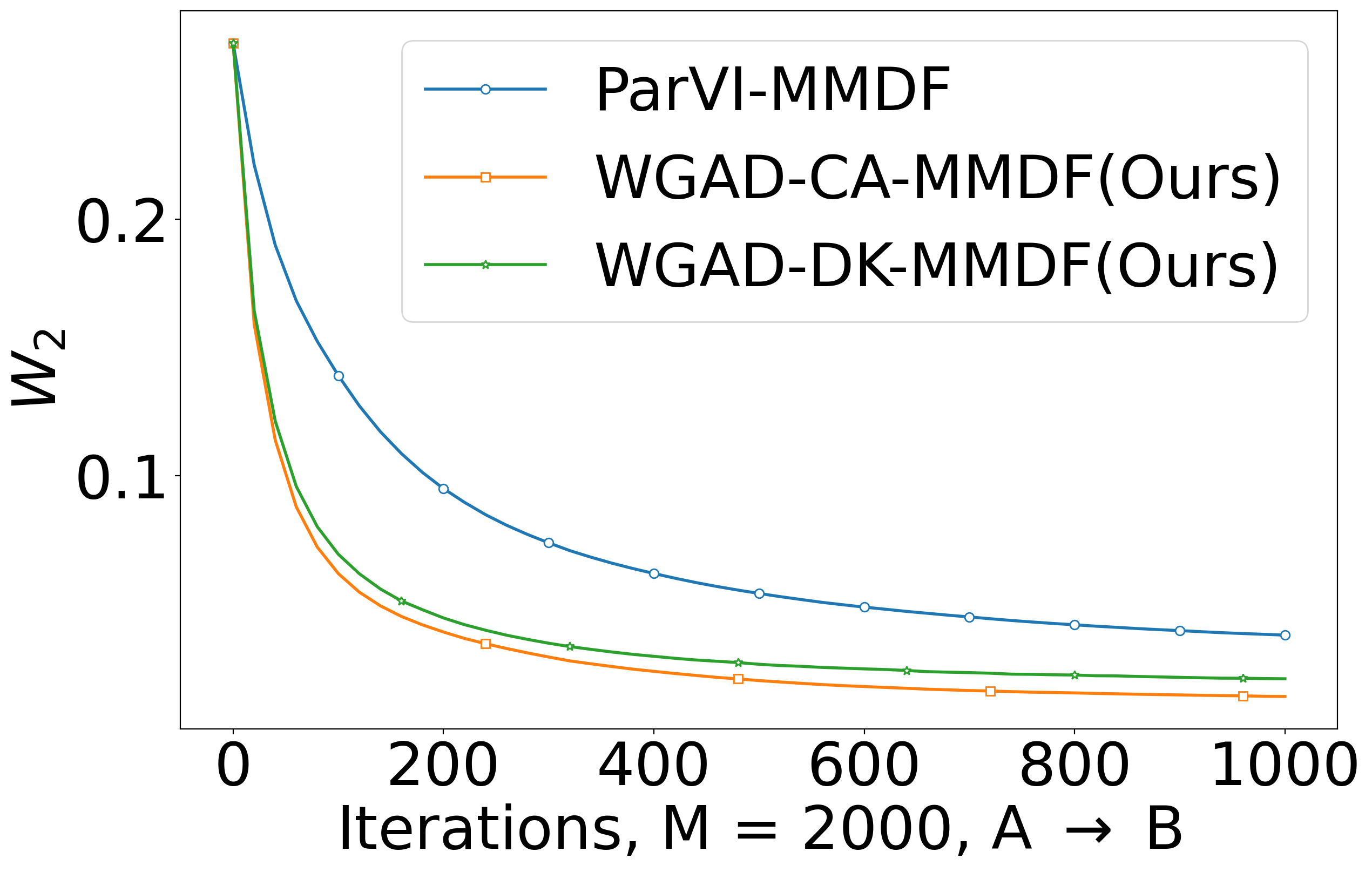

Supplement: Supplementary file 1 [file entropy-26-00679-s001.zip › dpvi_discrete-master/figures_morphing/5_figures_big/MMDF_w2AB.png]

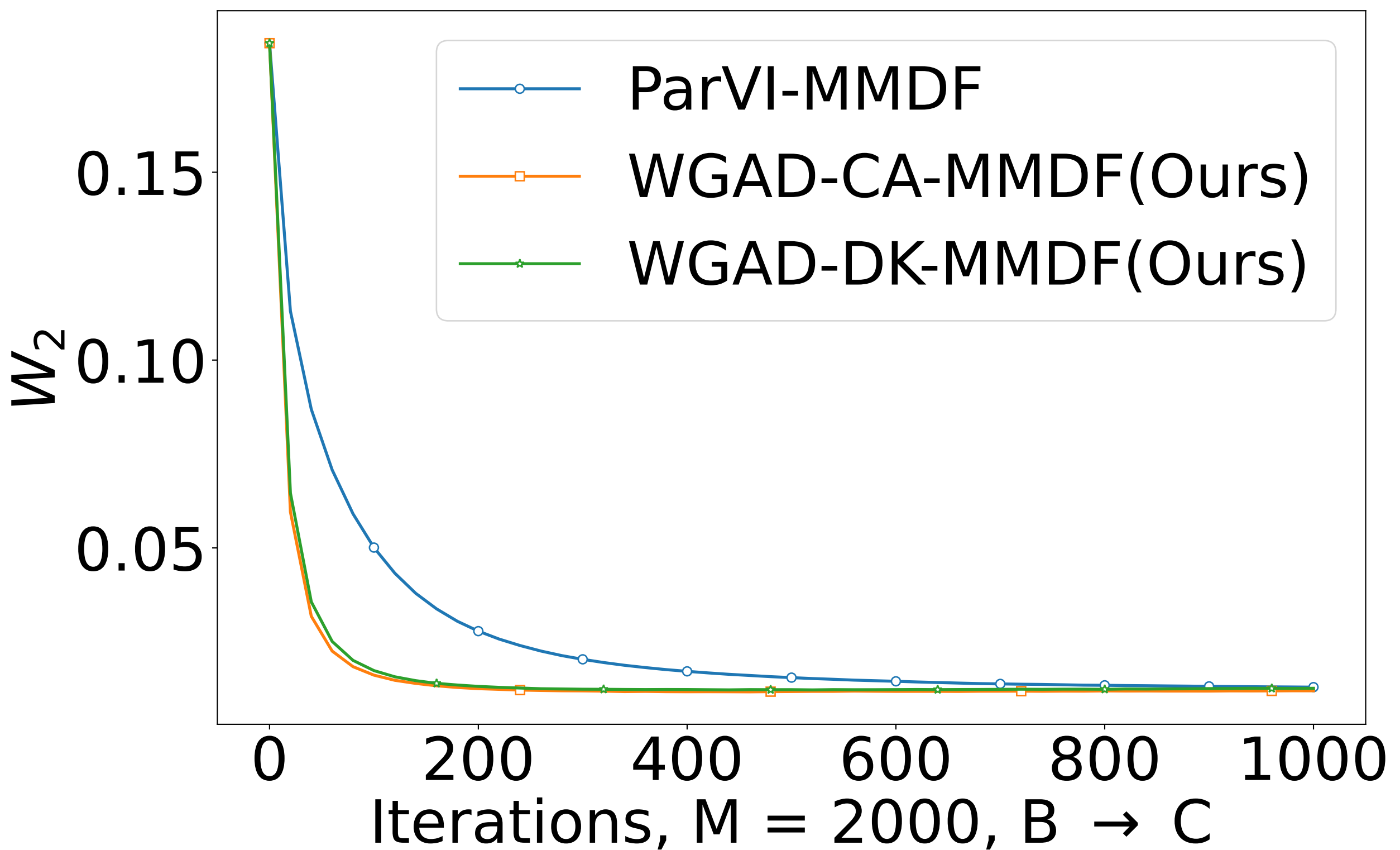

Supplement: Supplementary file 1 [file entropy-26-00679-s001.zip › dpvi_discrete-master/figures_morphing/5_figures_big/MMDF_w2BC.pdf]

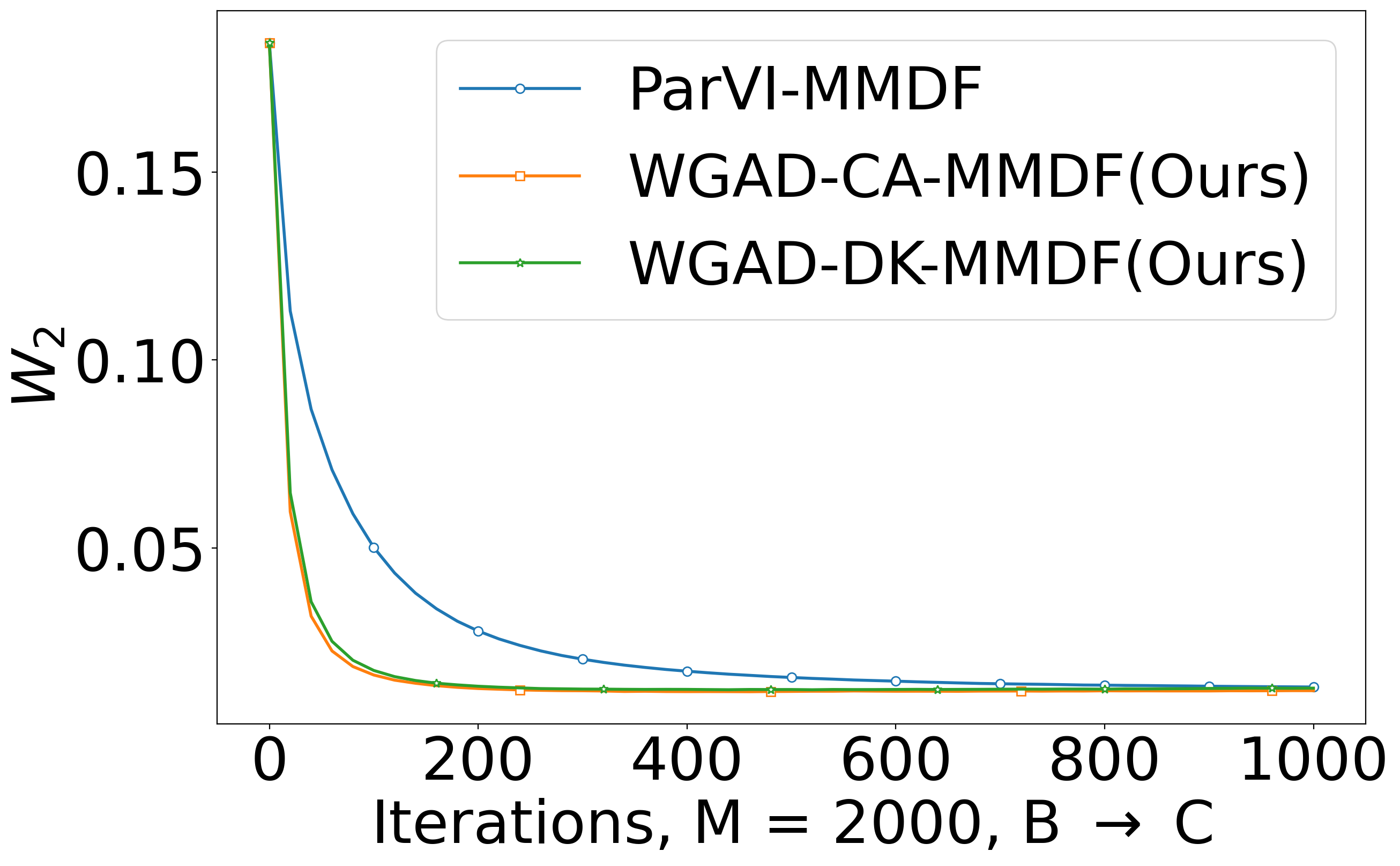

Supplement: Supplementary file 1 [file entropy-26-00679-s001.zip › dpvi_discrete-master/figures_morphing/5_figures_big/MMDF_w2BC.png]

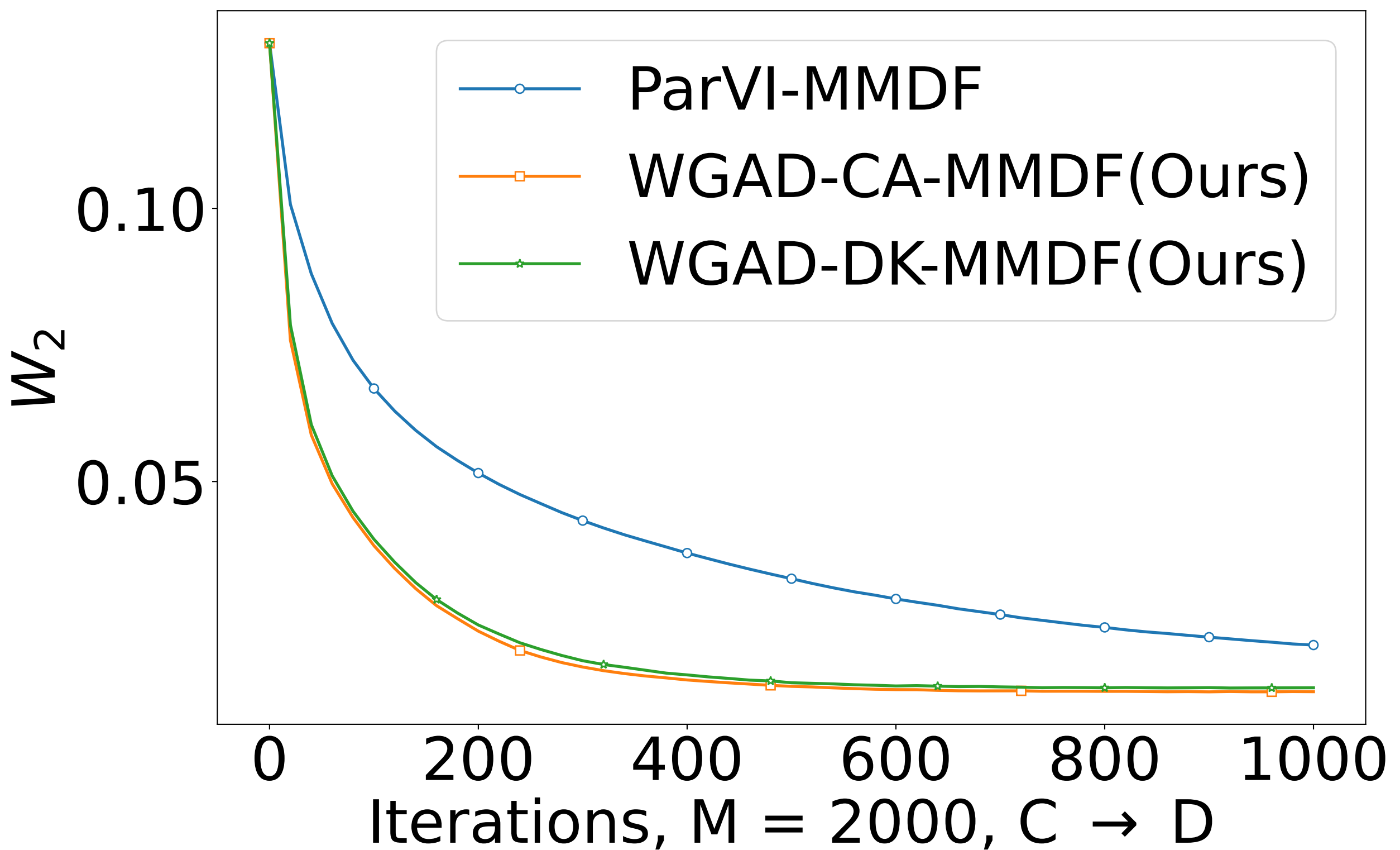

Supplement: Supplementary file 1 [file entropy-26-00679-s001.zip › dpvi_discrete-master/figures_morphing/5_figures_big/MMDF_w2CD.pdf]

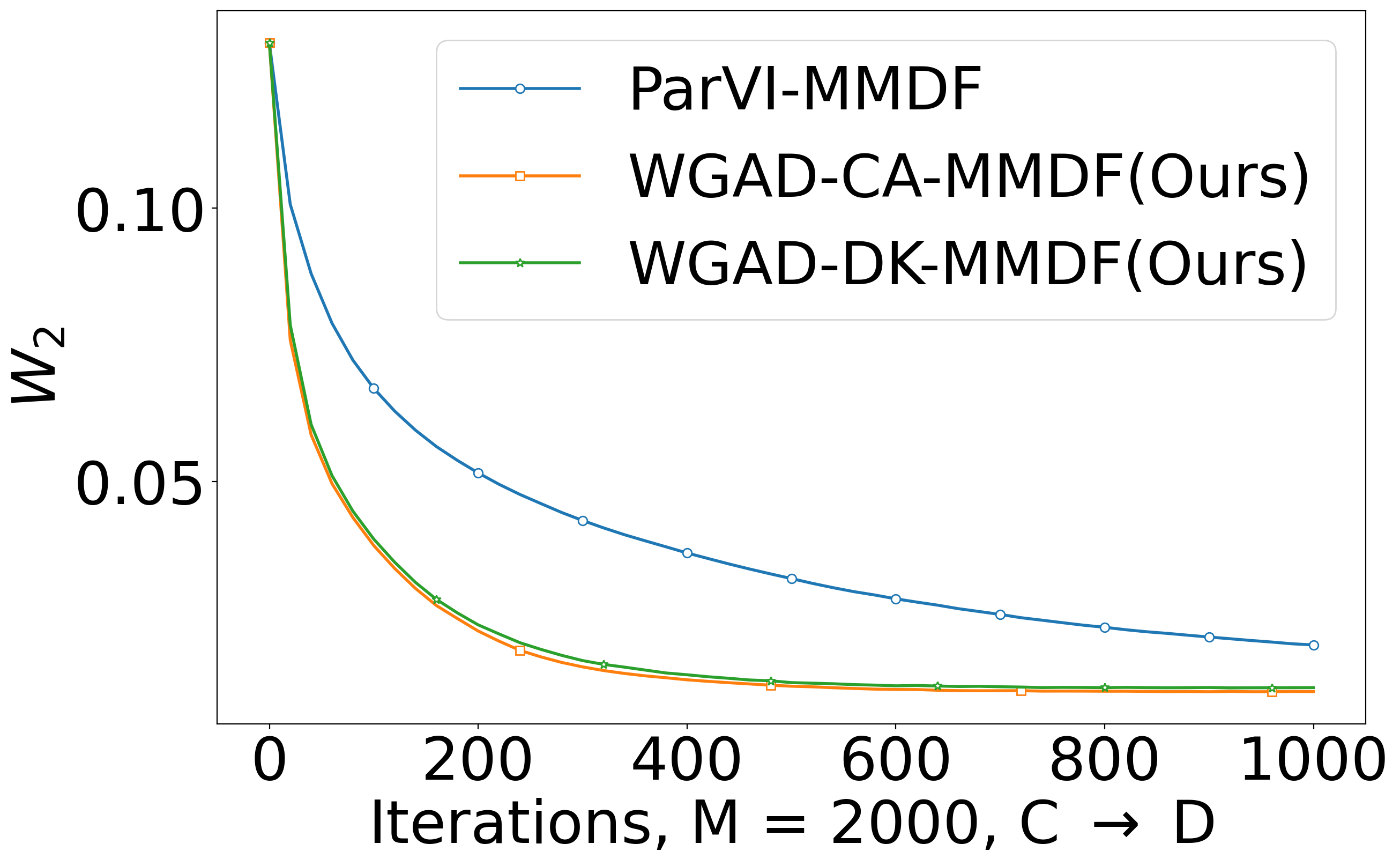

Supplement: Supplementary file 1 [file entropy-26-00679-s001.zip › dpvi_discrete-master/figures_morphing/5_figures_big/MMDF_w2CD.png]

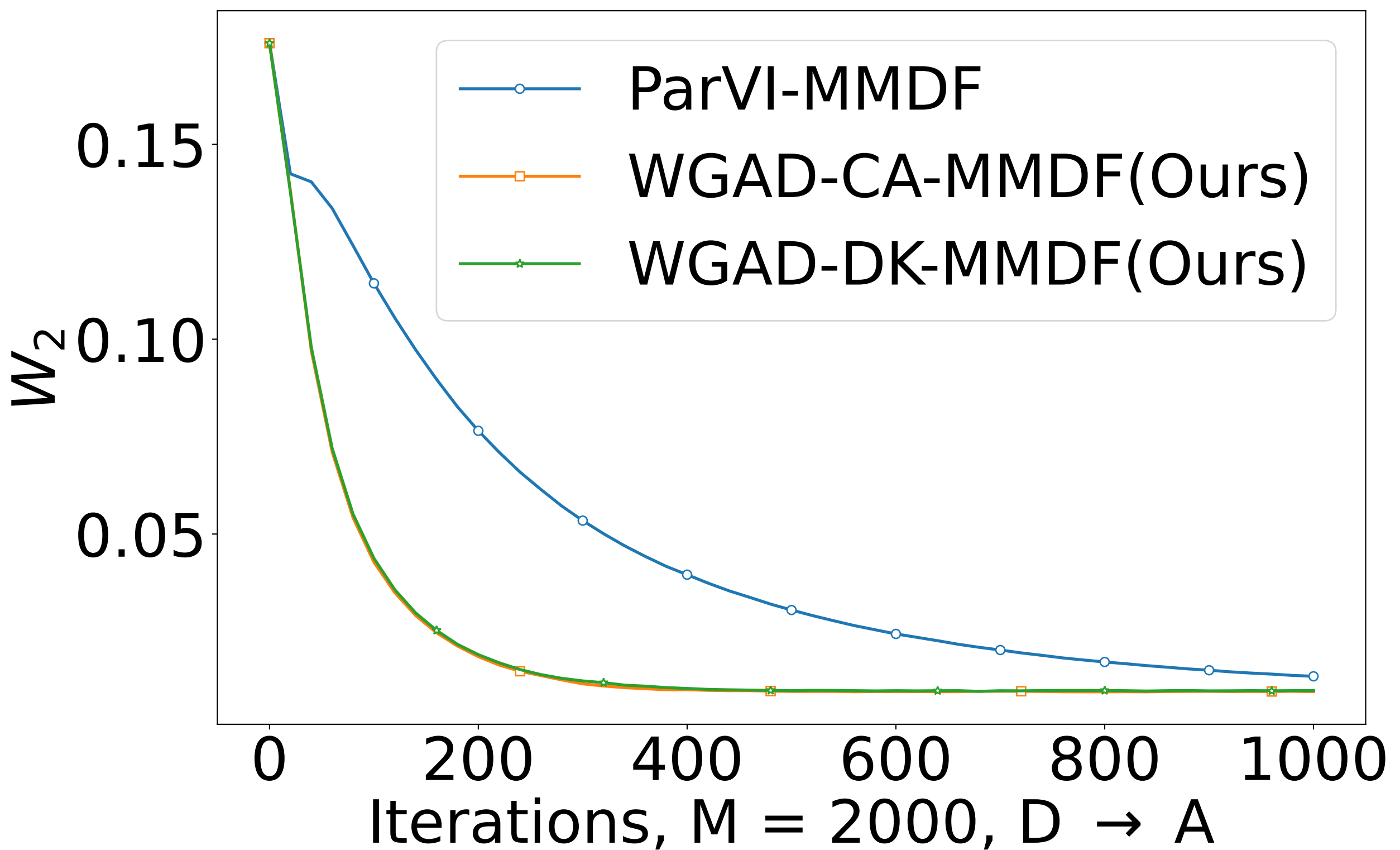

Supplement: Supplementary file 1 [file entropy-26-00679-s001.zip › dpvi_discrete-master/figures_morphing/5_figures_big/MMDF_w2DA.pdf]

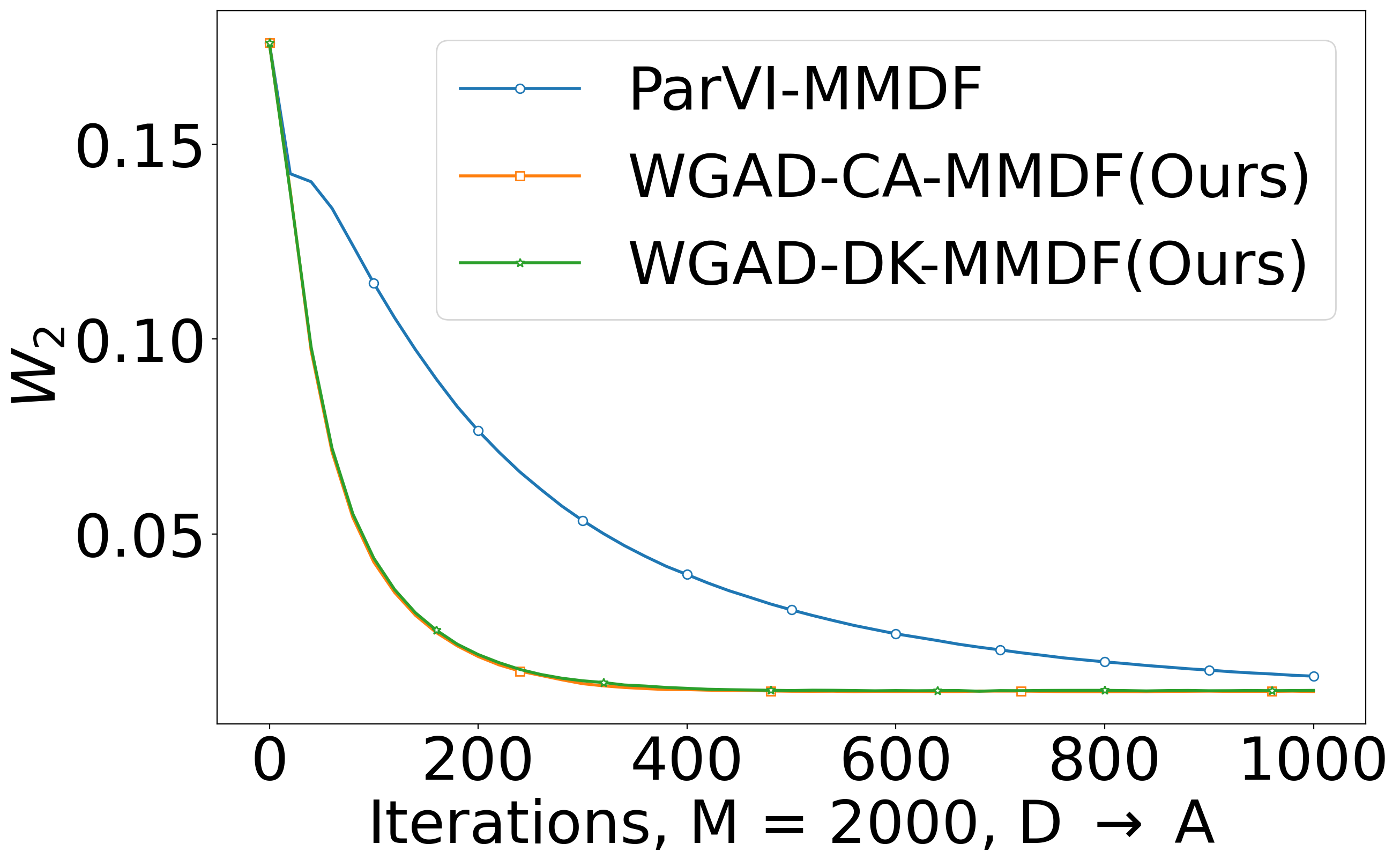

Supplement: Supplementary file 1 [file entropy-26-00679-s001.zip › dpvi_discrete-master/figures_morphing/5_figures_big/MMDF_w2DA.png]

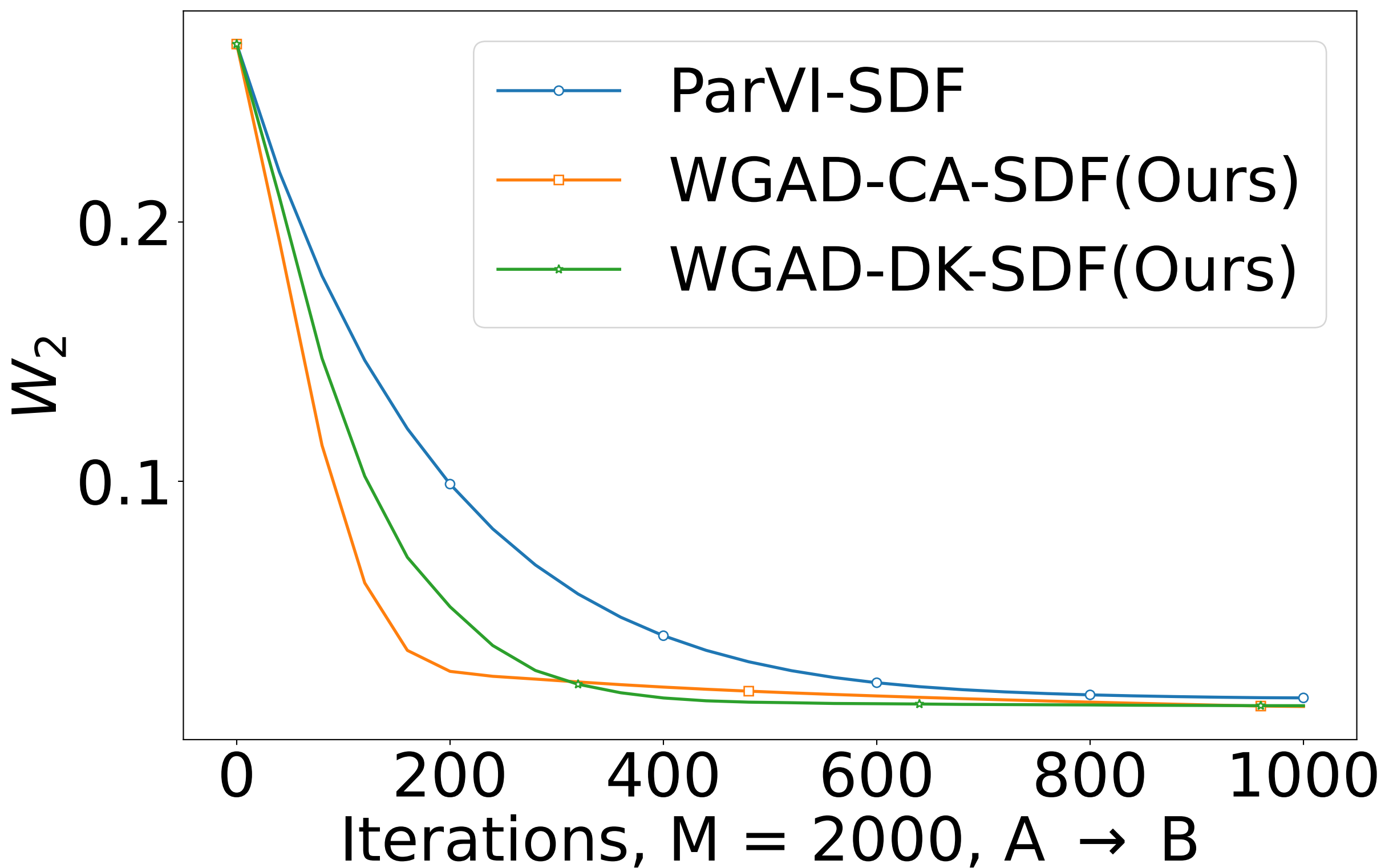

Supplement: Supplementary file 1 [file entropy-26-00679-s001.zip › dpvi_discrete-master/figures_morphing/5_figures_big/SD_w2AB.pdf]

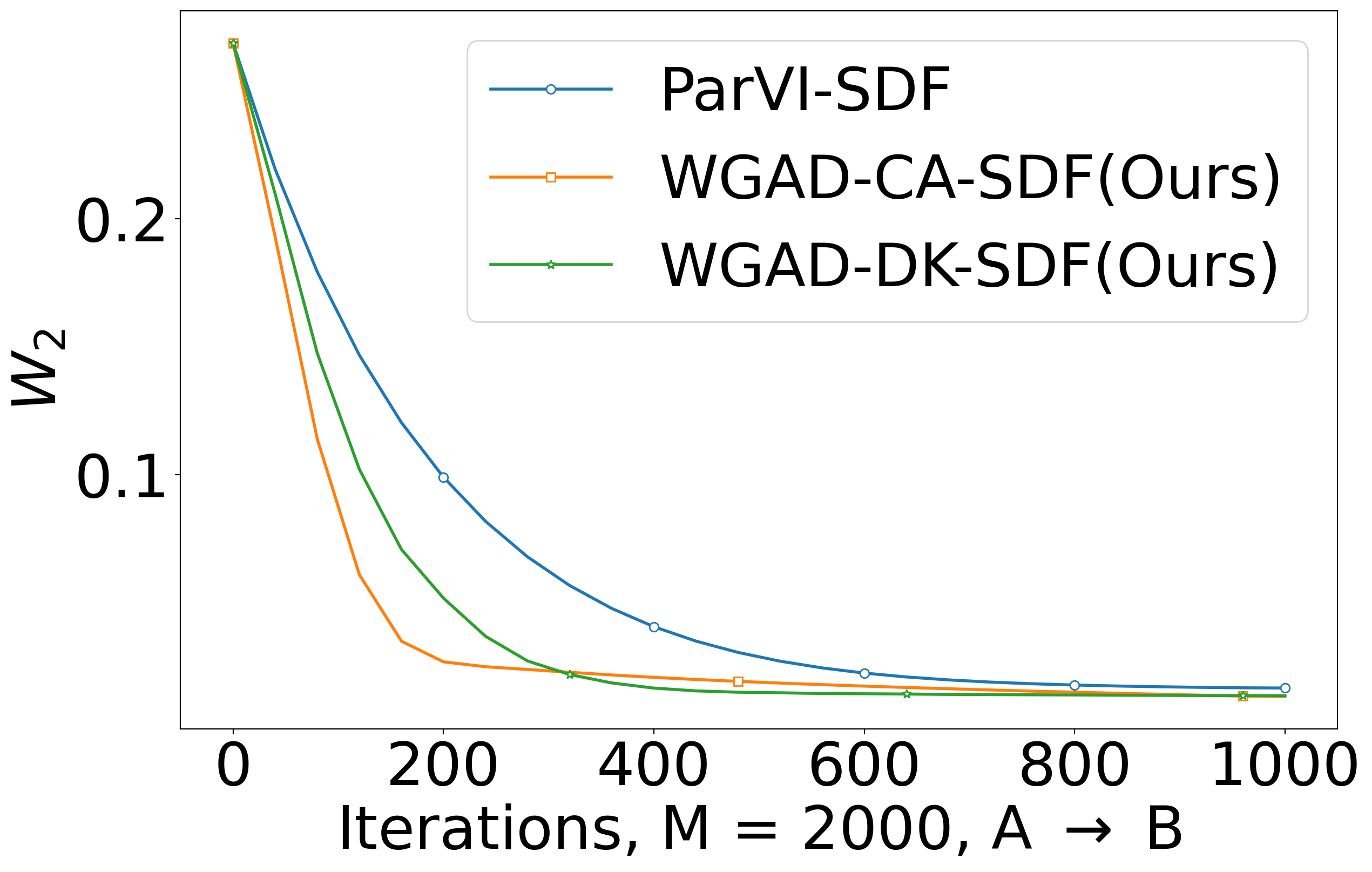

Supplement: Supplementary file 1 [file entropy-26-00679-s001.zip › dpvi_discrete-master/figures_morphing/5_figures_big/SD_w2AB.png]
